# *O*-prenylated 3-carboxycoumarins as a novel class of 15-LOX-1 inhibitors

**DOI:** 10.1371/journal.pone.0171789

**Published:** 2017-02-09

**Authors:** Atena Jabbari, Mina Mousavian, Seyed Mohamad Seyedi, Mehdi Bakavoli, Hamid Sadeghian

**Affiliations:** 1 Department of Chemistry, Faculty of Science, Ferdowsi University of Mashhad, Mashhad, Iran; 2 Department of Biology, Payame Noor University, Mashhad, Iran; 3 Neurogenic Inflammation Research Center, Mashhad University of Medical Sciences, Mashhad, Iran; 4 Department of Laboratory Sciences, School of Paramedical Sciences, Mashhad University of Medical Sciences, Mashhad, Iran; La Trobe University, AUSTRALIA

## Abstract

Allyloxy, Isopentenyloxy, geranyloxy and farnesyloxy derivatives of 3-carboxycoumarin, at position 5, 6, 7, and 8, were synthesized and their inhibitory potency against human 15-lipoxygenase-1 (human 15-LOX-1) were determined. Among the synthetic coumarins, *O*-allyl and *O*-isopentenyl derivatives demonstrated no considerable lipoxygenase inhibition while *O*-geranyl and *O*-farnesyl derivatives demonstrated potent inhibitory activity. 5-farnesyloxy-3-carboxycoumarin demonstrated the most potent inhibitory activity by IC_50_ = 0.74 μM while 6-farnesyloxy-3-carboxycoumarin was the weakest inhibitor among farnesyl analogs (IC_50_ = 10.4 μM). Bonding affinity of the designed molecular structures toward 15-LOX-1 3D structure complexed with RS75091, as potent 15-LOX-1 inhibitor, was studied by utilizing docking analysis. There was a direct relationship between lipoxygenase inhibitory potency and prenyl length chain. The ability of the prenyl portion to fill the lipophilic pocket which is formed by Ile663, Ala404, Arg403, Ile400, Ile173 and Phe167 side chains can explain the observed relationship. Similarity rate between the docked models and complexed form of RS75091, from point of view of configuration and conformation, could explain inhibitory potency variation between each prenyloxy substitution of 3-carboxycoumarins.

## Introduction

15-Lipoxygenase-1 (15-LOX-1) or reticulocyte 15-LOX is one of the most important enzyme of lipoxygenase family (5-LOX, 8-LOX, 12-LOX, 15-LOX-1 and 15-LOX-2) and for the first time, it was isolated from reticulocyte and eosinophil cells [1]. The activity of 15-lipoxygenase is greater in eosinophils when compared to other leukocytes by more than 100-fold [2].

In terms of aliphatic chain length, 15-LOX-1 shows wide substrate specificity: it can oxygenate unsaturated C18, C20, and C22 fatty acids [3]. The optimal substrate for 15-LOX-1 is linoleic acid [4], producing 13(S)-HPODE [5]. The reaction with arachidonic acid mainly forms 15(S)-HPETE and also 12(S)-HPETE as a side product [4, 5]. 15-LOX-1 is involved in the controlled degradation of reticulocyte mitochondria during the maturation of red cells [6]. It preferentially acts on mitochondrial membranes when compared with cell membranes, leading to the inactivation of respiratory enzymes [7]. It can change the mitochondrial pH gradient, dissipation of mitochondrial membrane potential, and finally the release of cytochrome c [8]. 15-LOX-1 protein expression is regulated during the maturation of red cells; it is not expressed in bone marrow, but the expression begins in the transition phase from late erythroblast to early reticulocyte stages [9].

With respect to atherogenesis, there are two considerable differences between 15-LOX-1 and other mammalian LOXes. As earlier mentioned, the optimal substrate for 15-LOX-1 is linoleic acid, which is abundant in LDL and it can also oxygenate fatty acid esters in more complex substrates such as cholesterol esters, phospholipids, lipoproteins, and biomembranes [4].

15-LOX-1 has been documented as a target for decreasing the biosynthesis of eoxines, one of the known pro-inflammatory mediators [10]. The 15-LOX-1 pathway has been demonstrated to generate eoxines in eosinophils, mast cells, and nasal polyps from allergic subjects, indicating that inhibition of 15-LOX-1 might be an attractive target for the treatment of inflammatory respiratory disorders such as asthma, rhinitis, and chronic obstructive pulmonary disease (COPD) in humans.

The critical role of the 15-LOX-1 metabolite (13-HODE) in the progression of prostate cancers and the inhibition of 15-LOX-1 activity for apoptosis induction in PC3 cells has been demonstrated. Human prostate tumors and prostate cancer cell lines express 15-LOX-1 and produce the 15-LOX-1 metabolite 13-HODE [11, 12].

15-LOX-1 can generate and develop atherosclerosis by oxidizing LDL cholesterol esters and phospholipids and its progression can be limited by some specific 15-LOX-1 inhibitors [1].

Human l5-LOX-1 may be one of the key mediators in neurodegenerative disease such as Alzheimer, because it is triggered by reactive oxygen species (ROS). Increased amounts of 15-LOX-1 have been found in experimental stroke in mice and in early phases of Alzheimer’s in humans. Cell culture studies have implicated l5-LOX-1 in neuronal models of oxidative stress related to Alzheimer’s [13] and Parkinson’s diseases [14].

The potential role of 15-LOX-1 in the development of obesity, particularly in adipocyte differentiation and the development of abdominal visceral obesity has also been documented [1].

Based on the above mentioned disorders, there is considerable interest in the development and evaluation of 15-LOX-1 inhibitors for therapeutic applications. In this study, based on our previous study on *O*-prenylated coumarins as 15-LOX inhibitors, [15] new derivatives of *O*-prenylated 3-carboxycoumarin involving farnesyloxy, geranyloxy and isopentenyloxy substituents at positions 5, 6, 7 and 8 of coumarin ring where designed, synthesized and their inhibitory potency against human 15-LOX-1 with SAR studies have been carried out.

## Results and discussion

**Fig 1:**
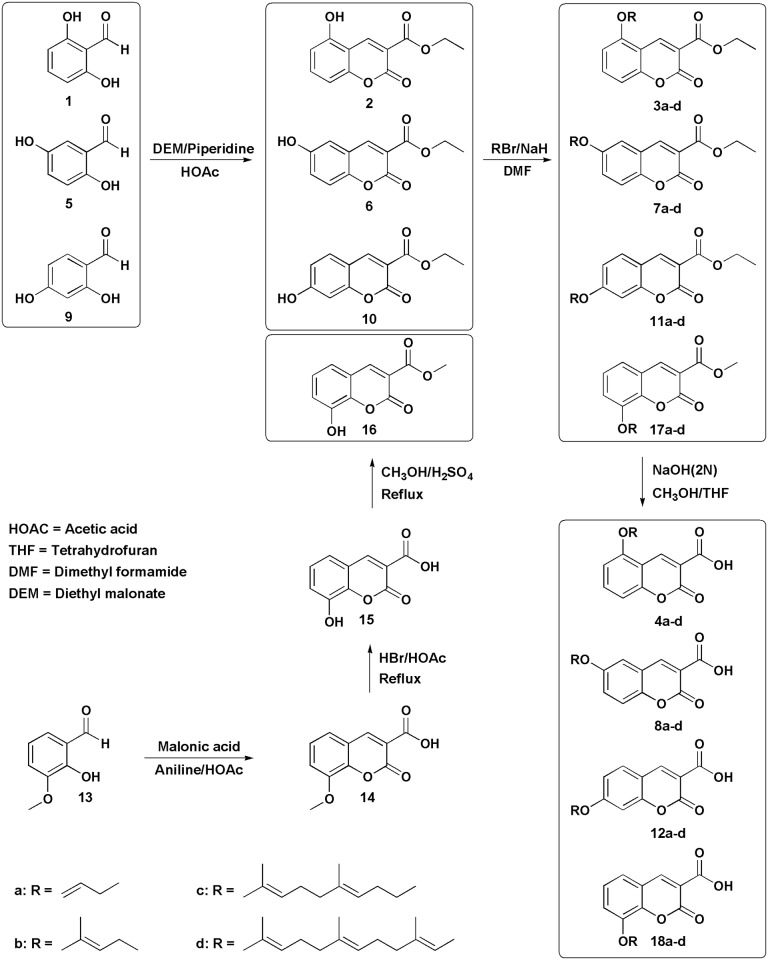
General procedure for the synthesis of *O*-prenylated-3-carboxycoumarins.

All of the *O*-prenylated 3-carboxycoumarins: **4a-d**, **8a-d**, **12a-d** and **18a-d** (Fig 1) were synthesized by the alkylation of ethyl or methyl ester of a hydroxycoumarin-3-carboxylic acid (compounds: **2**, **6**, **10** or **16**) with the desired prenyl bromide in the presence of sodium hydride in DMF and finally, the hydrolysis of the ester group by sodium hydroxide in a mixture of THF and methanol [16]. Compounds **2**, **6** and **10** were synthesized through Knoevenagel condensation of dihydroxybenzaldehyde (**1**, **5** and **9** respectively) with diethyl malonate [17] while compound **16** was prepared through esterification (with methanol) of demethylated form of coumarin **14**. Compound **14** was synthesized by Knoevenagel condensation of *ortho*-vanilline (**13**) and malonic acid [18] Synthesis of compound **16** starting from dihydroxybenzaldehyde leads to low yield (Fig 1).

The inhibitory activity of the synthetic compounds against human 15-LOX-1 was determined utilizing modified catalytic oxidative coupling of 3-methyl-2-benzothiazolinone (MBTH) with 3-(dimethylamino)benzoic acid (DMAB) as reported in previous studies [15, 19]. In this method, the basis for the determination of lipoxygenase activity is the measurement of peroxide concentration. Human 15-LOX-1 was prepared from isolated human eosinophil based on the procedure reported by Sigal et al [2]. In this procedure, 15-LOX-1 was partially purified utilizing ammonium sulfate precipitation method with activity of 104 nmol.mL^-1^.min^-1^ for 13(S)-HPODE formation. Because of the instability of the purified 15-LOX-1, the crude form of the enzyme was utilized [20]. Eosinophils from blood of hypereosinophilic donors (8–11% eosinophils) were purified to more than 85% through “hypotonic lysis and centrifugation” method reported by Samoszuk [21].

Among the synthetic coumarins, *O*-allyl and *O*-isopentenyl derivatives (**a** and **b** series) demonstrated no considerable lipoxygenase inhibition while *O*-geranyl and *O*-farnesyl derivatives (**c** and **d** series) demonstrated potent inhibitory activity at IC_50_ value below 11 μM compared to 4-MMPB (IC_50_ = 16.8 μM) (Table 1), as standard 15-LOX inhibitors [19, 22]. In all, 5-*O*-prenylated coumarins: **4a**, **4b**, **4c** and **4d**, demonstrated the best inhibitory activity at IC_50_ values of 93.5, 62.5, 4.8 and 0.74 μM, in contrast with their related isomers from each of the synthetic groups (**4**, **8**, **12** and **18**), respectively.

**Table 1:** Inhibitory assessment data of the synthetic compounds in comparison with 4-methyl-2-(4-methylpiperazinyl)pyrimido[4, 5-b]benzothiazine (4-MMPB) against human 15-LOX-1. The data are shown as ± SD (n = 3).

Compound	IC_50_	Compound	IC_50_
**4a**	**93.5 ± 7.6**	**18c**	**20.4 ± 2.2**
**8a**	**296.1 ± 26.3**	**4d**	**0.74 ± 0.05**
**12a**	**114.2 ± 10.1**	**8d**	**10.4 ± 2.1**
**18a**	**293.3 ± 20.3**	**12d**	**8.0 ± 0.51**
**4b**	**62.5 ± 5.9**	**18d**	**5.6 ± 0.54**
**8b**	**227.2 ± 22.1**	**4-MMPB**	**16.8 ± 1.1**
**12b**	**81.3 ± 5.3**	**4d'**	**1.6 ± 0.08**
**18b**	**263.1 ± 23.0**	**8d'**	**1.0 ± 0.06**
**4c**	**4.8 ± 0.55**	**12d'**	**3.9 ± 0.28**
**8c**	**21.5 ± 1.6**	**18d'**	**3.2 ± 0.23**
**12c**	**12.1 ± 0.91**		

By using the DPPH bleaching test, no radical scavenging activity was observed for the synthetic coumarins up to 250 μM when compared with NDGA (nordihydroguaiaretic acid) and ascorbic acid (50 μM).

**Fig 2:**
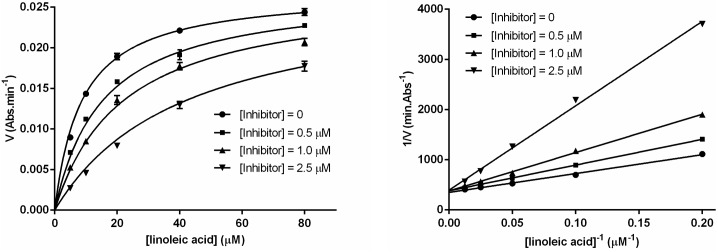
Michaelis-Menten (left) and Lineweaver-Burk (right) plots of human 15-LOX-1 inhibition by 4d. The Y-intercept average (1/V_max_) of the Lineweaver-Burk plot is 376 ± 45 min.Abs^-1^ and K_M_ = 8.73 ± 0.43 μM. The error bars are stated as ± SD (n = 4).

Determination of the type of enzyme inhibition using Lineweaver–Burk plot showed that the mentioned compounds inhibit lipoxygenase activity by competitive mechanism (Fig 2).

In order to find more information about the mechanism of action of the inhibitors, molecular models of the enzyme-inhibitor complex were simulated for the synthetic coumarins utilizing X-ray 3D structures of rabbit 15-LOX-1 (PDB entry: 2P0M). There is acceptable homology between the human 15-LOX-1 and rabbit 15-LOX-1 (positives: 91%, identities: 81%, extracted from NCBI-BLAST) [23]. This homology increases to 99% around 20 Å in the active site pocket. Therefore, the 3D structure of rabbit 15-LOX-1 was utilized instead of human type in docking analysis. For docking process, the Fe^II^ core of the 2P0M was modified to Fe^III^-OH [15] (It is the natural form of the lipoxygenase iron core before initiating the catalytic reaction) [24, 25].

Bonding affinity of the designed molecular structures toward rabbit 15-LOX-1 was studied. Docked conformers were generated in AutoDockTools (ADT) software. In docking process, flexible side chain of the active site pocket residues of rabbit 15-LOX-1 were allowed to be rotatable based on previous study: Phe353, Leu408, Phe415, Ile418, Met419, Ile593, Leu597.[15]

**Fig 3:**
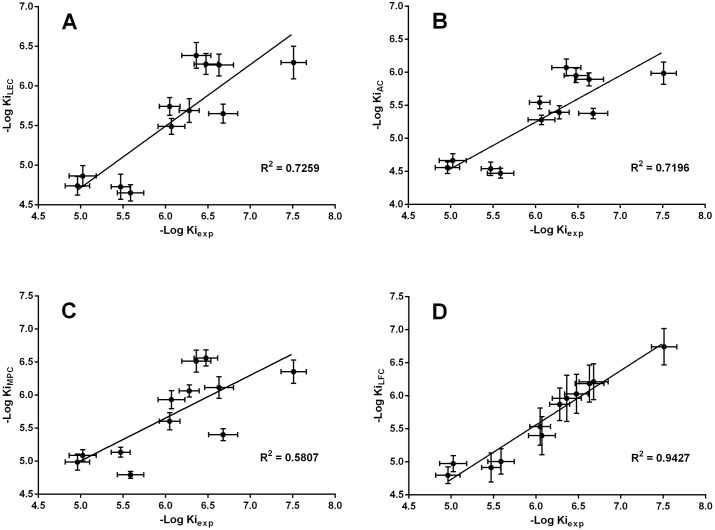
The four diagrams show the variations of -Log of Ki_LFC_ (A), Ki_AC_ (B), Ki_MPC_ and Ki_LFC_ versus -Log Ki_exp_. The data are presented as ± SEM.

To perform better analysis on docking results, the average Ki (estimated inhibitory constant) of the most populated cluster (Ki_MPC_), average of all the lowest Ki from each cluster (Ki_LEC_), average Ki of all the conformers (Ki_AC_) and average Ki of a cluster in which lactone portion of coumarin directed towards Fe-OH core (Ki_LFC_), were calculated for each compound (Table 2). The aforementioned inhibitory constants were easily calculated from docked enzyme-inhibitor binding free energy (ΔG_b_) by using of the Gibbs equation: ΔG_b_ = 2.3RTLogKi. For LFC if there was more than one cluster with the same situation (for each compound), one with the lowest average Ki was utilized for analysis. Among the four clusters, there was only an acceptable convergence between Ki_LFC_ and Ki_exp_ results with R-square of 0.94 (Fig 3). This convergence was significantly observed for farnesyl and geranyl derivatives. Ki_exp_ was calculated by using of the Cheng-Prusoff equation which has been defined for competitive inhibition: Ki_exp_ = IC_50_/(1+[S]/Km); Km = 8.73 μM, [S] = 200 μM.

**Table 2:** Data of the docking analyses results: Ki of the most populated cluster (Ki_MPC_), average of all the lowest Ki from each cluster (Ki_LEC_), average Ki of all the conformers (Ki_AC_) and average Ki of a cluster in which lactone portion of coumarin directed towards Fe-OH core (Ki_LFC_). N = number of conformers. The data are shown as ± SEM.

Compd.	Log Ki_MPC_	N	Log Ki_LFC_	N	Log Ki_AC_	N	Log Ki_LEC_	N
**4b**	**-4.794 ± 0.0523**	**33**	**-5.006 ± 0.1909**	**18**	**-4.473 ± 0.0747**	**200**	**-4.652 ± 0.1023**	**32**
**4c**	**-5.402 ± 0.0899**	**35**	**-6.213 ± 0.2720**	**12**	**-5.377 ± 0.0785**	**200**	**-5.651 ± 0.1186**	**35**
**4d**	**-6.356 ± 0.1760**	**14**	**-6.742 ± 0.2752**	**12**	**-5.986 ± 0.1677**	**200**	**-6.296 ± 0.2044**	**32**
**8b**	**-5.088 ± 0.0859**	**43**	**-4.974 ± 0.1216**	**32**	**-4.666 ± 0.1038**	**200**	**-4.865 ± 0.1299**	**30**
**8c**	**-5.605 ± 0.1303**	**21**	**-5.534 ± 0.2822**	**13**	**-5.544 ± 0.0946**	**200**	**-5.742 ± 0.1125**	**42**
**8d**	**-6.515 ± 0.1665**	**19**	**-5.962 ± 0.3501**	**12**	**-6.073 ± 0.1281**	**200**	**-6.386 ± 0.1623**	**43**
**12b**	**-5.136 ± 0.0753**	**48**	**-4.914 ± 0.2199**	**27**	**-4.541 ± 0.1013**	**200**	**-4.729 ± 0.1575**	**26**
**12c**	**-6.064 ± 0.0916**	**38**	**-5.871 ± 0.2466**	**13**	**-5.395 ± 0.1011**	**200**	**-5.690 ± 0.1498**	**33**
**12d**	**-6.563 ± 0.1230**	**36**	**-6.029 ± 0.2950**	**15**	**-5.951 ± 0.1059**	**200**	**-6.277 ± 0.1316**	**43**
**18b**	**-4.987 ± 0.1229**	**37**	**-4.798 ± 0.1264**	**24**	**-4.558 ± 0.0886**	**200**	**-4.741 ± 0.1166**	**37**
**18c**	**-5.932 ± 0.1365**	**26**	**-5.397 ± 0.2870**	**12**	**-5.280 ± 0.0708**	**200**	**-5.490 ± 0.1006**	**49**
**18d**	**-6.116 ± 0.1639**	**23**	**-6.186 ± 0.2802**	**15**	**-5.894 ± 0.0976**	**200**	**-6.264 ± 0.1373**	**42**

**Fig 4:**
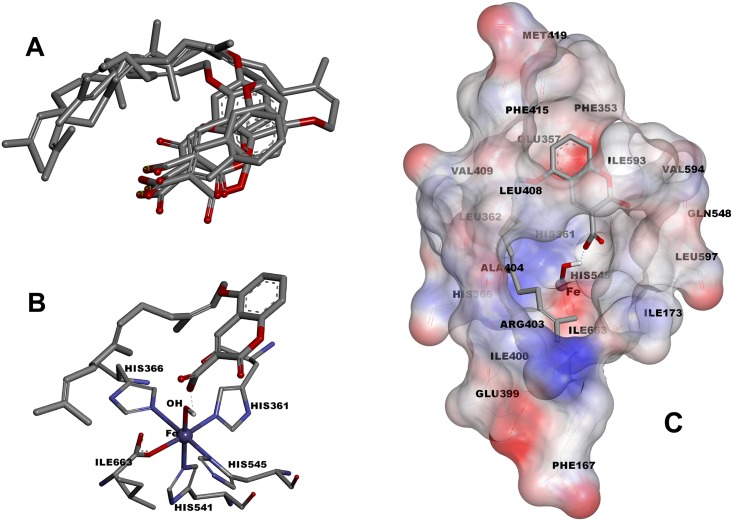
(A) Stick view of bonding conformer with the lowest Ki which relates to the compounds 4d, 8d, 12d and 18d from the LFC cluster (consensus structures). (B) Stick view of consensus structure of compound 4d and its hydrogen bonding (dash line) with Fe-OH core. (C) Stick and solvent surface view of the rabbit 15-LOX-1 active site residues interactions with the consensus structure of 4d.

In the earlier mentioned cluster (LFC), most of the conformers have hydrogen bonds with Fe-OH core through their carboxylic acid moiety and their prenyl portion are covered by side chain of Leu173, Leu362, His363, His366 Ile400, Arg403, Ala404, Leu408, Val409, Phe415 and Ile663 (Fig 4). In the above mentioned cluster, the coumarin rings are surrounded by Phe353, Glu357, Met419, Gln548, Ile593, Val594, and Leu597 (Fig 4). Among all the conformers in a cluster, the one with the least Ki was named as ‘consensus structure’ (Fig 4). The results imply that the hydrogen bonding between Fe-OH core and carboxylic acid portion of the coumarin ring beside the hydrophobic interaction of prenyl moiety with the side chain of lipophilic residues are the major factors in managing the inhibitory potency of the mentioned compounds.

**Fig 5:**
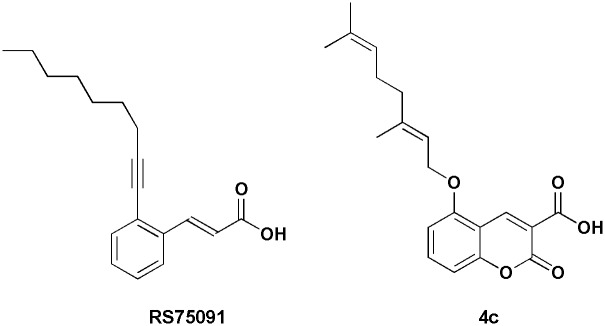
Comparison between the chemical structure of compounds RS75091 and 4c.

It is interesting to note that the mentioned conformation of the docked inhibitors are similar to RS75091 ((2*E*)-3-(2-oct-1-yn-1-ylphenyl)acrylic acid) complex with Rabbit 15-LOX-1 in the X-ray 3D of 2P0M [26]. This similarity is highly considerable for compound **4c** (Fig 5).

**Fig 6:**
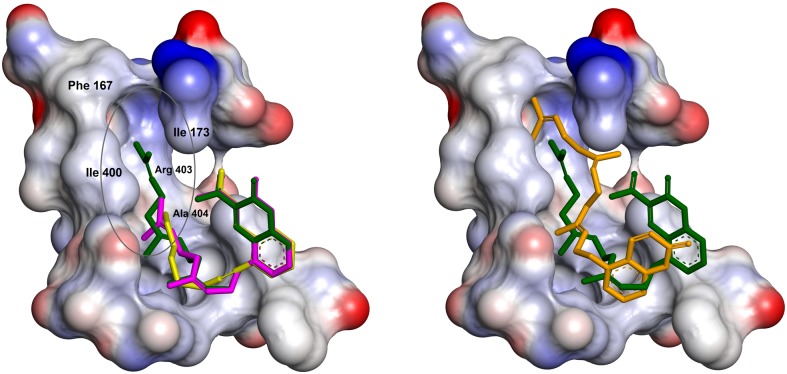
Superimposition of consensus structures of compounds 4c (pink stick) and 4d (green stick) from LFC on RS75091 (yellow stick) in rabbit 15-LOX-1 active site (left). Superimposition of consensus structure of 4d (green stick) from LFC on consensus structure of 4d' (brown stick) from similar cluster (right). In both figures, Ile663 is not shown.

When the consensus structure of **4c** and **4d** from the desired cluster (LFC) are superimposed on the 3D structure of 2P0M-RS75091 complex, a high similarity is seen between the situations of **4c**, **4d** and RS75091 especially for coumarin and acrylic moieties (Fig 6 - left). The space occupancy of geranyl moiety of **4c** is similar to octynyl portion of RS75091. This occupancy is formed in the cavity shaped by side chain of Ile663, Ala404, Arg403, Ile400, Ile173 and Phe167. The main part of the cavity is not occupied by aliphatic portions of **4c** and RS75091 but most of this lipophilic space is filled by farnesyl moiety of **4d** (Fig 6 - left). The similarity rate between the docked models and complexed form of RS75091, from the point of view of configuration and conformation, could be another reason for the explanation of lipoxygenase inhibitory potency variation of the synthetic 3-carboxycoumarins. In addition, the ability of the prenyl portion of the compounds to fill the lipophilic pocket which is formed by Ile663, Ala404, Arg403 (butyl portion of the Arg side chain), Ile400, Ile173 and Phe167 side chains can explain the direct relationship between lipoxygenase inhibition potency and prenyl length chain. The importance of the above mentioned cavity for substrate binding in mammalian 15-lipoxygenase and also the effective role of Arg403, Ile400 and Ile173 in its functionality has been well documented in previous studies [25, 26].

**Fig 7:**
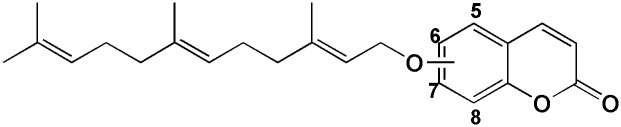
General structure of compounds 4d', 8d', 12d' and 18d' which relates to farnesyloxy substitution at position 5, 6, 7 and 8 respectively.

To find the effect of carboxylic acid moiety on inhibitory potency, lipoxygenase inhibition of the synthetic compounds was also compared to the related analogs with no carboxylic acid substituent. For this purpose, lipoxygenase inhibitory of O-farnesyl derivatives of 5-, 6-, 7-, and 8-hydroxycoumarin (**4d'**, **8d'**, **12d'** and **18d'** respectively), and their inhibitory activity against 15-LOX reported in previous study,[15] were measured in comparison with the present compounds (Fig 7). It was interesting to note that except **4d**, the other farnesyl derivative had less lipoxygenase inhibitory potency when compared to the corresponding carboxylate-off analogs by 1.5 to 10 folds. **4d** with farnesyloxy substituent at position 5 had higher lipoxygenase inhibitory activity by 2 folds while **8d** with 6-farnesyloxy substituent showed lower activity by 10 folds when compared to **4d'** and **8d'**.

The situation of coumarin moiety of the docked conformers of *O*-prenylated 3-carboxycoumarins from the mentioned cluster are different from those reported for carboxylate-off analogs in previous study [15], while it is similar for the prenyl portions. In both clusters, prenyl chains are situated in a hydrophobic pocket which is formed by side chain of Ile663, Ala404, Arg403, Ile400, Ile173 and Phe167 (Fig 6 - right). The observed similarity demonstrates the important role of the earlier mentioned hydrophobic pocket for 15-LOX inhibition and it can serve as a lead for designing of new inhibitors.

In spite of reduction in lipoxygenase inhibitory potency of farnesyloxy 3-carboxycoumarins in comparison with the related farnesyloxy coumarin (with exception of compound **4d**), it must be considered that the present compounds are soluble in water at physiological pH (pH > 7) due to their carboxylic acid moiety. The above mentioned property can be valuable from the point of view of drug delivery and drug metabolism [27].

## Materials and methods

### Instruments

The IR spectra were obtained on a 4300 Shimadzu Spectrometer. ^1^H NMR (300 MHz) was obtained by using a Bruker Avance DRX-300 and -400 Fourier transformer spectrometer. Chemical shifts are reported in parts per million (δ) downfield from tetramethylsilane (TMS). The mass spectra were scanned on a Varian Mat CH-7 instrument at 70 eV. Elemental analysis was obtained on a Thermo Finnigan Flash EA microanalyzer. Sonication was down by BANDELIN SONOPULS mini20. All measurements of DPPH bleaching and lipoxygenase activities were carried out using BioTek Synergy HTX Multi-Mode reader. Chemicals were purchased from Aldrich and Merck Co.

### Structure optimization

The desired structures were drawn in ChemDraw Ultra 8.0. [28] Then the 2D structures were exported to the HyperChem 8.07 software and geometrically optimized using classic MM+ (RMS gradient = 0.05 kcal mol^-1^) [29] and semi-empirical AM2 methods (convergence limit = 0.01; Iteration limit = 50; RMS gradient = 0.05 kcal mol^-1^; Polak-Ribiere optimizer algorithm) to simulating of the 3D structures.

Crystal structure of Rabbit 15-lipoxygenase-1 (Rabbit 15-LO-1) complex with RS75091 was retrieved from RCSB Protein Data Bank (PDB entry: 2P0M).

### Molecular docking

The ligand of Rabbit 15-LO-1 3D structure was omitted. Then the Fe was modified to Fe^III^-OH, geometrically optimized by MM+ method in HyperChem8.0 and outputted in pdb format for docking process [15].

Docking of the minimized structures into the active site of 2P0M was down by AutoDock 4.2. [30] The torsion angles of the ligands were identified, bond distances were edited, hydrogens and solvent parameters were added to the enzyme 3D structure. Partial atomic charges were then assigned to the macromolecule as well as ligands (Gasteiger for the ligands and Kollman for the protein).

The docking regions of the enzyme were defined by considering Cartesian chart -53.10, 166.95 and 35.82 as the central of a grid size with 68, 52 and 66 points in X, Y and Z axis. The docking parameter files were generated using Lamarckian genetic algorithm Parameters (GALS) while number of generations and maximum number of energy evaluations was set to 200 and 2,500,000, respectively. The 200 docked complexes were clustered with a root-mean-square deviation tolerance (RMSD) of 2.0 Å. Docking results were submitted to Accelrys Discovery Studio v3.1 [31] for further evaluations.

### Enzyme preparation

100 mL of EDTA-anticoagulated blood (800–1100 eosinophils/μL) was added to 2000 mL of distilled water and slowly inverted (repeatedly) for 1 min, after which 200 mL of 10X phosphate-buffer saline was added. The mixture was then centrifuged at 1200 g (5 min), and the supernatant was aspirated. The tan pellet of eosinophils was suspended in 50 mL of distilled water and incubated for a minute, followed by the addition of 5 mL of neutralizing buffer (Tris 1M; pH 7.4). The suspension was centrifuged at 1200 g (5 min) and then washed with normal saline solution and centrifuged at 1200 g (5 min) [21].

The eosinophils pellet (~10^8^ cells; purity > 87%) was suspended in 4 mL potassium phosphate buffer (10 mM; pH 7.0) with protease inhibitor (50 μL of protease inhibitor cocktail; Sigma 13786). Then the mixture was sonicated at 20 KHz for 1 min at 4°C (6 intervals of 10 sec). Cell sonicates were centrifuged at 100,000 g (30 min) [2].

The supernatant was saturated with ammonium sulfate to 30% concentration. After stirring for 30–45 min, precipitated proteins were separated by centrifugation (10,000 g for 20 min). The supernatant was more concentrated to 60%, stirred, and centrifuged as like as before [2]. Both of the pellets (30 and 60%) after dialyzing with 50 mL potassium phosphate buffer solution (10 mM; pH 7.0) on cellulose acetate membrane (MWCO 50 KDa Sigma), due to removing of salts and molecules with M.W. < 50 KDa, were dissolved in the aforementioned buffer (5 mL) and used for enzyme assay. Lipoxygenase activity against substrate linoleic acid (product: 13-HPODE) was determined by using of measuring absorbance increase at 234 nm in Tris buffer (50 mM, pH 7.2) similar to the previous literatures: [32] 0.047 absorbance increase at 234 nm per min per 100 μL in 1 mL reaction medium at 35°C equal to 10.4 nmol production of 13-HPODE per min per 100 μL (104 unit/mL). It is equal to 0.023 absorbance increase at 598 nm per min per 100 μL in 1 mL reaction medium at 30°C by DMAB-MBTH method.

### Lipoxygenase inhibitory assessment

Linoleic acid and two assay solutions (A and B) were prepared in advance.

Solution A was 50 mM DMAB in an l00 mM phosphate buffer (pH 7.0). Solution B was a mixture of l0 mM MBTH (3 mL), hemoglobin (5 mg/mL, 3 mL) in 50 mM phosphate buffer at pH 5.0 (25 ml). A linoleic acid solution was prepared by mixing 5.6 mg of linoleic acid (Sigma Aldrich, L1376) with 0.5 mL methanol and then diluted with KOH 100 mM to a final volume of 5 mL (4 mM).

In the standard assay, the sample in DMSO (12.5 μL), 15-LOX-1 (104 units/mL; 30 μL) and phosphate buffer, pH 7.0 (50 mM; 435 μL) were mixed in 48 well plate and perincubation was carried out for 10 min at 30°C. A control test was done with the same volume of ethanol. After the preincubation, linoleic acid solution (25 μL) was added to start the peroxidation reaction at 30°C, and, 10 min later, solution A (135 μL) and then solution B (65 μL) was added to start the color formation. Further, 3 min later, 100 μL of a 2% SDS solution was added to terminate the reaction. The absorbance at 598 nm was compared with the control test [15, 19]. These experiments were performed in triplicate. The data analysis was performed using GraphPad Prism 5.01.

### Michaelis-Menten enzyme kinetics

In this kinetic study, the lipoxygenase activity was assayed in Tris buffer (50 mM, pH 7.2). linoleic acid concentrations ranging from 0.1 to 1.6 mM were made using the 4 mM solution (preparation described in the last section). The enzyme activity was measured in the absence or presence of final concentrations of the inhibitor **4d** (0, 0.5, 1 and 2.5 μM).

10 μL of the enzyme solution (104 units/mL) was mixed with mixture of assay buffer (170 μL) and inhibitor (10 μL of ethanol solution of inhibitor or 10 μL of ethanol for absence of the inhibitor) incubated for 10 min at 30°C in UV-transparent 96 well plate. Subsequently, 10 μL of linoleic acid solutions (0.1–1.6 mM) was added to the mixture to start the enzyme reaction. After 10 min, the absorbance was read at 235 nm. By using of the results (reaction velocity (Abs.min^-1^) and substrate concentrations (μM), Michaelis-Menten and Lineweaver-Burk plots, K_M_ and V_max_ were derived. All experiments were performed in triplicate. The data analysis was performed using GraphPad Prism 5.01.

### Determination of DPPH bleaching

25 μM solution of DPPH in absolute ethanol was prepared. This solution was added to an equal volume of the solution of the test compounds (dissolved in ethanol) to obtain a desired concentration. Ethanol was used as control solution. After 30 min at room temperature, the absorbance was read at 517 nm and the significant decrease in absorbance in comparison with the control was recorded [22].

### General procedure for preparation of hydroxycoumarin-3-carboxylic acid ethyl esters (2), (6), (10)

Desired hydroxysalicylaldehyde (4-hydroxysalicylaldehyde and 5-hydroxysalicylaldehyde) (1.6 mmol, 0.35 g), diethyl malonate (1.8 mmol, 0.27 mL), acetic acid (0.1 mL), piperidine (0.1 mL) and ethanol (5 mL) were refluxed together. After complection the reaction (control with TLC), solvent was removed by vacuo and crud products were recrystallized from ethanol. In the case of 5-hydroxycoumarin-3-carboxylic acid ethyl ester (**2**), Piperidine (0.05 mL) was added to a cooled mixture of 2,6-dihydroxybenzaldehyde (1.4 mmol, 0.2 g.) and diethyl malonate (1.6 mmol, 0.25 mL) and ethanol (1 mL). The resulting red liquid, left at 35–40°C overnight. It was treated with dilute cold hydrochloric acid; the resulting solid crystallized from ethanol in yellowish plates [33].

#### Ethyl 5-hydroxy-2-oxo-2H-chromene-3-carboxylate (2)

Orange needle, mp: 232°C (from ethanol); Lit: [33] 229–230°C.

#### Ethyl 6-hydroxy-2-oxo-2H-chromene-3-carboxylate (6)

Yellow needle, mp: 184–186°C (from ethanol); Lit: [17] 188–189°C.

#### Ethyl 7-hydroxy-2-oxo-2H-chromene-3-carboxylate (10)

White needle, mp: 164–166°C (from ethanol); Lit: [17] 165–166°C.

### General procedure for preparation of 8-hydroxycoumarin-3-carboxylic acid methyl ester (16)

3-methoxysalicylaldehyde (0.01 mol; 1.53 g), malonic acid (0.01 mol; 1.04g), acetic acid (5 mmol; 0.3 g), aniline (0.4 mmol; 0.037 g) and benzene (5 mL) were refluxed together. After 8 hours the reaction mixture was cooled and filtered. The produced methoxy-3-carboxycoumarin (**14**) was washed with benzene and after drying used for next step without further purification [18].

A mixture of 8-methoxycoumarin-3-carboxylic acid (2 mmol, 0.44 g) (**14**) and acetic acid (3 mL) and hydrobromic acid 47% (3 mL) was refluxed for 24 hours. After concentrated in vacuo 8-hydroxycoumarin -3-carboxylic acid (**15**) as a green precipitated was obtained.

#### 8-Hydroxy-2-oxo-2H-chromene-3-carboxylic acid (15)

Dark green amorphous powder, mp: 310°C (from water).

In the last step, 8-hydroxycoumarin-3-carboxylic acid (**15**) (2 mmol, 0.41g) was refluxed in methanol (5 mL) and concentrated sulfuric acid (0.1 mL). After 5 hours the reaction mixture was cooled and precipitated was filtrated and washed with cold methanol and water. 8-hydroxycoumarin-3-carboxylic acid methyl ester was prepared as pale-yellow needle in a good purity and yield.

#### Methyl 8-hydroxy-2-oxo-2H-chromene-3-carboxylate (16)

Pale-yellow needle, 72% yield; mp: 214–216°C; ^1^H NMR (400 MHz, CDCl_3_): δ = 3.98 (s, 3H, OCH_3_), 6.04 (brs, 1H, OH), 7.18–7.31 (m, 3H, ArH), 8.61 (s, 1H, H-4 coumarin); ^13^C NMR (100 MHz, CDCl_3_): δ = 52.99, 117.33, 120.62, 120.79, 125.38, 144.69, 146.91, 148.73, 150.06, 162.98 ppm; IR (KBr): 3297, 2945, 1764, 1703, 1621 cm^−1^; MS (*m/z*) 220 (M^+^), 218, 204, 188, 159; Anal. calcd for C_10_H_6_O_5_: C 58.26; H 2.93, Found: C 58.21; H 2.90%.

### General procedure for preparation of prenyloxycoumarin-3-carboxylic acid ethyl esters (3a-d, 7a-d, 11a-d, 17a-d)

In dry dimethylformamide (5 mL) was dissolved of desired hydroxycoumarin-3-carboxylic acid ethyl ester (1.8 mmol, 0.4 g), and sodium hydride (3.6 mmol, 0.08 g) was then added to the solution with stirring and ice cooling. The reaction mixture was kept at the same condition for 30 minutes. The temperature was then elevated and prenyl bromide (1.8 mmol) was added dropwise at 50°C. After complection the reaction (control by TLC) whitin 5–10 hours, reaction mixture was poured into 10% hydrochloric acid (30 mL) added with ice, the resulting extracted with chloroform tree times (2×15 mL) and organic layer then washed with dilute solution of sodium hydroxide and was dried over anhydrous Na_2_SO_4_ and concentrated in vacuo. The residue was purified by silica gel column chromatography, eluting with EtOAc/n-hexane.

#### Ethyl 5-(allyloxy)-2-oxo-2H-chromene-3-carboxylate (3a)

Yellow amorphous powder, 70% yield; mp: 102–103°C; ^1^H NMR (300 MHz, CDCl_3_): δ = 1.31–1.36 (t, *J* = 6.9 Hz, 3H, -OCH_2_ CH_3_), 4.31–4.37 (q, *J* = 7.2 Hz, 2H, -OCH_2_ CH_3_), 4.62 (d, *J* = 6.9 Hz, 2H, -OCH_2_ (Allyl)), 5.28–5.32 (dd, *J* = 1.2 and 10.2 Hz, 1H, = CH_2_ (Allyl)), 5.36–5.42 (dd, *J* = 1.2 and 17.4 Hz, 1H, = CH_2_ (Allyl)), 5.95–6.08 (m, 1H, = CH (Allyl)), 6.64 (d, *J* = 8.4 Hz, 1H, H-8 (coumarin)), 6.83 (d, *J* = 5.1 Hz, 1H, H-6 (coumarin)), 7.24–7.48 (t, *J* = 5.1 Hz, 1H, H-7 (coumarin)), 8.89 (s, 1H, H-4 (coumarin)); ^13^C NMR (75 MHz, CDCl_3_): δ = 14.29, 61.83, 69.77, 106.43, 108.94, 109.15, 116.06, 118.76, 131.96, 135.17, 144.12, 156.20, 156.32, 156.91, 163.42 ppm; IR (KBr): 3084, 2982, 1765, 1703, 1605 cm^−1^; MS (*m/z*) 274 (M^+^), 271, 244, 227, 200, 187; Anal. calcd for C_15_H_14_O_5_: C 65.79, H 5.15, Found: C 65.59, H 5.20%.

#### Ethyl 5-(3-methylbut-2-enyloxy)-2-oxo-2H-chromene-3-carboxylate (3b)

Yellow amorphous powder, 65% yield; mp: 93–94°C; ^1^H NMR (300 MHz, CDCl_3_): δ = 1.41–1.46 (t, *J* = 6.9 Hz, 3H, -OCH_2_ CH_3_), 1.80 (s, 3H, CH_3_ (prenyl)), 1.85 (s, 3H, CH_3_ (prenyl)), 4.41–4.48 (q, *J* = 7.2 Hz, 2H, -OCH_2_ CH_3_), 4.69 (d, *J* = 6.6 Hz, 2H, -OCH_2_ (prenyl)), 5.51–5.55 (t, *J* = 6.3 Hz, 1H, = CH (prenyl)), 6.75 (d, *J* = 8.4 Hz, 1H, H-8 (coumarin)), 6.92 (d, *J* = 8.4 Hz, 1H, H-6 (coumarin)), 7.52–7.57 (t, *J* = 8.4 Hz, 1H, H-7 (coumarin)), 8.98 (s, 1H, H-4 (coumarin)); ^13^C NMR (75 MHz, CDCl_3_): δ = 14.31, 18.36, 25.84, 61.84, 65.97, 106.35, 108.61, 109.23, 115.93, 118.54, 135.18, 139.25, 144.45, 156.26, 156.79, 163.64 ppm; IR (KBr): 3064, 2979, 1766, 1708, 1608 cm^−1^; MS (*m/z*) 302 (M^+^), 300, 255, 232, 188, 161; Anal. calcd for C_17_H_18_O_5_: C 67.54, H 6.00, Found: C 67.50, H 5.91%

#### Ethyl 5-((E)-3,7-dimethylocta-2,6-dienyloxy)-2-oxo-2H-chromene-3-carboxylate (3c)

Yellow liquid, 55% yield; ^1^H NMR (300 MHz, CDCl_3_): δ = 1.41–1.46 (t, *J* = 6.9 Hz, 3H, -OCH_2_ CH_3_), 1.46 (s, 3H, CH_3_ (geranyl)), 1.71 (s, 3H, CH_3_ (geranyl)), 1.80 (s, 3H CH_3_ (geranyl)), 2.11–2.18 (m, 4H, 2-CH_2_ (geranyl)), 4.41–4.48 (q, *J* = 7.2 Hz, 2H, -OCH_2_ CH_3_), 4.72 (d, *J* = 6 Hz, 2H, -OCH_2_ (geranyl)), 5.08–5.12 (m, 1H, = CH (geranyl)), 5.52–5.55 (t, *J* = 7.2 Hz, 1H, = CH (geranyl)), 6.74 (d, *J* = 8.4 Hz, 1H, H-8 (coumarin)), 6.93 (d, *J* = 8.4 Hz, 1H, H-6 (coumarin)), 7.52–7.58 (t, *J* = 8.4 Hz, 1H, H-7 (coumarin)), 8.94 (s, 1H, H-4 (coumarin)); ^13^C NMR (75 MHz, CDCl_3_): δ = 14.28, 16.78, 17.72, 25.68, 26.23, 39.50, 61.79, 66.07, 106.42, 108.61, 109.27, 115.99, 118.38, 123.57, 132.01, 135.11, 142.33, 144.34, 156.27, 156.80, 163.59 ppm; IR (Nujol): 3064, 2977, 1766, 1711, 1691, 1620 cm^−1^; MS (*m/z*) 370 (M^+^), 368, 232, 201, 187, 161; Anal. calcd for C_22_H_26_O_5_: C 71.33, H 7.07, Found: C 71.30, H 7.00%.

#### Ethyl 5-((2E,6E)-3,7,11-trimethyldodeca-2,6,10-trienyloxy)-2-oxo-2H-chromene-3-carboxylate (3d)

Yellow liquid, 55% yield; ^1^H NMR (300 MHz, CDCl_3_): δ = 1.41–1.46 (t, *J* = 6.9 Hz, 3H, -OCH_2_ CH_3_), 1.61 (s, 3H, CH_3_ (farnesyl)), 1.63 (s, 3H, CH_3_ (farnesyl)), 1.71 (s, 3H, CH_3_ (farnesyl)), 1.80 (s, 3H, CH_3_ (farnesyl)), 1.97–2.19 (m, 8H, 4-CH_2_ (farnesyl)), 4.40–4.47 (q, *J* = 7.2 Hz, 2H, -OCH_2_ CH_3_)), 4.72 (d, *J* = 6.6 Hz, 2H, -OCH_2_ (farnesyl)), 5.07–5.15 (m, 2H, = CH (farnesyl)), 5.50–5.54 (t, *J* = 6.6 Hz, 1H, = CH (farnesyl)), 6.74 (d, *J* = 8.1 Hz, 1H, H-8 (coumarin)), 6.92 (d, *J* = 8.4 Hz, 1H, H-6 (coumarin)), 7.52–7.54 (t, *J* = 8.4 Hz, 1H, H-7 (coumarin)), 8.94 (s, 1H, H-4 (coumarin)); ^13^C NMR (75 MHz, CDCl_3_): δ = 14.28, 16.05, 16.82, 17.69, 25.70, 26.17, 26.70, 39.52, 39.69, 61.80, 66.06, 106.39, 108.61, 109.25, 115.95, 118.37, 123.47, 124.25, 131.37, 135.13, 135.65, 142.36, 144.38, 156.26, 156.79, 157.00, 163.58 ppm; IR (Nojul): 3056, 2966, 1767, 1710, 1670, 1608 cm^−1^; MS (*m/z*) 439 (M^+^), 232, 204, 187, 161; Anal. calcd for C_27_H_34_O_5_: C 73.94, H 7.81, Found: C 74.00, H 7.83%

#### Ethyl 6-(allyloxy)-2-oxo-2H-chromene-3-carboxylate (7a)

Yellow amorphous powder, 78% yield; mp: 100–101°C; ^1^H NMR (300 MHz, CDCl_3_): δ = 1.41–1.46 (t, *J* = 6.9 Hz, 3H, -OCH_2_ CH_3_), 4.41–4.48 (q, *J* = 7.2 Hz, 2H, -OCH_2_ CH_3_), 4.6–4.63 (m, 2H, -OCH_2_ (Allyl)), 5.34–5.39 (dd, *J* = 1.2 and 10.5 Hz, 1H, = CH_2_ (Allyl)), 5.43–5.49 (dd, *J* = 1.2 and 17.4 Hz, 1H, = CH_2_ (Allyl)), 6.04–6.11 (m, 1H, = CH (Allyl)), 7.04 (d, *J* = 2.7 Hz, 1H, H-5 (coumarin)), 7.25–7.3 (dd, *J* = 2.7 and 9.3 Hz, 1H, H-7 (coumarin)), 7.32 (d, *J* = 9 Hz, 1H, H-8 (coumarin)), 8.49 (s, 1H, H-4 (coumarin)); ^13^C NMR (75 MHz, CDCl_3_): δ = 14.25, 62, 69.55, 111.92, 117.90, 118.16, 118.64, 123.18, 132.46, 148.83, 155.23, 156.91, 163.21 ppm; IR (KBr): 3040, 2937, 1751, 1699 cm^−1^; MS (*m/z*) 274 (M^+^), 271, 231, 203, 174, 160; Anal. calcd for C_15_H_14_O_5_: C 65.69, H 5.15, Found: C, 65.61; H, 5.10%.

#### Ethyl 6-(3-methylbut-2-enyloxy)-2-oxo-2H-chromene-3-carboxylate (7b)

Yellow amorphous powder, 75% yield; mp: 97–98°C; ^1^H NMR (300 MHz, CDCl_3_): δ = 1.41–1.46 (t, *J* = 6.9 Hz, 3H, -OCH_2_ CH_3_), 1.79 (s, 3H, CH_3_ (prenyl)), 1.84 (s, 3H, CH_3_ (prenyl)), 4.41–4.48 (q, *J* = 7.2 Hz, 2H, -OCH_2_ CH_3_), 4.57 (d, *J* = 6.6 Hz, 2H, -OCH_2_ (prenyl)), 5.47–5.57 (m, 1H, = CH (prenyl)),7.04 (d, *J* = 2.7 Hz, 1H, H-5 (coumarin)), 7.23–7.27 (dd, *J* = 3 and 9 Hz, 1H, H-7 (coumarin)), 7.31 (d, *J* = 9.3 Hz, 1H, H-8 (coumarin)), 8.49 (s, 1H, H-4 (coumarin)); ^13^C NMR (75 MHz, CDCl_3_): δ = 14.25, 18.28, 25.84, 61.99, 65.59, 111.69, 117.85, 118.14, 118.53, 118.88, 123.27, 139.14, 148.48, 149.72, 155.52, 157.00, 163.28 ppm; IR (KBr): 3047, 2933, 1750, 1705, 1621 cm^−1^; MS (*m/z*) 302 (M^+^), 299, 232, 188, 161; Anal. calcd for C_17_H_18_O_5_: C 67.54, H 6.00, Found: C 67.49, H 5.92%.

#### Ethyl 6-((E)-3,7-dimethylocta-2,6-dienyloxy)-2-oxo-2H-chromene-3-carboxylate (7c)

Yellow amorphous powder, 64% yield; mp: 70–71°C; ^1^H NMR (300 MHz, CDCl_3_): δ = 1.41–1.46 (t, *J* = 6.9 Hz, 3H, -OCH_2_ CH_3_), 1.63 (s, 3H, CH_3_ (geranyl)), 1.70 (s, 3H, CH_3_ (geranyl)), 1.78 (s, 3H, CH_3_ (geranyl)), 2.14–2.17 (m, 4H, 2-CH_2_ (geranyl)), 4.41–4.48 (q, *J* = 7.2 Hz, 2H, -OCH_2_ CH_3_), 4.60 (d, *J* = 6.6 Hz, 2H, -OCH_2_ (geranyl)), 5.08–5.15 (m, 1H, = CH (geranyl)), 5.49–5.55 (t, *J* = 6.6 Hz, 1H, = CH (geranyl)), 7.04 (d, *J* = 3 Hz, 1H, H-5 (coumarin)), 7.24–7.27 (dd, *J* = 2.7 and 9.3 Hz, 1H, H-7 (coumarin)), 7.33 (d, *J* = 9.3 Hz, 1H, H-8 (coumarin)), 8.49 (s, 1H, H-4 (coumarin)); ^13^C NMR (75 MHz, CDCl_3_): δ = 14.25, 16.67, 17.12, 25.68, 26.25, 39.52, 61.99, 65.68, 111.77, 117.82, 118.14, 118.52, 118.69, 123.28, 123.68, 123.62, 131.98, 142.19, 148.47, 149.71, 155.54, 156.99, 163.27 ppm; IR (KBr): 3056, 2974, 1764, 1743, 1699, 1618 cm^−1^; MS (*m/z*) 370 (M^+^), 367, 322, 233, 188, 161; Anal. calcd for C_22_H_26_O_5_: C 71.33, H 7.07, Found: C 71.25, H 7.01%.

#### Ethyl 6-((2E,6E)-3,7,11-trimethyldodeca-2,6,10-trienyloxy)-2-oxo-2H-chromene-3-carboxylate (7d)

Yellow amorphous powder, 60% yield; mp: 53–55°C; ^1^H NMR (300 MHz, CDCl_3_): δ = 1.39–1.43 (t, *J* = 7.2 Hz, 3H, -OCH_2_ CH_3_), 1.60 (s, 6H, 2CH_3_ (farnesyl)), 1.67 (s, 3H, CH_3_ (farnesyl)), 1.76 (s,3H, CH_3_ (farnesyl)), 1.94–2.16 (m, 8H, 4-CH_2_ (farnesyl)), 4.38–4.45 (q, *J* = 7.2 Hz, 2H, -OCH_2_ CH_3_), 4.57 (d, *J* = 6.6 Hz, 2H, -OCH_2_ (farnesyl)), 5.08–5.13 (m, 2H, = CH (farnesyl)), 5.46–5.52 (t, *J* = 6.3 Hz, 1H, = CH (farnesyl)), 7.01 (d, *J* = 2.7 Hz, 1H, H-5 (coumarin)), 7.21–7.24 (dd, *J* = 2.7 and 9.3 Hz, 1H, H-7 (coumarin)), 7.28 (d, *J* = 9.3 Hz, 1H, H-8 (coumarin)), 8.46 (s, 1H, H-4 (coumarin)); ^13^C NMR (75 MHz, CDCl_3_): δ = 14.25, 16.05, 16.77, 17.69, 21.16, 25.69, 26.71, 39.53, 39.69, 61.97, 65.67, 111.75, 117.81, 118.15, 118.52, 118.71, 123.25, 123.50, 124.26, 131.37, 135.61, 142.20, 148.45, 149.71, 155.55, 156.97, 163.26 ppm; IR (KBr): 3043, 2968, 1744, 1699, 1619 cm^−1^; MS (*m/z*) 438 (M^+^), 233, 188; Anal. calcd for C_27_H_34_O_5_: C 73.94, H 7.81, Found: C 74.00; H 7.86%.

#### Ethyl 7-(allyloxy)-2-oxo-2H-chromene-3-carboxylate (11a)

White amorphous powder, 77% yield; mp: 99–100°C; ^1^H NMR (300 MHz, CDCl_3_): δ = 1.41–1.45 (t, *J* = 6.9 Hz, 3H, -OCH_2_ CH_3_), 4.39–4.46 (q, *J* = 7.2 Hz, 2H, -OCH_2_ CH_3_), 4.66 (d, *J* = 5.4 Hz, 2H, -OCH_2_ (Allyl)), 5.37–5.41 (dd, *J* = 1.2 and 10.5 Hz, 1H, = CH_2_ (Allyl)), 5.44–5.51 (dd, *J* = 1.5 and 17.1 Hz, 1H, = CH_2_ (Allyl)), 6.02–6.08 (m, 1H, = CH (Allyl)), 6.85 (d, *J* = 2.1 Hz, 1H, H-8 (coumarin)), 6.92–6.95 (dd, *J* = 2.4 and 8.7 Hz,1H, H-6 (coumarin)), 7.52 (d, *J* = 8.7 Hz, 1H, H-5 (coumarin)), 8.53 (s, 1H, H-4 (coumarin)); ^13^C NMR (75 MHz, CDCl_3_): δ = 14.29, 61.73, 69.52, 101.24, 111.74, 114.14, 118.92, 130.70, 131.73, 148.92, 157.13, 157.50, 163.47, 164.07 ppm; IR (KBr): 3056, 2976, 1755, 1703, 1617 cm^−1^; MS (*m/z*) 274 (M^+^), 271, 227, 200, 187, 176; Anal. calcd for C_15_H_14_O_5_: C 65.69, H 5.15, Found: C 65.59, H 5.09%.

#### Ethyl 7-(3-methylbut-2-enyloxy)-2-oxo-2H-chromene-3-carboxylate (11b)

White amorphous powder, 70% yield; mp: 93–94°C; ^1^H NMR (300 MHz, CDCl_3_): δ = 1.41–1.45 (t, *J* = 7.2 Hz, 3H, -OCH_2_ CH_3_), 1.81 (s, 3H, CH_3_ (prenyl)), 1.84 (s, 3H, CH_3_ (prenyl)), 4.39–4.46 (q, *J* = 7.2 Hz, 2H, -OCH_2_ CH_3_)), 4.63 (d, *J* = 6.9 Hz, 2H, -OCH_2_ (prenyl)), 5.94 (m,1H, = CH (prenyl)), 6.84 (d, *J* = 2.1 Hz, 1H, H-8 (coumarin)), 6.89–6.93 (dd, *J* = 2.4 and 8.7 Hz, 1H, H-6 (coumarin)), 7.51 (d, *J* = 8.7 Hz, 1H, H-5 (coumarin)), 8.53 (s, 1H, H-4 (coumarin)); ^13^C NMR (75 MHz, CDCl_3_): δ = 14.30, 18.33, 25.82, 61.69, 65.74, 101.05, 111.53, 113.97, 114.27, 118.26, 130.64, 139.75, 149.01, 157.24, 157.57, 163.52, 164.50 ppm; IR (KBr): 3031, 2960, 1761, 1702, 1621 cm^−1^; MS (*m/z*) 302 (M^+^), 300, 255, 232, 205, 188, 161; Anal. calcd for C_17_H_18_O_5_ C: C 67.54, H 6.00, Found: C, 67.55; H, 6.02%.

#### Ethyl 7-((E)-3,7-dimethylocta-2,6-dienyloxy)-2-oxo-2H-chromene-3-carboxylate (11c)

White amorphous powder, 65% yield; mp: 45–46°C; ^1^H NMR (300 MHz, CDCl_3_): δ = 1.41–1.45 (t, *J* = 7.2 Hz, 3H, -OCH_2_ CH_3_), 1.63 (s, 3H, CH_3_ (geranyl)), 1.69 (s, 3H, CH_3_ (geranyl)), 1.79 (s, 3H, CH_3_ (geranyl)), 2.14–2.20 (m, 4H, 2-CH_2_ (geranyl)), 4.39–4.46 (q, *J* = 7.2 Hz, 2H, -OCH_2_ CH_3_)), 4.66 (d, *J* = 6.6 Hz, 2H, -OCH_2_ (geranyl)), 5.11–5.16 (m, 1H, = CH (geranyl)), 5.46–5.51 (m, 1H, = CH (geranyl)), 6.84 (d, *J* = 2.1 Hz, 1H, H-8 (coumarin)), 6.89–6.93 (dd, *J* = 2.4 and 8.7 Hz, 1H, H-6 (coumarin)), 7.50 (d, *J* = 8.7 Hz, 1H, H-5 (coumarin)), 8.52 (s, 1H, H-4 (coumarin)); ^13^C NMR (75 MHz, CDCl_3_): δ = 14.29, 16.80, 17.70, 25.65, 26.22, 39.50, 61.68, 65.80, 101.09, 111.53, 113.99, 114.26, 118.04, 123.55, 130.61, 132.02, 142.83, 148.98, 157.21, 157.57, 163.53, 164.51 ppm; IR (KBr): 3056, 2974, 1746, 1743, 1699, 1618 cm^−1^; MS (*m/z*) 370 (M^+^), 367, 233, 188, 161; Anal. calcd for C_22_H_26_O_5_: C 71.33, H 7.07, Found: C 71.35; H 7.09%.

#### Ethyl 7-((2E,6E)-3,7,11-trimethyldodeca-2,6,10-trienyloxy)-2-oxo-2H-chromene-3-carboxylate (11d)

White amorphous powder, 65% yield; mp: 40–41°C; ^1^H NMR (300 MHz, CDCl_3_): δ = 1.40–1.45 (t, *J* = 14.1 Hz, 3H, -OCH_2_ CH_3_), 1.61 (s, 6H, 2CH_3_ (farnesyl)), 1.69 (s, 3H, CH_3_ (farnesyl)), 1.79 (s, 3H, CH_3_ (farnesyl)), 1.97–2.17 (m, 8H, 4-CH_2_ (farnesyl)), 4.38–4.45 (q, *J* = 7.2 Hz, 2H, -OCH_2_ CH_3_), 4.65 (d, *J* = 6.6 Hz, 2H, -OCH_2_ (farnesyl)), 5.07–5.11 (m, 2H, = CH (farnesyl)), 5.46–5.50 (t, *J* = 5.7 Hz, 1H, = CH (farnesyl)), 6.83 (d, *J* = 2.1 Hz, 1H, H-8 (coumarin)), 6.89–6.93 (dd, *J* = 2.4 and 11.1 Hz, 1H, H-6 (coumarin)), 7.50 (d, *J* = 8.7 Hz, 1H, H-5 (coumarin)), 8.53 (s, 1H, H-4 (coumarin)); ^13^C NMR (75 MHz, CDCl_3_): δ = 14.31, 16.07, 16.83, 17.70, 25.71, 26.10, 26.69, 39.51, 39.67, 61.70, 65.78, 101,06, 111.51, 113.89, 114.26, 118.02, 123.41, 124.27, 130.65, 131.37, 135.66, 142.88, 149.05, 157.56, 163.52, 164.51 ppm; IR (KBr): 3043, 2968, 1744, 1699, 1619 cm^−1^; MS (*m/z*) 438 (M^+^), 433, 233, 204, 188, 161; Anal. calcd for C_27_H_34_O_5_: C 73.94, H 7.81, Found: C 73.90, H 7.88%.

#### Methyl 8-(allyloxy)-2-oxo-2H-chromene-3-carboxylate (17a)

Pale-brown amorphous powder, 74% yield; mp: 97–98°C; ^1^H NMR (400 MHz, CDCl_3_): δ = 3.97 (s, 3H, OCH_3_), 4.73 (d, *J* = 5.2 Hz, 2H, -OCH_2_ (Allyl)), 5.35 (d, *J*_*cis*_ = 10.8 Hz, 1H, = CH_2_ (Allyl)), 5.47 (d, *J*_*trans*_ = 17.2 Hz, 1H, = CH_2_ (Allyl)), 6.05–6.15 (m, 1H, = CH (Allyl)), 7.19–7.29 (m, 3H, H-7,H-6, H-5 (coumarin)), 8.56 (s, 1H, H-4 (coumarin)); ^13^C NMR (100 MHz, CDCl_3_): δ = 52.96, 70.25, 117.87, 118.11, 118.63, 118.67, 121.03, 124.67, 132.36, 145.27, 146, 149.42, 156.24, 163.83 ppm; IR (KBr): 3051, 2949, 1755, 1700, 1615 cm^−1^; MS (*m/z*) 260 (M^+^), 257, 227, 217, 190, 162; Anal. calcd for C_14_H_12_O_5_: C 64.61, H 4.65, Found: C 64.60, H 4.63%.

#### Methyl 8-(3-methylbut-2-enyloxy)-2-oxo-2H-chromene-3-carboxylate (17b)

Pale-brown amorphous powder, 75% yield; mp: 78–80°C; ^1^H NMR (300 MHz, CDCl_3_): δ = 1.78 (s, 3H, CH_3_ (prenyl)), 1.81 (s, 3H, CH_3_ (prenyl)), 3.97 (s, 3H, OCH_3_), 4.70 (d, *J* = 6.6 Hz, 2H, -OCH_2_ (prenyl)), 5.51–5.56 (m, 1H, = CH (prenyl)), 7.17–7.29 (m, 3H, H-7,H-6, H-5 (coumarin)), 8.56 (s, 1H, H-4 (coumarin)); ^13^C NMR (75 MHz, CDCl_3_): δ = 18.31, 25.80, 52.91, 66.35, 117.76, 118.07, 118.58, 119.03, 120.68, 124.65, 138.84, 145.41, 146.35, 149.44, 156.29, 163.89 ppm; IR (KBr): 3047, 2953, 1762, 1696, 1617 cm^−1^; MS (*m/z*) 187 (M^+^), 255, 218, 187, 159; Anal. calcd for C_16_H_16_O_5_: C 66.66, H, 5.59, Found: C 67.60, H 5.51%.

#### Methyl 8-((E)-3,7-dimethylocta-2,6-dienyloxy)-2-oxo-2H-chromene-3-carboxylate (17c)

Pale-brown amorphous powder, 64% yield; mp: 50–52°C; ^1^H NMR (400 MHz, CDCl_3_): δ = 1.72 (s, 3H, CH_3_ (geranyl)), 1.77 (s, 3H, CH_3_ (geranyl)), 1.82 (s, 3H, CH_3_ (geranyl)) 2.06–2.15 (m, 4H, 2-CH_2_ (geranyl)), 3.97 (s, 3H, -OCH_3_), 4.71 (d, *J* = 5.6 Hz, 2H, -OCH_2_ (geranyl)), 5.07–5.09 (t, *J* = 6.4 Hz, 1H, = CH (geranyl)), 5.51–5.54 (t, *J* = 6.4 Hz, 1H, = CH (geranyl)), 7.18–7.29 (m, 3H, H-7,H-6, H-5 (coumarin)), 8.57 (s, 1H, H-4 (coumarin)); ^13^C NMR (100 MHz, CDCl_3_): δ = 16.79, 17.75, 25.71, 26.21, 39.51, 52.95, 66.40, 117.78, 118.01, 118.57, 118.85, 120.68, 123.67, 124.67, 131.95, 141.90, 145.53, 146.32, 149.53, 156.33, 163.90; IR (KBr): 3051, 2970, 2916, 1744, 1701, 1611 cm^−1^; MS (*m/z*) 355 (M^+^), 353, 322, 218, 187, 159; Anal. calcd for C_21_H_24_O_5_: C 70.77, H 6.79, Found: C, 70.70; H, 6.71%.

#### Methyl 8-((2E,6E)-3,7,11-trimethyldodeca-2,6,10-trienyloxy)-2-oxo-2H-chromene-3-carboxylate (17d)

Pale-brown amorphous powder, 61% yield; mp: 41–42°C; ^1^H NMR (400 MHz, CDCl_3_): δ = 1.62 (s, 6H, 2-CH_3_ (farnesyl)), 1.70 (s, 3H, CH_3_ (farnesyl)), 1.78 (s,3H, CH_3_ (farnesyl)), 1.96–2.20 (m, 8H, 4-CH_2_ (farnesyl)), 3.98 (s,3H, -OCH_3_), 4.75 (d, *J* = 6.4 Hz, 2H, -OCH_2_ (farnesyl)), 5.09–5.11 (m, 2H, = CH (farnesyl)), 5.52–5.55 (t, *J* = 6.4 Hz, 1H, = CH (farnesyl)), 7.18–7.29 (m, 3H, H-7,H-6, H-5 (coumarin)), 8.57 (s, 1H, H-4 (coumarin)); ^13^C NMR (100 MHz, CDCl_3_): δ = 16.07, 16.83, 17.72, 25.73, 26.17, 26.71, 39.53, 39.70, 52.96, 66.39, 117.77, 118.02, 118.58, 118.82, 120.68, 123.56, 124.31, 124.66, 131.36, 135.57, 141.98, 145.40, 146.32, 149.53, 156.33, 163.92; IR (KBr): 3055, 2965, 1760, 1743, 1696, 1610 cm^−1^; MS (*m/z*) 422 (M^+^), 390, 217, 202, 187, 160; Anal. calcd for C_26_H_32_O_5_: C 73.56, H 7.60, Found: C 73.49, H 7.55%.

### General procedure for preparation of prenyloxycoumarin-3-carboxylic acids (4a-d, 8a-d, 12a-d, 18a-d)

To solution of methanol (3 mL) and tetrahydrofuran (3 mL) and sodium hydroxide 2N (3 mL), prenyloxycoumarin-3-carboxylic acid ethyl esters (1 mmol) were added and then heated at 50°C for 2–3 hours, except 5-prenyloxycoumarin-3-carboxylic acid ethyl ester that reaction was carried on at room temperature for 8–9 hours.

After acidified the reaction mixture with concentrated hydrochloric acid, aqueous layer extracted with ethylacetate tree times (3×10) and organic layer was dried over anhydrous Na_2_SO_4_ and concentrated in vacuo. The residue was purified by silica gel column chromatography, eluting with chloroform/methanol or recrystallization from (HOAc/H_2_O).

#### 5-(allyloxy)-2-oxo-2H-chromene-3-carboxylic acid (4a)

Yellow needle, 66% yield; mp: 115–117°C; ^1^H NMR (300 MHz, CDCl_3_): δ = 4.75 (d, *J* = 5.4 Hz, 2H, -OCH_2_ (Allyl)), 5.41–5.45 (d, *J* = 10.5 Hz, 1H, = CH_2_ (Allyl)), 5.47–5.52 (d, *J* = 17.1 Hz, 1H, = CH_2_ (Allyl)), 6.05–6.18 (m, 1H, = CH (Allyl)), 6.86 (d, *J* = 8.4 Hz, 1H, H-8 (coumarin)), 7.06 (d, *J* = 8.4 Hz, 1H, H-6 (coumarin)), 7.66–7.72 (t, *J* = 8.4 Hz, 1H, H-7 (coumarin)), 9.36 (s,1H, H-4 (coumarin)), 12.28 (s, 1H, COOH); ^13^C NMR (75 MHz, CDCl_3_): δ = 70.16, 107.66, 109.04, 110.07, 112.58, 119.41, 131.48, 136.72, 147.17, 155.50, 157.10, 162.75, 164.24 ppm; IR (KBr): 3064, 2925, 1755, 1673, 1602 cm^−1^; Anal. calcd for C_13_H_10_O_5_: C 63.42, H 4.09, Found: C 63.40, H 4.02%.

#### 5-(3-methylbut-2-enyloxy)-2-oxo-2H-chromene-3-carboxylic acid (4b)

Yellow needle, 60% yield; mp: 85–86°C; ^1^H NMR (300 MHz, CDCl_3_): δ = 1.80 (s, 3H, CH_3_ (prenyl)), 1.85 (s, 3H, CH_3_ (prenyl)), 4.69 (d, *J* = 6.6 Hz, 2H, -OCH_2_ (prenyl)), 5.51–5.55 (t, *J* = 6.3 Hz, 1H, = CH (prenyl)), 6.75 (d, *J* = 8.4 Hz, 1H, H-8 (coumarin)), 6.92 (d, *J* = 8.4 Hz, 1H, H-6 (coumarin)), 7.52–7.57 (t, *J* = 8.4 Hz, 1H, H-7 (coumarin)), 8.98 (s, 1H, H-4 (coumarin)), 12.28 (s, 1H, COOH); ^13^C NMR (75 MHz, CDCl_3_): δ = 18.36, 25.84, 65.97, 106.35, 108.61, 109.23, 115.93, 118.54, 135.18, 139.25, 144.45, 156.26, 156.79, 163.64 ppm; IR (KBr): 3052, 2970, 1737, 1691, 1620 cm^−1^; MS (*m/z*) 274 (M^+^), 231, 205, 187, 162; Anal. calcd for C_15_H_14_O_5_: C 65.69, H, 5.15, Found: C, 65.70; H, 5.17%.

#### 5-((E)-3,7-dimethylocta-2,6-dienyloxy)-2-oxo-2H-chromene-3-carboxylic acid (4c)

Yellow amorphous solid, 57% yield; mp: 49–50°C; ^1^H NMR (300 MHz, CDCl_3_): δ = 1.64 (s, 3H, CH_3_ (geranyl)), 1.70 (s, 3H, CH_3_ (geranyl)), 1.79 (s, 3H CH_3_ (geranyl)), 2.15–2.20 (m, 2-CH_2_ (geranyl)), 4.73 (d, *J* = 6.3 Hz, 2H, -OCH_2_ (geranyl)), 5.08–5.11 (m, 1H, = CH (geranyl)), 5.52–5.55 (t, *J* = 6 Hz, 1H, = CH (geranyl)), 6.85 (d, *J* = 8.1 Hz, 1H, H-8 (coumarin)), 7.03 (d, *J* = 8.1 Hz, 1H, H-6 (coumarin)), 7.65–7.70 (t, *J* = 8.1 Hz, 1H, H-7 (coumarin)), 9.36 (s, 1H, H-4 (coumarin)), 13.30 (s, 1H, COOH); ^13^C NMR (75 MHz, CDCl_3_): δ = 16.81, 17.73, 25.42, 25.72, 26.50, 39.50, 66.29, 107.55, 108.62, 110.12, 117.81, 123.54, 132.09, 136.79, 143.10, 147.52, 155.50, 157.57, 163.02, 164.37 ppm; IR (KBr): 3060, 2966, 1759, 1679, 1606 cm^−1^; MS (*m/z*) 342 (M^+^), 339, 205, 188, 161; Anal. calcd for C_20_H_22_O_5_: C 70.16, H 6.48, Found: C 70.12, H 6.41%.

#### 5-((2E,6E)-3,7,11-trimethyldodeca-2,6,10-trienyloxy)-2-oxo-2H-chromene-3-carboxylic acid (4d)

Yellow amorphous solid, 50% yield; mp: 39–40°C; ^1^H NMR (300 MHz, CDCl_3_): δ = 1.52 (s, 3H, CH_3_ (farnesyl)), 1.54 (s, 3H, CH_3_ (farnesyl)), 1.60 (s, 3H CH_3_ (farnesyl)), 1.70 (s, 3H, CH_3_ (farnesyl)), 1.89–2.08 (m, 8H, 4-CH_2_ (farnesyl)), 4.64 (d, *J* = 6.6 Hz, 2H, -OCH_2_ (farnesyl)), 4.98–5.03 (m, 2H = CH (farnesyl)), 5.41–5.45 (t, *J* = 6.3 Hz, 1H, = CH (farnesyl)), 6.76 (d, *J* = 8.4 Hz, 1H, H-8 (coumarin)), 6.93 (d, *J* = 8.4 Hz, 1H, H-6 (coumarin)), 7.55–7.61 (t, *J* = 8.4 Hz, 1H, H-7 (coumarin)), 9.26 (s, 1H, H-4 (coumarin)), 12.33 (bs, 1H, COOH); ^13^C NMR (75 MHz, CDCl_3_): δ = 16.07, 16.83, 17.68, 25.68, 26.11, 26.71, 39.50, 39.68, 66.32, 107.57, 108.64, 110.14, 112.35, 117.81, 123.41, 124.27, 131.36, 135.71, 136.72, 143.16, 147.46, 155.54, 157.60, 162.89, 164.35 ppm; IR (KBr): 3060, 2966, 1759, 1679, 1606 cm^−1^; MS (*m/z*) 410 (M^+^), 409, 204, 187, 161; Anal. calcd for C_25_H_30_O_5_: C 73.15, H 7.37, Found: C, 73.10; H, 7.31%.

#### 6-(allyloxy)-2-oxo-2H-chromene-3-carboxylic acid (8a)

Yellow needle, 75% yield; mp: 167–168°C; ^1^H NMR (300 MHz, CDCl_3_): δ = 4.64–4.66 (m, 2H, -OCH_2_ (Allyl)), 5.4–5.41 (dd, *J* = 1.2 and 10.5 Hz, 1H, = CH_2_ (Allyl)), 5.44–5.52 (dq, *J* = 1.2 and 15.9 Hz, 1H, = CH_2_ (Allyl)), 6.02–6.14 (m, 1H, = CH (Allyl)), 7.16 (d, *J* = 2.7 Hz, 1H, H-5 (coumarin)), 7.38–7.42 (dd, *J* = 2.7 and 9 Hz, 1H, H-7 (coumarin)), 7.45 (d, *J* = 9.3 Hz, 1H, H-8 (coumarin)), 8.92 (s, 1H, H-4 (coumarin)), 12.40 (bs, 1H, COOH); ^13^C NMR (75 MHz, CDCl_3_): δ = 69.65, 112.28, 114.98, 118.33, 118.68, 118.88, 124.93, 132.09, 149.22, 151.24, 156.13, 162.54, 164.20 ppm; IR (KBr): 3047, 2933, 1748, 1684, 1621 cm^−1^; MS (*m/z*) 246 (M^+^), 243, 203, 176, 160; Anal. calcd for C_13_H_10_O_5_: C 63.42, H 4.09, Found: C 63.35, H 4.00%.

#### 6-(3-methylbut-2-enyloxy)-2-oxo-2H-chromene-3-carboxylic acid (8b)

Yellow needle, 73% yield; mp: 158–159°C; ^1^H NMR (300 MHz, CDCl_3_): δ = 1.81 (s, 3H, CH_3_ (prenyl)), 1.85 (s, 3H, CH_3_ (prenyl)), 4.62 (d, *J* = 6.9 Hz, 2H, -OCH_2_ (prenyl)), 5.48–5.53 (m, 1H, = CH (prenyl)), 7.14 (d, *J* = 2.7 Hz, 1H, H-5 (coumarin)), 7.36–7.40 (dd, *J* = 2.7 and 9 Hz, 1H, H-7 (coumarin)), 7.44 (d, *J* = 9.3 Hz, 1H, H-8 (coumarin)), 8.91 (s, 1H, H-4 (coumarin)), 12.41 (s, 1H, COOH); ^13^C NMR (75 MHz, CDCl_3_): δ = 18.32, 25.82, 65.76, 112.04, 114.88, 118.25, 118.56, 118.87, 125.04, 139.49, 149.12, 151.29, 156.48, 162.59, 164.25 ppm; IR (KBr): 3056, 2921, 1756, 1684 cm^−1^; MS (*m/z*) 274 (M^+^), 271, 228, 204, 187, 161; Anal. calcd for C_15_H_14_O_5_: C 65.69, H 5.15, Found: C, 65.65; H, 5.09%.

#### Ethyl 6-((E)-3,7-dimethylocta-2,6-dienyloxy)-2-oxo-2H-chromene-3-carboxylic acid (8c)

Yellow needle, 70% yield; mp: 130–131°C; ^1^H NMR (300 MHz, CDCl_3_): δ = 1.61 (s, 3H, CH_3_ (geranyl)), 1.67 (s, 3H, CH_3_ (geranyl)), 1.77 (s, 3H, CH_3_ (geranyl)), 2.08–2.14 (m, 4H, 2-CH_2_ (geranyl)), 4.62 (d, *J* = 6.6 Hz, 2H, -OCH_2_ (geranyl)), 5.05–5.10 (m, 1H, = CH (geranyl)), 5.45–5.49 (t, *J* = 6.6 Hz, 1H, = CH (geranyl)), 7.12 (d, *J* = 3 Hz, 1H, H-5 (coumarin)), 7.34–7.38 (dd, *J* = 2.7 and 9.3 Hz, 1H, H-7 (coumarin)), 7.45 (d, *J* = 9.3 Hz, 1H, H-8 (coumarin)), 8.89 (s, 1H, H-4 (coumarin)), 12.41 (s, 1H, COOH); ^13^C NMR (75 MHz, CDCl_3_): δ = 16.81, 17.73, 25.68, 26.23, 39.51, 56.82, 112.07, 114.87, 118.24, 118.33, 118.86, 123.54, 125.05, 132.03, 142.62, 149.11, 151.30, 156.49, 162.60, 164.26 ppm; IR (KBr): 3054, 2965, 1751, 1688, 1618 cm^−1^; MS (*m/z*) 340 (M^+^), 338, 205, 187, 161; Anal. calcd for C_20_H_22_O_5_: C 70.16, H 6.48, Found: C, 70.20; H, 6.50%.

#### 6-((2E,6E)-3,7,11-trimethyldodeca-2,6,10-trienyloxy)-2-oxo-2H-chromene-3-carboxylic acid (8d)

Yellow needle, 68% yield; mp: 98–99°C; ^1^H NMR (300 MHz, CDCl_3_): δ = 1.62 (s,3H, CH_3_ (farnesyl)), 1.70 (s, 3H, CH_3_ (farnesyl)), 1.80 (s, 3H CH_3_ (farnesyl)), 1.98–2.18 (m, 8H, 4-CH_2_ (farnesyl)), 4.64 (d, *J* = 6.3 Hz, 2H, -OCH_2_ (farnesyl)), 5.07–5.12 (m, 2H, = CH (farnesyl)), 5.47–5.52 (t, *J* = 5.7 Hz, 1H, = CH (farnesyl)), 7.13 (d, *J* = 2.7 Hz, 1H, H-5 (coumarin)), 7.36–7.40 (dd, *J* = 2.7 and 9.3 Hz, 1H, H-7 (coumarin)), 8.91 (s, 1H, H-4 (coumarin)), 12.41 (s, 1H, COOH); ^13^C NMR (75 MHz, CDCl_3_): δ = 16.06, 16.81, 17.68, 25.68, 26.11, 26.70, 39.51, 39.67, 65.81, 112.07, 114.90, 118.23, 118.38, 118.87, 123.40, 124.24, 125.01, 131.39, 135.66, 142.60, 149.11, 151.26, 156.49, 162.58, 164.24 ppm; IR (KBr): 3047, 2966, 1745, 1688, 1620 cm^−1^; MS (*m/z*) 410 (M^+^), 204, 188; Anal. calcd for C_25_H_30_O_5_: C 73.15, H 7.37, Found: C 73.14, H 7.35%.

#### 7-(allyloxy)-2-oxo-2H-chromene-3-carboxylic acid (12a)

White needle, 76% yield; mp: 205–207°C; ^1^H NMR (300 MHz, CDCl_3_): δ = 4.71 (d, *J* = 5.4 Hz, 2H, -OCH_2_ (Allyl)), 5.40–5.44 (dd, *J* = 1.5 and 10.5 Hz, 1H, = CH_2_ (Allyl)), 5.46–5.52 (dd, *J* = 1.2 and 15.6 Hz, 1H, = CH_2_ (Allyl)), 6.09–6.14 (m, 1H, = CH (Allyl)), 6.97 (d, *J* = 2.4 Hz, 1H, H-8 (coumarin)), 7.05–7.08 (dd, *J* = 2.4 and 8.7 Hz,1H, H-6 (coumarin)), 7.67 (d, *J* = 8.7 Hz, 1H, H-5 (coumarin)), 8.89 (s, 1H, H-4 (coumarin)), 12.21 (bs, 1H, COOH); ^13^C NMR (75 MHz, CDCl_3_): δ = 69.83, 101.67, 111.05, 112.42, 115.57, 119.26, 131.33, 131.71, 151.18, 156.98, 163.04, 164.54, 165.20 ppm; IR (KBr): 3047, 2652, 2781, 1737, 1682, 1602 cm^−1^; MS (*m/z*) 246 (M^+^), 243, 200, 186; Anal. calcd for C_13_H_10_O_5_: C 63.42, H 4.09, Found: C 63.37, H 4.01%.

#### 7-(3-methylbut-2-enyloxy)-2-oxo-2H-chromene-3-carboxylic acid (12b)

White needle, 70% yield; mp: 147–148°C; ^1^H NMR (300 MHz, CDCl_3_): δ = 1.73 (s, 6H, 2CH_3_ (prenyl)), 4.58 (d, *J* = 6.9 Hz, 2H, -OCH_2_ (prenyl)), 5.37–5.43 (m, 1H, = CH (prenyl)), 6.85 (d, *J* = 2.1 Hz, 1H, H-8 (coumarin)), 6.92–6.96 (dd, *J* = 2.4 and 8.7 Hz, 1H, H-6 (coumarin)), 7.56 (d, *J* = 8.7 Hz, 1H, H-5 (coumarin)), 8.78 (s, 1H, H-4 (coumarin)), 12.14 (s, 1H, COOH); ^13^C NMR (75 MHz, CDCl_3_): 18.35, 25.02, 66.11, 101.48, 110.72, 112.21, 115.71, 117.85, 131.65, 140.25, 151.22, 157.06, 163.13, 164.63, 165.65 ppm; IR (KBr): 3052, 2965, 2785, 1737, 1691, 1620 cm^−1^; MS (*m/z*) 275 (M^+^), 273, 271, 204, 187, 161; C, Anal. calcd for C_15_H_14_O_5_: C 65.69, H 5.15, Found: C 65.60, H 5.10%.

#### 7-((E)-3,7-dimethylocta-2,6-dienyloxy)-2-oxo-2H-chromene-3-carboxylic acid (12c)

White needle, 64% yield; mp: 118–119°C; ^1^H NMR (300 MHz, CDCl_3_): δ = 1.63 (s, 3H, CH_3_ (geranyl)), 1.69 (s, 3H, CH_3_ (geranyl)), 1.81 (s, 3H CH_3_ (geranyl)), 2.15–2.20 (m, 4H, 2-CH_2_ (geranyl)), 4.66–4.72 (dd, *J* = 6 and 13.5 Hz, 2H, -OCH_2_ (geranyl)), 5.11–5.15 (m, 1H, = CH (geranyl)), 5.47–5.51 (t, *J* = 6.9 Hz, 1H, = CH (geranyl)), 6.95 (d, *J* = 2.1 Hz, 1H, H-8 (coumarin)), 7.02–7.06 (dd, *J* = 2.4 and 8.7 Hz, 1H, H-6 (coumarin)), 7.65 (d, *J* = 8.7 Hz, 1H, H-5 (coumarin)), 8.88 (s, 1H, H-4 (coumarin)), 12.24 (s, 1H, COOH); ^13^C NMR (75 MHz, CDCl_3_): δ = 16.84, 17.71, 25.65, 26.19, 39.49, 66.17, 101.51, 110.73, 112.20, 115.72, 117.64, 123.45, 131.62, 132.10, 143.36, 151.21, 157.06, 163.13, 164.63, 165.67 ppm; IR (KBr): 3051, 2962, 1739, 1690, 1619 cm^−1^; MS (*m/z*) 342 (M^+^), 340, 205, 187, 161; Anal. calcd for C_20_H_22_O_5_: C 70.16, H 6.48, Found: C, 70.12; H, 6.44%.

#### 7-((2E,6E)-3,7,11-trimethyldodeca-2,6,10-trienyloxy)-2-oxo-2H-chromene-3-carboxylic acid (12d)

White needle, 58% yield; mp: 87–90°C; ^1^H NMR (300 MHz, CDCl_3_): δ = 1.52 (s, 3H, CH_3_ (farnesyl)), 1.53 (s, 3H, CH_3_ (farnesyl)), 1.6 (s, 3H CH_3_ (farnesyl)), 1.71 (s, 3H CH_3_ (farnesyl)), 1.87–2.09 (m, 8H, 4-CH_2_ (farnesyl)), 4.61 (d, *J* = 6.6 Hz, 2H, -OCH_2_ (farnesyl)), 4.97–5.01 (m, 2H, = CH (farnesyl)), 5.31–5.41 (t, *J* = 6.6 Hz, 1H, = CH (farnesyl)), 6.84 (d, *J* = 2.1 Hz, 1H, H-8 (coumarin)), 6.92–6.95 (dd, *J* = 2.4 and 8.7 Hz, 1H, H-6 (coumarin)), 7.54 (d, *J* = 8.7 Hz, 1H, H-5 (coumarin)), 8.78 (s, 1H, H-4 (coumarin)), 12.14 (s, 1H, COOH); ^13^C NMR (75 MHz, CDCl_3_): δ = 16.06, 16.84, 17.68, 25.68, 26.07, 26.69, 39.49, 39.66, 66.16, 101.50, 110.73, 112.21, 115.71, 117.67, 123.31, 124.23, 131.40, 131.62, 135.75, 143.36, 151.20, 157.06, 163.13, 164.63, 165.67 ppm; IR (KBr): 3047, 1973, 1739, 1688, 1617 cm^−1^; MS (*m/z*) 410 (M^+^), 205, 188, 161; Anal. calcd for C_25_H_30_O_5_: C 73.15, H 7.37, Found: C, 73.18; H, 7.40%.

#### 8-(allyloxy)-2-oxo-2H-chromene-3-carboxylic acid (18a)

Brown needle, 76% yield; mp: 198–200°C; ^1^H NMR (300 MHz, CDCl_3_): δ = 4.76–4.78 (m, 2H, -OCH_2_ (Allyl)), 5.37–5.54 (m, 2H, = CH_2_ (Allyl)), 6.05–6.18 (m,1H, = CH (Allyl)), 7.29–7.43 (m, 3H, 3H, H-7,H-6, H-5 (coumarin)), 8.94 (s, 1H, H-4 (coumarin)), 12.28 (bs, 1H, COOH); ^13^C NMR (75 MHz, CDCl_3_): δ = 70.31, 115.01, 118.84, 118.97, 119.27, 121.65, 126.08, 131.92, 144.45, 146.35, 151.73, 162.51, 163.79 ppm; IR (KBr): 3084, 2925, 2794, 1746, 1677, 1609 cm^−1^; MS (*m/z*) 246 (M^+^), 245, 204, 161; Anal. calcd for C_13_H_10_O_5_: C 63.42, H 4.09, Found: C, 63.40; H, 4.05%.

#### 8-(3-methylbut-2-enyloxy)-2-oxo-2H-chromene-3-carboxylic acid (18b)

Brown needle, 71% yield; mp: 143–144°C; ^1^H NMR (400 MHz, CDCl_3_): δ = 1.79 (s, 3H, CH_3_ (prenyl)), 1.83 (s, 3H, CH_3_ (prenyl)), 4.73 (d, *J* = 6.8 Hz, 2H, -OCH_2_ (prenyl)), 5.52–5.55 (m, 1H, = CH (prenyl)), 7.28–7.40 (m, 3H, H-7,H-6, H-5 (coumarin)), 8.93 (s, 1H, H-4 (coumarin)), 12.34 (s, 1H, COOH); ^13^C NMR (100 MHz, CDCl_3_): δ = 18.35, 25.82, 66.47, 114.94, 118.65, 118.74, 119.23, 121.28, 126.07, 139.50, 144.58, 146.70, 151.75, 162.54, 163.88; IR (KBr): 3064, 2962, 1741, 1691, 1606 cm^−1^; MS (*m/z*), 206, 205, 187, 161; Anal. calcd for C_15_H_14_O_5_: C 65.69, H 5.15, Found: C 65.70, H 5.17%.

#### 8-((E)-3,7-dimethylocta-2,6-dienyloxy)-2-oxo-2H-chromene-3-carboxylic acid (18c)

Brown needle, 61% yield; mp: 100–101°C; ^1^H NMR (400 MHz, CDCl_3_): δ = 2.01(s, 3H, CH_3_ (geranyl)), 2.08 (s, 3H, CH_3_ (geranyl)), 2.11 (s, 3H, CH_3_ (geranyl)), 2.13–2.20 (m, 4H, 2-CH_2_ (geranyl)), 4.78 (d, *J* = 6.8 Hz, 2H, -OCH_2_ (geranyl)), 5.08–5.10 (m, 1H, = CH (geranyl)), 5.25–5.55 (t, *J* = 5.6 Hz, 1H, = CH (geranyl)), 7.29–7.41 (m, 3H, H-7,H-6, H-5 (coumarin)), 8.94 (s, 1H, H-4 (coumarin)), 12.27 (bs, 1H, COOH); ^13^C NMR (100 MHz, CDCl_3_): δ = 16.84, 17.76, 25.73, 26.52, 39.51, 66.51, 118.36, 118.62, 118.76, 119.20, 121.30, 123.56, 126.11, 132.06, 142.57, 144.52, 146.64, 151.84, 162.63, 163.89; IR (KBr): 3047, 2922, 1725, 1684, 1607 cm^−1^; MS (*m/z*) 342 (M^+^), 254, 205, 187, 162; Anal. calcd for C_20_H_22_O_5_: C 70.16, H 6.48, Found: C, 70.11; H, 6.46%.

#### 8-((2E,6E)-3,7,11-trimethyldodeca-2,6,10-trienyloxy)-2-oxo-2H-chromene-3-carboxylic acid (18d)

Brown needle, 59% yield; mp: 987–90°C; ^1^H NMR (400 MHz, CDCl_3_): δ = 1.62 (s, 6H, 2-CH_3_ (farnesyl)), 1.70 (s, 3H, CH_3_ (farnesyl)), 1.80 (s, 3H, CH_3_ (farnesyl)), 1.98–2.20 (m, 8H, 4-CH_2_ (farnesyl)), 4.78 (d, *J* = 6.4 Hz, 2H, -OCH_2_ (farnesyl)), 5.08–5.11 (m, 2H, = CH (farnesyl)), 5.53–5.56 (t, *J* = 5.6 Hz, 1H, = CH (farnesyl)), 7.29–7.41 (m, 3H, H-7,H-6, H-5 (coumarin)), 8.94 (s, 1H, H-4 (coumarin)), 12.30 (bs, 1H, COOH); ^13^C NMR (100 MHz, CDCl_3_): δ = 16.07, 16.87, 17.72, 25.74, 26.69, 39.52, 39.69, 66.47, 114.83, 118.36, 118.72, 119.20, 121.29, 123.44, 124.27, 126.10, 131.39, 135.65, 142.60, 144.50, 146.62, 151.81, 162.63, 163.87 ppm; IR (KBr): 3047, 2966, 1751, 1683, 1606 cm^−1^; MS (*m/z*) 410 (M^+^), 391, 204, 187, 161; Anal. calcd for C_25_H_30_O_5_: C 73.15, H 7.37, Found: C, 73.11; H, 7.32%.

## Supporting information

S1 Fig CNMR, HNMR and mass spectra of all of the new synthetic compounds. (PDF)
