# Supplementary material for: O-prenylated 3-carboxycoumarins as a novel class of 15-LOX-1 inhibitors
Source: PLoS One. 2017 Feb 9;12(2):e0171789. doi: 10.1371/journal.pone.0171789 (PMC5300203; doi:10.1371/journal.pone.0171789)
Supplement: S1 Fig — (PDF) [file pone.0171789.s001.pdf]

Table of content

^{1}H NMR spectra of compound 16……………………………………………………………………….6

^{1}H NMR spectra of compound 3a ……………………………………………………………….7

^{13}C NMR spectra of compound 3a……………………………………………………………………….8

Mass spestra of compound 3a………………………………………………………………………………9

^{1}H NMR spectra of compound 3b……………………………………………………………………......10

^{13}C NMR spectra of compound 3b…………………………………………………………………….....11

Mass spestra of compound 3b……………………………………………………………………………..12

^{1}H NMR spectra of compound 3c……………………………………………………………………......13

^{13}C NMR spectra of compound 3c…………………………………………………………………......14

Mass spestra of compound 3c……………………………………………………………………….…15

^{1}H NMR spectra of compound 3d……………………………………………………………………….16

^{13}C NMR spectra of compound 3d…………………………………………………………………...17

Mass spestra of compound 3d…………………………………………………………………………….18

^{1}H NMR spectra of compound7a……………………………………………………………………… 19

^{13}C NMR spectra of compound7a………………………………………………………………………20

Mass spestra of compound 7a…………………………………………………………………….….21

^{1}H NMR spectra of compound 7b………………………………………………………………………22

^{13}C NMR spectra of compound 7b……………………………………………………………………..23

Mass spestra of compound 7b……………………………………………………………………………….24

^{1}H NMR spectra of compound 7c……………………………………………………………………….25

^{13}C NMR spectra of compound 7c……………………………………………………………………….26

Mass spestra of compound 7c………………………………………………………………………………27

^{1}H NMR spectra of compound 7d………………………………………………………………………..28

^{13}C NMR spectra of compound 7d……………………………………………………………………….29

Mass spestra of compound 7d………………………………………………………………………………30

^{1}H NMR spectra of compound 11a……………………………………………………………………….31

^{13}C NMR spectra of compound 11a…………………………………………………………………….…32

Mass spestra of compound 11a……………………………………………………………………………33

^{1}H NMR spectra of compound 11b ……………………………………………………………………..34

^{13}C NMR spectra of compound 11b……………………………………………………………………..35

Mass spestra of compound 11b…………………………………………………………………………….36

^{1}H NMR spectra of compound 11c……………………………………………………………………………37

^{13}C NMR spectra of compound 11c…………………………………………………………………………38

Mass spestra of compound 11c……………………………………………………………………………39

^{1}H NMR spectra of compound 11d……………………………………………………………………….40

^{13}C NMR spectra of compound 11d……………………………………………………………………….41

Mass spestra of compound 11d……………………………………………………………………………42

^{1}H NMR spectra of compound 17a……………………………………………………………………………43

^{13}C NMR spectra of compound 17a…………………………………………………………………………44

Mass spestra of compound 17a……………………………………………………………………………..45

^{1}H NMR spectra of compound 17b…………………………………………………………………………….46

^{13}C NMR spectra of compound 17b…………………………………………………………………………….47

Mass spestra of compound 17b……………………………………………………………………………48

^{1}H NMR spectra of compound 17c……………………………………………………………………………49

^{13}C NMR spectra of compound 17c……………………………………………………………………………50

Mass spestra of compound 17c……………………………………………………………………………..51

^{1}H NMR spectra of compound 17d…………………………………………………………………………….52

^{13}C NMR spectra of compound 17d……………………………………………………………………………53

Mass spestra of compound 17d……………………………………………………………………………54

^{1}H NMR spectra of compound 4a……………………………………………………………………………55

^{13}C NMR spectra of compound 4a……………………………………………………………………………56

Mass spestra of compound 4a…………………………………………………………………………….57

^{1}H NMR spectra of compound 4b……………………………………………………………………………58

^{13}C NMR spectra of compound 4b……………………………………………………………………………59

Mass spestra of compound 4b……………………………………………………………………………..60

^{1}H NMR spectra of compound 4c……………………………………………………………………………..61

^{13}C NMR spectra of compound 4c…………………………………………………………………………...62

Mass spestra of compound 4c……………………………………………………………………………63

^{1}H NMR spectra of compound 4d…………………………………………………………………………….64

^{13}C NMR spectra of compound 4d……………………………………………………………………………..65

Mass spestra of compound 4d………………………………………………………………………………..66

^{1}H NMR spectra of compound 8a………………………………………………………………………….67

^{13}C NMR spectra of compound 8a…………………………………………………………………………68

Mass spestra of compound 8a………………………………………………………………………………69

^{1}H NMR spectra of compound 8b…………………………………………………………………………..70

^{13}C NMR spectra of compound 8b…………………………………………………………………………..71

Mass spestra of compound 8b………………………………………………………………………………..72

^{1}H NMR spectra of compound 8c……………………………………………………………………………73

^{13}C NMR spectra of compound 8c……………………………………………………………………………74

Mass spestra of compound 8c…………………………………………………………………………………75

^{1}H NMR spectra of compound 8d……………………………………………………………………………76

^{13}C NMR spectra of compound 8d……………………………………………………………………………77

Mass spestra of compound 8d…………………………………………………………………………………78

^{1}H NMR spectra of compound 12a…………………………………………………………………………..79

^{13}C NMR spectra of compound 12a…………………………………………………………………………..80

Mass spestra of compound 12a……………………………………………………………………………81

^{1}H NMR spectra of compound 12b…………………………………………………………………………82

^{13}C NMR spectra of compound 12b…………………………………………………………………………83

Mass spestra of compound 12b………………………………………………………………………………84

^{1}H NMR spectra of compound 12c…………………………………………………………………………………85

^{13}C NMR spectra of compound 12c…………………………………………………………………………………86

Mass spestra of compound 12c……………………………………………………………………………………..87

^{1}H NMR spectra of compound 12d……………………………………………………………………………………..88

^{13}C NMR spectra of compound 12d…………………………………………………………………………………..89

Mass spestra of compound 12d…………………………………………………………………………………….90

^{1}H NMR spectra of compound 18a………………………………………………………………………………….91

^{13}C NMR spectra of compound 18a…………………………………………………………………………………….92

Mass spestra of compound 18a……………………………………………………………………………………….93

^{1}H NMR spectra of compound 18b……………………………………………………………………………………..94

^{13}C NMR spectra of compound 18b…………………………………………………………………………………95

Mass spestra of compound 18b………………………………………………………………………………………96

^{1}H NMR spectra of compound 18c………………………………………………………………………………..…97

^{13}C NMR spectra of compound 18c……………………………………………………………………………………..98

Mass spestra of compound 18c……………………………………………………………………………………….99

^{1}H NMR spectra of compound 18d………………………………………………………………………………….100

^{13}C NMR spectra of compound 18d…………………………………………………………………………………101

Mass spestra of compound 18d……………………………………………………………………………………….102

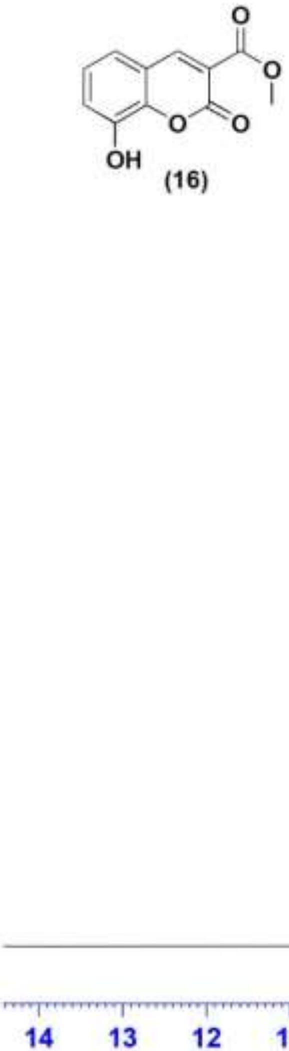

(16)

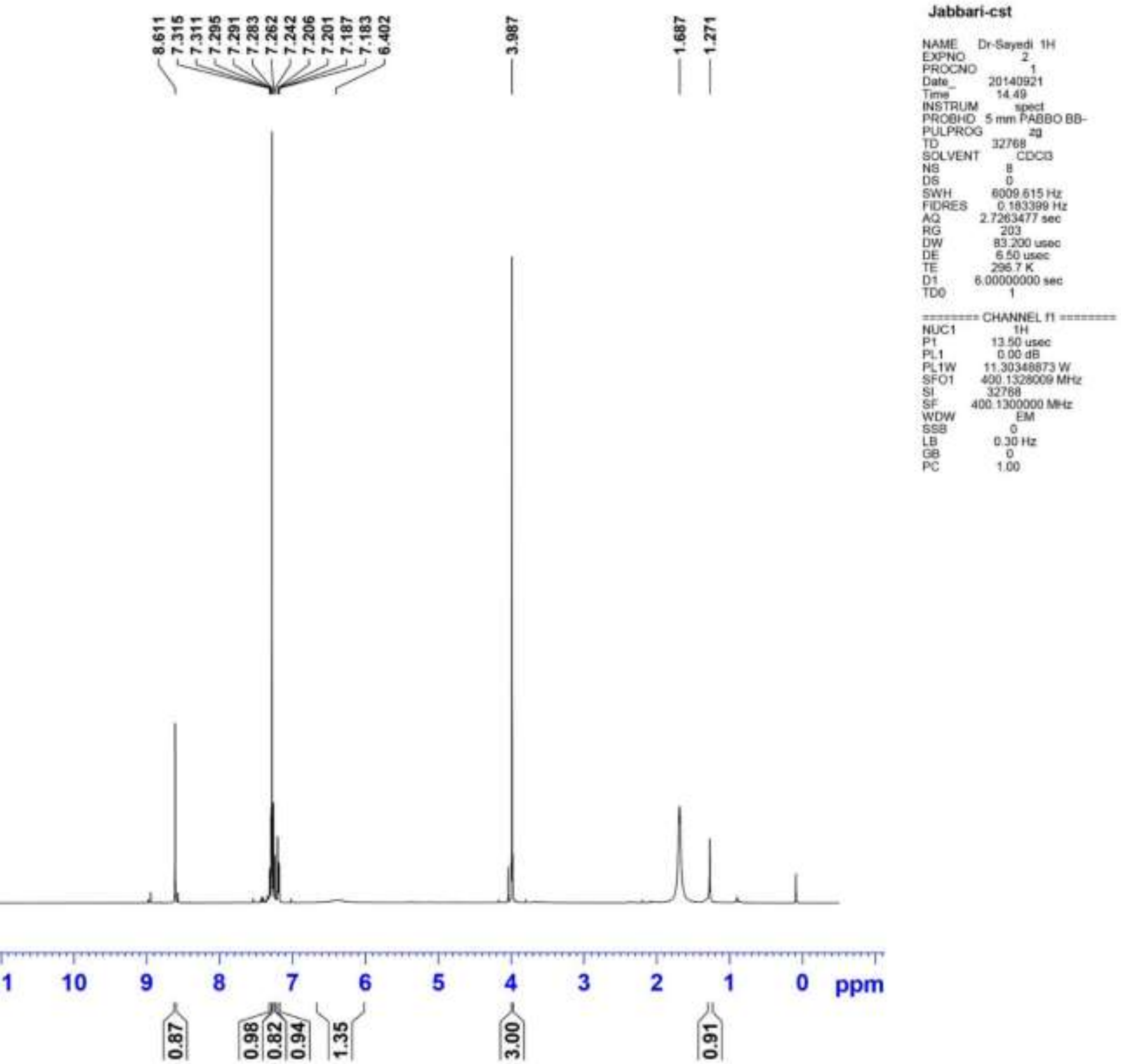
8.611
7.315
7.311
7.295
7.291
7.283
7.262
7.242
7.206
7.201
7.187
7.183
6.402
3.987
1.687
1.271
14 13 12 11 10 9 8 7 6 5 4 3 2 1 0 ppm
0.87
0.98
0.82
0.94
1.35
3.00
0.91
Jabbari-cst
NAME Dr-Sayedi 1H
EXPNO 2
PROCNO 1
Date_ 20140921
Time 14.49
INSTRUM spect
PROBHD 5 mm PABBO BB-
PULPROG zg
TD 32768
SOLVENT CDCl3
NS 8
DS 0
SWH 6009.615 Hz
FIDRES 0.183399 Hz
AQ 2.7263477 sec
RG 203
DW 83.200 usec
DE 6.50 usec
TE 296.7 K
D1 6.00000000 sec
TD0 1
======== CHANNEL f1 ========
NUC1 1H
P1 13.50 usec
PL1 0.00 dB
PL1W 11.30348873 W
SFO1 400.1328009 MHz
SI 32768
SF 400.1300000 MHz
WDW EM
SSB 0
LB 0.30 Hz
GB 0
PC 1.00

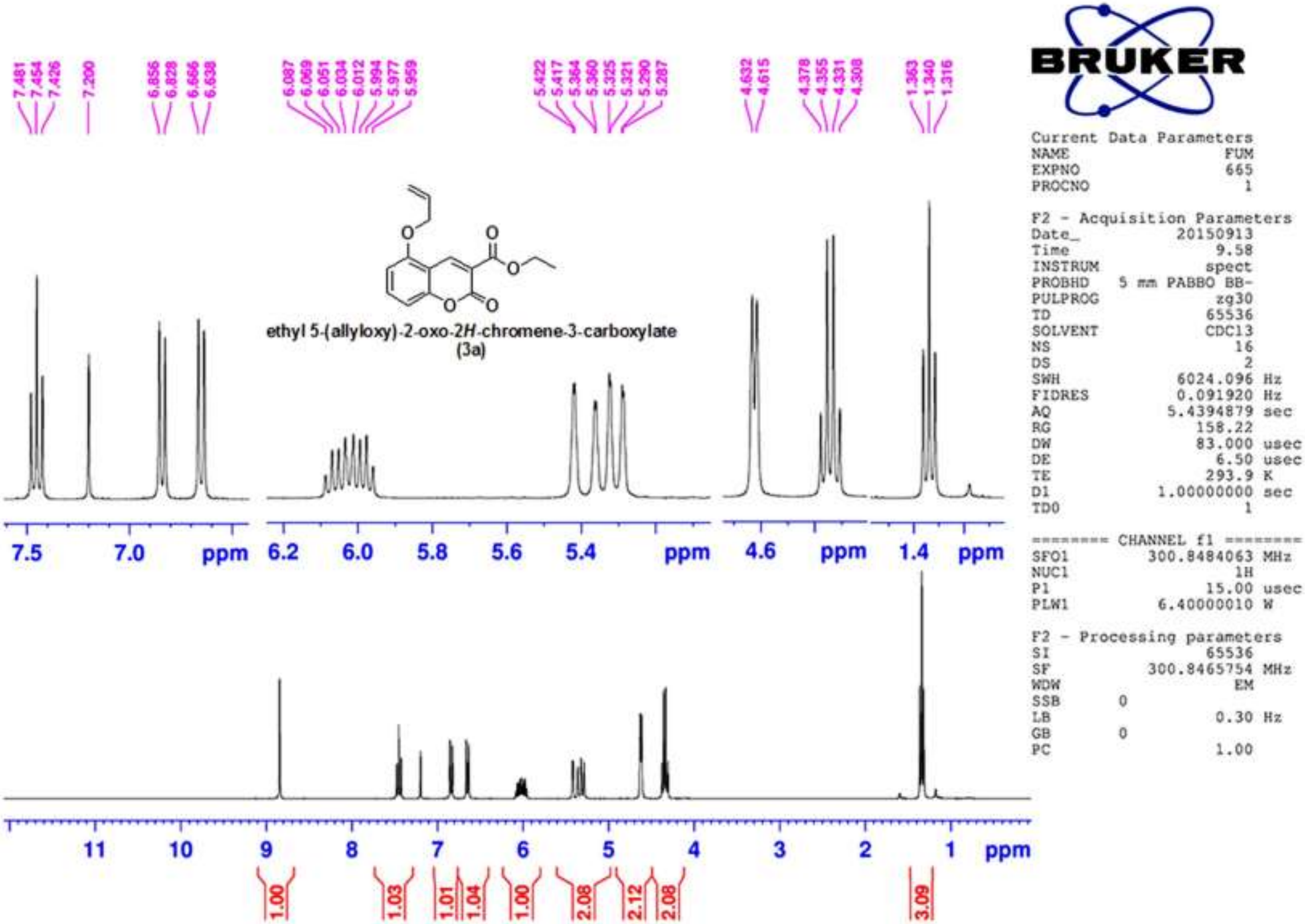
7.481
7.454
7.426
7.200
6.856
6.828
6.666
6.638
6.087
6.069
6.051
6.034
6.012
5.994
5.977
5.959
5.422
5.417
5.364
5.360
5.325
5.321
5.290
5.287
4.632
4.615
4.378
4.355
4.331
4.308
1.363
1.340
1.316
ethyl 5-(allyloxy)-2-oxo-2H-chromene-3-carboxylate
(3a)
7.5
7.0
ppm
6.2
6.0
5.8
5.6
5.4
ppm
4.6
ppm
1.4
ppm
11
10
9
8
7
6
5
4
3
2
1
ppm
1.00
1.03
1.01
1.04
1.00
2.08
2.12
2.08
3.09
BRUKER
Current Data Parameters
NAME FUM
EXPNO 665
PROCNO 1
F2 - Acquisition Parameters
Date_ 20150913
Time 9.58
INSTRUM spect
PROBHD 5 mm PABBO BB-
PULPROG zg30
TD 65536
SOLVENT CDCl3
NS 16
DS 2
SWH 6024.096 Hz
FIDRES 0.091920 Hz
AQ 5.4394879 sec
RG 158.22
DW 83.000 usec
DE 6.50 usec
TE 293.9 K
D1 1.00000000 sec
TD0 1
======== CHANNEL f1 ========
SFO1 300.8484063 MHz
NUC1 1H
P1 15.00 usec
PLW1 6.40000010 W
F2 - Processing parameters
SI 65536
SF 300.8465754 MHz
WDW EM
SSB 0
LB 0.30 Hz
GB 0
PC 1.00

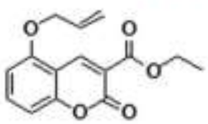

Ethyl 5-(allyloxy)-2-oxo-2H-chromene-3-carboxylate
(3a)

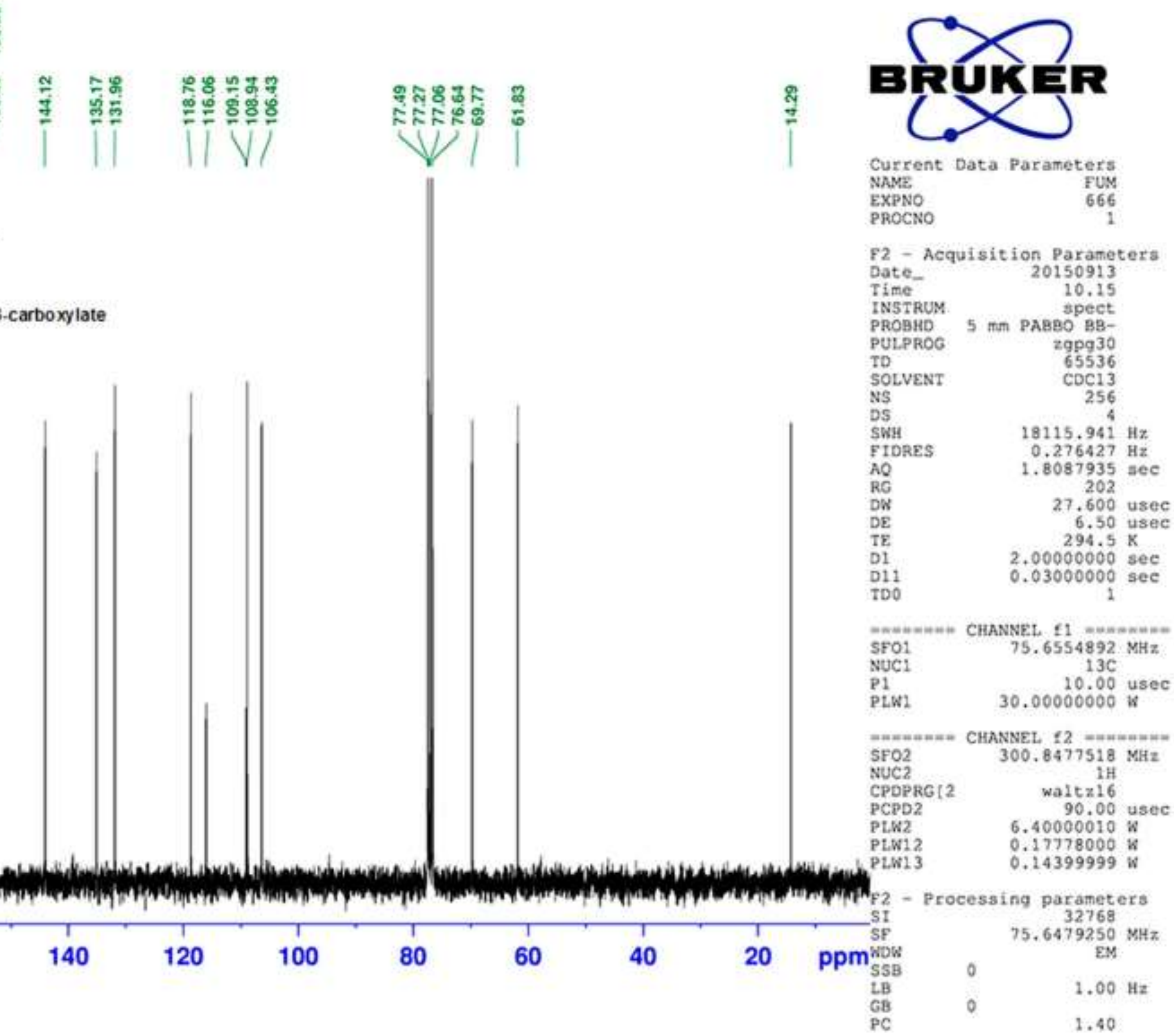

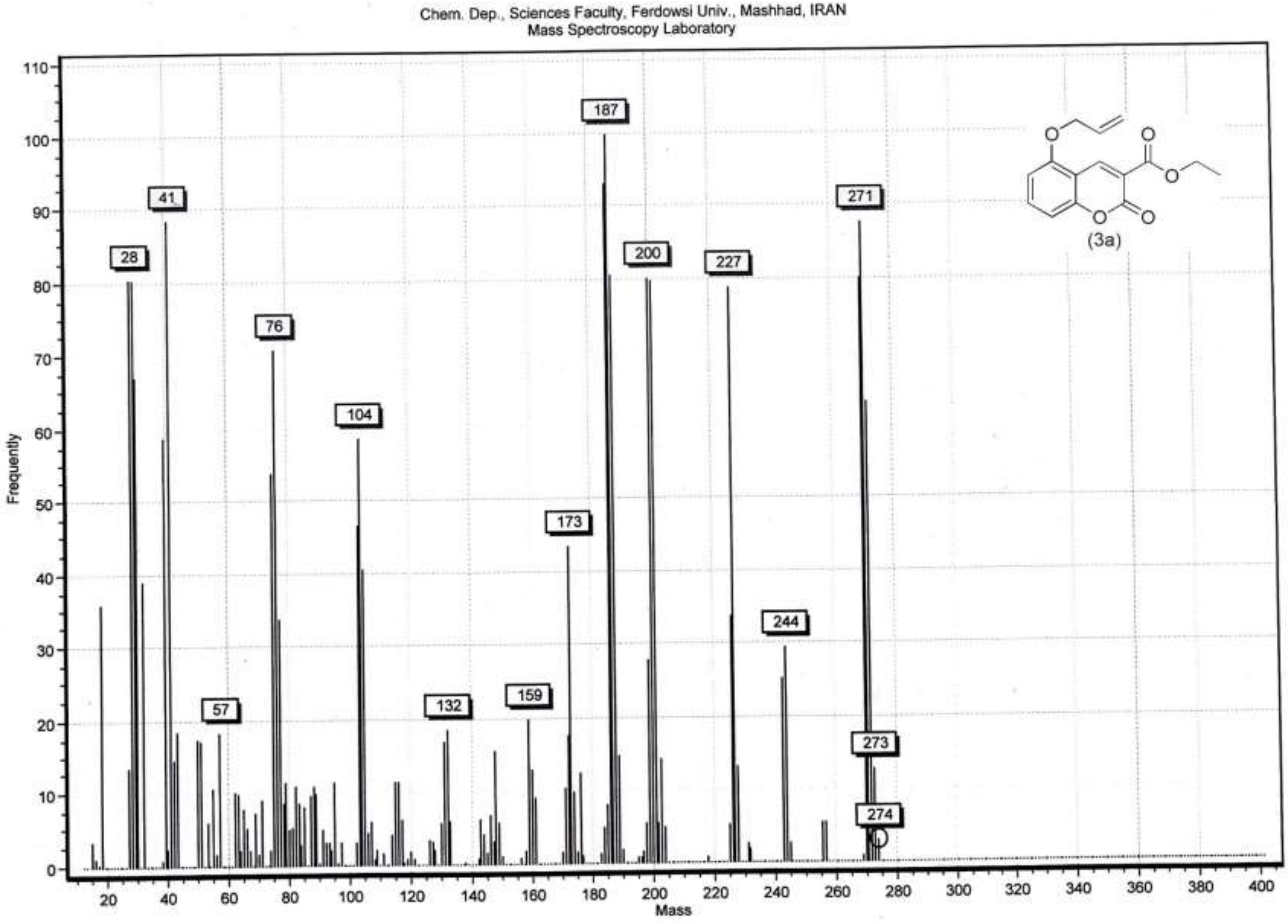
Chem. Dep., Sciences Faculty, Ferdowsi Univ., Mashhad, IRAN
Mass Spectroscopy Laboratory
Frequently
Mass
28
41
57
76
104
132
159
173
187
200
227
244
271
273
274
(3a)

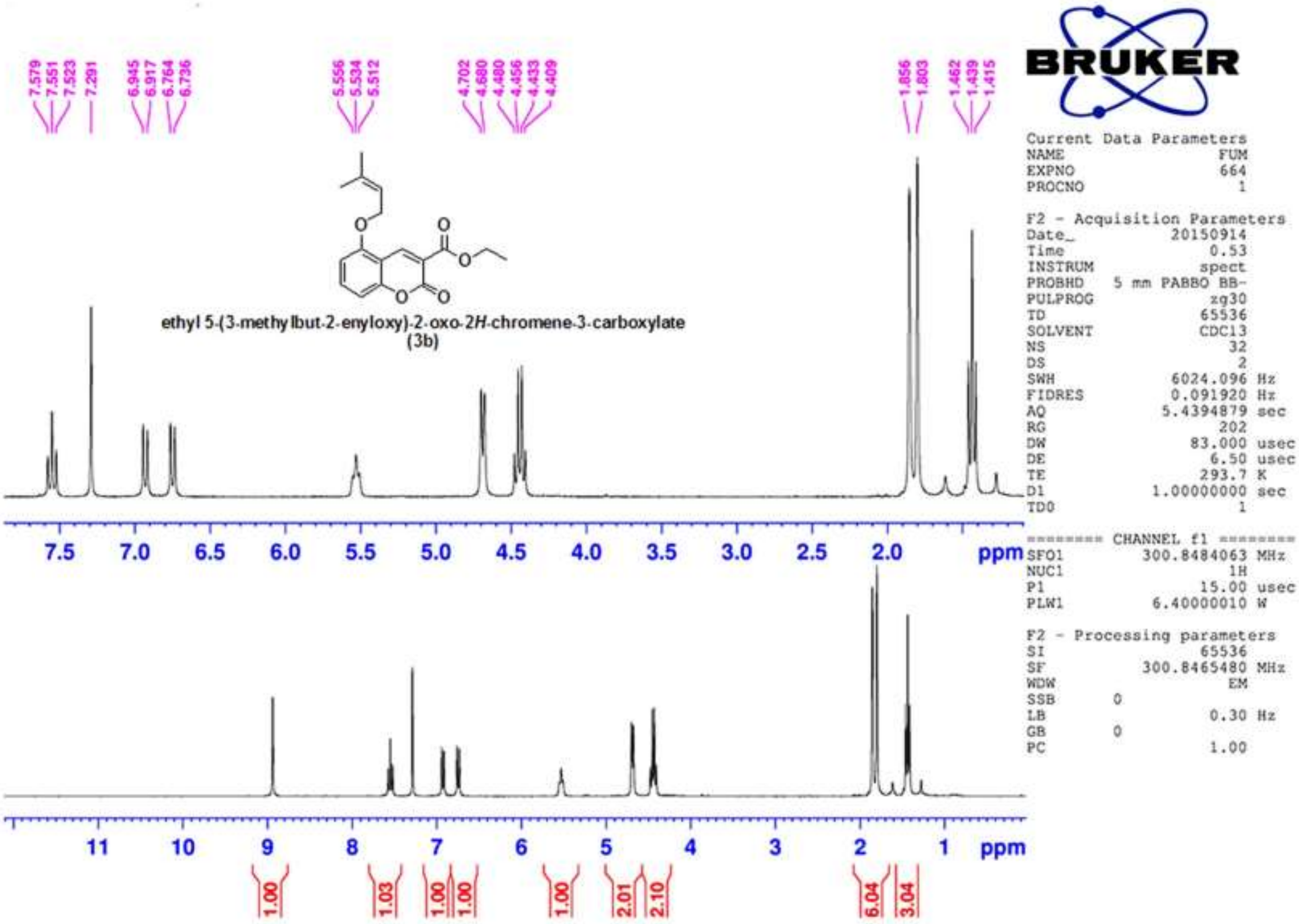
7.579
7.551
7.523
7.291
6.945
6.917
6.764
6.736
5.556
5.534
5.512
4.702
4.680
4.480
4.456
4.433
4.409
1.856
1.803
1.462
1.439
1.415
BRUKER
ethyl 5-(3-methylbut-2-enyloxy)-2-oxo-2H-chromene-3-carboxylate
(3b)
Current Data Parameters
NAME FUM
EXPNO 664
PROCNO 1
F2 - Acquisition Parameters
Date_ 20150914
Time 0.53
INSTRUM spect
PROBHD 5 mm PABBO BB-
PULPROG zg30
TD 65536
SOLVENT CDCl3
NS 32
DS 2
SWH 6024.096 Hz
FIDRES 0.091920 Hz
AQ 5.4394879 sec
RG 202
DW 83.000 usec
DE 6.50 usec
TE 293.7 K
D1 1.00000000 sec
TD0 1
======== CHANNEL f1 ========
SFO1 300.8484063 MHz
NUC1 1H
P1 15.00 usec
PLW1 6.40000010 W
F2 - Processing parameters
SI 65536
SF 300.8465480 MHz
WDW EM
SSB 0
LB 0.30 Hz
GB 0
PC 1.00
7.5 7.0 6.5 6.0 5.5 5.0 4.5 4.0 3.5 3.0 2.5 2.0 ppm
11 10 9 8 7 6 5 4 3 2 1 ppm
1.00
1.03
1.00
1.00
1.00
2.01
2.10
6.04
3.04

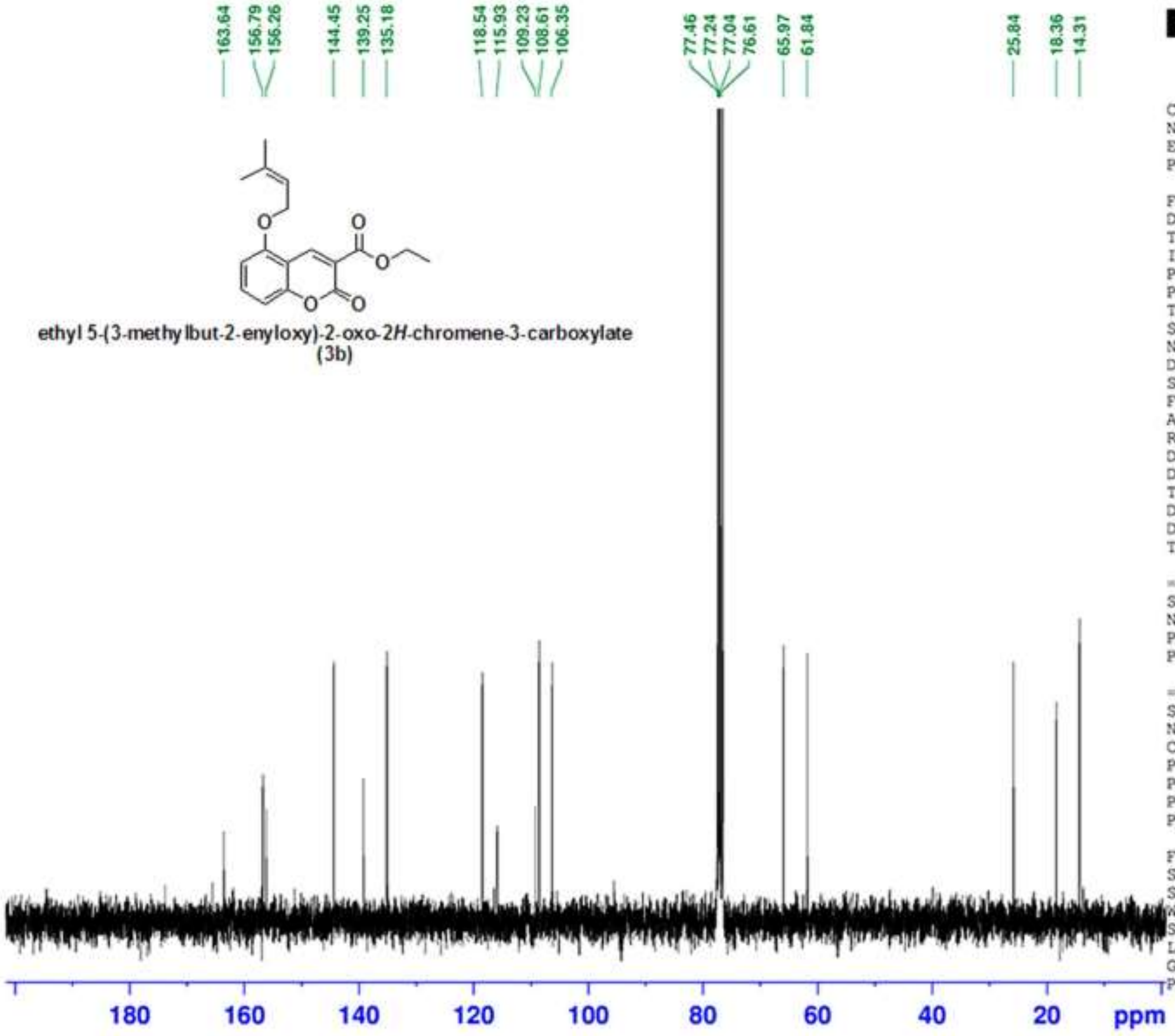

ethyl 5-(3-methylbut-2-enyloxy)-2-oxo-2*H*-chromene-3-carboxylate
(3b)

BRUKER

Current Data Parameters
NAME FUM
EXPNO 710
PROCNO 1

F2 - Acquisition Parameters
Date_ 20150914
Time 1.38
INSTRUM spect
PROBHD 5 mm PABBO BB-
PULPROG zgpg30
TD 65536
SOLVENT CDCl3
NS 688
DS 4
SWH 18115.941 Hz
FIDRES 0.276427 Hz
AQ 1.8087935 sec
RG 202
DW 27.600 usec
DE 6.50 usec
TE 294.5 K
D1 2.00000000 sec
D11 0.03000000 sec
TD0 1

======== CHANNEL f1 ========
SFO1 75.6554892 MHz
NUC1 13C
P1 10.00 usec
PLW1 30.00000000 W

======== CHANNEL f2 ========
SFO2 300.8477518 MHz
NUC2 1H
CPDPRG[2 waltz16
PCPD2 90.00 usec
PLW2 6.40000010 W
PLW12 0.17778000 W
PLW13 0.14399999 W

F2 - Processing parameters
SI 32768
SF 75.6479250 MHz
WDW EM
SSB 0
LB 1.00 Hz
GB 0
PC 1.40

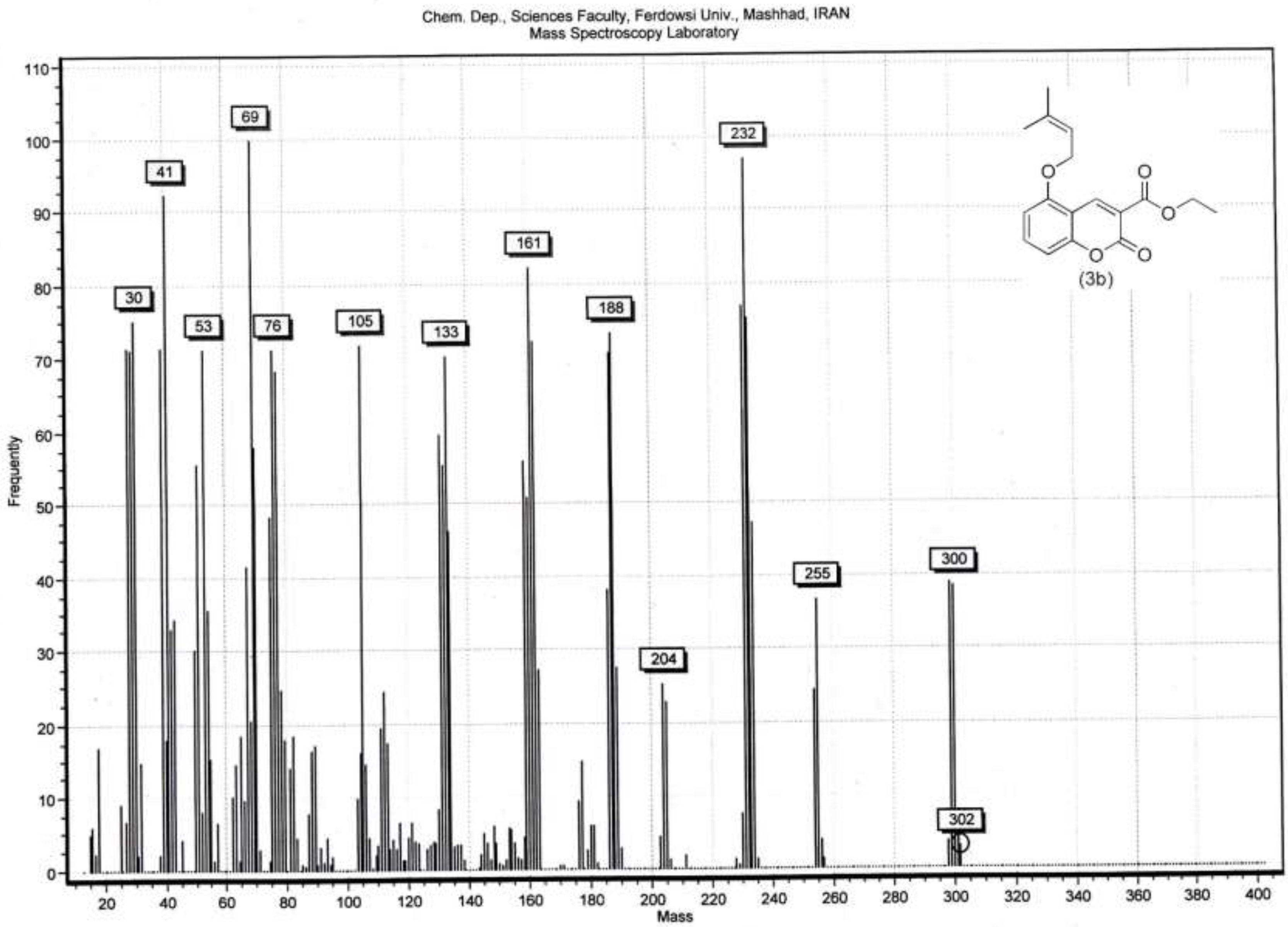
Chem. Dep., Sciences Faculty, Ferdowsi Univ., Mashhad, IRAN
Mass Spectroscopy Laboratory
Frequently
Mass
30
41
53
69
76
105
133
161
188
204
232
255
300
302
(3b)

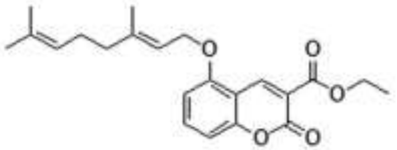

ethyl 5-((*E*)-3,7-dimethylocta-2,6-dienyloxy)-2-oxo-2*H*-chromene-3-carboxylate (3c)

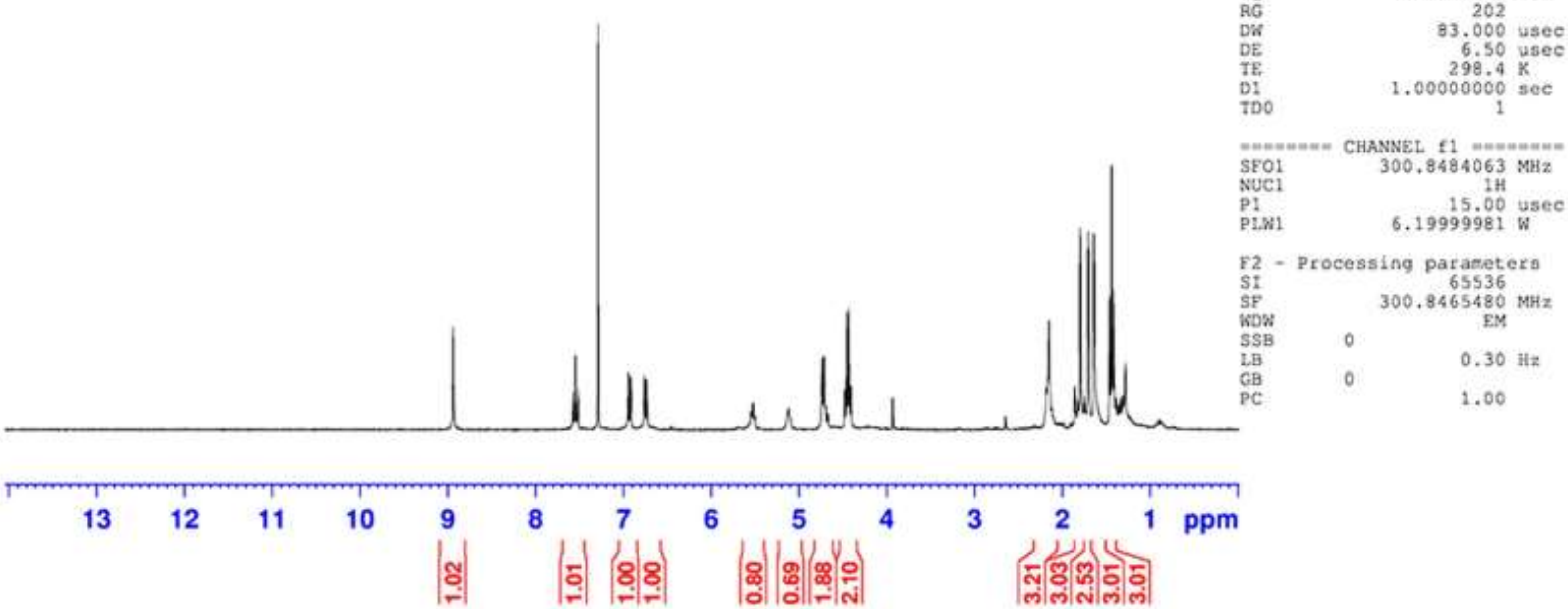

Current Data Parameters
NAME FUM
EXPNO 226
PROCNO 1

F2 - Acquisition Parameters
Date_ 20150621
Time 5.03
INSTRUM spect
PROBHD 5 mm DUL 13C-1
PULPROG zg30
TD 65536
SOLVENT CDCl3
NS 16
DS 2
SWH 6024.096 Hz
FIDRES 0.091920 Hz
AQ 5.4394879 sec
RG 202
DW 83.000 usec
DE 6.50 usec
TE 298.4 K
D1 1.00000000 sec
TD0 1

======== CHANNEL f1 ========
SFO1 300.8484063 MHz
NUC1 1H
P1 15.00 usec
PLW1 6.19999981 W

F2 - Processing parameters
SI 65536
SF 300.8465480 MHz
WDW EM
SSB 0
LB 0.30 Hz
GB 0
PC 1.00

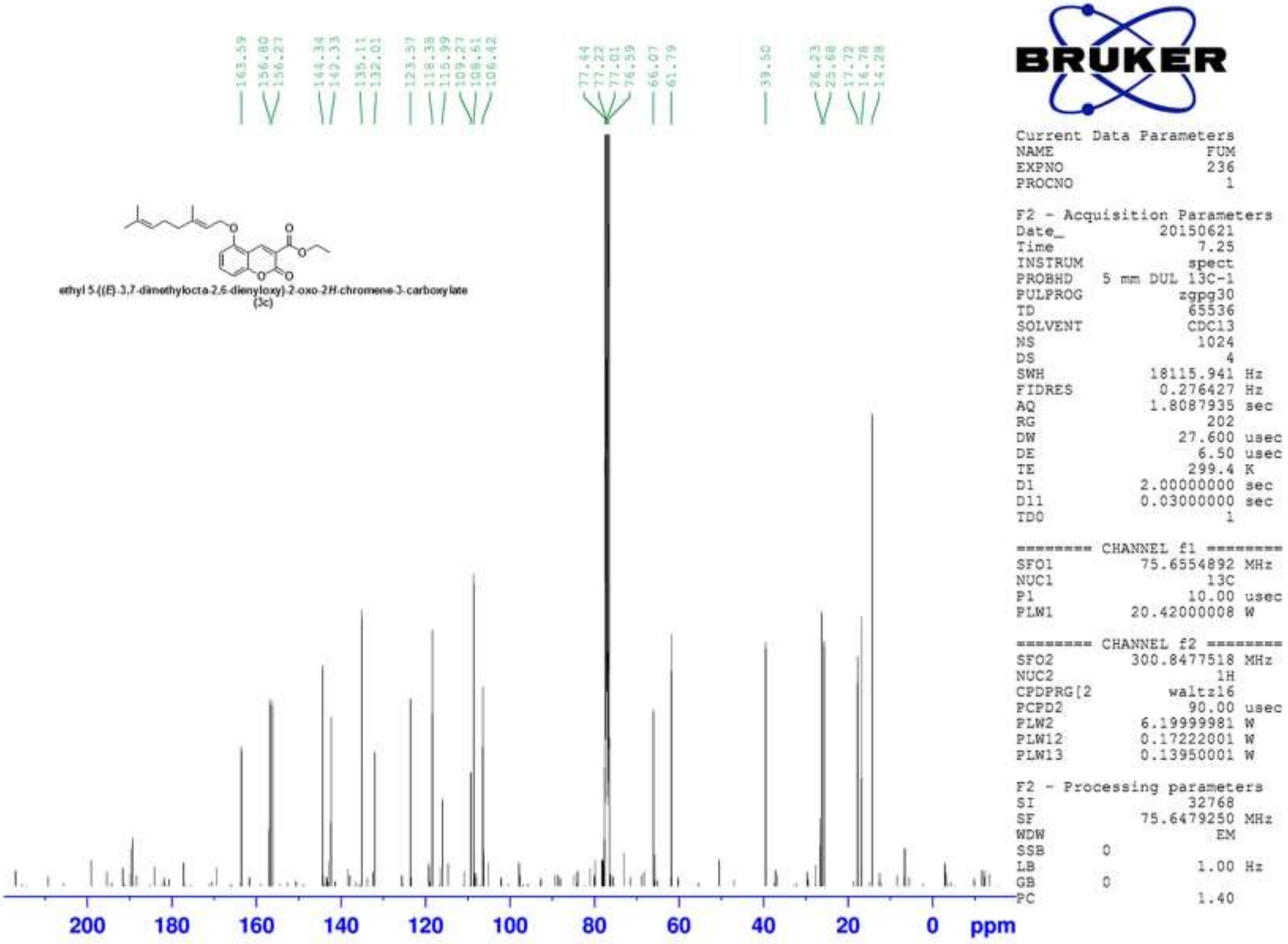
163.59
156.80
156.27
144.34
142.33
135.11
132.01
123.57
118.38
115.99
109.27
108.61
106.42
77.44
77.22
77.01
76.59
66.07
61.79
39.50
26.23
25.68
17.72
16.78
14.28
ethyl 5-((E)-3,7-dimethylocta-2,6-dienyloxy)-2-oxo-2H-chromene-3-carboxylate (3c)
BRUKER
Current Data Parameters
NAME FUM
EXPNO 236
PROCNO 1
F2 - Acquisition Parameters
Date_ 20150621
Time 7.25
INSTRUM spect
PROBHD 5 mm DUL 13C-1
PULPROG zgpg30
TD 65536
SOLVENT CDCl3
NS 1024
DS 4
SWH 18115.941 Hz
FIDRES 0.276427 Hz
AQ 1.8087935 sec
RG 202
DW 27.600 usec
DE 6.50 usec
TE 299.4 K
D1 2.00000000 sec
D11 0.03000000 sec
TD0 1
======== CHANNEL f1 ========
SFO1 75.6554892 MHz
NUC1 13C
P1 10.00 usec
PLW1 20.42000008 W
======== CHANNEL f2 ========
SFO2 300.8477518 MHz
NUC2 1H
CPDPRG[2 waltz16
PCPD2 90.00 usec
PLW2 6.19999981 W
PLW12 0.17222001 W
PLW13 0.13950001 W
F2 - Processing parameters
SI 32768
SF 75.6479250 MHz
WDW EM
SSB 0
LB 1.00 Hz
GB 0
PC 1.40
200
180
160
140
120
100
80
60
40
20
0
ppm

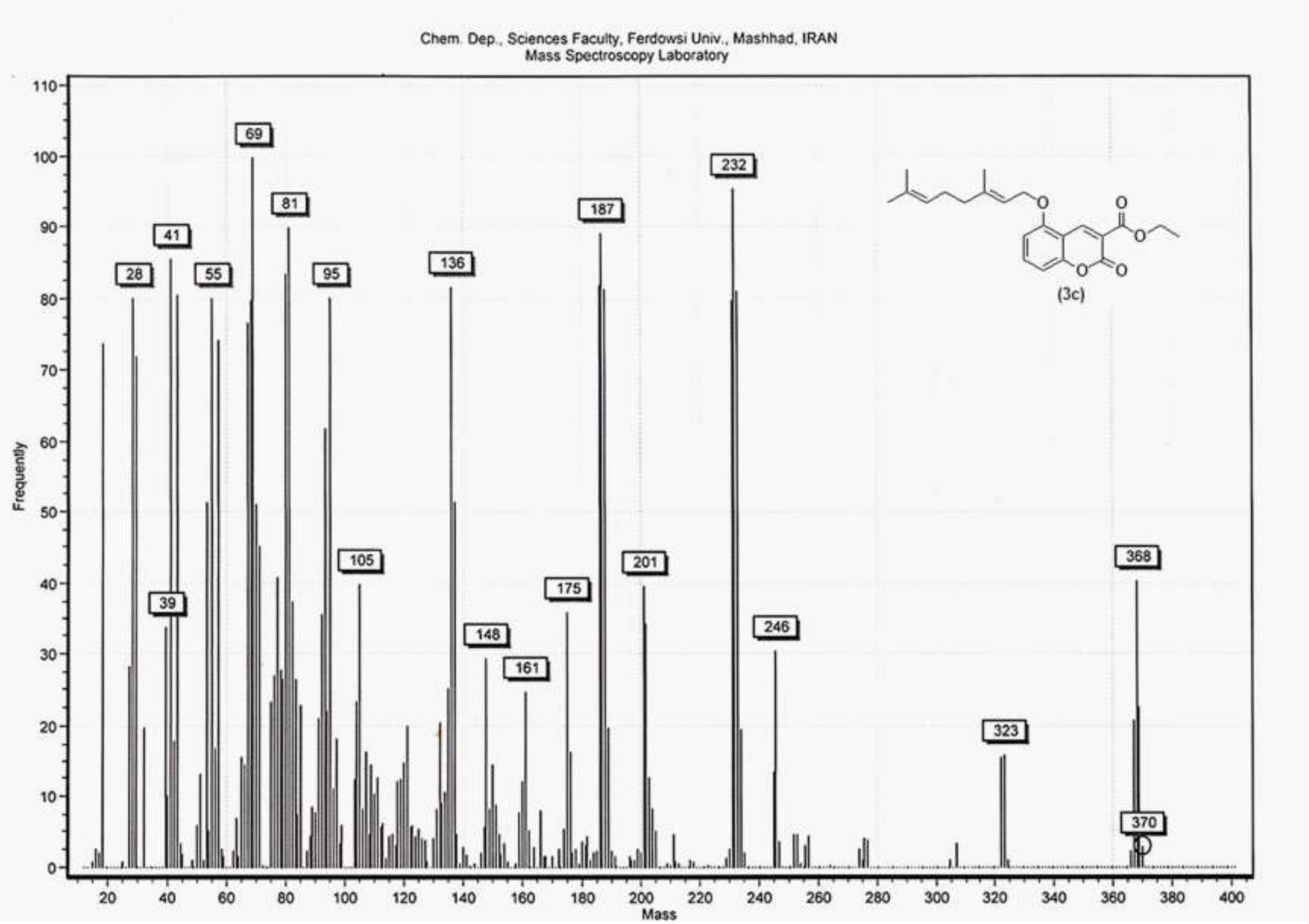

Chem. Dep., Sciences Faculty, Ferdowsi Univ., Mashhad, IRAN
Mass Spectroscopy Laboratory
Frequently
Mass
28
39
41
55
69
81
95
105
136
148
161
175
187
201
232
246
323
368
370
(3c)

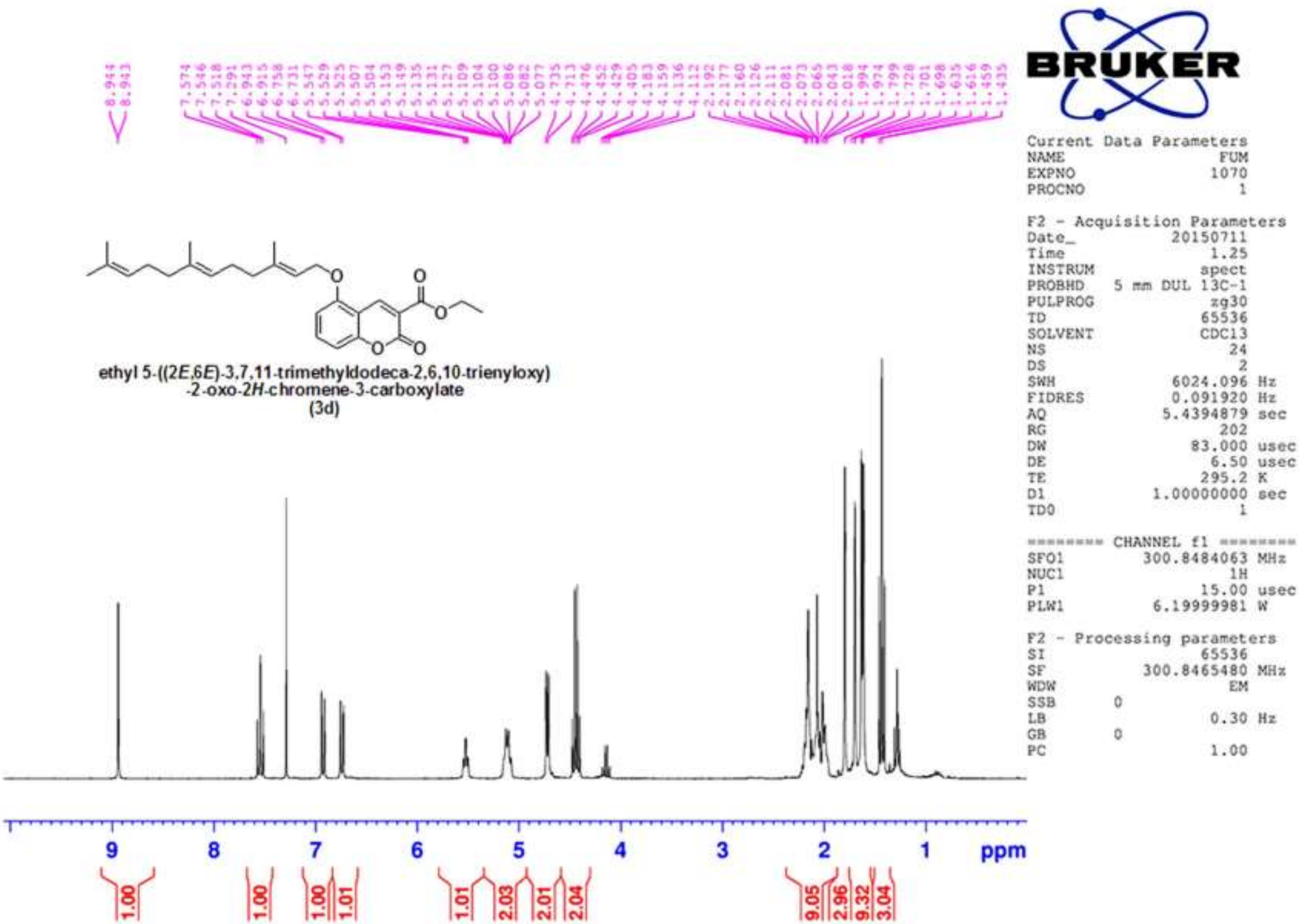
8.944
8.943
7.574
7.546
7.518
7.291
6.943
6.915
6.758
6.731
5.547
5.529
5.525
5.507
5.504
5.153
5.149
5.135
5.131
5.127
5.109
5.104
5.100
5.086
5.082
5.077
4.735
4.713
4.476
4.452
4.429
4.405
4.183
4.159
4.136
4.112
2.192
2.177
2.160
2.126
2.111
2.081
2.073
2.065
2.043
2.018
1.994
1.974
1.799
1.728
1.701
1.698
1.635
1.616
1.459
1.435
ethyl 5-((2*E*,6*E*)-3,7,11-trimethyldodeca-2,6,10-trienyloxy)
-2-oxo-2*H*-chromene-3-carboxylate
(3d)
BRUKER
Current Data Parameters
NAME FUM
EXPNO 1070
PROCNO 1
F2 - Acquisition Parameters
Date_ 20150711
Time 1.25
INSTRUM spect
PROBHD 5 mm DUL 13C-1
PULPROG zg30
TD 65536
SOLVENT CDCl3
NS 24
DS 2
SWH 6024.096 Hz
FIDRES 0.091920 Hz
AQ 5.4394879 sec
RG 202
DW 83.000 usec
DE 6.50 usec
TE 295.2 K
D1 1.00000000 sec
TD0 1
======== CHANNEL f1 ========
SFO1 300.8484063 MHz
NUC1 1H
P1 15.00 usec
PLW1 6.19999981 W
F2 - Processing parameters
SI 65536
SF 300.8465480 MHz
WDW EM
SSB 0
LB 0.30 Hz
GB 0
PC 1.00
9 8 7 6 5 4 3 2 1 ppm
1.00
1.00
1.00
1.01
1.01
2.03
2.01
2.04
9.05
2.96
9.32
3.04

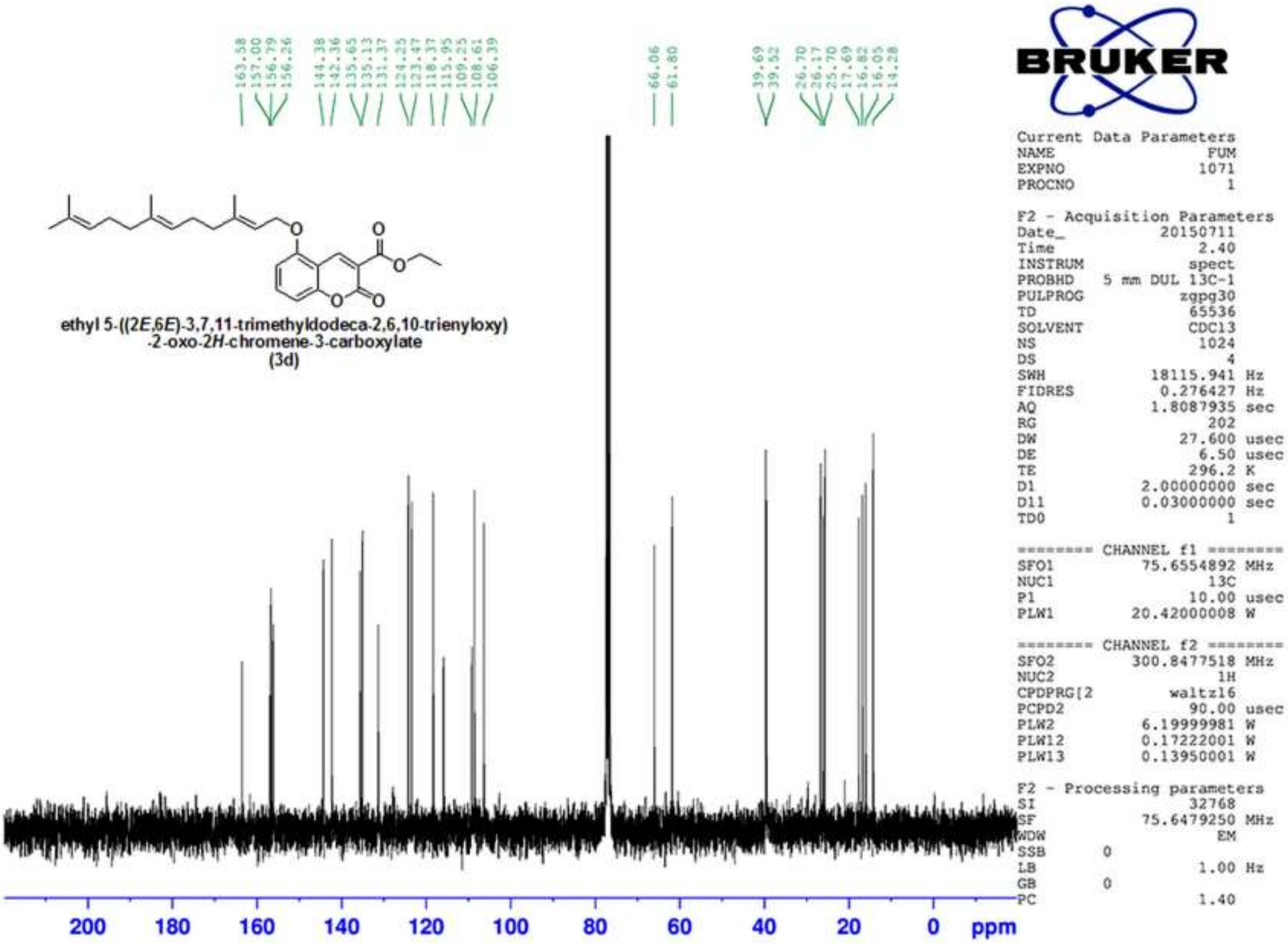
163.58
157.00
156.79
156.26
144.38
142.36
135.65
135.13
131.37
124.25
123.47
118.37
115.95
109.25
108.61
106.39
66.06
61.80
39.69
39.52
26.70
26.17
25.70
17.69
16.82
16.05
14.28
ethyl 5-((2E,6E)-3,7,11-trimethyldodeca-2,6,10-trienyloxy)-2-oxo-2H-chromene-3-carboxylate
(3d)
BRUKER
Current Data Parameters
NAME FUM
EXPNO 1071
PROCNO 1
F2 - Acquisition Parameters
Date_ 20150711
Time 2.40
INSTRUM spect
PROBHD 5 mm DUL 13C-1
PULPROG zgpg30
TD 65536
SOLVENT CDCl3
NS 1024
DS 4
SWH 18115.941 Hz
FIDRES 0.276427 Hz
AQ 1.8087935 sec
RG 202
DW 27.600 usec
DE 6.50 usec
TE 296.2 K
D1 2.00000000 sec
D11 0.03000000 sec
TD0 1
======== CHANNEL f1 ========
SFO1 75.6554892 MHz
NUC1 13C
P1 10.00 usec
PLW1 20.42000008 W
======== CHANNEL f2 ========
SFO2 300.8477518 MHz
NUC2 1H
CPDPRG[2 waltz16
PCPD2 90.00 usec
PLW2 6.19999981 W
PLW12 0.17222001 W
PLW13 0.13950001 W
F2 - Processing parameters
SI 32768
SF 75.6479250 MHz
WDW EM
SSB 0
LB 1.00 Hz
GB 0
PC 1.40
200 180 160 140 120 100 80 60 40 20 0 ppm

Chem. Dep., Sciences Faculty, Ferdowsi Univ., Mashhad, IRAN
Mass Spectroscopy Laboratory

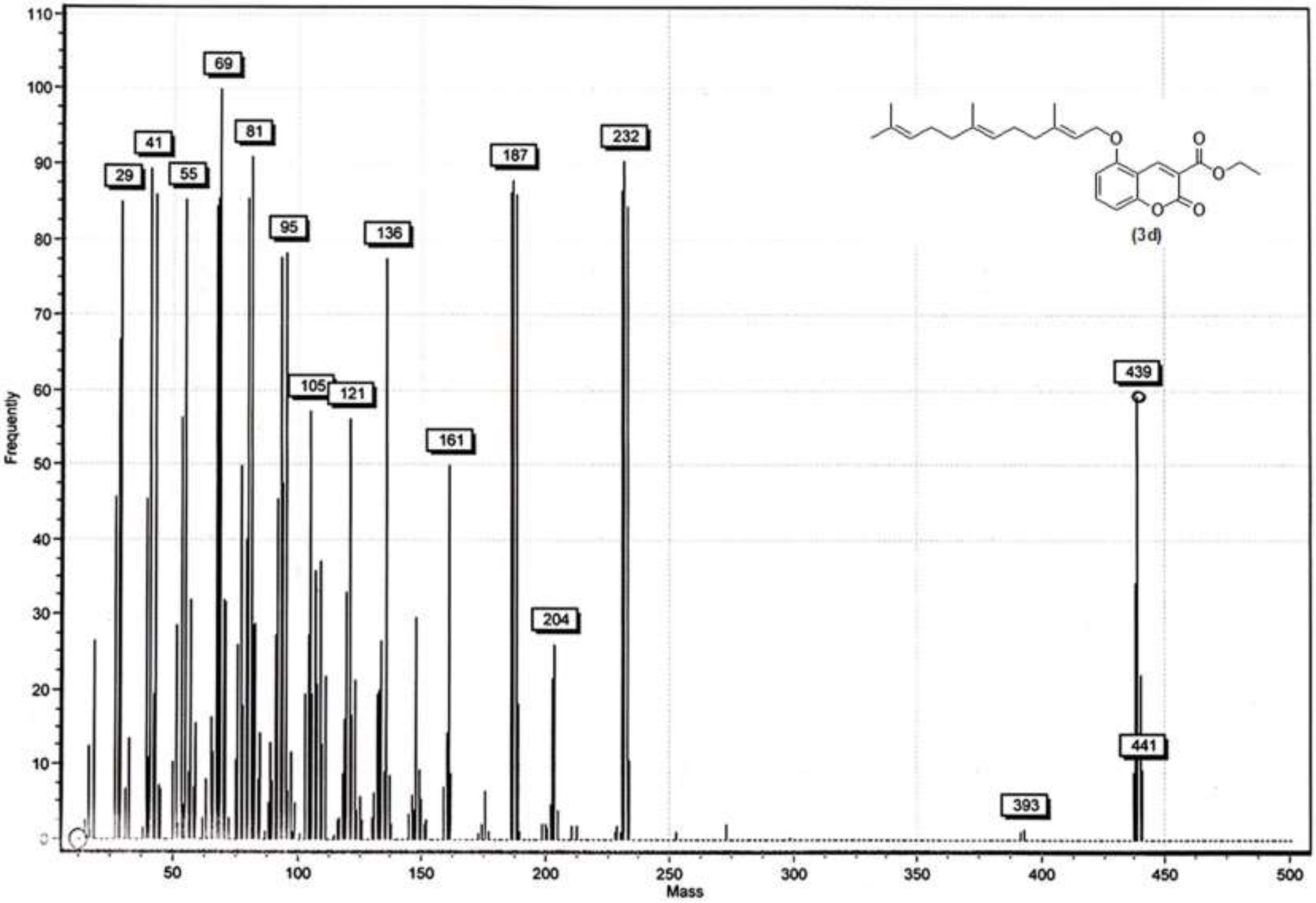

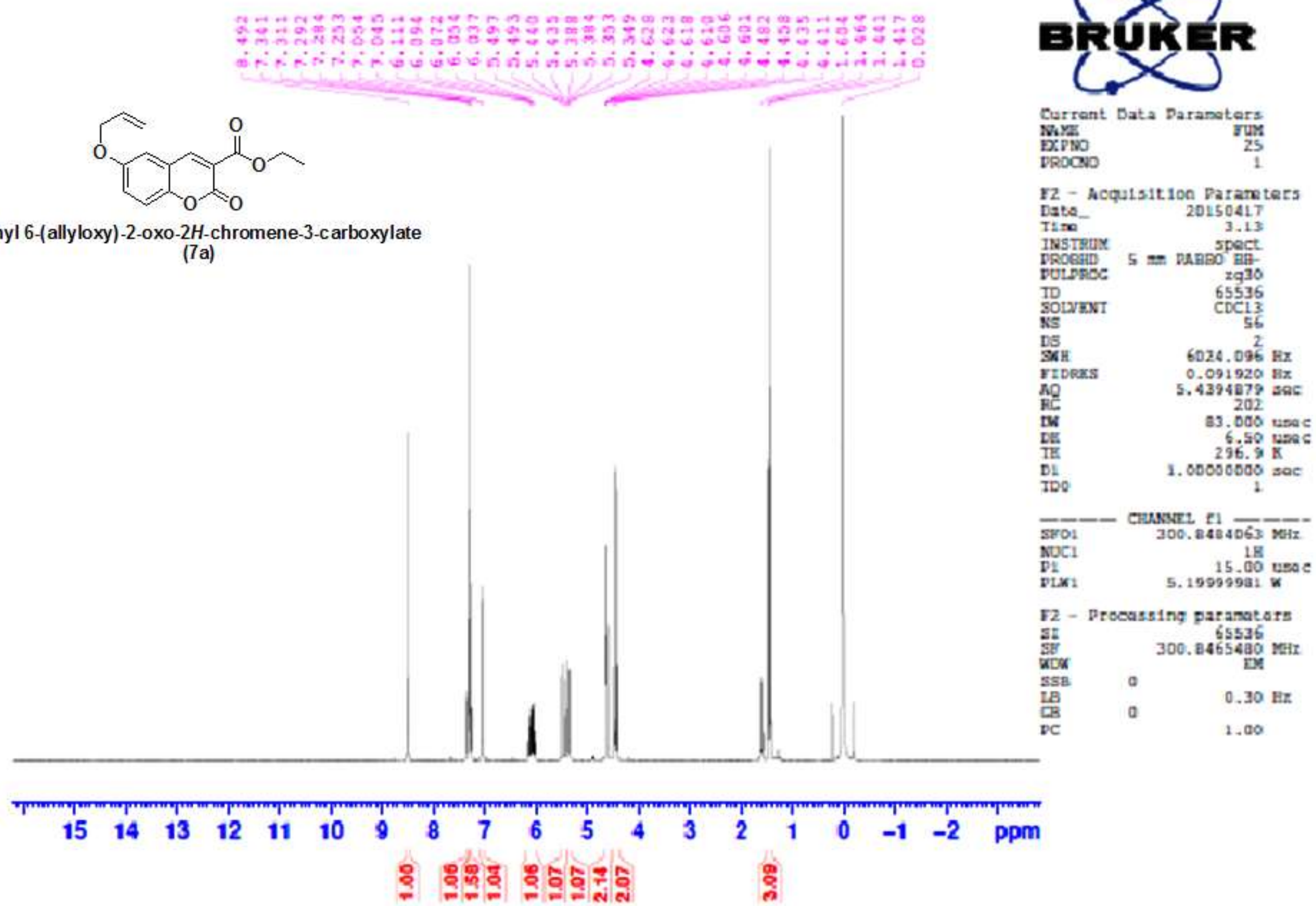

ethyl 6-(allyloxy)-2-oxo-2*H*-chromene-3-carboxylate
(7a)

ethyl 6-(allyloxy)-2-oxo-2*H*-chromene-3-carboxylate
(7a)

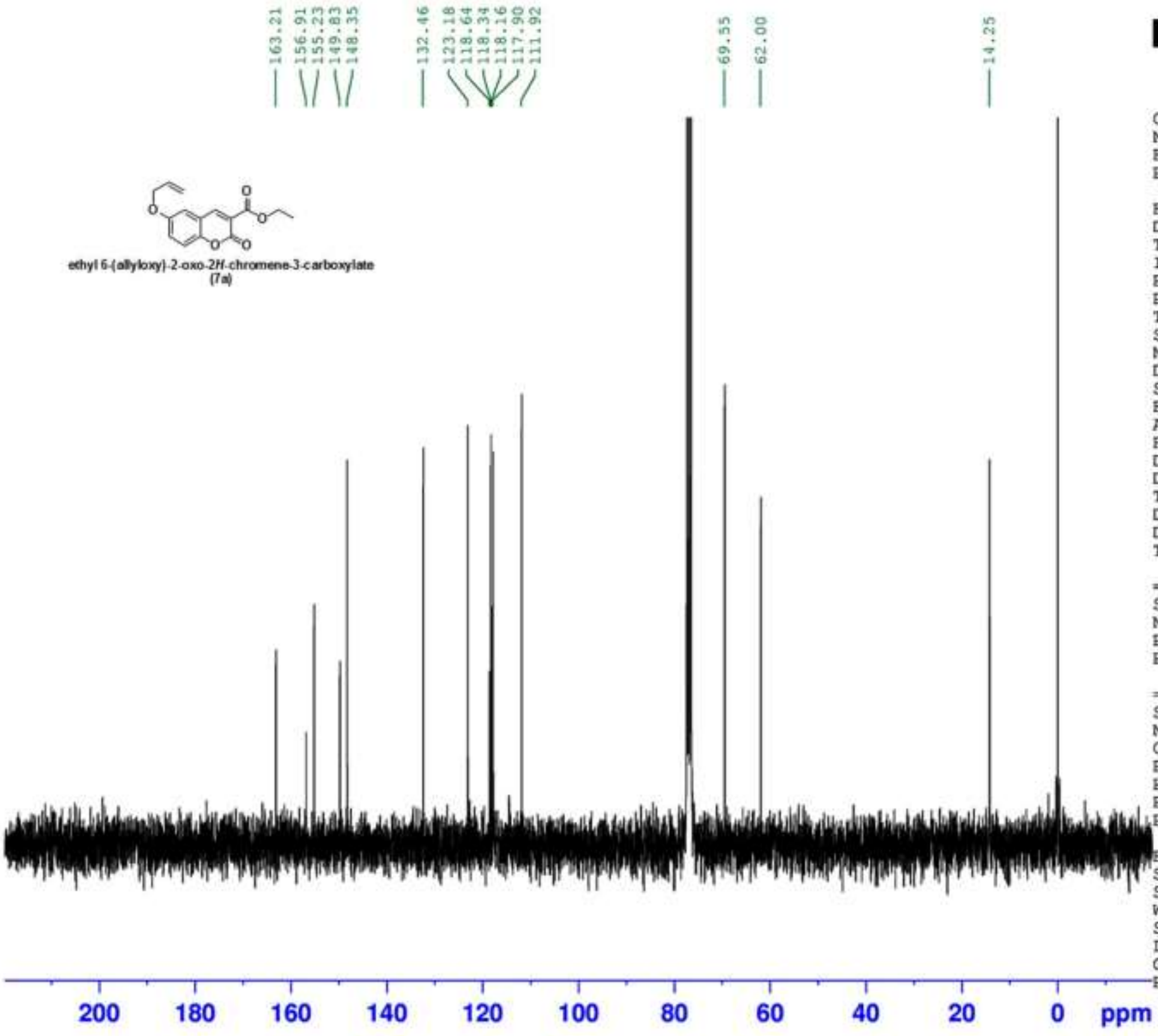

BRUKER

Current Data Parameters
NAME FUM
EXPNO 40
PROCNO 1

F2 - Acquisition Parameters
Date_ 20150514
Time 7.17
INSTRUM spect
PROBHD 5 mm DUL 13C-1
PULPROG zgpg30
TD 65536
SOLVENT CDCl3
NS 1024
DS 4
SWH 18115.941 Hz
FIDRES 0.276427 Hz
AQ 1.8087935 sec
RG 202
DW 27.600 use
DE 6.50 use
TE 297.4 K
D1 2.00000000 sec
D11 0.03000000 sec
TD0 1

======== CHANNEL f1 =======
SFO1 75.6554892 MHz
NUC1 13C
P1 10.00 use
PLW1 20.42000008 W

======== CHANNEL f2 =======
SFO2 300.8477518 MHz
NUC2 1H
CPDPRG[2 waltz16
PCPD2 90.00 use
PLW2 6.19999981 W
PLW12 0.17222001 W
PLW13 0.13950001 W

F2 - Processing parameters
SI 32768
SF 75.6479249 MHz
WDW EM
SSB 0
LB 1.00 Hz
GB 0
PC 1.40

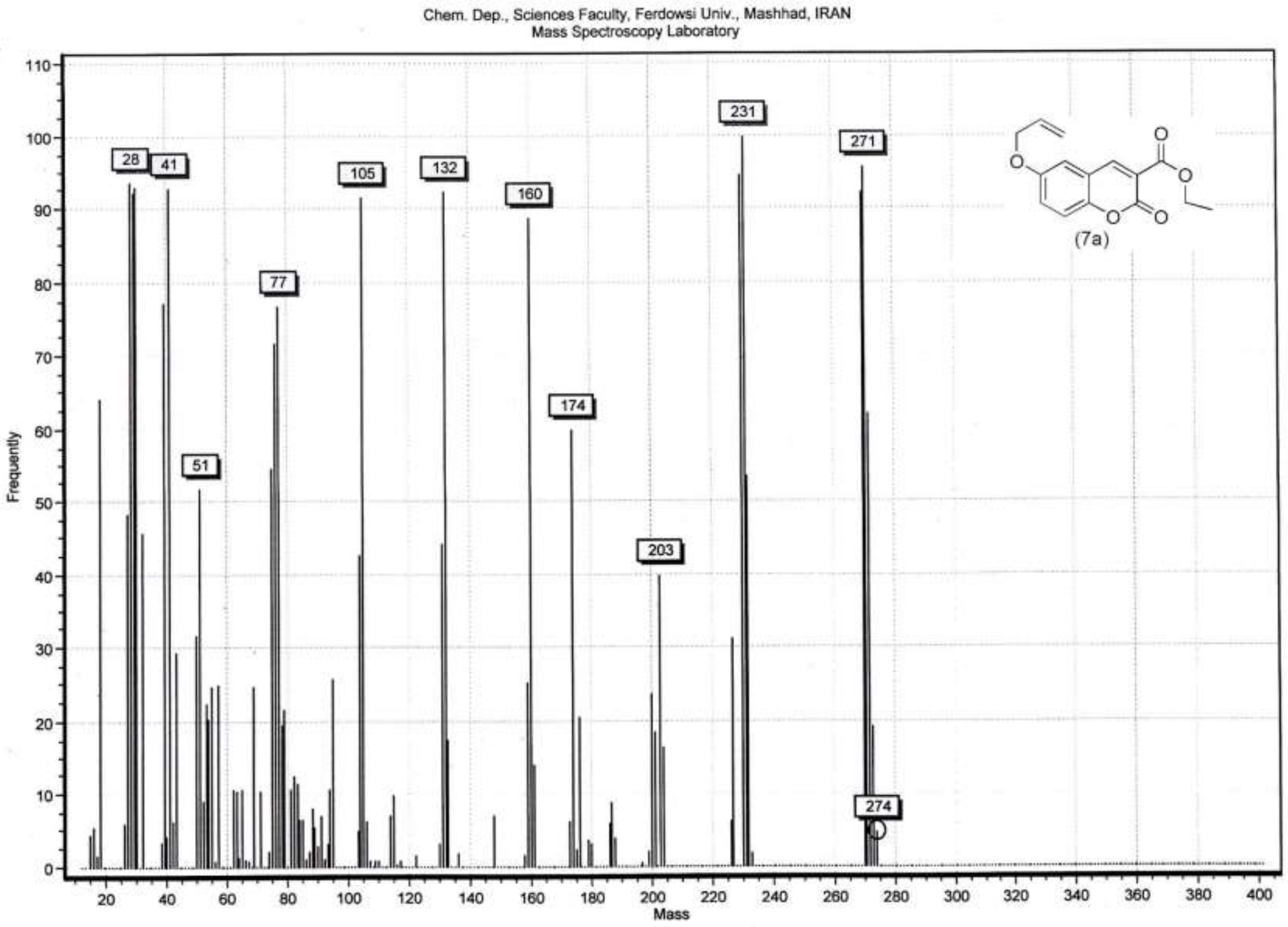

Chem. Dep., Sciences Faculty, Ferdowsi Univ., Mashhad, IRAN
Mass Spectroscopy Laboratory
Frequently
Mass
28
41
51
77
105
132
160
174
203
231
271
274
(7a)

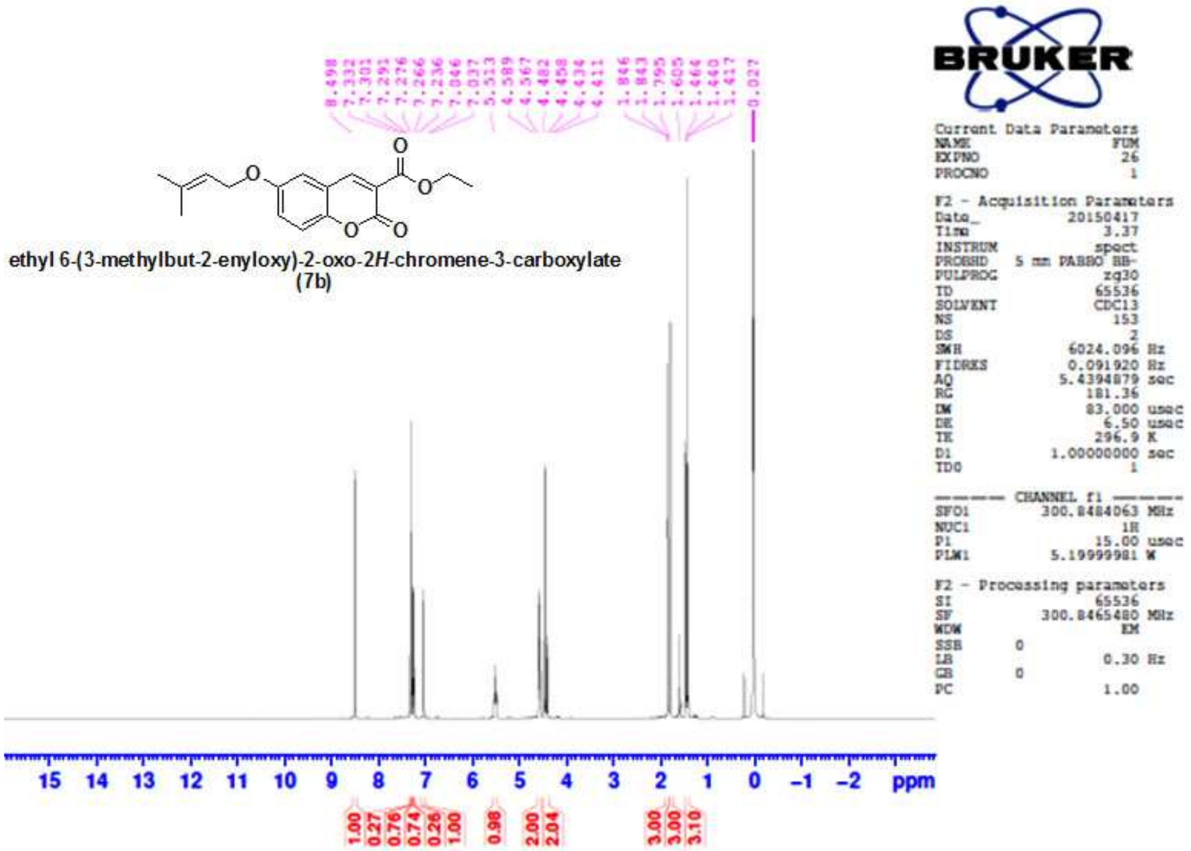

ethyl 6-(3-methylbut-2-enyloxy)-2-oxo-2*H*-chromene-3-carboxylate (7b)

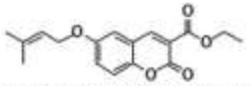

ethyl 6-(3-methylbut-2-enyloxy)-2-oxo-2*H*-chromene-3-carboxylate
(7b)

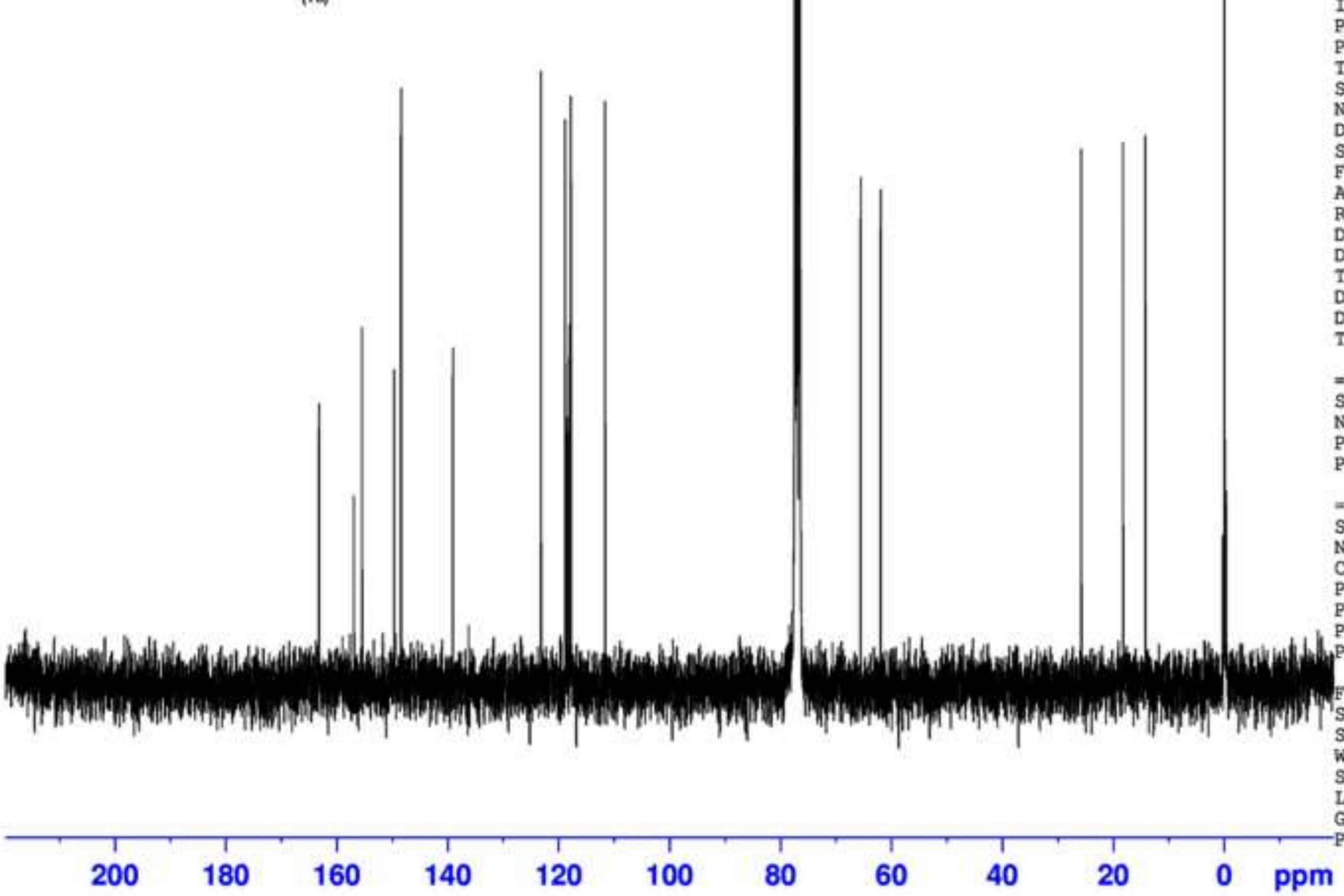

BRUKER

Current Data Parameters
NAME FUM
EXPNO 44
PROCNO 1

F2 - Acquisition Parameters
Date_ 20150514
Time 16.29
INSTRUM spect
PROBHD 5 mm DUL 13C-1
PULPROG zgpg30
TD 65536
SOLVENT CDCl3
NS 5120
DS 4
SWH 18115.941 Hz
FIDRES 0.276427 Hz
AQ 1.8087935 sec
RG 202
DW 27.600 usec
DE 6.50 usec
TE 297.6 K
D1 2.00000000 sec
D11 0.03000000 sec
TD0 1

======== CHANNEL f1 ========
SFO1 75.6554892 MHz
NUC1 13C
P1 10.00 usec
PLW1 20.42000008 W

======== CHANNEL f2 ========
SFO2 300.8477518 MHz
NUC2 1H
CPDPRG[2 waltz16
PCPD2 90.00 usec
PLW2 6.19999981 W
PLW12 0.17222001 W
PLW13 0.13950001 W

F2 - Processing parameters
SI 32768
SF 75.6479249 MHz
WDW EM
SSB 0
LB 1.00 Hz
GB 0
PC 1.40

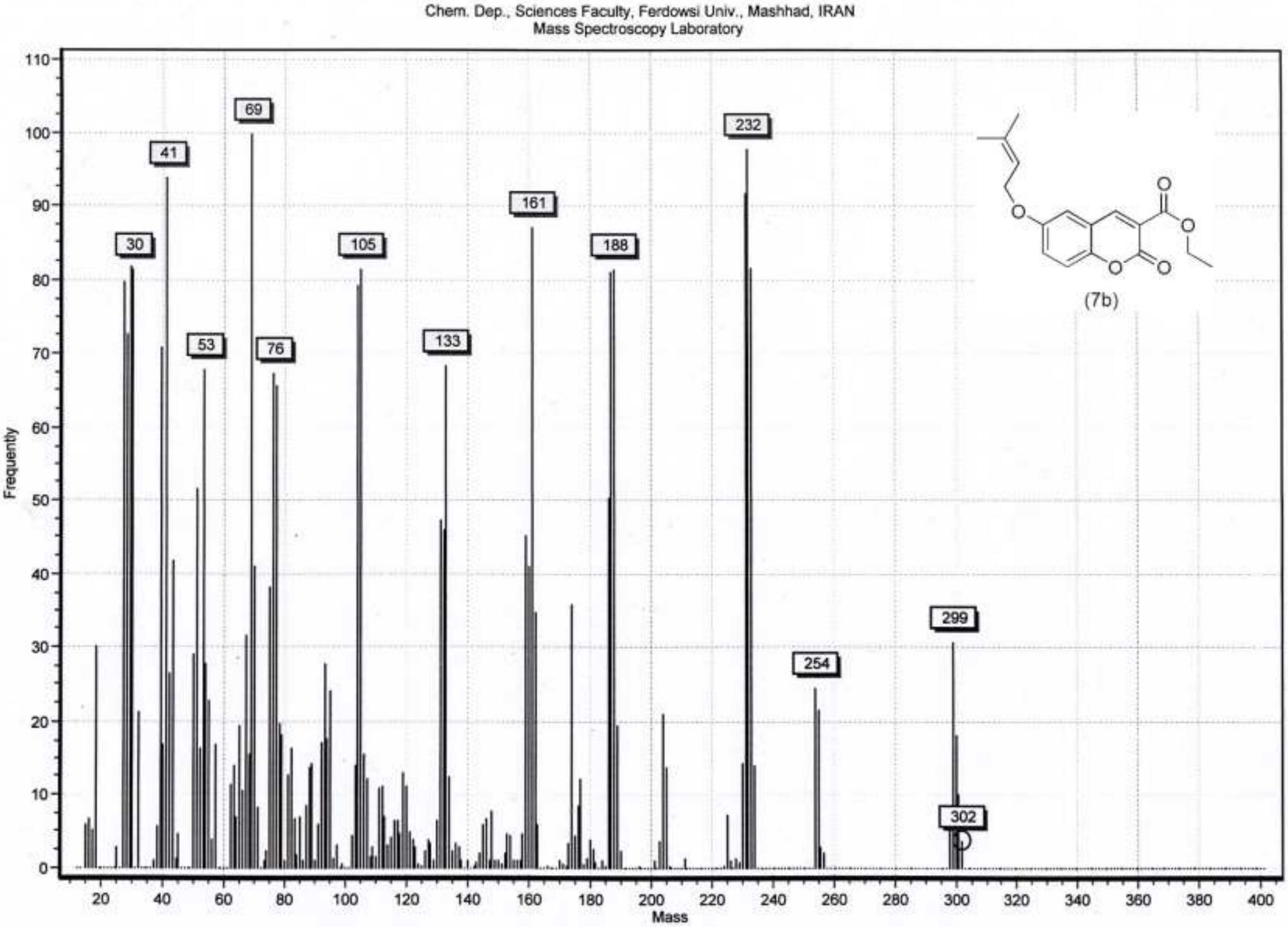

Chem. Dep., Sciences Faculty, Ferdowsi Univ., Mashhad, IRAN
Mass Spectroscopy Laboratory
Frequently
Mass
30
41
53
69
76
105
133
161
188
232
254
299
302
(7b)

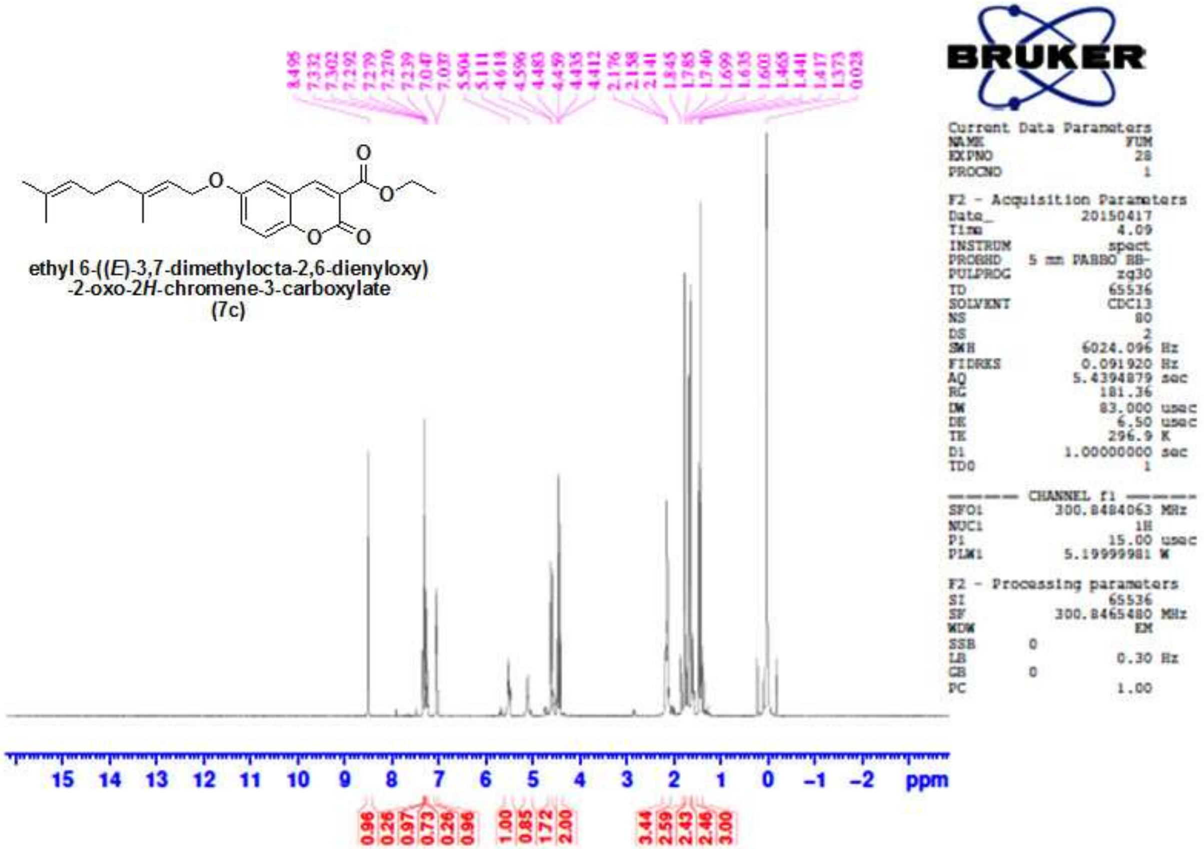
ethyl 6-((E)-3,7-dimethylocta-2,6-dienyloxy)
-2-oxo-2H-chromene-3-carboxylate
(7c)
BRUKER
Current Data Parameters
NAME FUM
EXPNO 28
PROCNO 1
F2 - Acquisition Parameters
Date_ 20150417
Time 4.09
INSTRUM spect
PROBHD 5 mm PABBO BB-
PULPROG zg30
TD 65536
SOLVENT CDCl3
NS 80
DS 2
SWH 6024.096 Hz
FIDRES 0.091920 Hz
AQ 5.4394879 sec
RG 181.36
DW 83.000 usec
DE 6.50 usec
TE 296.9 K
D1 1.00000000 sec
TD0 1
CHANNEL f1
SFO1 300.8484063 MHz
NUC1 1H
P1 15.00 usec
PLW1 5.19999981 W
F2 - Processing parameters
SI 65536
SF 300.8465480 MHz
WDW EM
SSB 0
LB 0.30 Hz
GB 0
PC 1.00
15 14 13 12 11 10 9 8 7 6 5 4 3 2 1 0 -1 -2 ppm

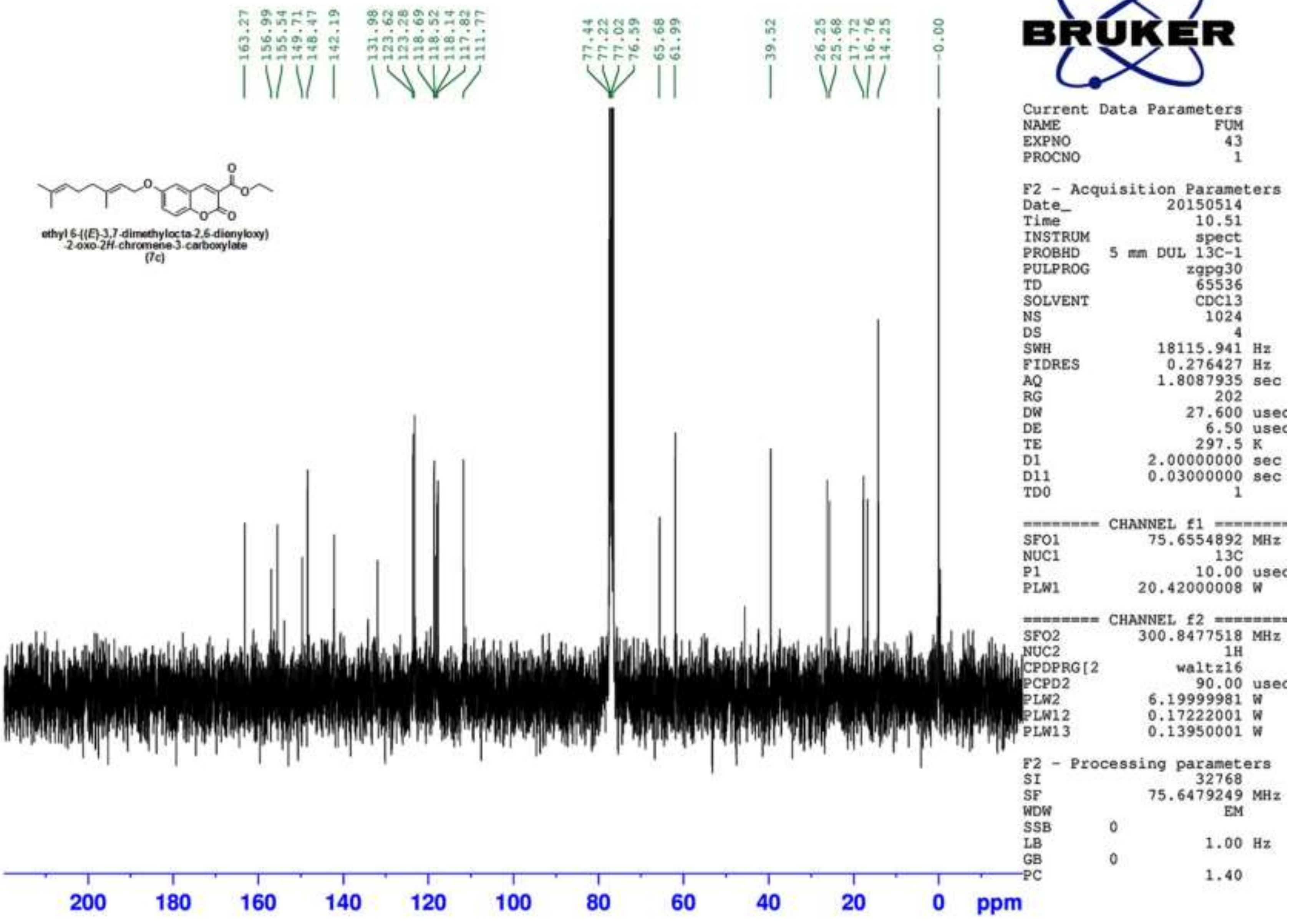

ethyl 6-((E)-3,7-dimethylocta-2,6-dienyloxy)-2-oxo-2H-chromene-3-carboxylate
(7c)
163.27
156.99
155.54
149.71
148.47
142.19
131.98
123.62
123.28
118.69
118.52
118.14
117.82
111.77
77.44
77.22
77.02
76.59
65.68
61.99
39.52
26.25
25.68
17.72
16.76
14.25
-0.00
200
180
160
140
120
100
80
60
40
20
0
ppm
Current Data Parameters
NAME FUM
EXPNO 43
PROCNO 1
F2 - Acquisition Parameters
Date_ 20150514
Time 10.51
INSTRUM spect
PROBHD 5 mm DUL 13C-1
PULPROG zgpg30
TD 65536
SOLVENT CDCl3
NS 1024
DS 4
SWH 18115.941 Hz
FIDRES 0.276427 Hz
AQ 1.8087935 sec
RG 202
DW 27.600 usec
DE 6.50 usec
TE 297.5 K
D1 2.00000000 sec
D11 0.03000000 sec
TD0 1
======== CHANNEL f1 ========
SFO1 75.6554892 MHz
NUC1 13C
P1 10.00 usec
PLW1 20.42000008 W
======== CHANNEL f2 ========
SFO2 300.8477518 MHz
NUC2 1H
CPDPRG[2 waltz16
PCPD2 90.00 usec
PLW2 6.19999981 W
PLW12 0.17222001 W
PLW13 0.13950001 W
F2 - Processing parameters
SI 32768
SF 75.6479249 MHz
WDW EM
SSB 0
LB 1.00 Hz
GB 0
PC 1.40

Chem. Dep., Sciences Faculty, Ferdowsi Univ., Mashhad, IRAN
Mass Spectroscopy Laboratory

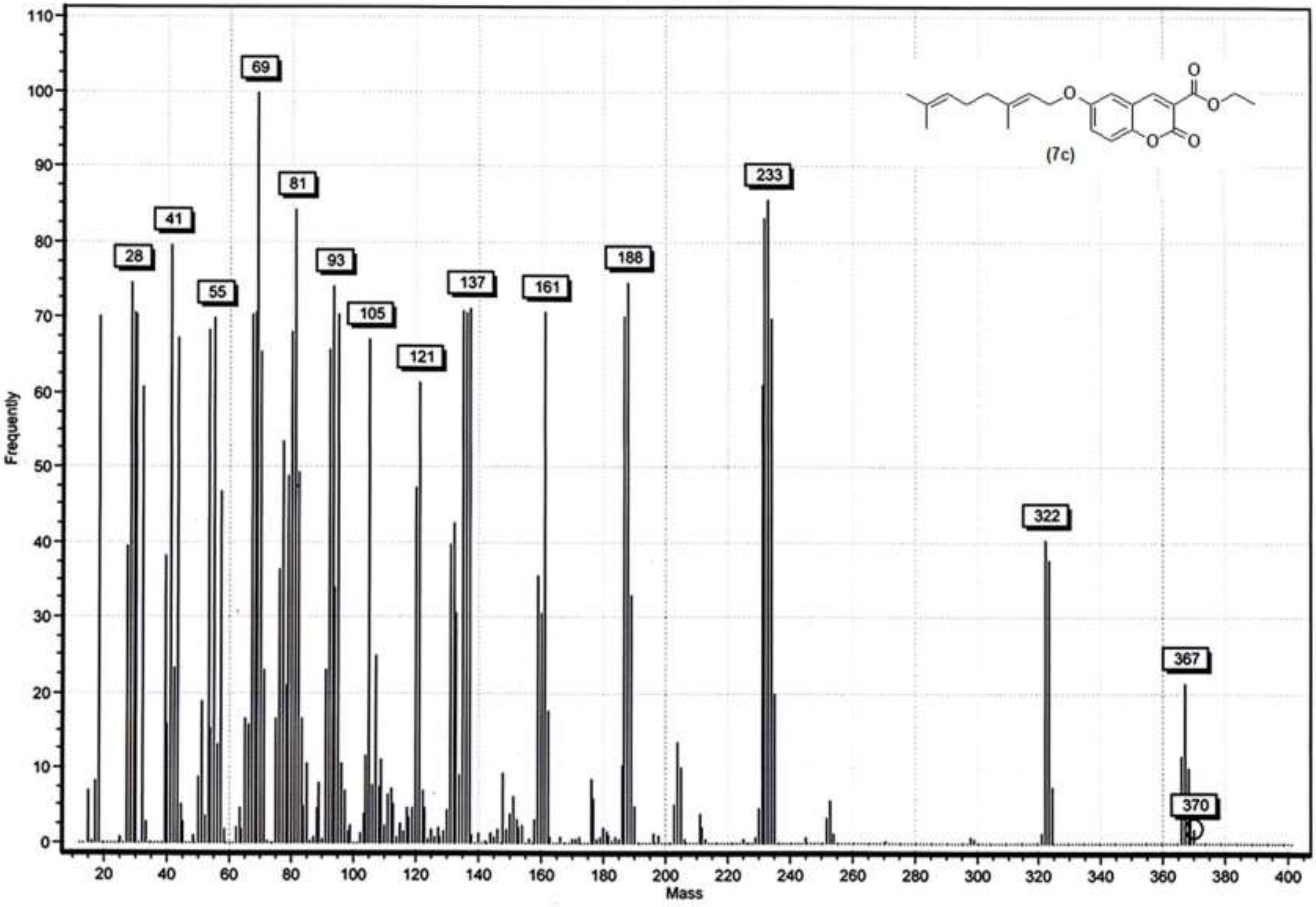

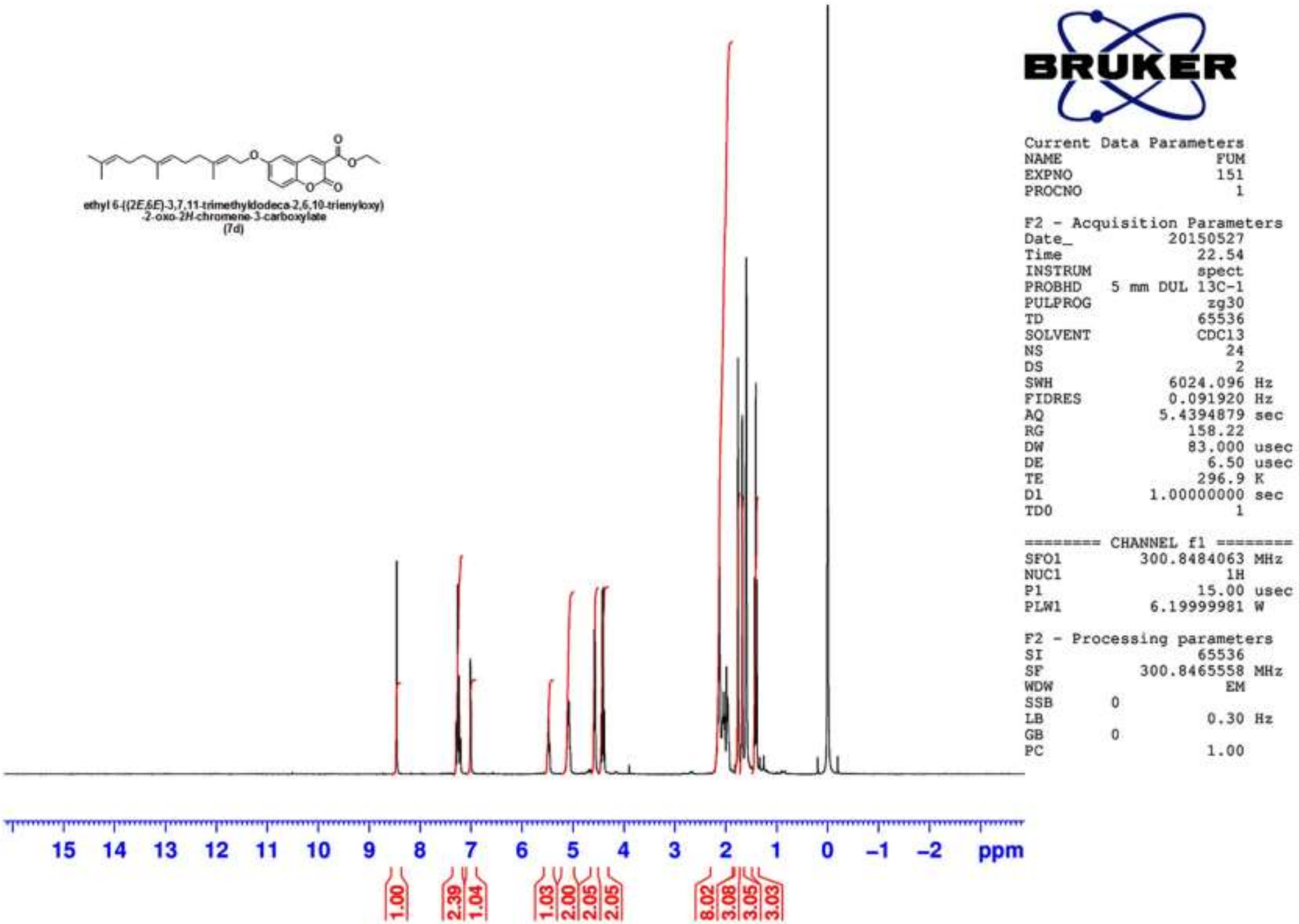
ethyl 6-((2E,6E)-3,7,11-trimethyldodeca-2,6,10-trienyloxy)-2-oxo-2H-chromene-3-carboxylate
(7d)
BRUKER
Current Data Parameters
NAME FUM
EXPNO 151
PROCNO 1
F2 - Acquisition Parameters
Date_ 20150527
Time 22.54
INSTRUM spect
PROBHD 5 mm DUL 13C-1
PULPROG zg30
TD 65536
SOLVENT CDCl3
NS 24
DS 2
SWH 6024.096 Hz
FIDRES 0.091920 Hz
AQ 5.4394879 sec
RG 158.22
DW 83.000 usec
DE 6.50 usec
TE 296.9 K
D1 1.00000000 sec
TD0 1
======== CHANNEL f1 ========
SFO1 300.8484063 MHz
NUC1 1H
P1 15.00 usec
PLW1 6.19999981 W
F2 - Processing parameters
SI 65536
SF 300.8465558 MHz
WDW EM
SSB 0
LB 0.30 Hz
GB 0
PC 1.00
15 14 13 12 11 10 9 8 7 6 5 4 3 2 1 0 −1 −2 ppm
1.00
2.39
1.04
1.03
2.00
2.05
2.05
8.02
3.08
3.05
3.03

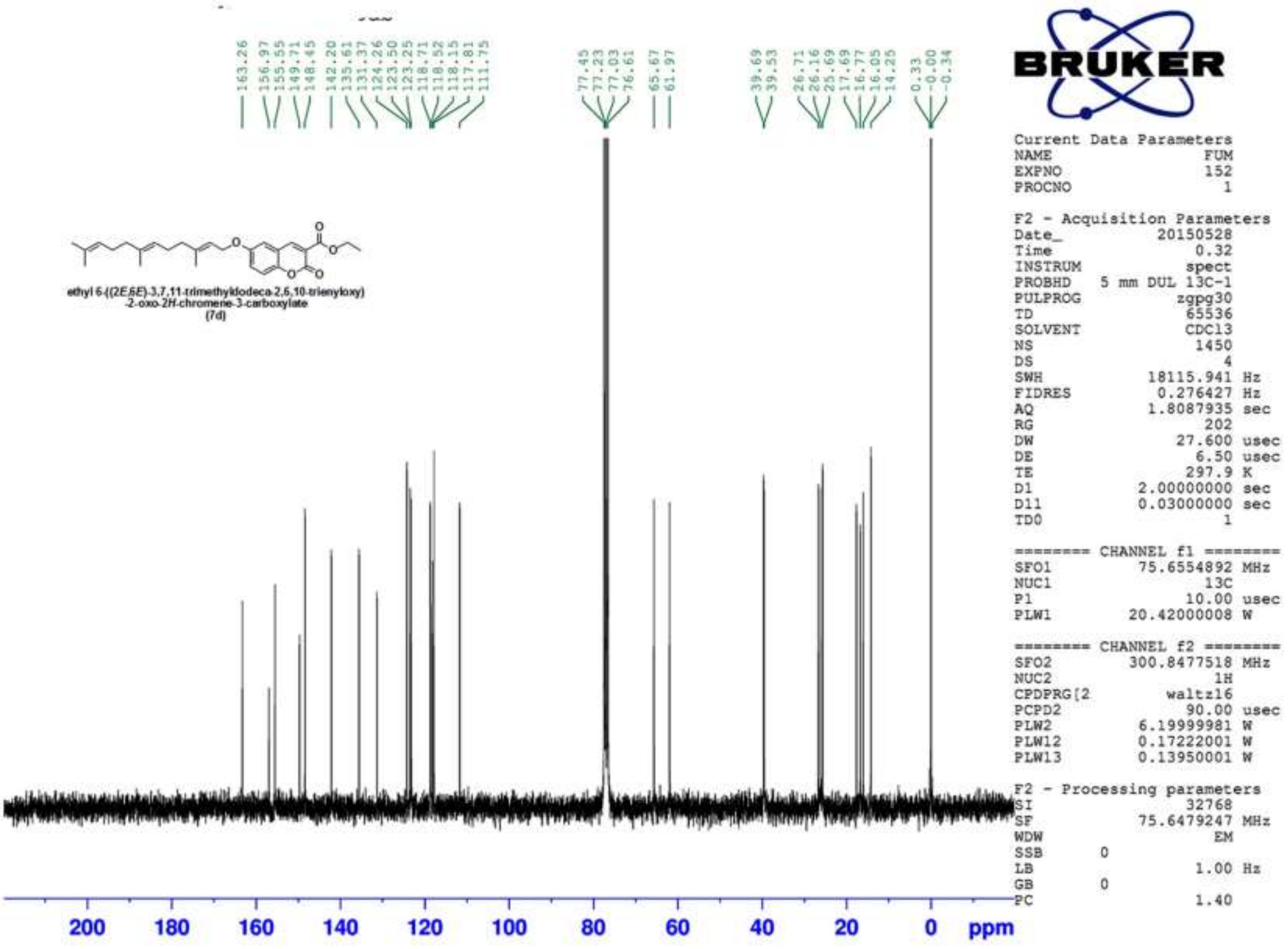
163.26
156.97
155.55
149.71
148.45
142.20
135.61
131.37
124.26
123.50
123.25
118.71
118.52
118.15
117.81
111.75
77.45
77.23
77.03
76.61
65.67
61.97
39.69
39.53
26.71
26.16
25.69
17.69
16.77
16.05
14.25
0.33
-0.00
-0.34
ethyl 6-((2E,6E)-3,7,11-trimethyldodeca-2,6,10-trienyloxy)-2-oxo-2H-chromene-3-carboxylate
(7d)
BRUKER
Current Data Parameters
NAME FUM
EXPNO 152
PROCNO 1
F2 - Acquisition Parameters
Date_ 20150528
Time 0.32
INSTRUM spect
PROBHD 5 mm DUL 13C-1
PULPROG zgpg30
TD 65536
SOLVENT CDCl3
NS 1450
DS 4
SWH 18115.941 Hz
FIDRES 0.276427 Hz
AQ 1.8087935 sec
RG 202
DW 27.600 usec
DE 6.50 usec
TE 297.9 K
D1 2.00000000 sec
D11 0.03000000 sec
TD0 1
======== CHANNEL f1 ========
SFO1 75.6554892 MHz
NUC1 13C
P1 10.00 usec
PLW1 20.42000008 W
======== CHANNEL f2 ========
SFO2 300.8477518 MHz
NUC2 1H
CPDPRG[2 waltz16
PCPD2 90.00 usec
PLW2 6.19999981 W
PLW12 0.17222001 W
PLW13 0.13950001 W
F2 - Processing parameters
SI 32768
SF 75.6479247 MHz
WDW EM
SSB 0
LB 1.00 Hz
GB 0
PC 1.40
200
180
160
140
120
100
80
60
40
20
0
ppm

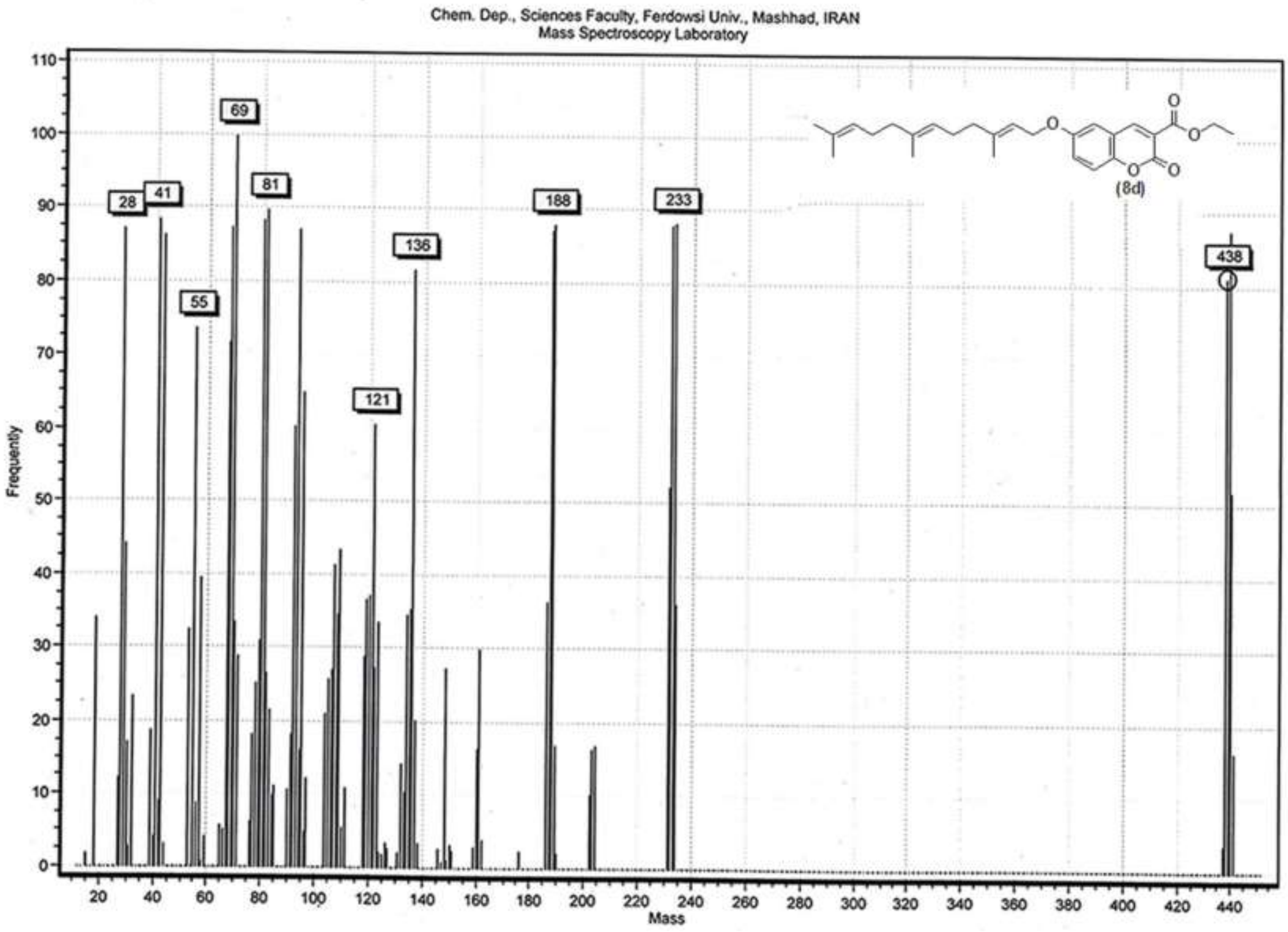

Chem. Dep., Sciences Faculty, Ferdowsi Univ., Mashhad, IRAN
Mass Spectroscopy Laboratory
Frequently
Mass
28
41
55
69
81
121
136
188
233
438
(8d)

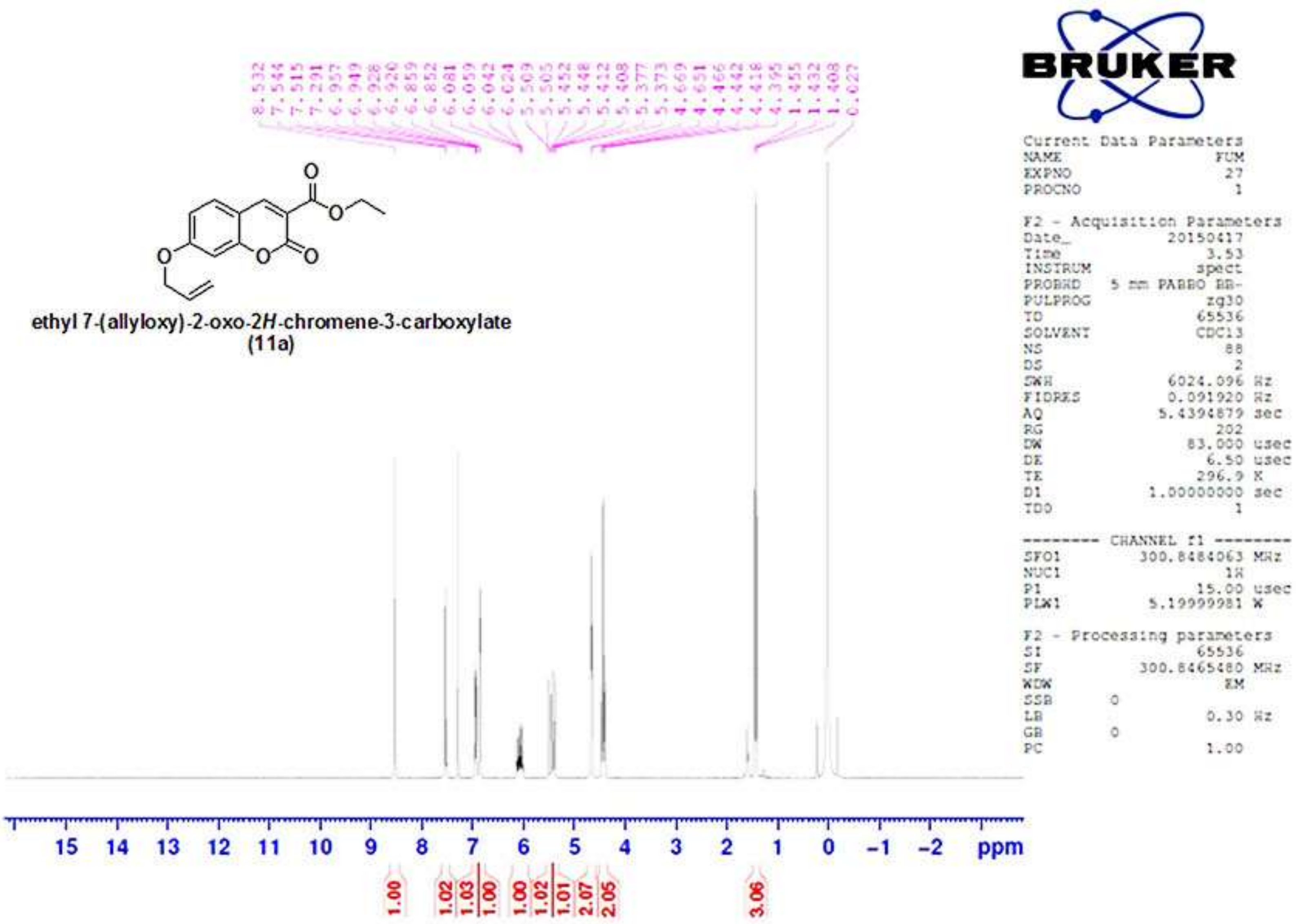

ethyl 7-(allyloxy)-2-oxo-2*H*-chromene-3-carboxylate
(11a)

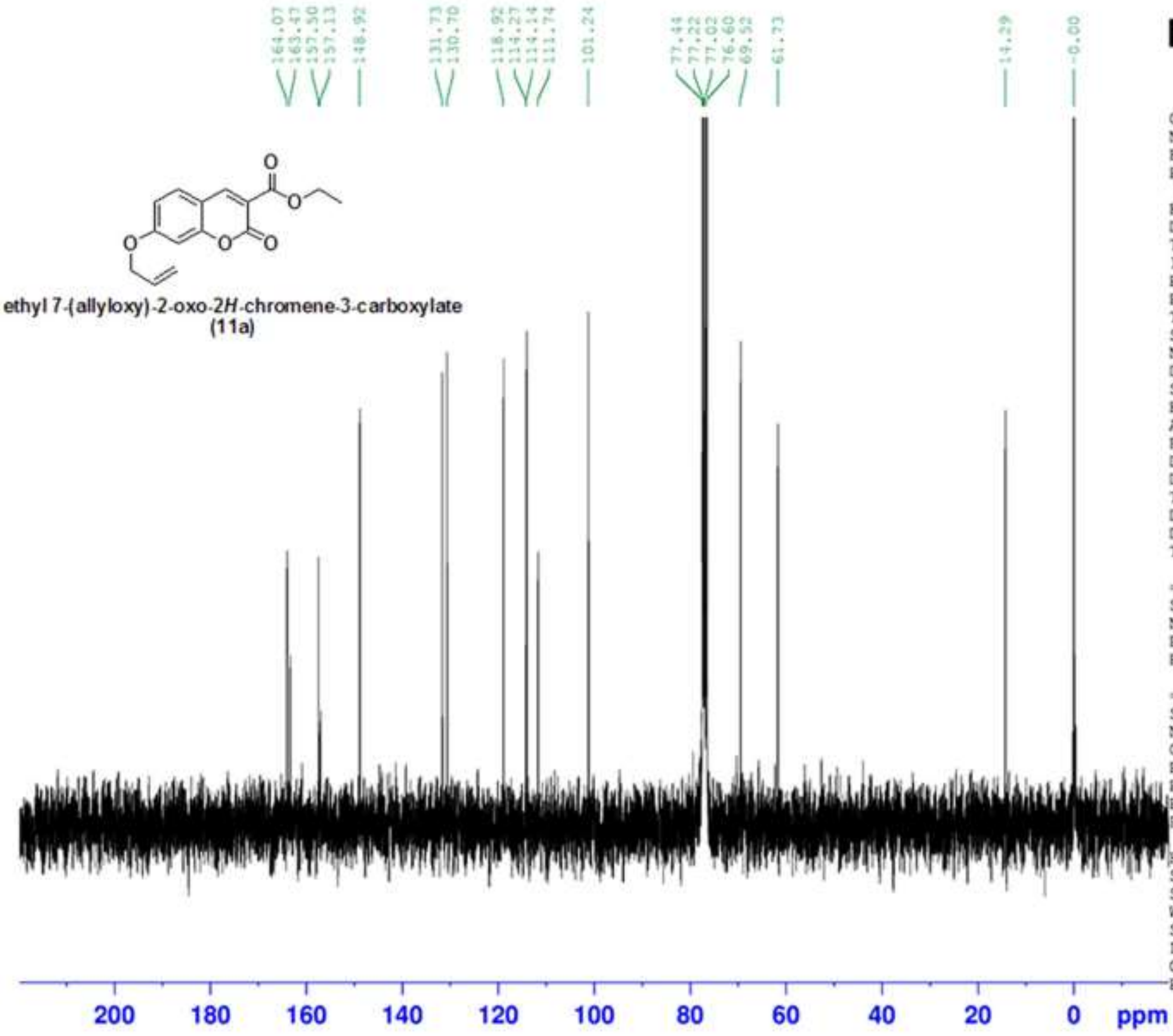

ethyl 7-(allyloxy)-2-oxo-2*H*-chromene-3-carboxylate
(11a)

BRUKER

Current Data Parameters
NAME FUM
EXPNO 41
PROCNO 1

F2 - Acquisition Parameters
Date_ 20150514
Time 8.28
INSTRUM spect
PROBHD 5 mm DUL 13C-1
PULPROG zgpg30
TD 65536
SOLVENT CDCl3
NS 1024
DS 4
SWH 18115.941 Hz
FIDRES 0.276427 Hz
AQ 1.8087935 sec
RG 202
DW 27.600 usec
DE 6.50 usec
TE 297.4 K
D1 2.00000000 sec
D11 0.03000000 sec
TD0 1

======== CHANNEL f1 ========
SFO1 75.6554892 MHz
NUC1 13C
P1 10.00 usec
PLW1 20.42000008 W

======== CHANNEL f2 ========
SFO2 300.8477518 MHz
NUC2 1H
CPDPRG[2 waltz16
PCPD2 90.00 usec
PLW2 6.19999981 W
PLW12 0.17222001 W
PLW13 0.13950001 W

F2 - Processing parameters
SI 32768
SF 75.6479247 MHz
WDW EM
SSB 0
LB 1.00 Hz
GB 0
PC 1.40

Chem. Dep., Sciences Faculty, Ferdowsi Univ., Mashhad, IRAN
Mass Spectroscopy Laboratory

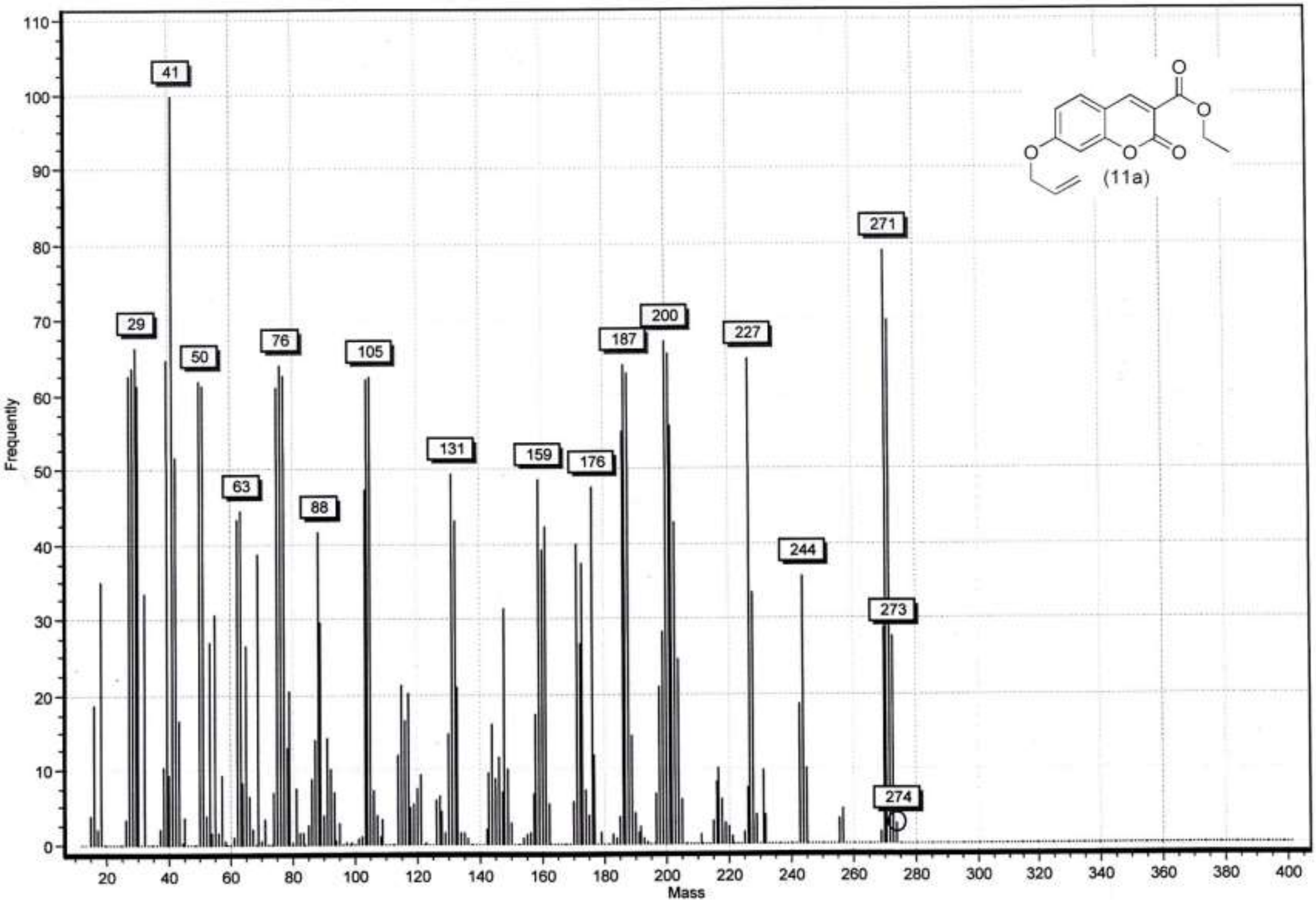

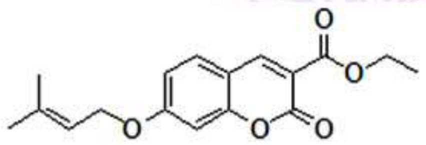

ethyl 7-(3-methylbut-2-enyloxy)-2-oxo-2*H*-chromene-3-carboxylate
(11b)

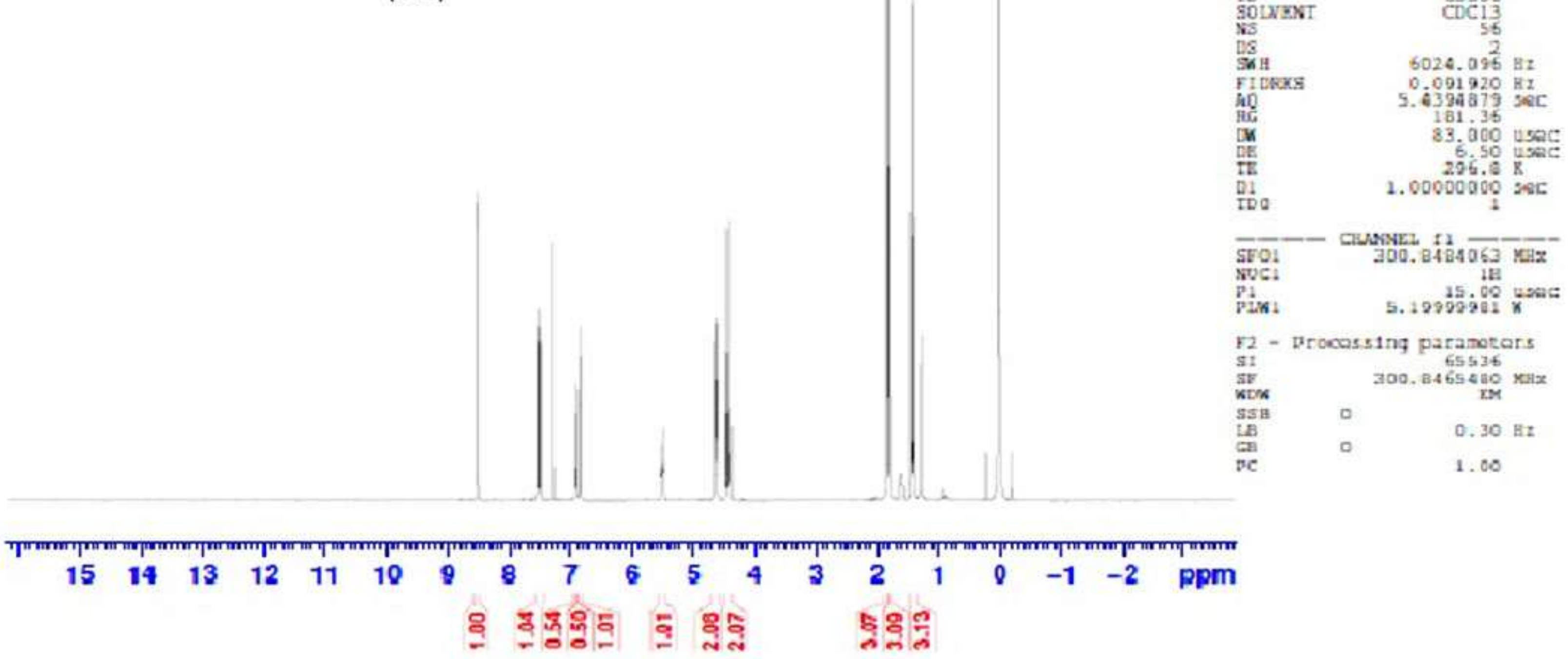

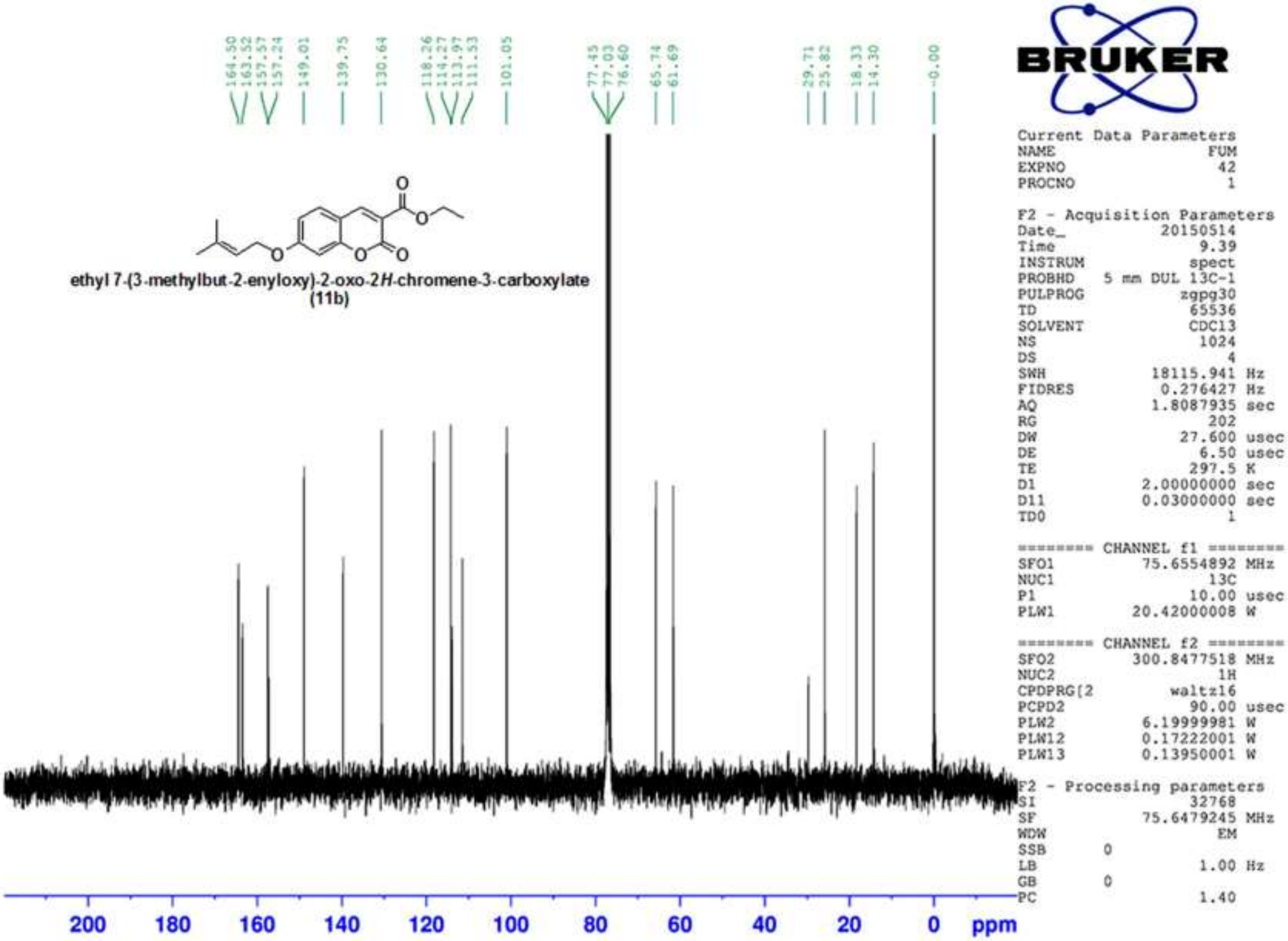
164.50
163.52
157.57
157.24
149.01
139.75
130.64
118.26
114.27
113.97
111.53
101.05
77.45
77.03
76.60
65.74
61.69
29.71
25.82
18.33
14.30
-0.00
ethyl 7-(3-methylbut-2-enyloxy)-2-oxo-2H-chromene-3-carboxylate
(11b)
BRUKER
Current Data Parameters
NAME FUM
EXPNO 42
PROCNO 1
F2 - Acquisition Parameters
Date_ 20150514
Time 9.39
INSTRUM spect
PROBHD 5 mm DUL 13C-1
PULPROG zgpg30
TD 65536
SOLVENT CDCl3
NS 1024
DS 4
SWH 18115.941 Hz
FIDRES 0.276427 Hz
AQ 1.8087935 sec
RG 202
DW 27.600 usec
DE 6.50 usec
TE 297.5 K
D1 2.00000000 sec
D11 0.03000000 sec
TD0 1
======== CHANNEL f1 ========
SFO1 75.6554892 MHz
NUC1 13C
P1 10.00 usec
PLW1 20.42000008 W
======== CHANNEL f2 ========
SFO2 300.8477518 MHz
NUC2 1H
CPDPRG[2 waltz16
PCPD2 90.00 usec
PLW2 6.19999981 W
PLW12 0.17222001 W
PLW13 0.13950001 W
F2 - Processing parameters
SI 32768
SF 75.6479245 MHz
WDW EM
SSB 0
LB 1.00 Hz
GB 0
PC 1.40
200
180
160
140
120
100
80
60
40
20
0
ppm

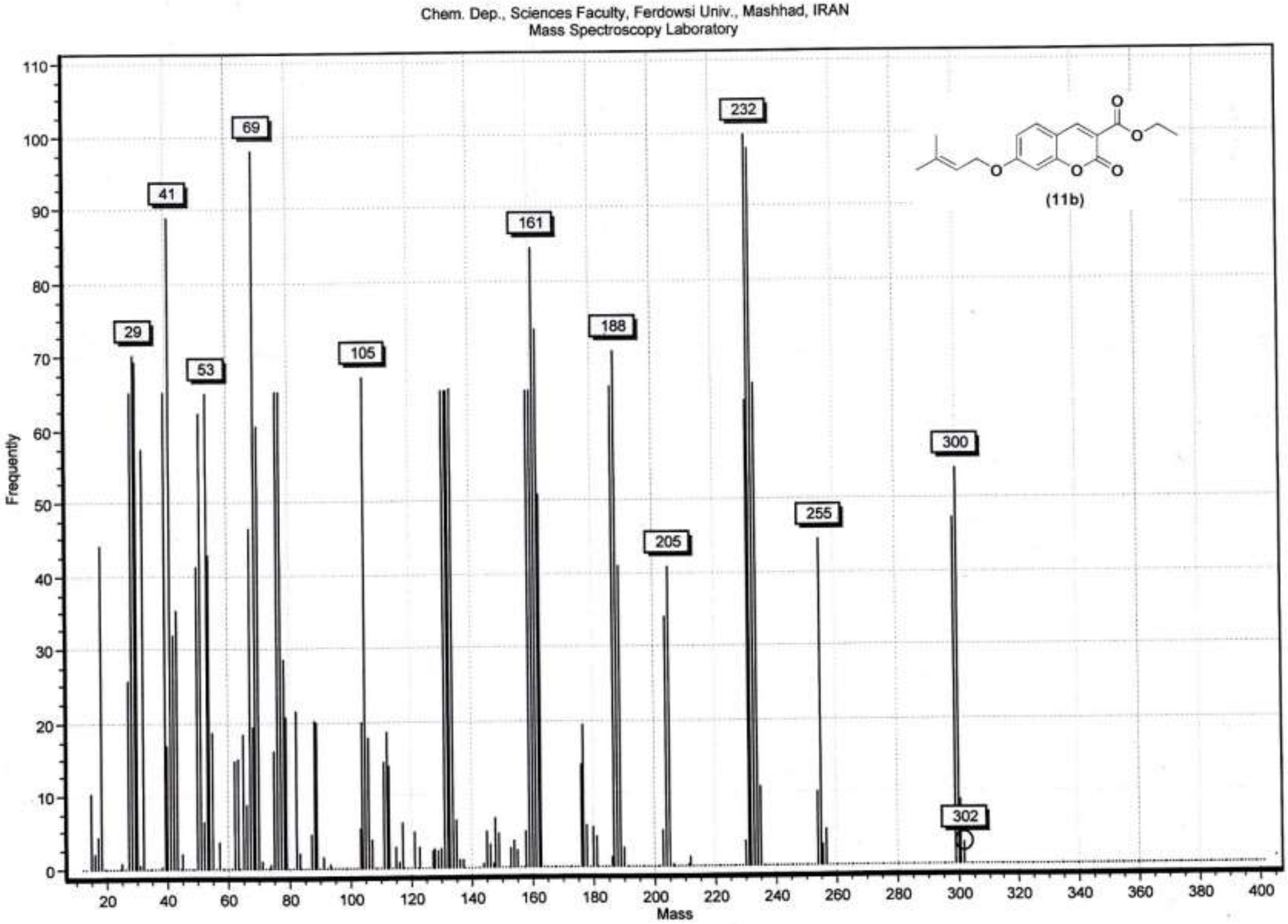
Chem. Dep., Sciences Faculty, Ferdowsi Univ., Mashhad, IRAN
Mass Spectroscopy Laboratory
Frequently
Mass
29
41
53
69
105
161
188
205
232
255
300
302
(11b)

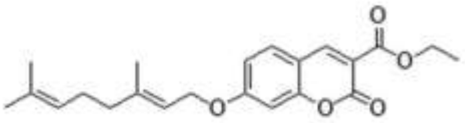

ethyl 7-((*E*)-3,7-dimethylocta-2,6-dienyloxy)
-2-oxo-2*H*-chromene-3-carboxylate
(11c)

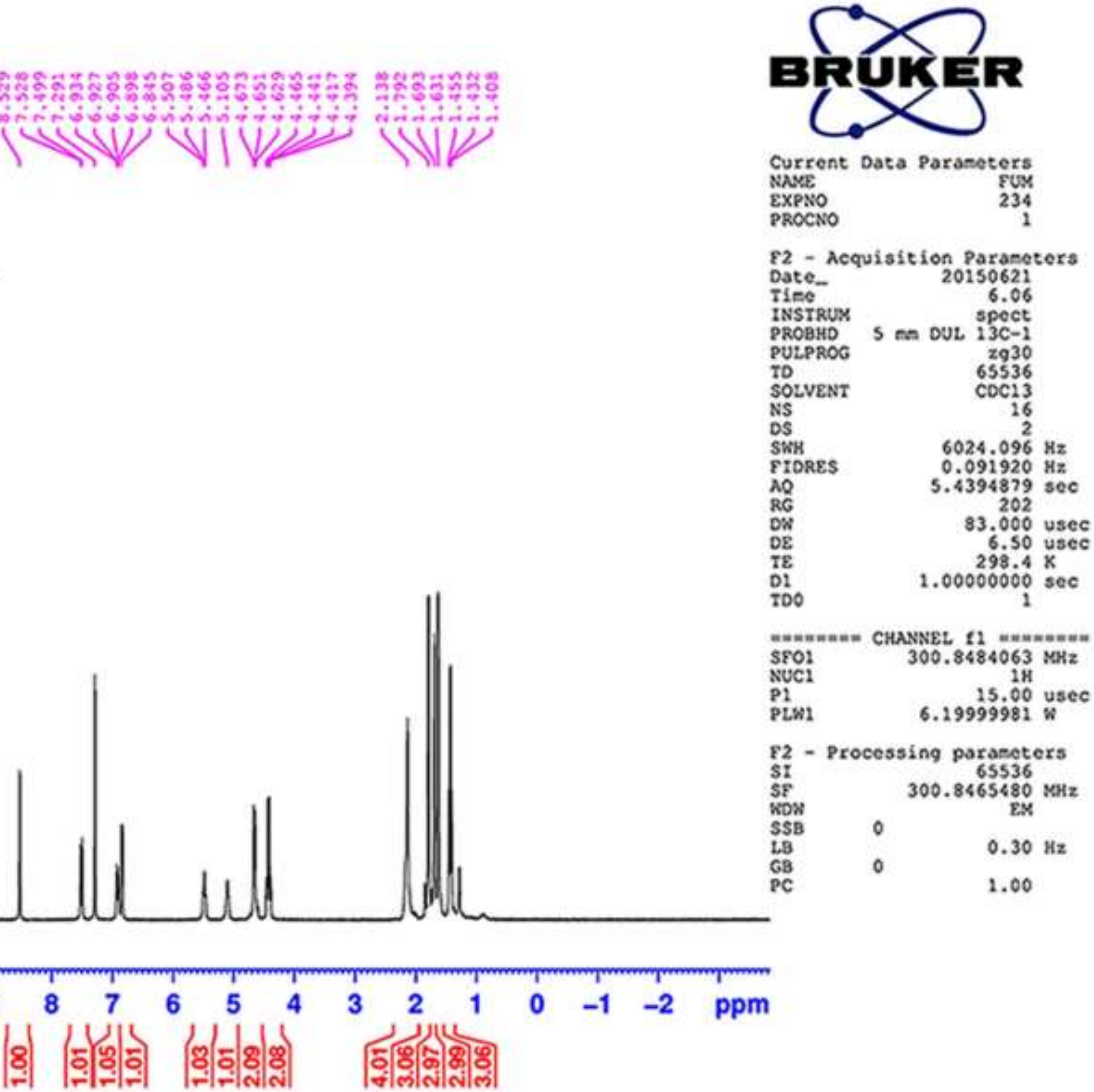

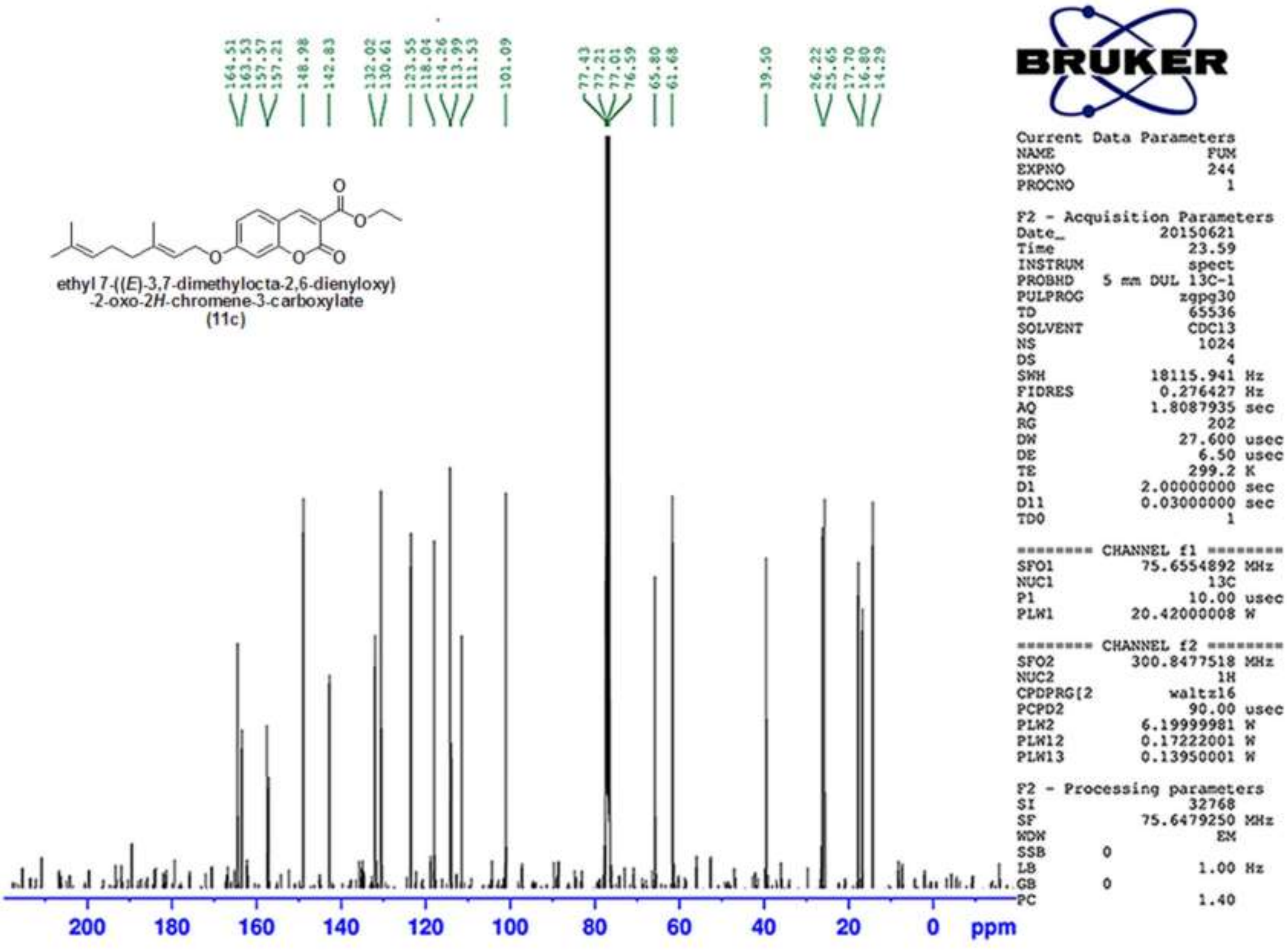
164.51
163.53
157.57
157.21
148.98
142.83
132.02
130.61
123.55
118.04
114.26
113.99
111.53
101.09
77.43
77.21
77.01
76.59
65.80
61.68
39.50
26.22
25.65
17.70
16.80
14.29
ethyl 7-((*E*)-3,7-dimethylocta-2,6-dienyloxy)
-2-oxo-2*H*-chromene-3-carboxylate
(11c)
200
180
160
140
120
100
80
60
40
20
0
ppm
BRUKER
Current Data Parameters
NAME FUM
EXPNO 244
PROCNO 1
F2 - Acquisition Parameters
Date_ 20150621
Time 23.59
INSTRUM spect
PROBHD 5 mm DUL 13C-1
PULPROG zgpg30
TD 65536
SOLVENT CDCl3
NS 1024
DS 4
SWH 18115.941 Hz
FIDRES 0.276427 Hz
AQ 1.8087935 sec
RG 202
DW 27.600 usec
DE 6.50 usec
TE 299.2 K
D1 2.00000000 sec
D11 0.03000000 sec
TD0 1
======== CHANNEL f1 ========
SFO1 75.6554892 MHz
NUC1 13C
P1 10.00 usec
PLW1 20.42000008 W
======== CHANNEL f2 ========
SFO2 300.8477518 MHz
NUC2 1H
CPDPRG[2 waltz16
PCPD2 90.00 usec
PLW2 6.19999981 W
PLW12 0.17222001 W
PLW13 0.13950001 W
F2 - Processing parameters
SI 32768
SF 75.6479250 MHz
WDW EM
SSB 0
LB 1.00 Hz
GB 0
PC 1.40

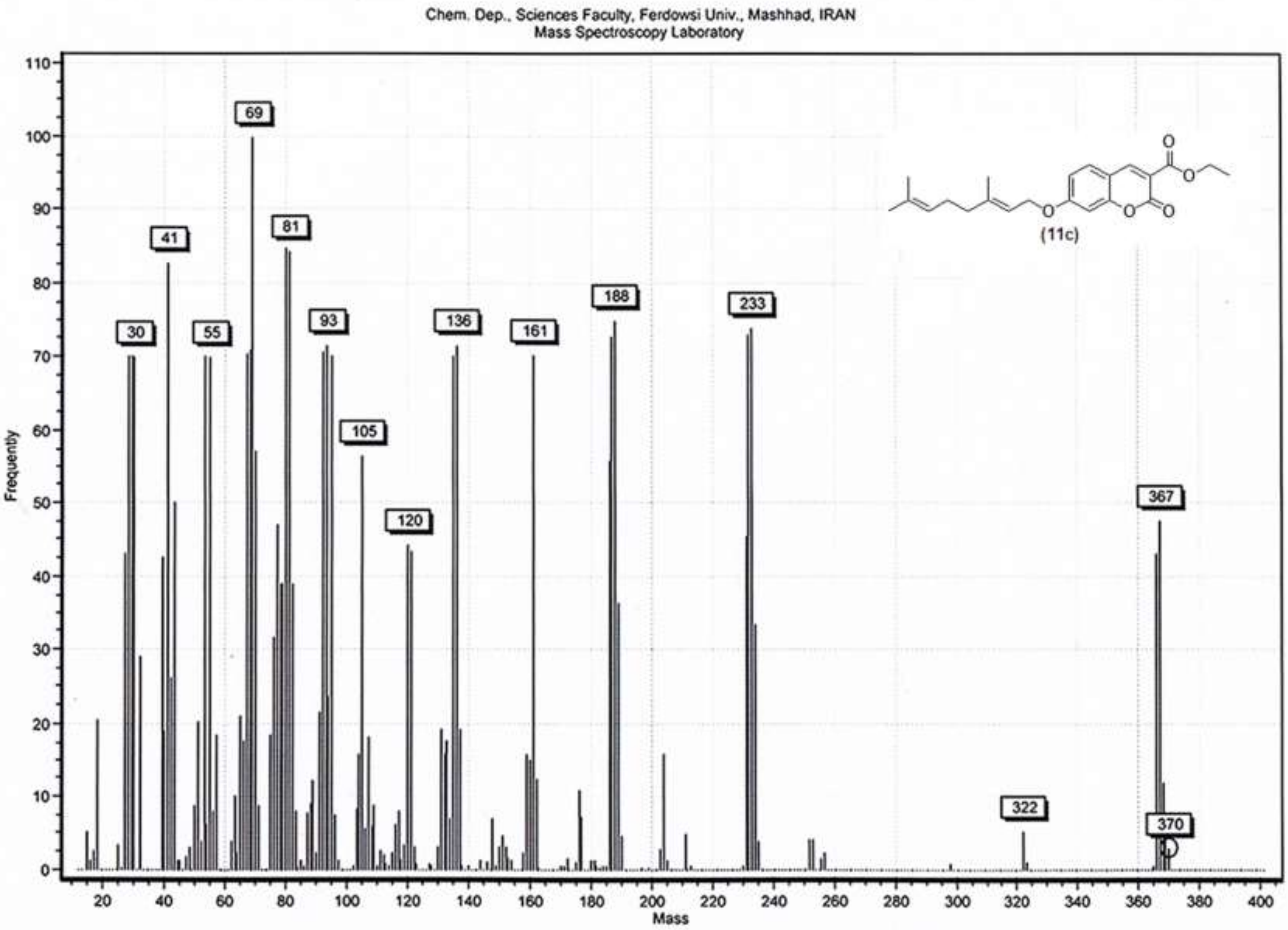
Chem. Dep., Sciences Faculty, Ferdowsi Univ., Mashhad, IRAN
Mass Spectroscopy Laboratory
Frequently
Mass
(11c)
30
41
55
69
81
93
105
120
136
161
188
233
322
367
370

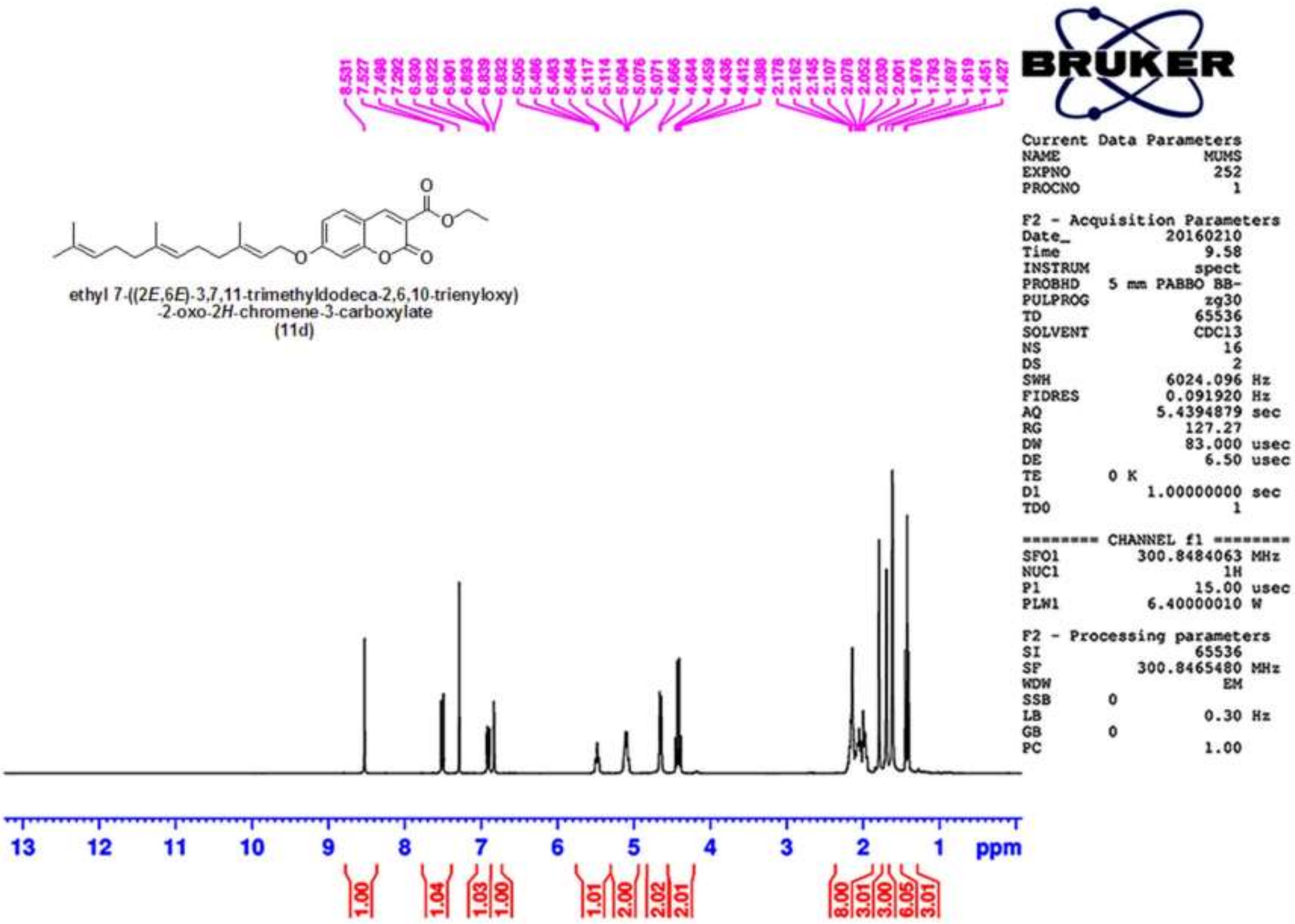

ethyl 7-((2*E*,6*E*)-3,7,11-trimethyldodeca-2,6,10-trienyloxy)
-2-oxo-2*H*-chromene-3-carboxylate
(11d)

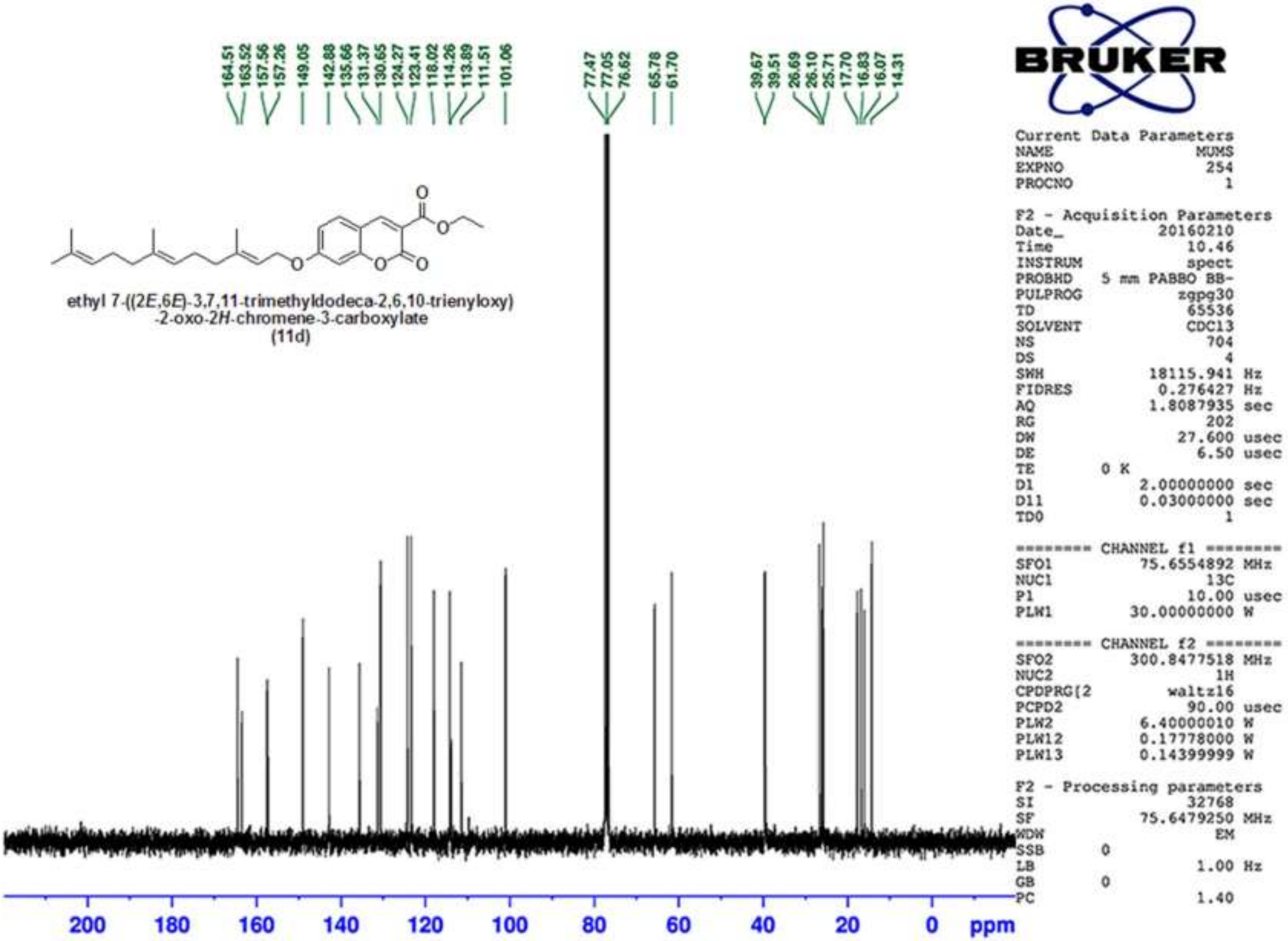

164.51
163.52
157.56
157.26
149.05
142.88
135.66
131.37
130.65
124.27
123.41
118.02
114.26
113.89
111.51
101.06
77.47
77.05
76.62
65.78
61.70
39.67
39.51
26.69
26.10
25.71
17.70
16.83
16.07
14.31
ethyl 7-((2*E*,6*E*)-3,7,11-trimethyldodeca-2,6,10-trienyloxy)
-2-oxo-2*H*-chromene-3-carboxylate
(11d)
200
180
160
140
120
100
80
60
40
20
0
ppm
Current Data Parameters
NAME MUMS
EXPNO 254
PROCNO 1
F2 - Acquisition Parameters
Date_ 20160210
Time 10.46
INSTRUM spect
PROBHD 5 mm PABBO BB-
PULPROG zgpg30
TD 65536
SOLVENT CDCl3
NS 704
DS 4
SWH 18115.941 Hz
FIDRES 0.276427 Hz
AQ 1.8087935 sec
RG 202
DW 27.600 usec
DE 6.50 usec
TE 0 K
D1 2.00000000 sec
D11 0.03000000 sec
TD0 1
======== CHANNEL f1 ========
SFO1 75.6554892 MHz
NUC1 13C
P1 10.00 usec
PLW1 30.00000000 W
======== CHANNEL f2 ========
SFO2 300.8477518 MHz
NUC2 1H
CPDPRG[2 waltz16
PCPD2 90.00 usec
PLW2 6.40000010 W
PLW12 0.17778000 W
PLW13 0.14399999 W
F2 - Processing parameters
SI 32768
SF 75.6479250 MHz
WDW EM
SSB 0
LB 1.00 Hz
GB 0
PC 1.40

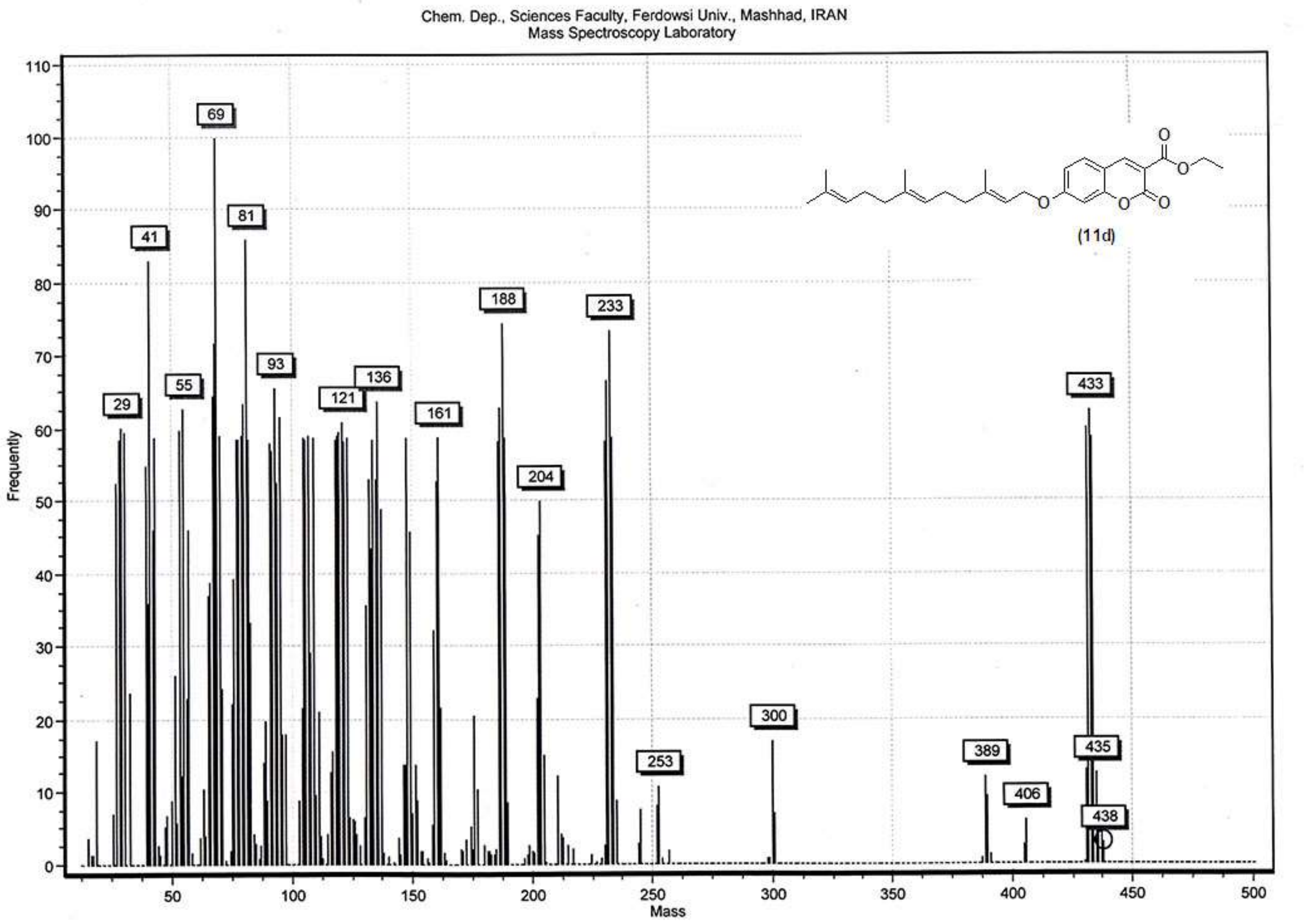

Chem. Dep., Sciences Faculty, Ferdowsi Univ., Mashhad, IRAN
Mass Spectroscopy Laboratory
Frequently
Mass
29
41
55
69
81
93
121
136
161
188
204
233
253
300
389
406
433
435
438
(11d)

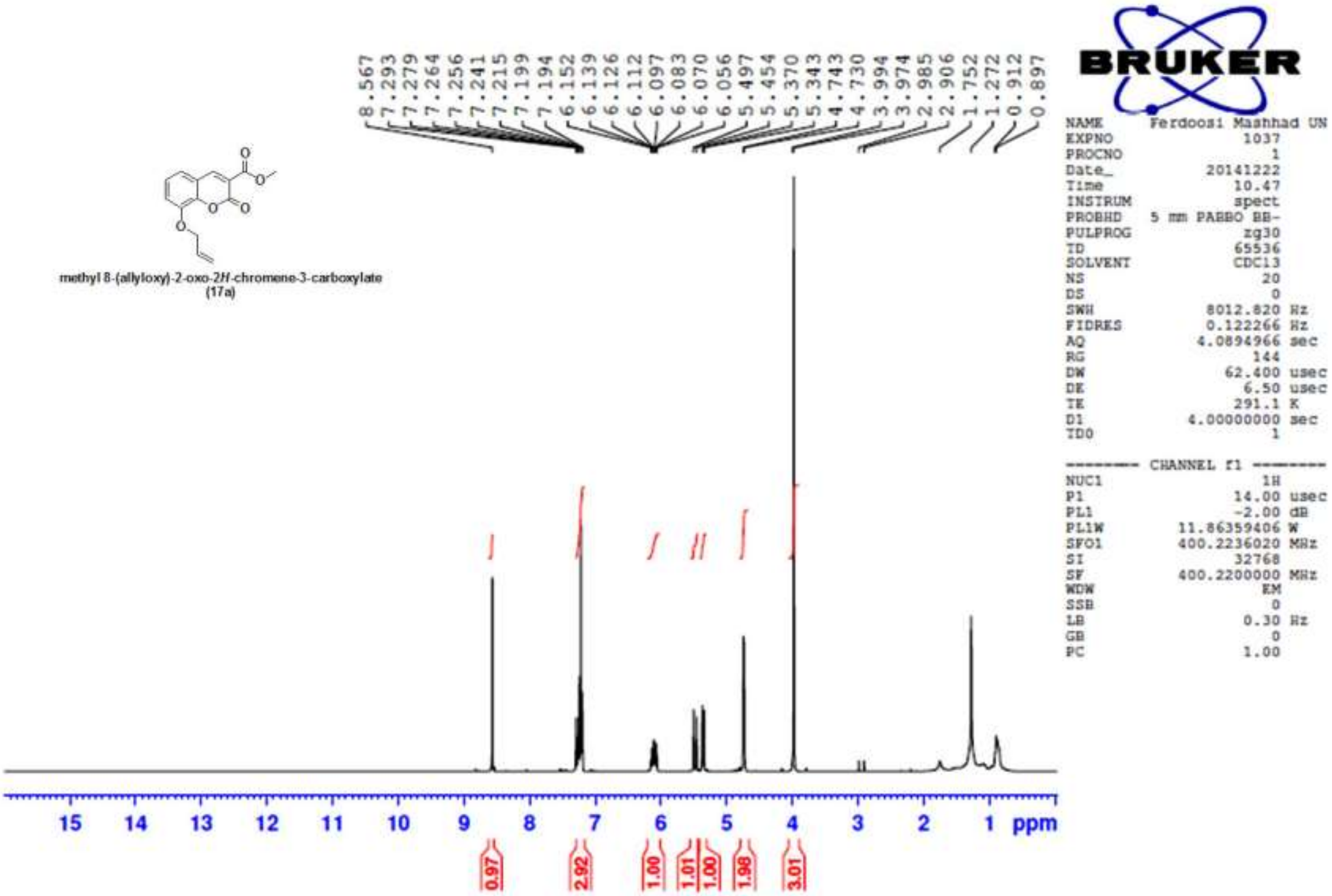
methyl 8-(allyloxy)-2-oxo-2H-chromene-3-carboxylate
(17a)
8.567
7.293
7.279
7.264
7.256
7.241
7.215
7.199
7.194
6.152
6.139
6.126
6.112
6.097
6.083
6.070
6.056
5.497
5.454
5.370
5.343
4.743
4.730
3.994
3.974
2.985
2.906
1.752
1.272
0.912
0.897
BRUKER
NAME Ferdoosi Mashhad UN
EXPNO 1037
PROCNO 1
Date_ 20141222
Time 10.47
INSTRUM spect
PROBHD 5 mm PABBO BB-
PULPROG zg30
TD 65536
SOLVENT CDCl3
NS 20
DS 0
SWH 8012.820 Hz
FIDRES 0.122266 Hz
AQ 4.0894966 sec
RG 144
DW 62.400 usec
DE 6.50 usec
TE 291.1 K
D1 4.00000000 sec
TD0 1
======== CHANNEL f1 ========
NUC1 1H
P1 14.00 usec
PL1 -2.00 dB
PL1W 11.86359406 W
SFO1 400.2236020 MHz
SI 32768
SF 400.2200000 MHz
WDW EM
SSB 0
LB 0.30 Hz
GB 0
PC 1.00
15
14
13
12
11
10
9
8
7
6
5
4
3
2
1
ppm
0.97
2.92
1.00
1.01
1.00
1.98
3.01

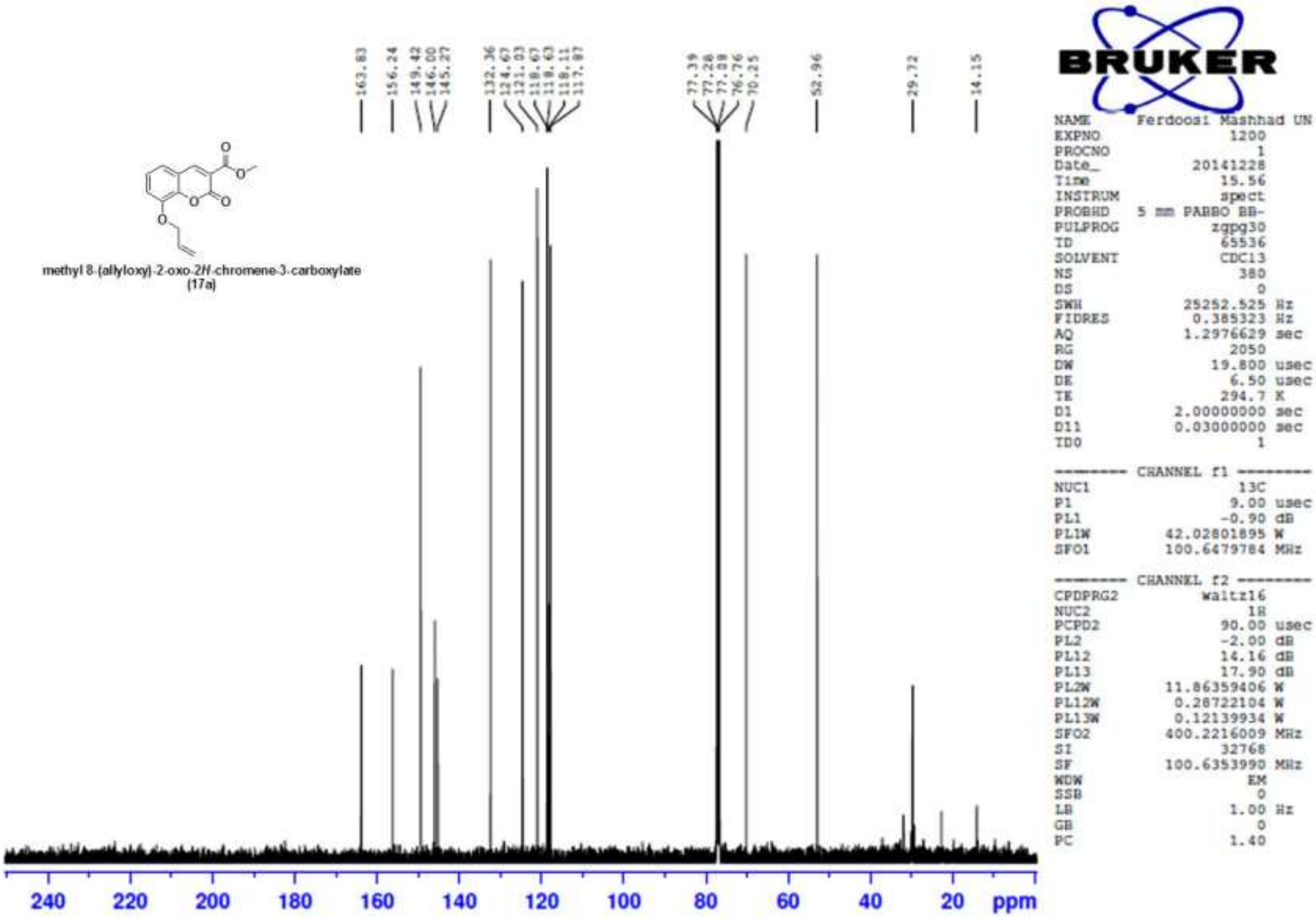
methyl 8-(allyloxy)-2-oxo-2H-chromene-3-carboxylate
(17a)
163.83
156.24
149.42
146.00
145.27
132.36
124.67
121.03
118.67
118.43
118.11
117.87
77.39
77.28
77.08
76.76
70.25
52.96
29.72
14.15
240 220 200 180 160 140 120 100 80 60 40 20 ppm
BRUKER
NAME Ferdoosi Mashhad UN
EXPNO 1200
PROCNO 1
Date_ 20141228
Time 15.56
INSTRUM spect
PROBHD 5 mm PABBO BB-
PULPROG zgpg30
TD 65536
SOLVENT CDCl3
NS 380
DS 0
SWH 25252.525 Hz
FIDRES 0.385323 Hz
AQ 1.2976629 sec
RG 2050
DW 19.800 usec
DE 6.50 usec
TE 294.7 K
D1 2.00000000 sec
D11 0.03000000 sec
TD0 1
======== CHANNEL f1 ========
NUC1 13C
P1 9.00 usec
PL1 -0.90 dB
PL1W 42.02801895 W
SFO1 100.6479784 MHz
======== CHANNEL f2 ========
CPDPRG2 waltz16
NUC2 1H
PCPD2 90.00 usec
PL2 -2.00 dB
PL12 14.16 dB
PL13 17.90 dB
PL2W 11.86359406 W
PL12W 0.28722104 W
PL13W 0.12139934 W
SFO2 400.2216009 MHz
SI 32768
SF 100.6353990 MHz
WDW EM
SSB 0
LB 1.00 Hz
GB 0
PC 1.40

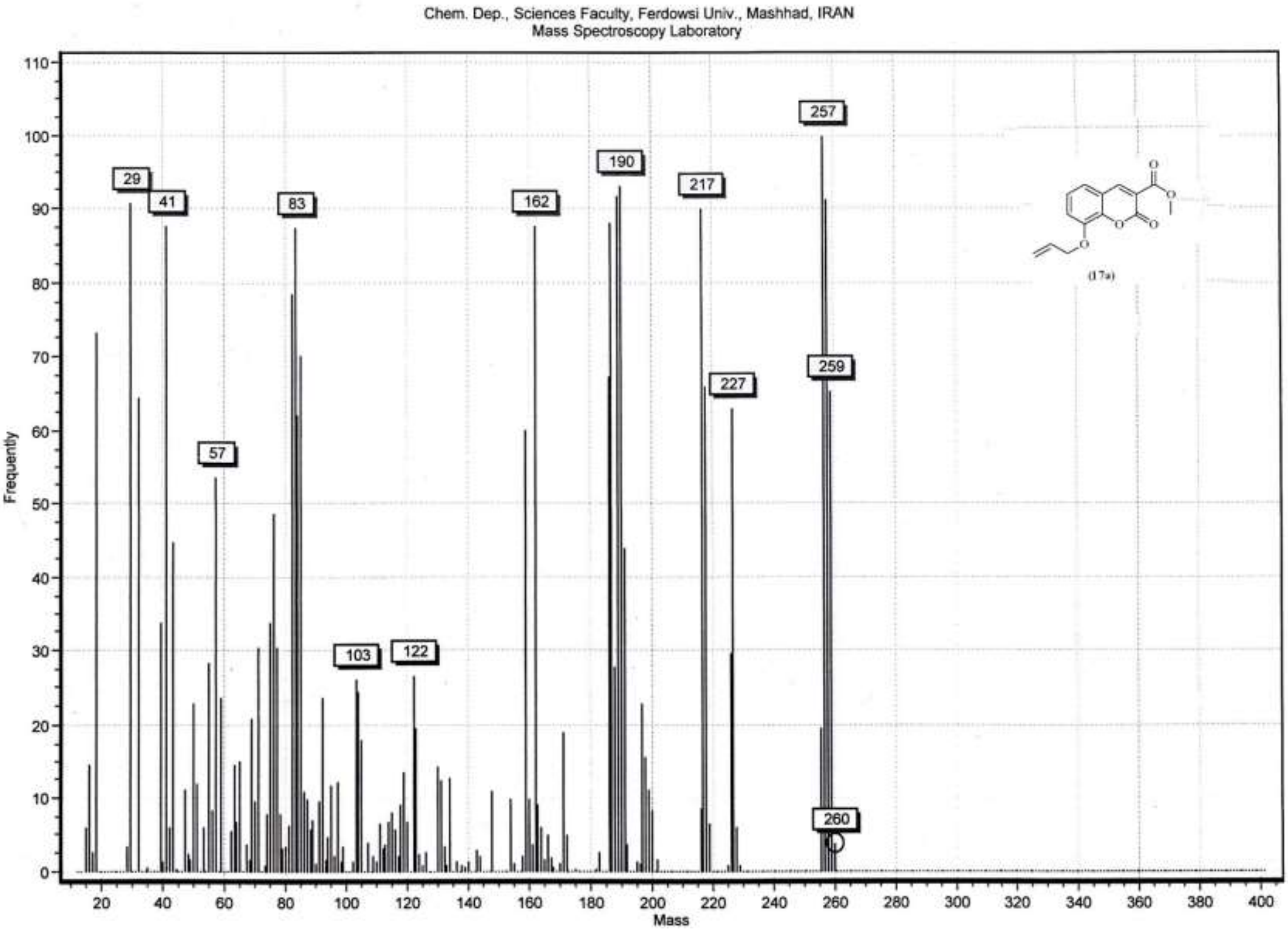
Chem. Dep., Sciences Faculty, Ferdowsi Univ., Mashhad, IRAN
Mass Spectroscopy Laboratory
Frequently
Mass
29
41
57
83
103
122
162
190
217
227
257
259
260
(17a)

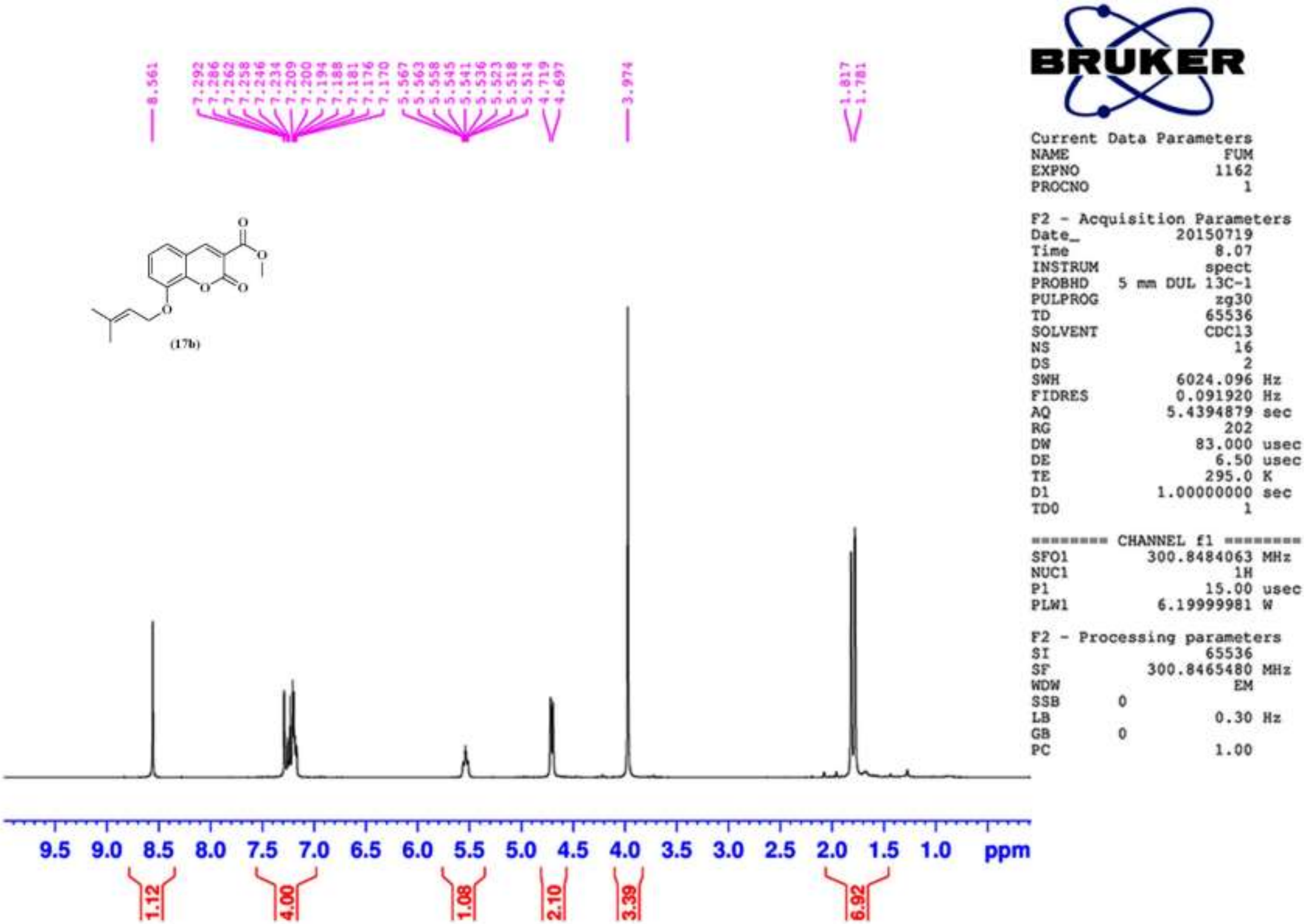
8.561
7.292 7.286 7.262 7.258 7.246 7.234 7.209 7.200 7.194 7.188 7.181 7.176 7.170
5.567 5.563 5.558 5.545 5.541 5.536 5.523 5.518 5.514
4.719 4.697
3.974
1.817 1.781
(17b)
BRUKER
Current Data Parameters
NAME FUM
EXPNO 1162
PROCNO 1
F2 - Acquisition Parameters
Date_ 20150719
Time 8.07
INSTRUM spect
PROBHD 5 mm DUL 13C-1
PULPROG zg30
TD 65536
SOLVENT CDCl3
NS 16
DS 2
SWH 6024.096 Hz
FIDRES 0.091920 Hz
AQ 5.4394879 sec
RG 202
DW 83.000 usec
DE 6.50 usec
TE 295.0 K
D1 1.00000000 sec
TD0 1
======== CHANNEL f1 ========
SFO1 300.8484063 MHz
NUC1 1H
P1 15.00 usec
PLW1 6.19999981 W
F2 - Processing parameters
SI 65536
SF 300.8465480 MHz
WDW EM
SSB 0
LB 0.30 Hz
GB 0
PC 1.00
9.5 9.0 8.5 8.0 7.5 7.0 6.5 6.0 5.5 5.0 4.5 4.0 3.5 3.0 2.5 2.0 1.5 1.0 ppm
1.12 4.00 1.08 2.10 3.39 6.92

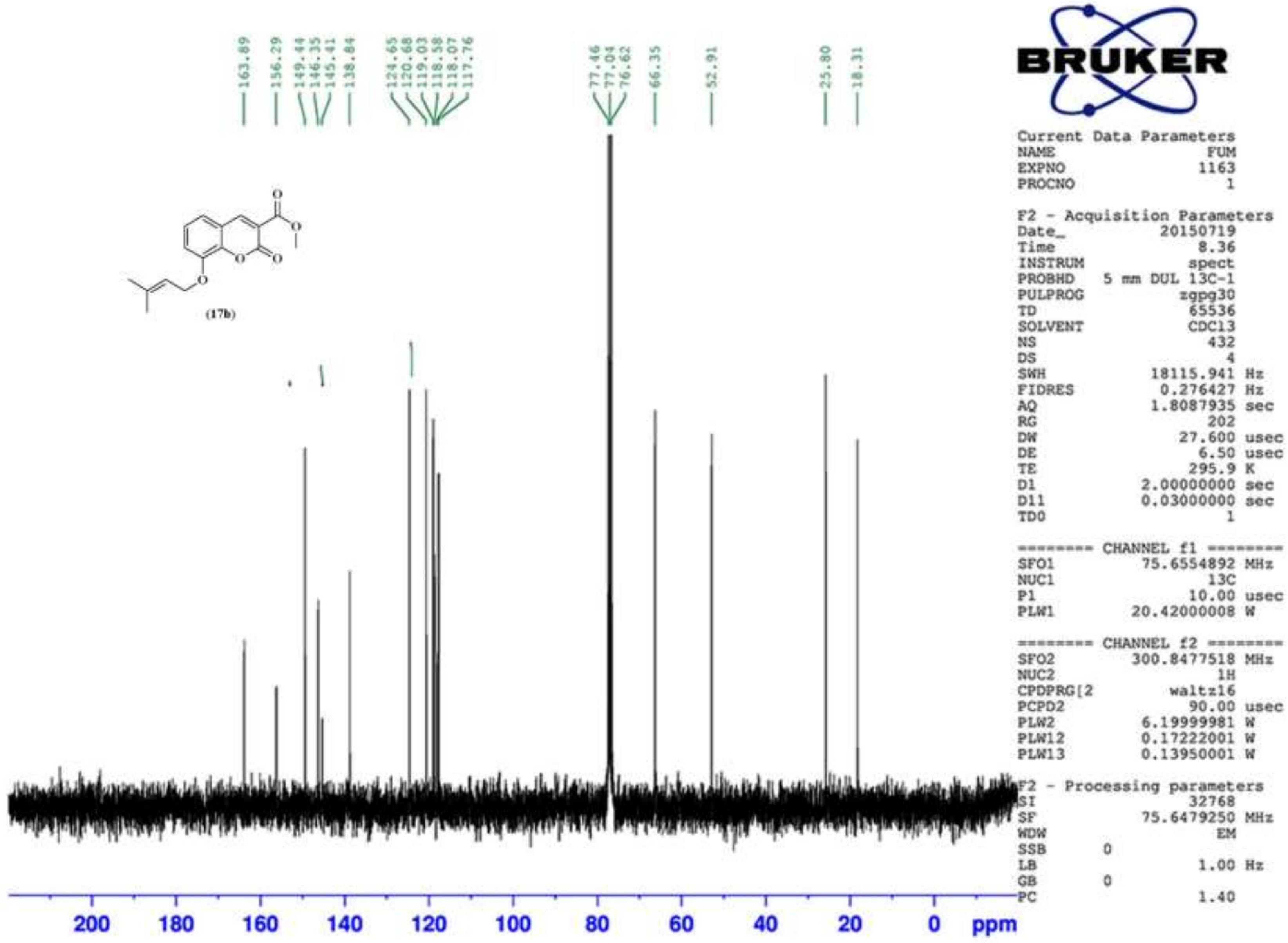
163.89
156.29
149.44
146.35
145.41
138.84
124.65
120.68
119.03
118.58
118.07
117.76
77.46
77.04
76.62
66.35
52.91
25.80
18.31
(17b)
BRUKER
Current Data Parameters
NAME FUM
EXPNO 1163
PROCNO 1
F2 - Acquisition Parameters
Date_ 20150719
Time 8.36
INSTRUM spect
PROBHD 5 mm DUL 13C-1
PULPROG zgpg30
TD 65536
SOLVENT CDCl3
NS 432
DS 4
SWH 18115.941 Hz
FIDRES 0.276427 Hz
AQ 1.8087935 sec
RG 202
DW 27.600 usec
DE 6.50 usec
TE 295.9 K
D1 2.00000000 sec
D11 0.03000000 sec
TD0 1
======== CHANNEL f1 ========
SFO1 75.6554892 MHz
NUC1 13C
P1 10.00 usec
PLW1 20.42000008 W
======== CHANNEL f2 ========
SFO2 300.8477518 MHz
NUC2 1H
CPDPRG[2 waltz16
PCPD2 90.00 usec
PLW2 6.19999981 W
PLW12 0.17222001 W
PLW13 0.13950001 W
F2 - Processing parameters
SI 32768
SF 75.6479250 MHz
WDW EM
SSB 0
LB 1.00 Hz
GB 0
PC 1.40
200 180 160 140 120 100 80 60 40 20 0 ppm

Chem. Dep., Sciences Faculty, Ferdowsi Univ., Mashhad, IRAN
Mass Spectroscopy Laboratory

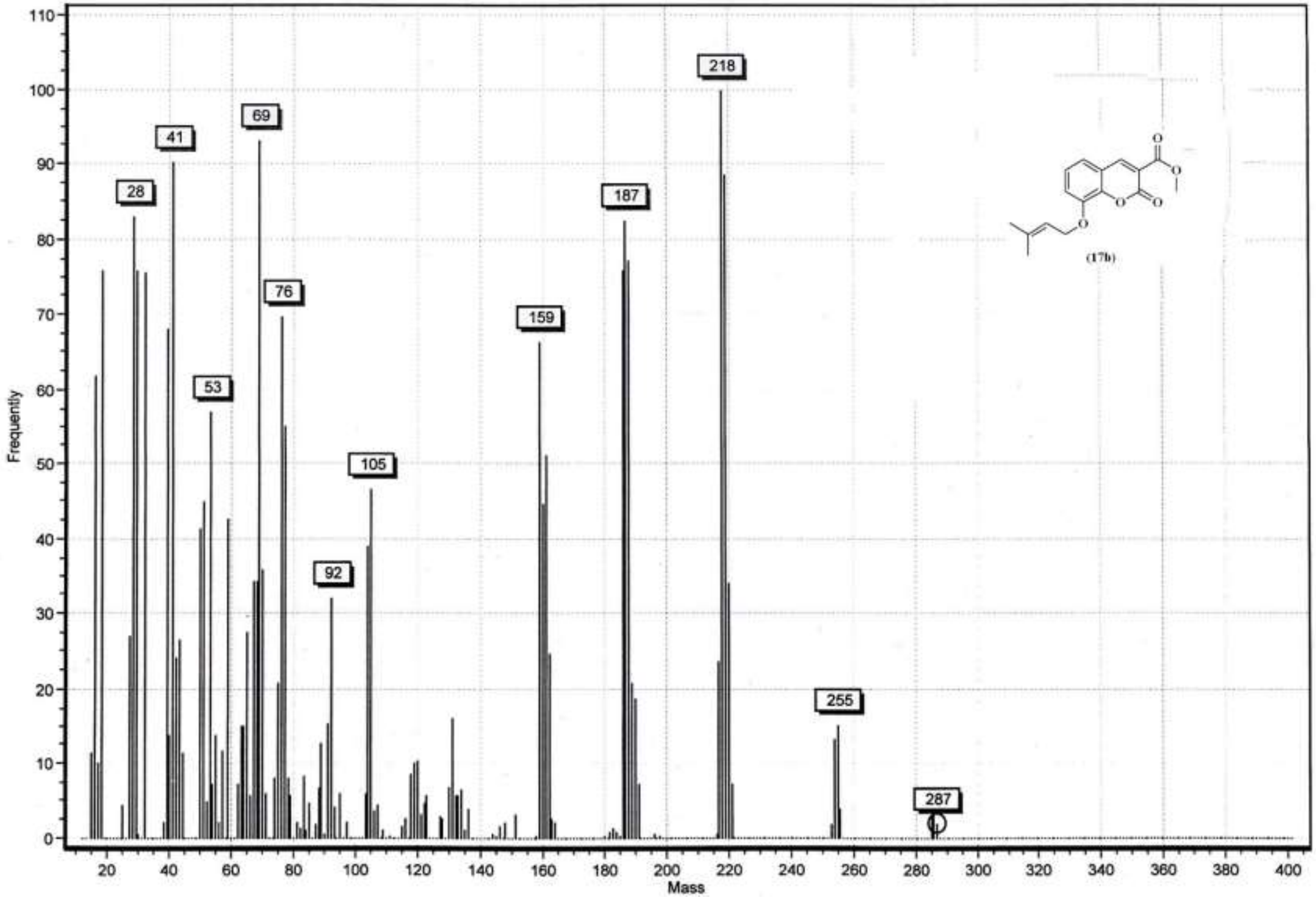

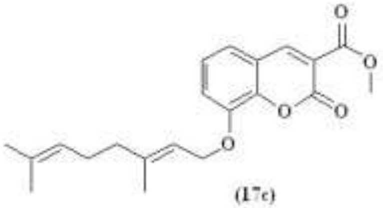

(17c)

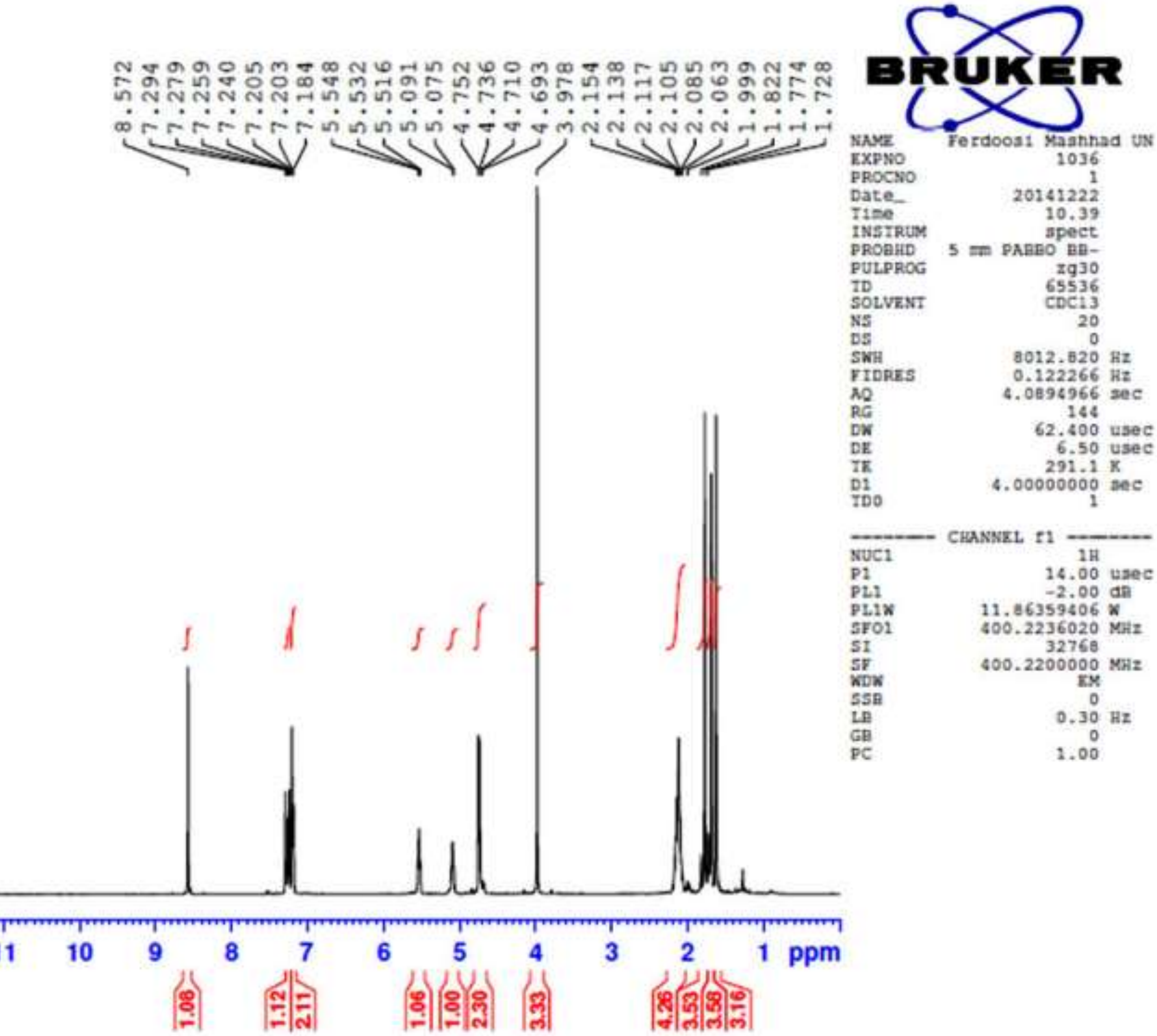
NAME Ferdoosi Mashhad UN
EXPNO 1036
PROCNO 1
Date_ 20141222
Time 10.39
INSTRUM spect
PROBHD 5 mm PABBO BB-
PULPROG zg30
TD 65536
SOLVENT CDCl3
NS 20
DS 0
SWH 8012.820 Hz
FIDRES 0.122266 Hz
AQ 4.0894966 sec
RG 144
DW 62.400 usec
DE 6.50 usec
TE 291.1 K
D1 4.00000000 sec
TD0 1
CHANNEL f1
NUC1 1H
P1 14.00 usec
PL1 -2.00 dB
PL1W 11.86359406 W
SFO1 400.2236020 MHz
SI 32768
SF 400.2200000 MHz
WDW EM
SSB 0
LB 0.30 Hz
GB 0
PC 1.00
ppm

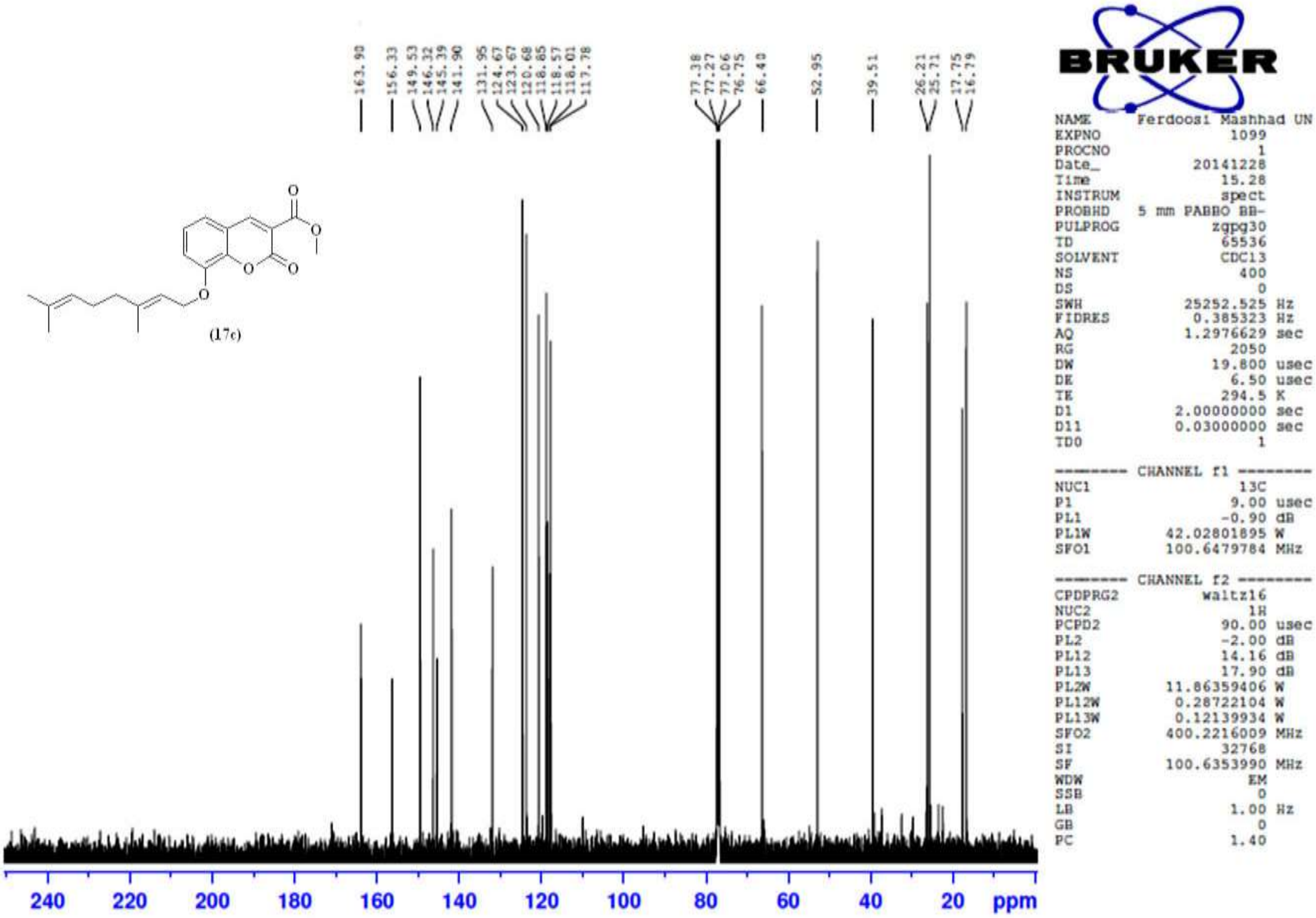
(17c)
163.90
156.33
149.53
146.32
145.39
141.90
131.95
124.67
123.67
120.68
118.85
118.57
118.01
117.78
77.38
77.27
77.06
76.75
66.40
52.95
39.51
26.21
25.71
17.75
16.79
240 220 200 180 160 140 120 100 80 60 40 20 ppm
BRUKER
NAME Ferdoosi Mashhad UN
EXPNO 1099
PROCNO 1
Date_ 20141228
Time 15.28
INSTRUM spect
PROBHD 5 mm PABBO BB-
PULPROG zgpg30
TD 65536
SOLVENT CDCl3
NS 400
DS 0
SWH 25252.525 Hz
FIDRES 0.385323 Hz
AQ 1.2976629 sec
RG 2050
DW 19.800 usec
DE 6.50 usec
TE 294.5 K
D1 2.00000000 sec
D11 0.03000000 sec
TD0 1
======== CHANNEL f1 ========
NUC1 13C
P1 9.00 usec
PL1 -0.90 dB
PL1W 42.02801895 W
SFO1 100.6479784 MHz
======== CHANNEL f2 ========
CPDPRG2 waltz16
NUC2 1H
PCPD2 90.00 usec
PL2 -2.00 dB
PL12 14.16 dB
PL13 17.90 dB
PL2W 11.86359406 W
PL12W 0.28722104 W
PL13W 0.12139934 W
SFO2 400.2216009 MHz
SI 32768
SF 100.6353990 MHz
WDW EM
SSB 0
LB 1.00 Hz
GB 0
PC 1.40

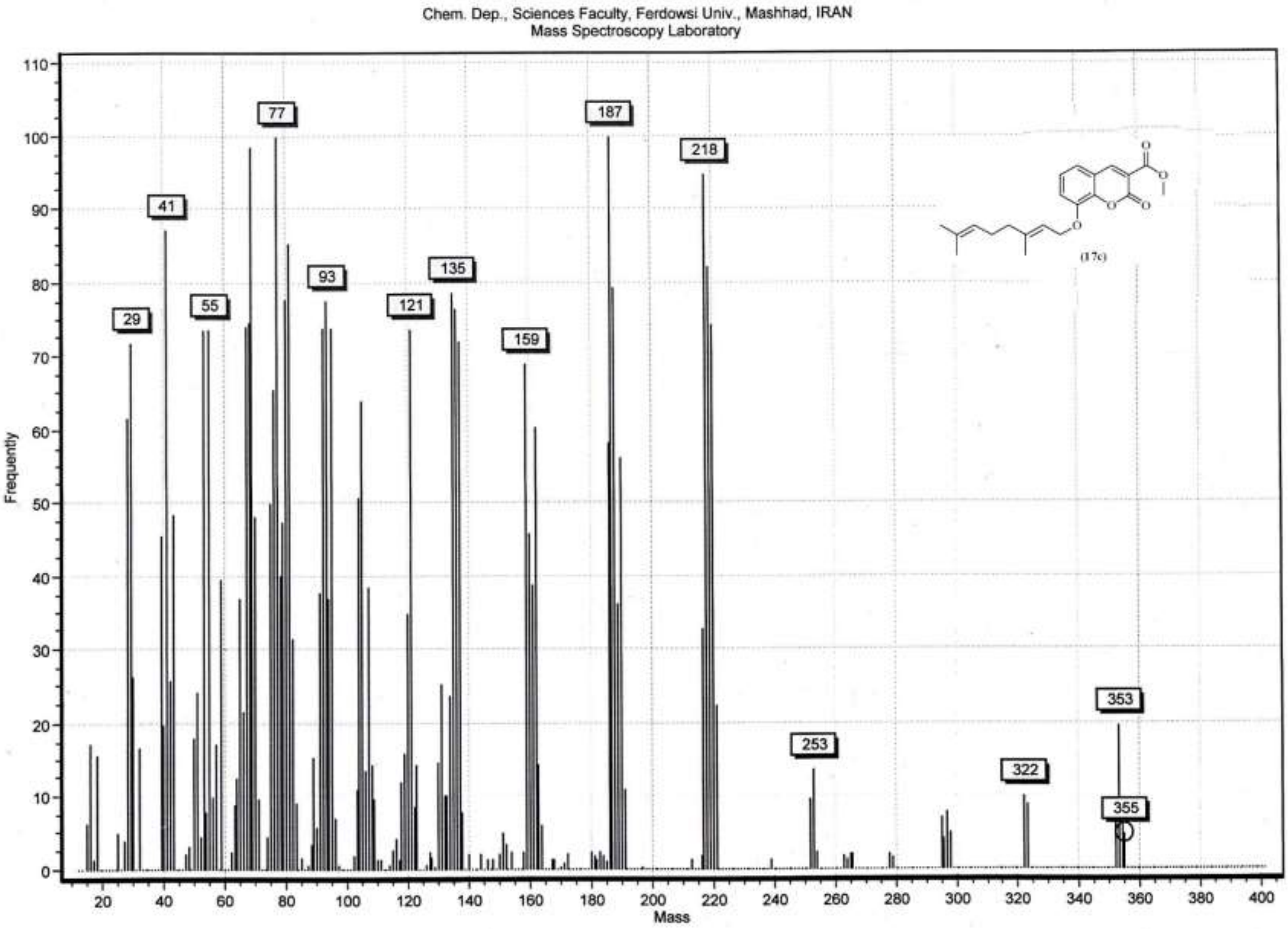
Chem. Dep., Sciences Faculty, Ferdowsi Univ., Mashhad, IRAN
Mass Spectroscopy Laboratory
Frequently
Mass
29
41
55
77
93
121
135
159
187
218
253
322
353
355
(17c)

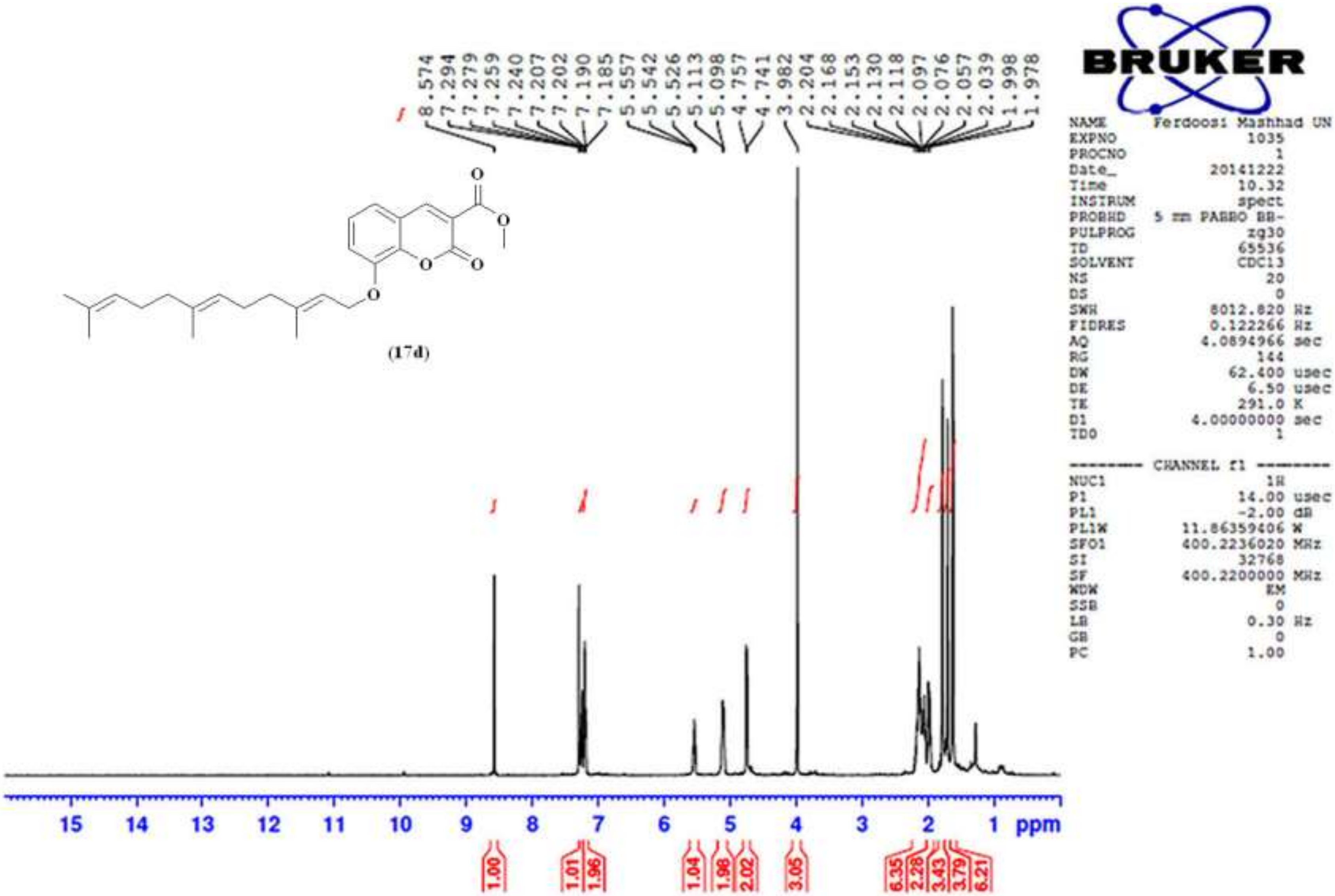
8.574
7.294
7.279
7.259
7.240
7.207
7.202
7.190
7.185
5.557
5.542
5.526
5.113
5.098
4.757
4.741
3.982
2.204
2.168
2.153
2.130
2.118
2.097
2.076
2.057
2.039
1.998
1.978
BRUKER
NAME Ferdoosi Mashhad UN
EXPNO 1035
PROCNO 1
Date_ 20141222
Time 10.32
INSTRUM spect
PROBHD 5 mm PABBO BB-
PULPROG zg30
TD 65536
SOLVENT CDCl3
NS 20
DS 0
SWH 8012.820 Hz
FIDRES 0.122266 Hz
AQ 4.0894966 sec
RG 144
DW 62.400 usec
DE 6.50 usec
TE 291.0 K
D1 4.00000000 sec
TD0 1
CHANNEL f1
NUC1 1H
P1 14.00 usec
PL1 -2.00 dB
PL1W 11.86359406 W
SFO1 400.2236020 MHz
SI 32768
SF 400.2200000 MHz
WDW EM
SSB 0
LB 0.30 Hz
GB 0
PC 1.00
(17d)
15 14 13 12 11 10 9 8 7 6 5 4 3 2 1 ppm
1.00
1.01
1.96
1.04
1.98
2.02
3.05
6.35
2.28
3.43
3.79
6.21

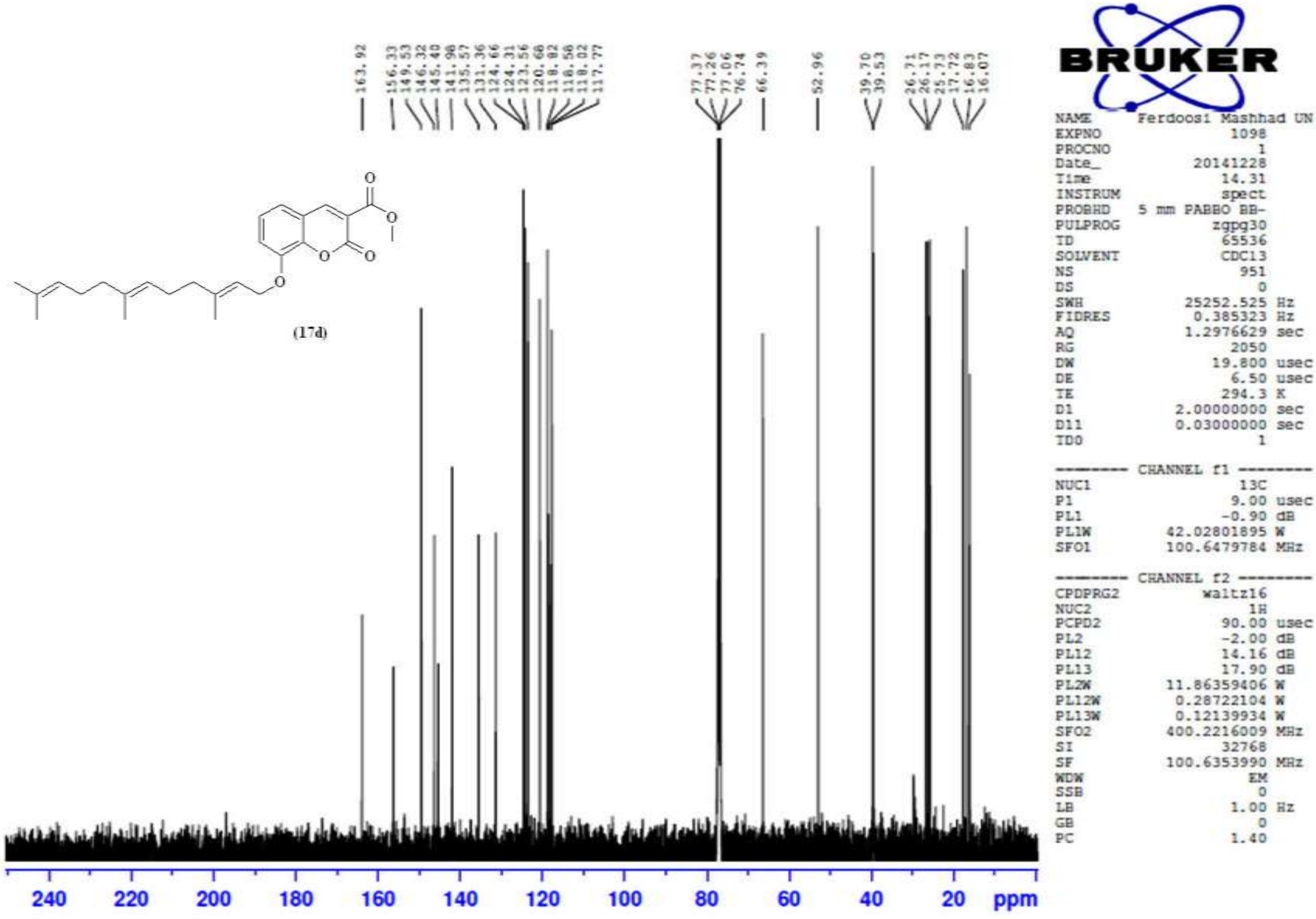
163.92
156.33
149.53
146.32
145.40
141.98
135.57
131.36
124.66
124.31
123.56
120.68
118.82
118.56
118.02
117.77
77.37
77.26
77.06
76.74
66.39
52.96
39.70
39.53
26.71
26.17
25.73
17.72
16.83
16.07
(17d)
240
220
200
180
160
140
120
100
80
60
40
20
ppm
NAME Ferdoosi Mashhad UN
EXPNO 1098
PROCNO 1
Date_ 20141228
Time 14.31
INSTRUM spect
PROBHD 5 mm PABBO BB-
PULPROG zgpg30
TD 65536
SOLVENT CDCl3
NS 951
DS 0
SWH 25252.525 Hz
FIDRES 0.385323 Hz
AQ 1.2976629 sec
RG 2050
DW 19.800 usec
DE 6.50 usec
TE 294.3 K
D1 2.00000000 sec
D11 0.03000000 sec
TD0 1
======== CHANNEL f1 ========
NUC1 13C
P1 9.00 usec
PL1 -0.90 dB
PL1W 42.02801895 W
SFO1 100.6479784 MHz
======== CHANNEL f2 ========
CPDPRG2 waltz16
NUC2 1H
PCPD2 90.00 usec
PL2 -2.00 dB
PL12 14.16 dB
PL13 17.90 dB
PL2W 11.86359406 W
PL12W 0.28722104 W
PL13W 0.12139934 W
SFO2 400.2216009 MHz
SI 32768
SF 100.6353990 MHz
WDW EM
SSB 0
LB 1.00 Hz
GB 0
PC 1.40

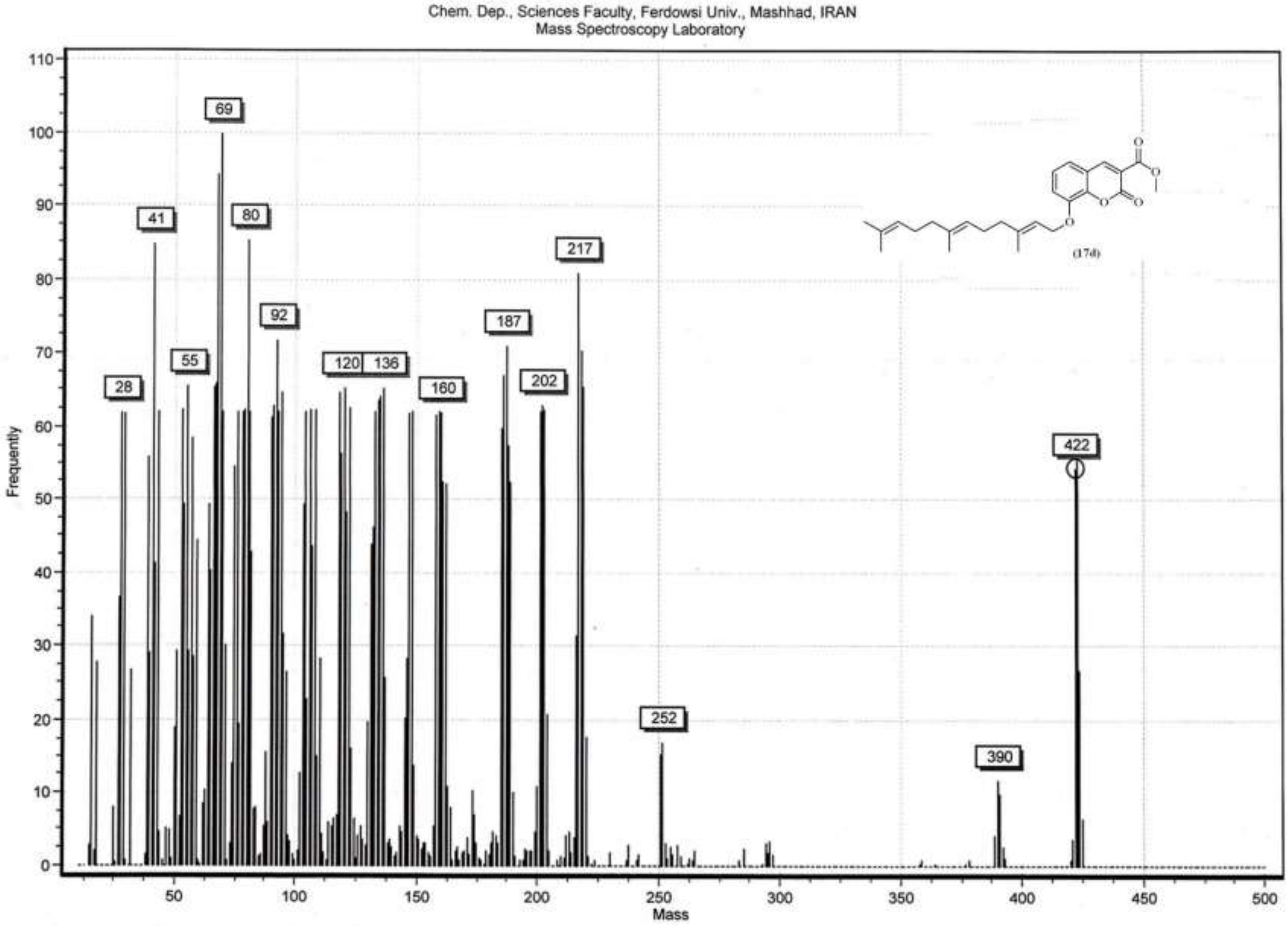

Chem. Dep., Sciences Faculty, Ferdowsi Univ., Mashhad, IRAN
Mass Spectroscopy Laboratory
Frequently
Mass
28
41
55
69
80
92
120
136
160
187
202
217
252
390
422
(17d)

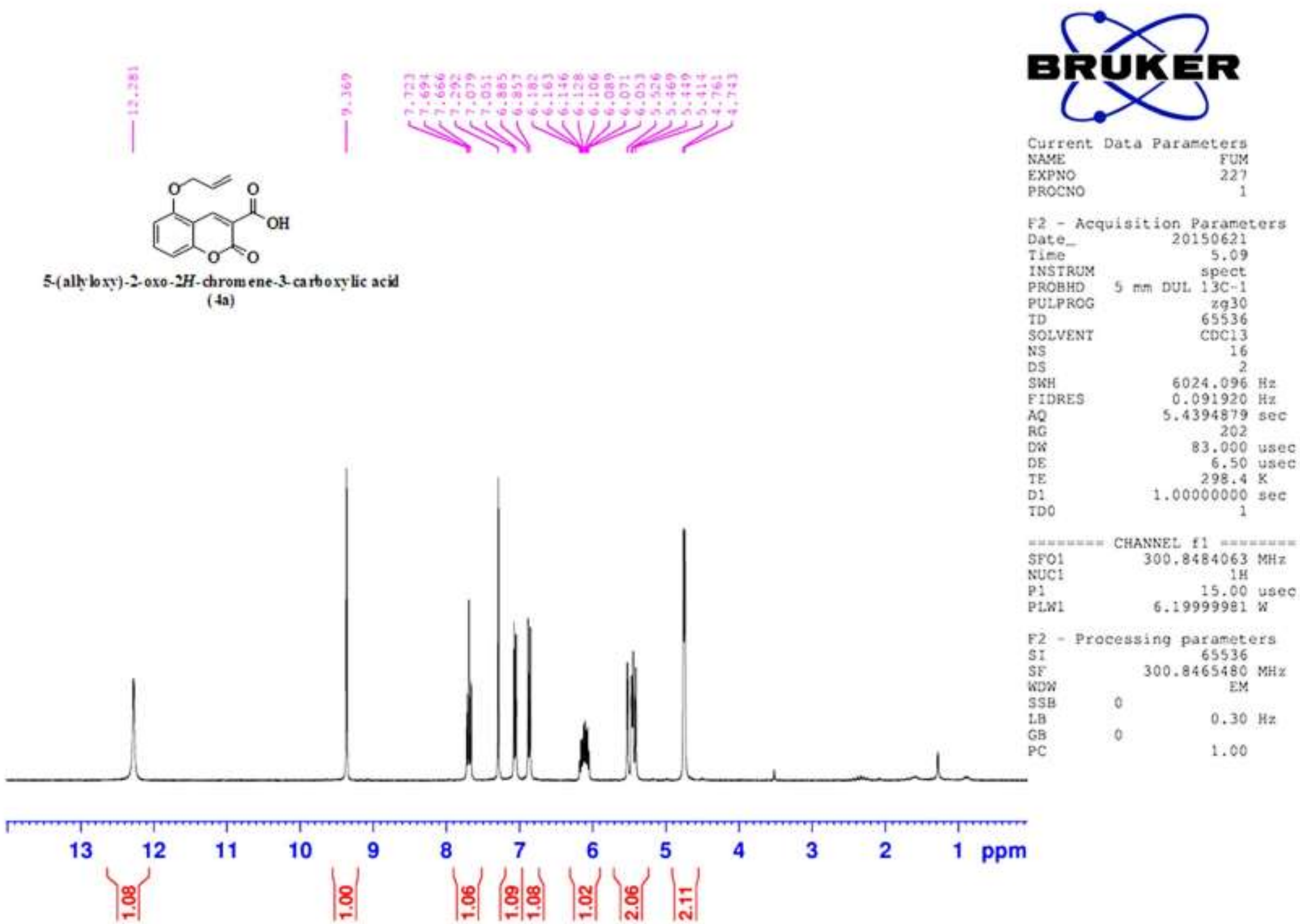
12.281
9.369
7.723 7.694 7.666 7.292 7.079 7.051 6.885 6.857 6.182 6.163 6.146 6.128 6.106 6.089 6.071 6.053 5.526 5.469 5.449 5.414 4.761 4.743
OH
O
5-(allyloxy)-2-oxo-2H-chromene-3-carboxylic acid
(4a)
13 12 11 10 9 8 7 6 5 4 3 2 1 ppm
1.08 1.00 1.06 1.09 1.08 1.02 2.06 2.11
BRUKER
Current Data Parameters
NAME FUM
EXPNO 227
PROCNO 1
F2 - Acquisition Parameters
Date_ 20150621
Time 5.09
INSTRUM spect
PROBHD 5 mm DUL 13C-1
PULPROG zg30
TD 65536
SOLVENT CDCl3
NS 16
DS 2
SWH 6024.096 Hz
FIDRES 0.091920 Hz
AQ 5.4394879 sec
RG 202
DW 83.000 usec
DE 6.50 usec
TE 298.4 K
D1 1.00000000 sec
TD0 1
======== CHANNEL f1 ========
SFO1 300.8484063 MHz
NUC1 1H
P1 15.00 usec
PLW1 6.19999981 W
F2 - Processing parameters
SI 65536
SF 300.8465480 MHz
WDW EM
SSB 0
LB 0.30 Hz
GB 0
PC 1.00

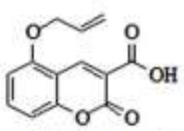

5-(allyloxy)-2-oxo-2*H*-chromene-3-carboxylic acid
(4a)

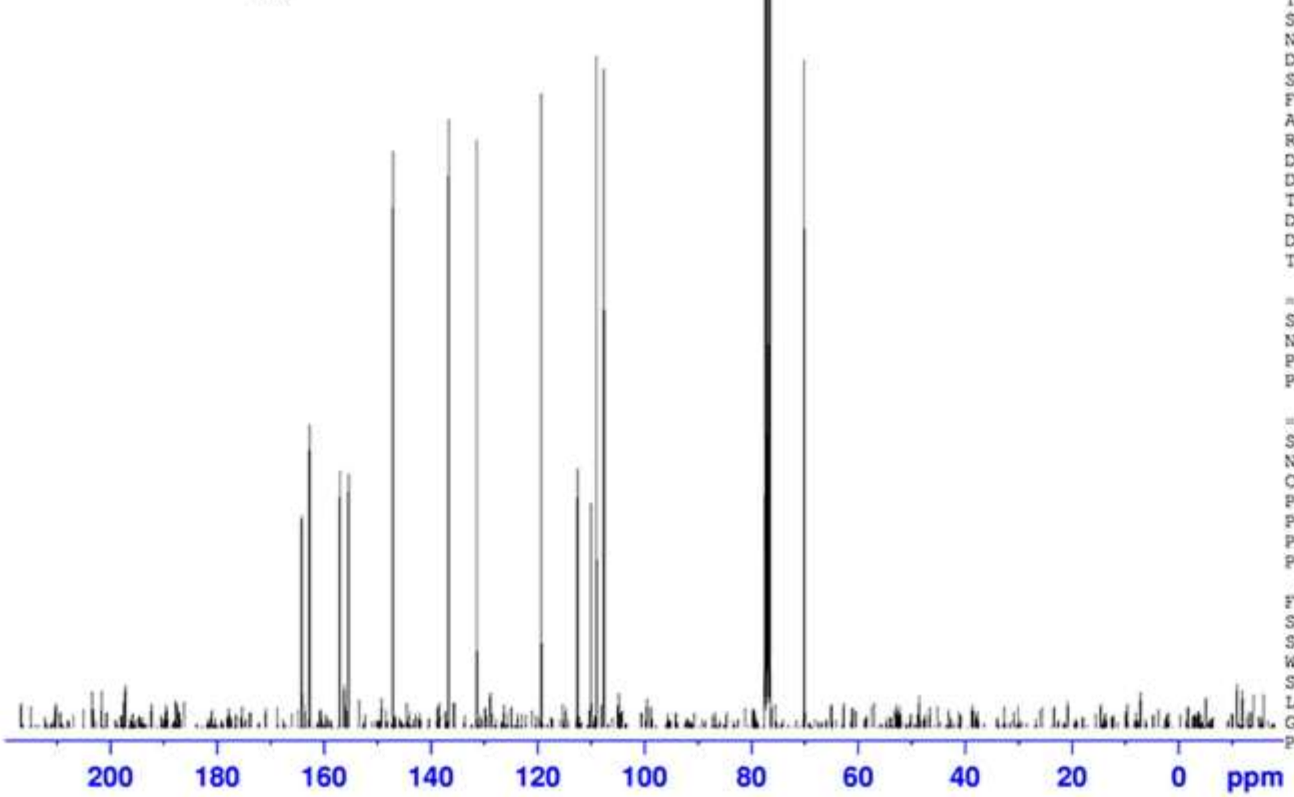

BRUKER

Current Data Parameters
NAME FUM
EXPNO 237
PROCNO 1

F2 - Acquisition Parameters
Date_ 20150621
Time 7.56
INSTRUM spect
PROBHD 5 mm DUL 13C-1
PULPROG zgpg30
TD 65536
SOLVENT CDCl3
NS 288
DS 4
SWH 18115.941 Hz
FIDRES 0.276427 Hz
AQ 1.8087935 sec
RG 202
DW 27.600 usec
DE 6.50 usec
TE 299.4 K
D1 2.00000000 sec
D11 0.03000000 sec
TD0 1

======== CHANNEL f1 ========
SFO1 75.6554892 MHz
NUC1 13C
P1 10.00 usec
PLW1 20.42000008 W

======== CHANNEL f2 ========
SFO2 300.8477518 MHz
NUC2 1H
CPDPRG[2 waltz16
PCPD2 90.00 usec
PLW2 6.19999981 W
PLW12 0.17222001 W
PLW13 0.13950001 W

F2 - Processing parameters
SI 32768
SF 75.6479250 MHz
WDW EM
SSB 0
LB 1.00 Hz
GB 0
PC 1.40

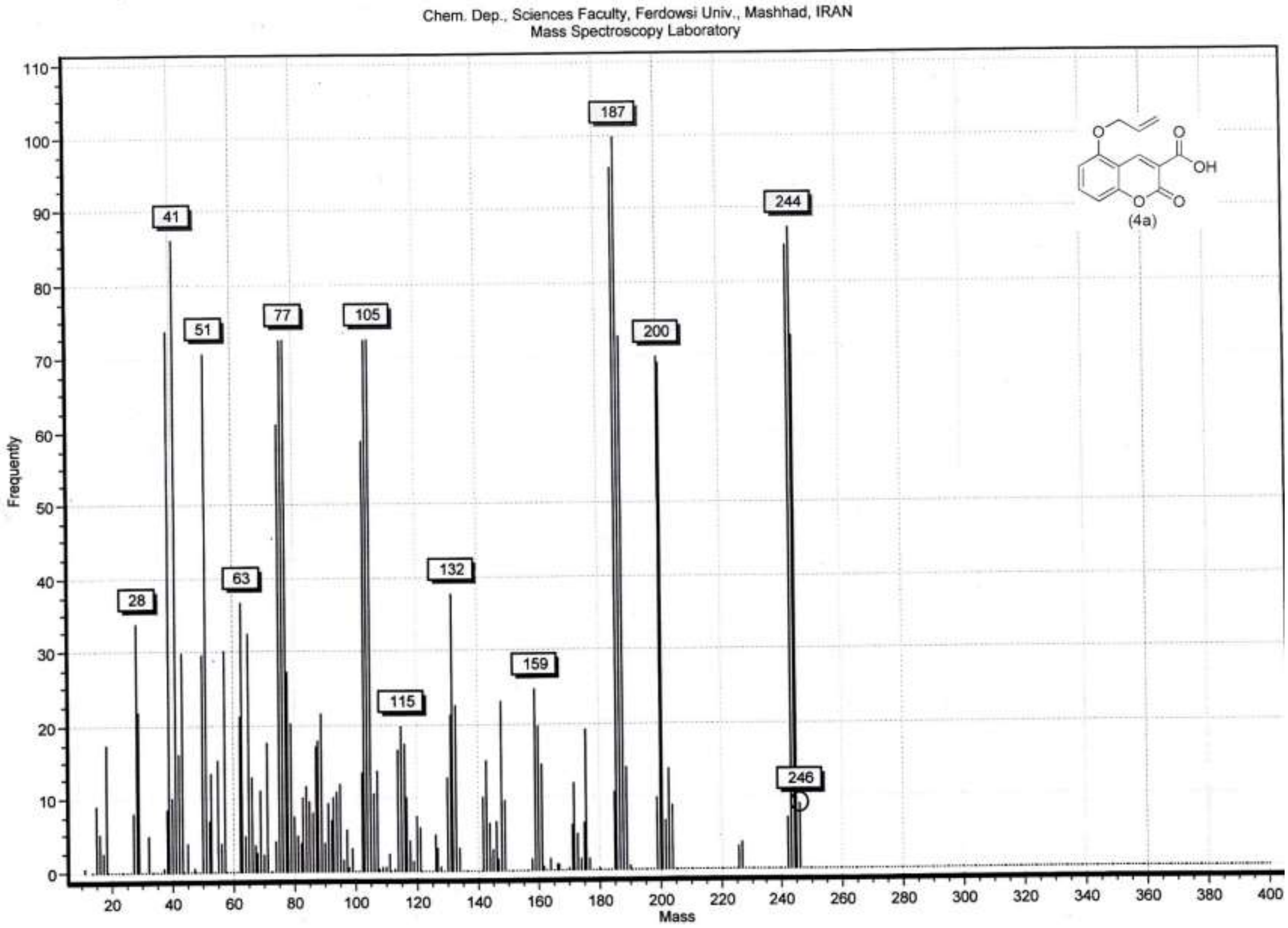
Chem. Dep., Sciences Faculty, Ferdowsi Univ., Mashhad, IRAN
Mass Spectroscopy Laboratory
Frequently
Mass
187
41
244
51
77
105
200
63
28
132
159
115
246
O
OH
(4a)

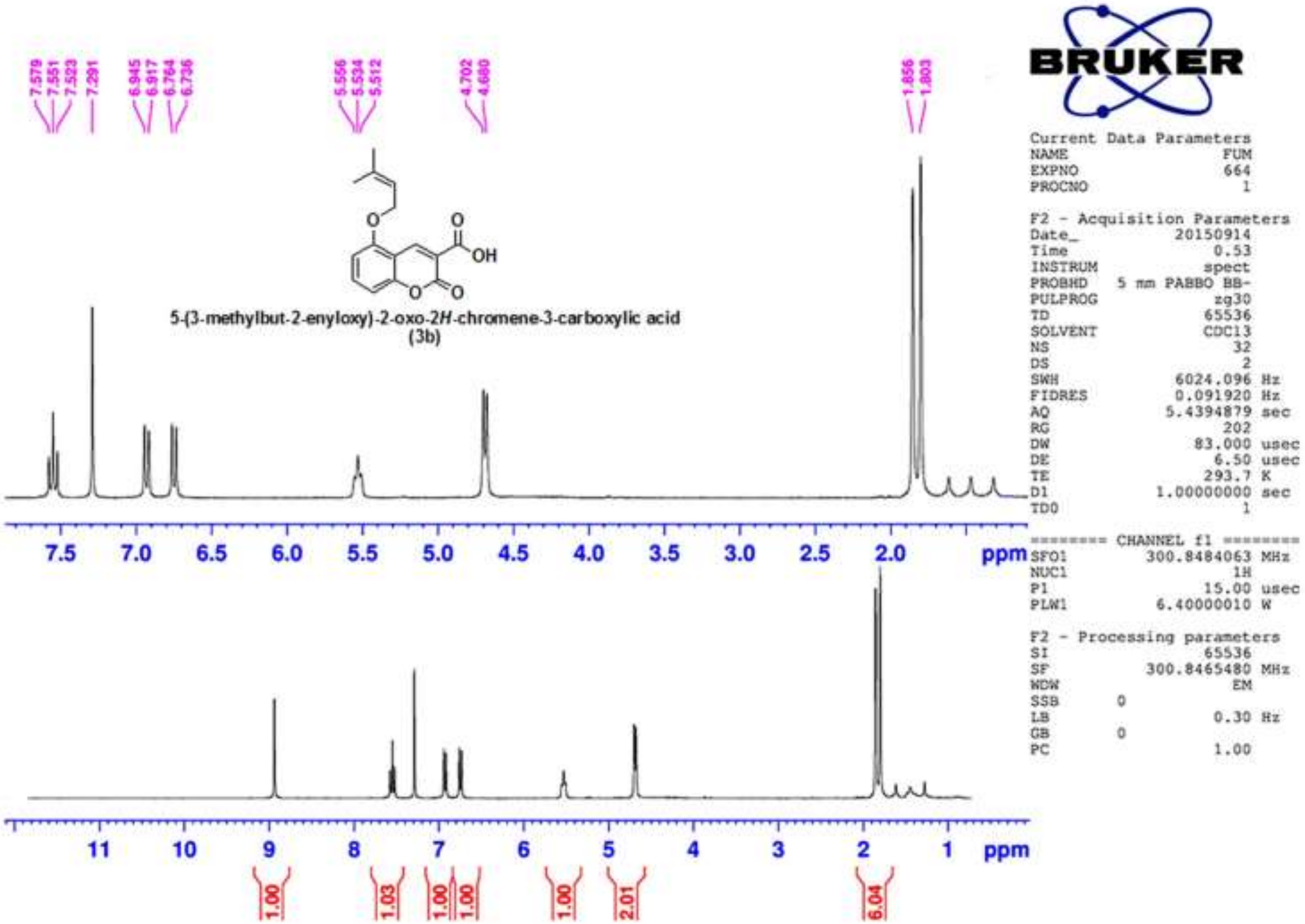
7.579
7.551
7.523
7.291
6.945
6.917
6.764
6.736
5.556
5.534
5.512
4.702
4.680
1.856
1.803
5-(3-methylbut-2-enyloxy)-2-oxo-2H-chromene-3-carboxylic acid
(3b)
BRUKER
Current Data Parameters
NAME FUM
EXPNO 664
PROCNO 1
F2 - Acquisition Parameters
Date_ 20150914
Time 0.53
INSTRUM spect
PROBHD 5 mm PABBO BB-
PULPROG zg30
TD 65536
SOLVENT CDCl3
NS 32
DS 2
SWH 6024.096 Hz
FIDRES 0.091920 Hz
AQ 5.4394879 sec
RG 202
DW 83.000 usec
DE 6.50 usec
TE 293.7 K
D1 1.00000000 sec
TD0 1
======== CHANNEL f1 ========
SFO1 300.8484063 MHz
NUC1 1H
P1 15.00 usec
PLW1 6.40000010 W
F2 - Processing parameters
SI 65536
SF 300.8465480 MHz
WDW EM
SSB 0
LB 0.30 Hz
GB 0
PC 1.00
ppm
1.00
1.03
1.00
1.00
1.00
2.01
6.04

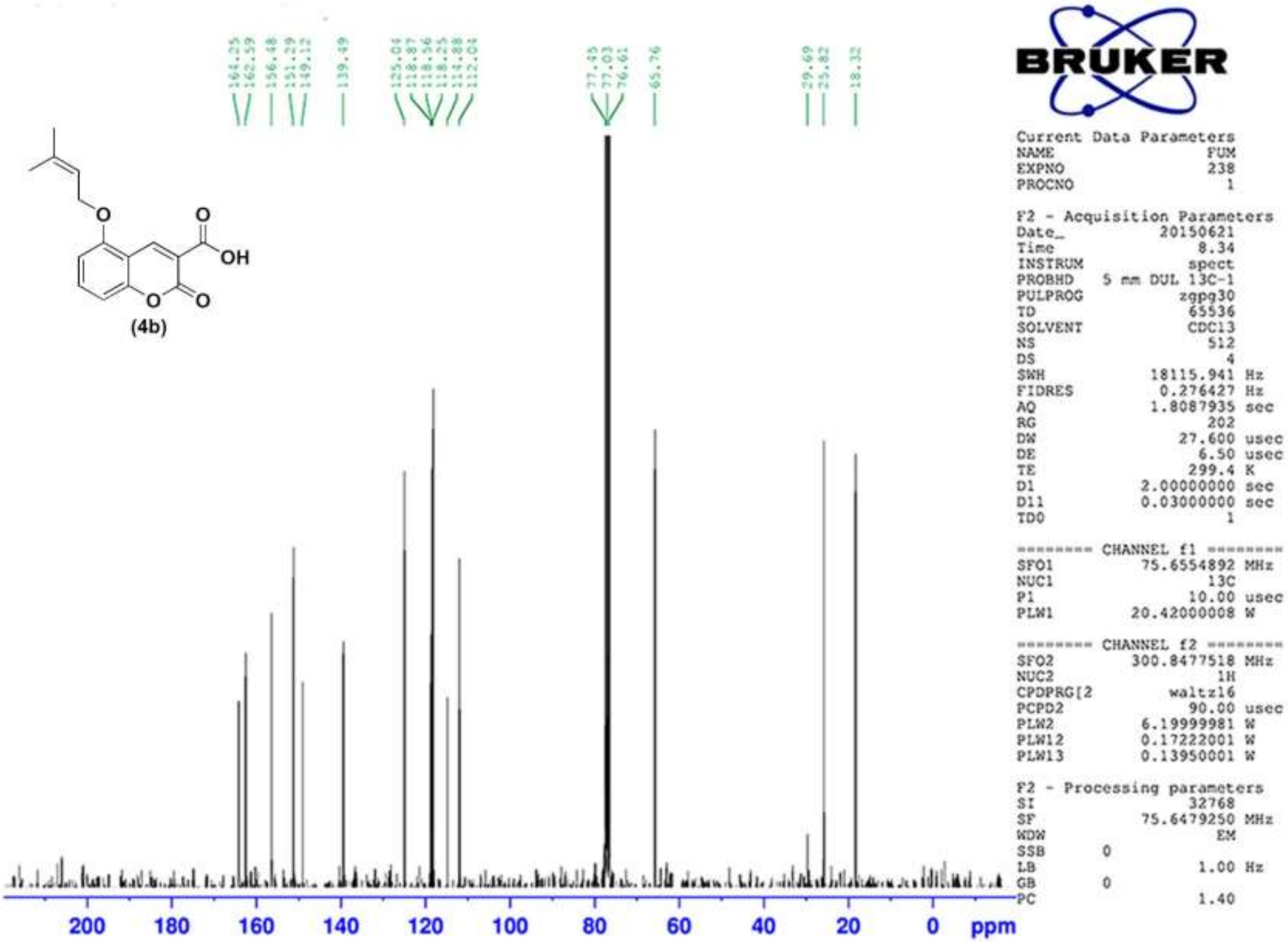

164.25
162.59
156.48
151.29
149.12
139.49
125.04
118.87
118.56
118.25
114.88
112.04
77.45
77.03
76.61
65.76
29.69
25.82
18.32
O
OH
O
O
O
(4b)
BRUKER
Current Data Parameters
NAME FUM
EXPNO 238
PROCNO 1
F2 - Acquisition Parameters
Date_ 20150621
Time 8.34
INSTRUM spect
PROBHD 5 mm DUL 13C-1
PULPROG zgpg30
TD 65536
SOLVENT CDCl3
NS 512
DS 4
SWH 18115.941 Hz
FIDRES 0.276427 Hz
AQ 1.8087935 sec
RG 202
DW 27.600 usec
DE 6.50 usec
TE 299.4 K
D1 2.00000000 sec
D11 0.03000000 sec
TD0 1
======== CHANNEL f1 ========
SFO1 75.6554892 MHz
NUC1 13C
P1 10.00 usec
PLW1 20.42000008 W
======== CHANNEL f2 ========
SFO2 300.8477518 MHz
NUC2 1H
CPDPRG[2 waltz16
PCPD2 90.00 usec
PLW2 6.19999981 W
PLW12 0.17222001 W
PLW13 0.13950001 W
F2 - Processing parameters
SI 32768
SF 75.6479250 MHz
WDW EM
SSB 0
LB 1.00 Hz
GB 0
PC 1.40
200
180
160
140
120
100
80
60
40
20
0
ppm

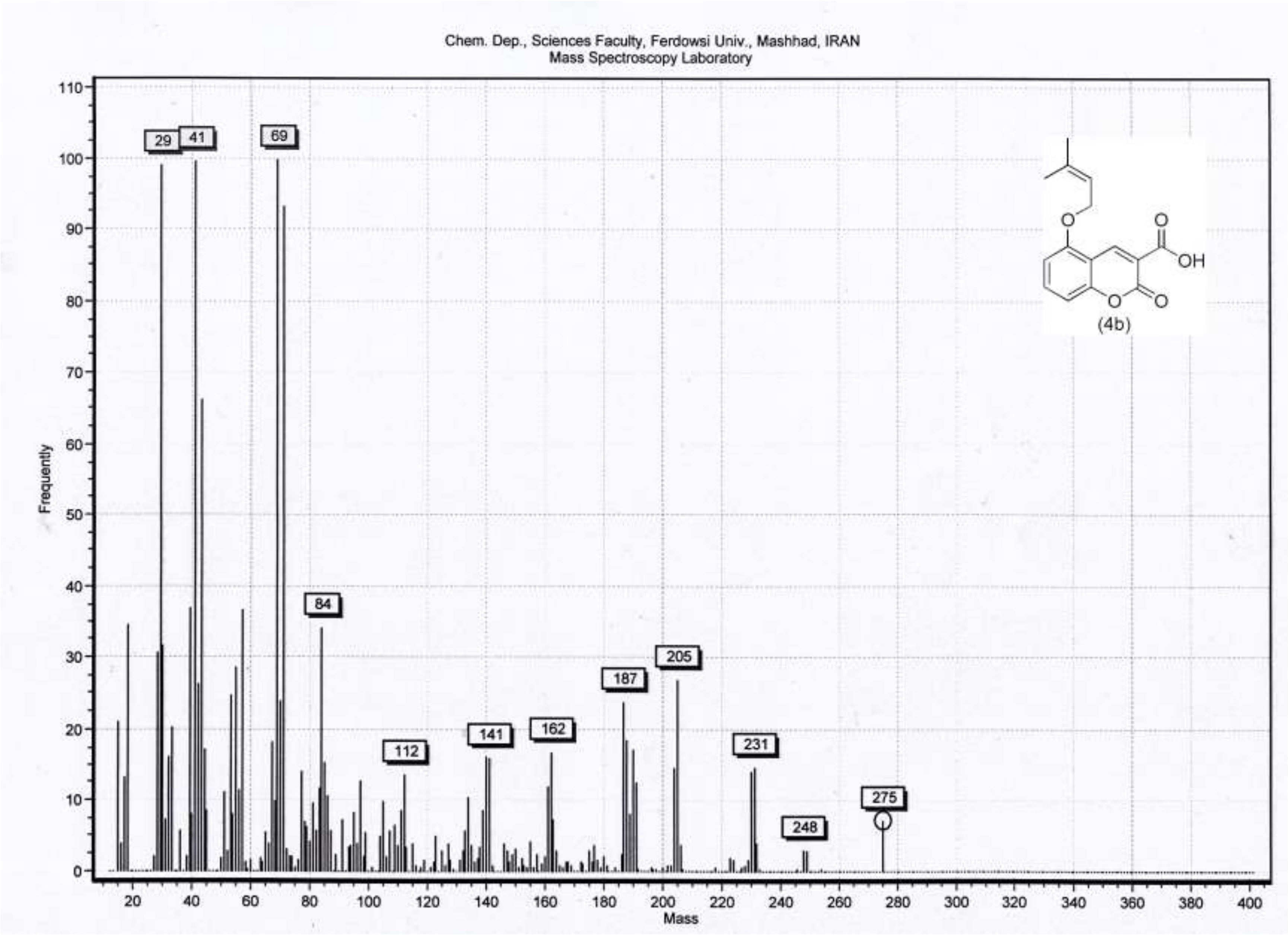
Chem. Dep., Sciences Faculty, Ferdowsi Univ., Mashhad, IRAN
Mass Spectroscopy Laboratory
Frequently
Mass
29
41
69
84
112
141
162
187
205
231
248
275
OH
(4b)

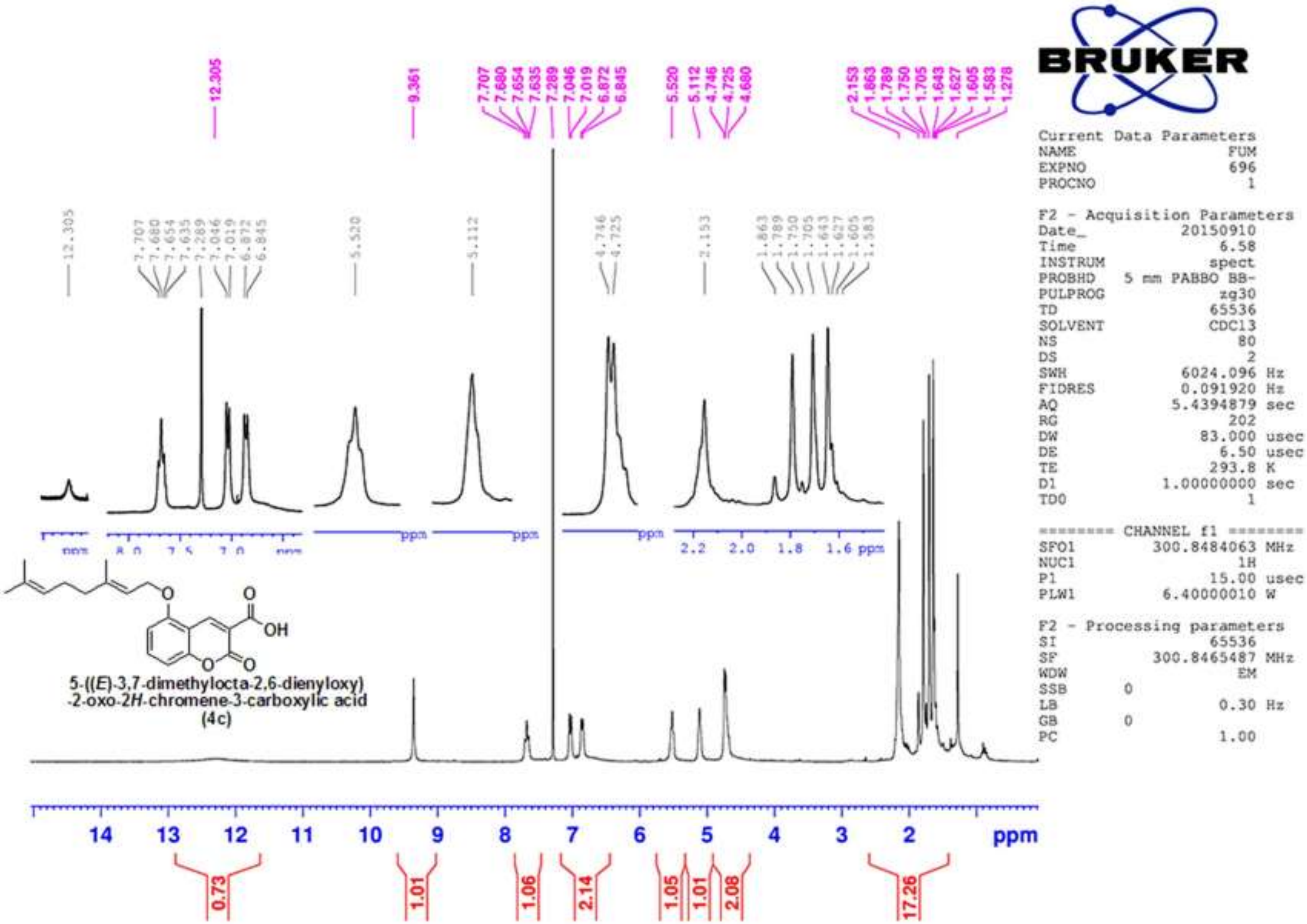

5-((*E*)-3,7-dimethylocta-2,6-dienyloxy)-2-oxo-2*H*-chromene-3-carboxylic acid (4c)

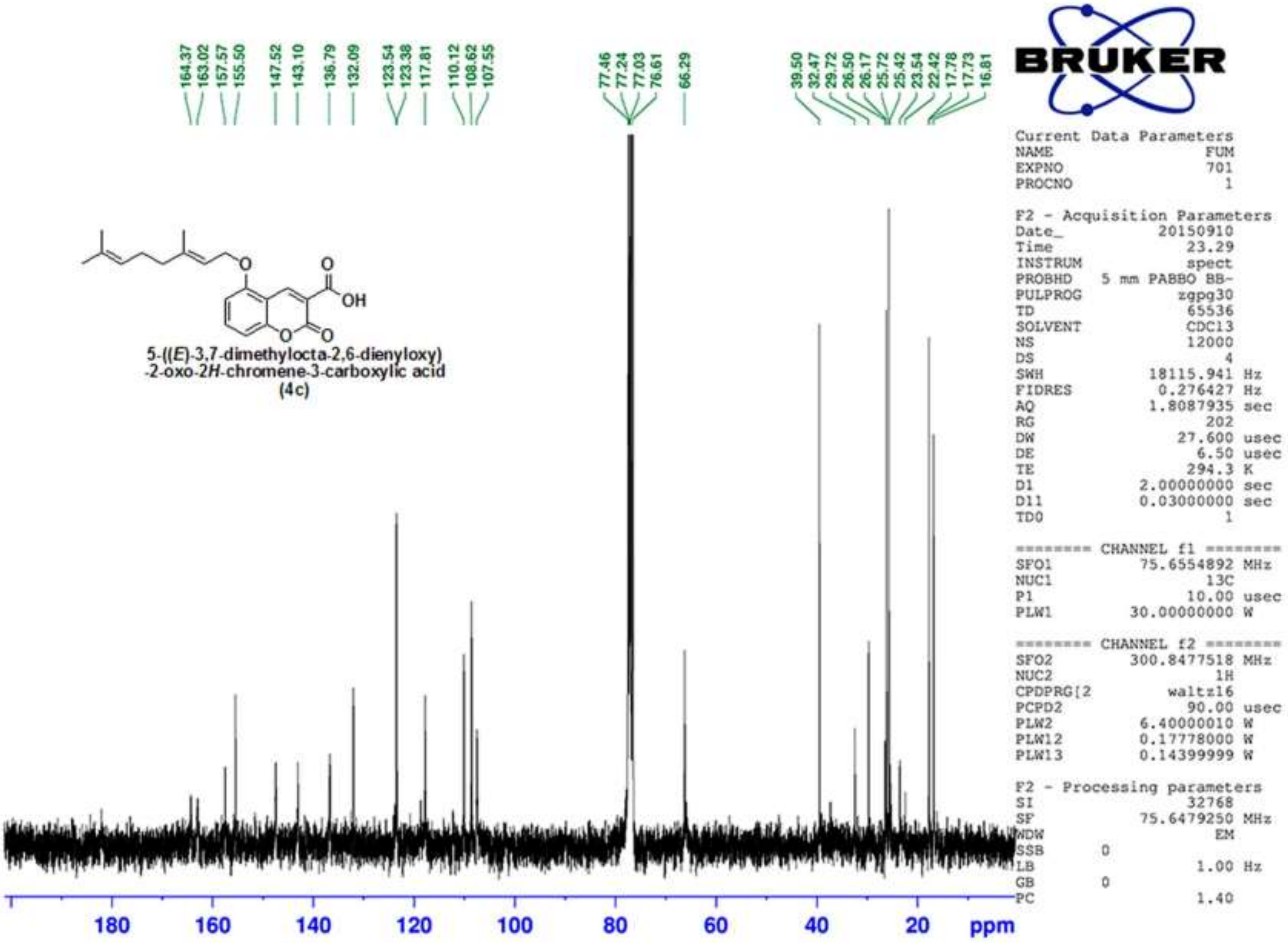
164.37
163.02
157.57
155.50
147.52
143.10
136.79
132.09
123.54
123.38
117.81
110.12
108.62
107.55
77.46
77.24
77.03
76.61
66.29
39.50
32.47
29.72
26.50
26.17
25.72
25.42
23.54
22.42
17.78
17.73
16.81
O
O
OH
O
O
5-((E)-3,7-dimethylocta-2,6-dienyloxy)
-2-oxo-2H-chromene-3-carboxylic acid
(4c)
BRUKER
Current Data Parameters
NAME FUM
EXPNO 701
PROCNO 1
F2 - Acquisition Parameters
Date_ 20150910
Time 23.29
INSTRUM spect
PROBHD 5 mm PABBO BB-
PULPROG zgpg30
TD 65536
SOLVENT CDCl3
NS 12000
DS 4
SWH 18115.941 Hz
FIDRES 0.276427 Hz
AQ 1.8087935 sec
RG 202
DW 27.600 usec
DE 6.50 usec
TE 294.3 K
D1 2.00000000 sec
D11 0.03000000 sec
TD0 1
======== CHANNEL f1 ========
SFO1 75.6554892 MHz
NUC1 13C
P1 10.00 usec
PLW1 30.00000000 W
======== CHANNEL f2 ========
SFO2 300.8477518 MHz
NUC2 1H
CPDPRG[2 waltz16
PCPD2 90.00 usec
PLW2 6.40000010 W
PLW12 0.17778000 W
PLW13 0.14399999 W
F2 - Processing parameters
SI 32768
SF 75.6479250 MHz
WDW EM
SSB 0
LB 1.00 Hz
GB 0
PC 1.40
180
160
140
120
100
80
60
40
20
ppm

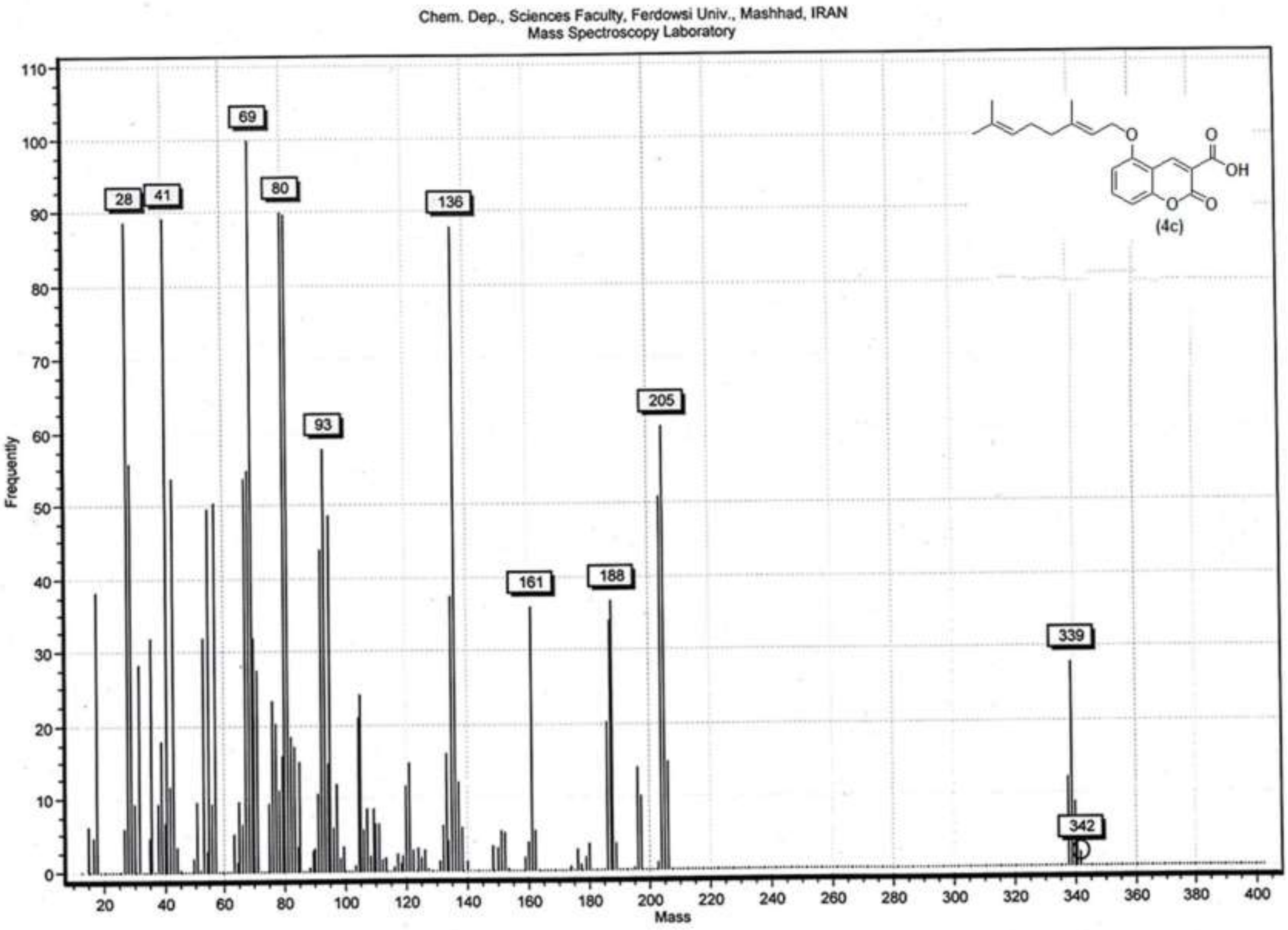
Chem. Dep., Sciences Faculty, Ferdowsi Univ., Mashhad, IRAN
Mass Spectroscopy Laboratory
Frequently
Mass
69
28
41
80
136
93
205
161
188
339
342
OH
(4c)

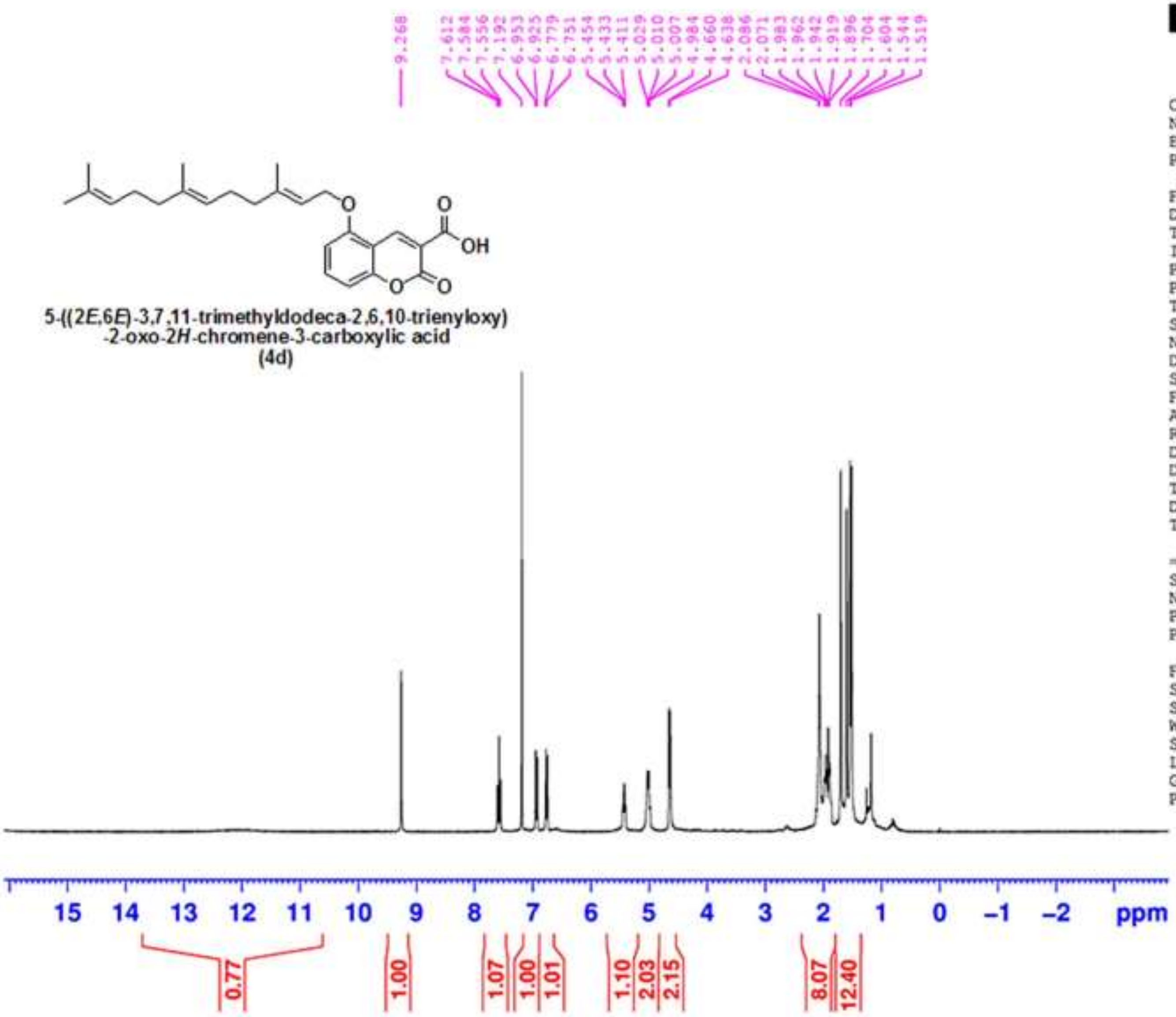

BRUKER

Current Data Parameters
NAME FUM
EXPNO 235
PROCNO 1

F2 - Acquisition Parameters
Date_ 20150621
Time 6.13
INSTRUM spect
PROBHD 5 mm DUL 13C-1
PULPROG zg30
TD 65536
SOLVENT CDCl3
NS 16
DS 2
SWH 6024.096 Hz
FIDRES 0.091920 Hz
AQ 5.4394879 sec
RG 202
DW 83.000 usec
DE 6.50 usec
TE 298.4 K
D1 1.00000000 sec
TD0 1

======== CHANNEL f1 ========
SFO1 300.8484063 MHz
NUC1 1H
P1 15.00 usec
PLW1 6.19999981 W

F2 - Processing parameters
SI 65536
SF 300.8465779 MHz
WDW EM
SSB 0
LB 0.30 Hz
GB 0
PC 1.00

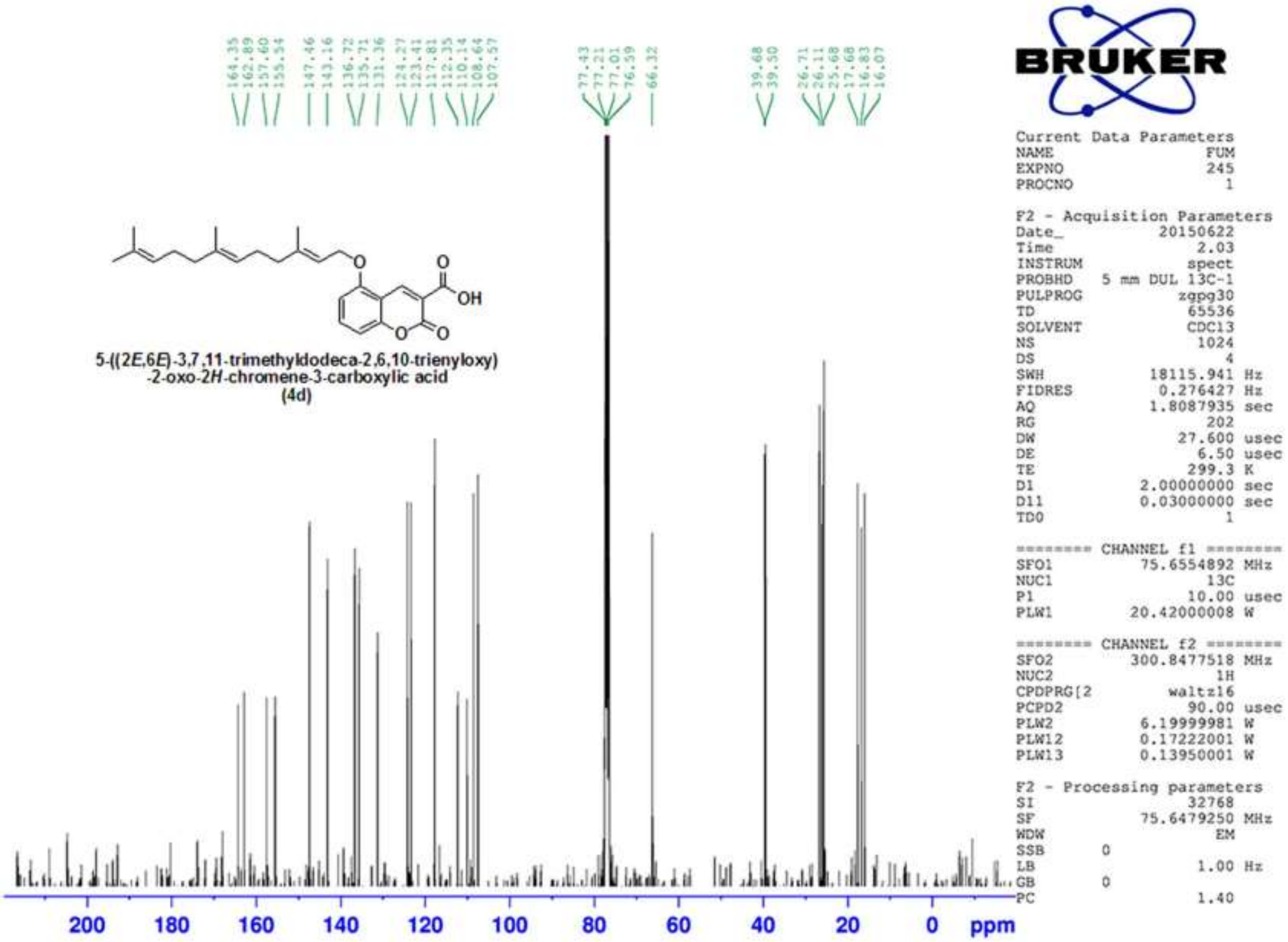
5-((2E,6E)-3,7,11-trimethyldodeca-2,6,10-trienyloxy)
-2-oxo-2H-chromene-3-carboxylic acid
(4d)
BRUKER
Current Data Parameters
NAME FUM
EXPNO 245
PROCNO 1
F2 - Acquisition Parameters
Date_ 20150622
Time 2.03
INSTRUM spect
PROBHD 5 mm DUL 13C-1
PULPROG zgpg30
TD 65536
SOLVENT CDCl3
NS 1024
DS 4
SWH 18115.941 Hz
FIDRES 0.276427 Hz
AQ 1.8087935 sec
RG 202
DW 27.600 usec
DE 6.50 usec
TE 299.3 K
D1 2.00000000 sec
D11 0.03000000 sec
TD0 1
======== CHANNEL f1 ========
SFO1 75.6554892 MHz
NUC1 13C
P1 10.00 usec
PLW1 20.42000008 W
======== CHANNEL f2 ========
SFO2 300.8477518 MHz
NUC2 1H
CPDPRG[2 waltz16
PCPD2 90.00 usec
PLW2 6.19999981 W
PLW12 0.17222001 W
PLW13 0.13950001 W
F2 - Processing parameters
SI 32768
SF 75.6479250 MHz
WDW EM
SSB 0
LB 1.00 Hz
GB 0
PC 1.40
164.35 162.89 157.60 155.54 147.46 143.16 136.72 135.71 131.36 124.27 123.41 117.81 112.35 110.14 108.64 107.57
77.43 77.21 77.01 76.59 66.32
39.68 39.50 26.71 26.11 25.68 17.68 16.83 16.07
200 180 160 140 120 100 80 60 40 20 0 ppm

Chem. Dep., Sciences Faculty, Ferdowsi Univ., Mashhad, IRAN
Mass Spectroscopy Laboratory

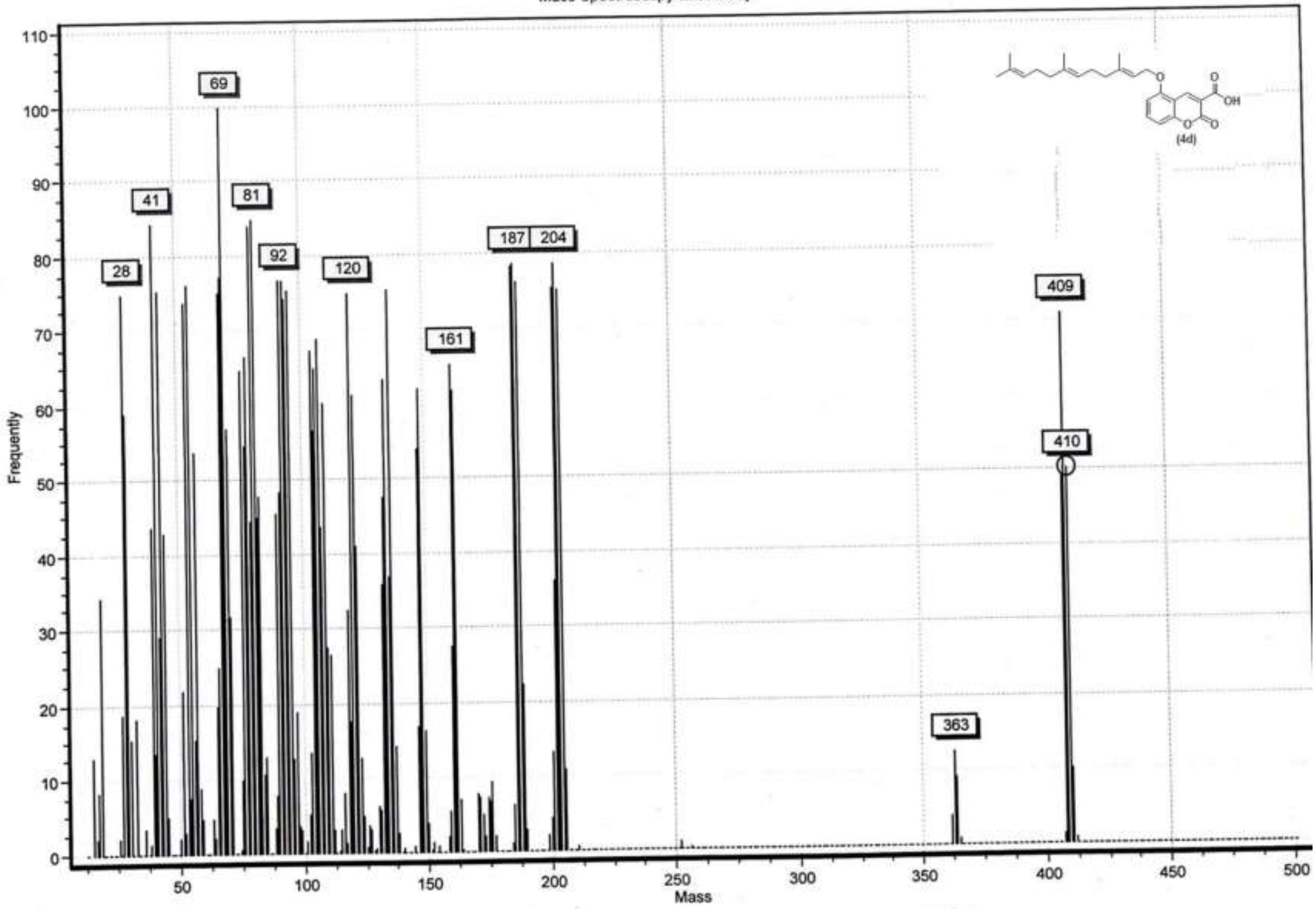

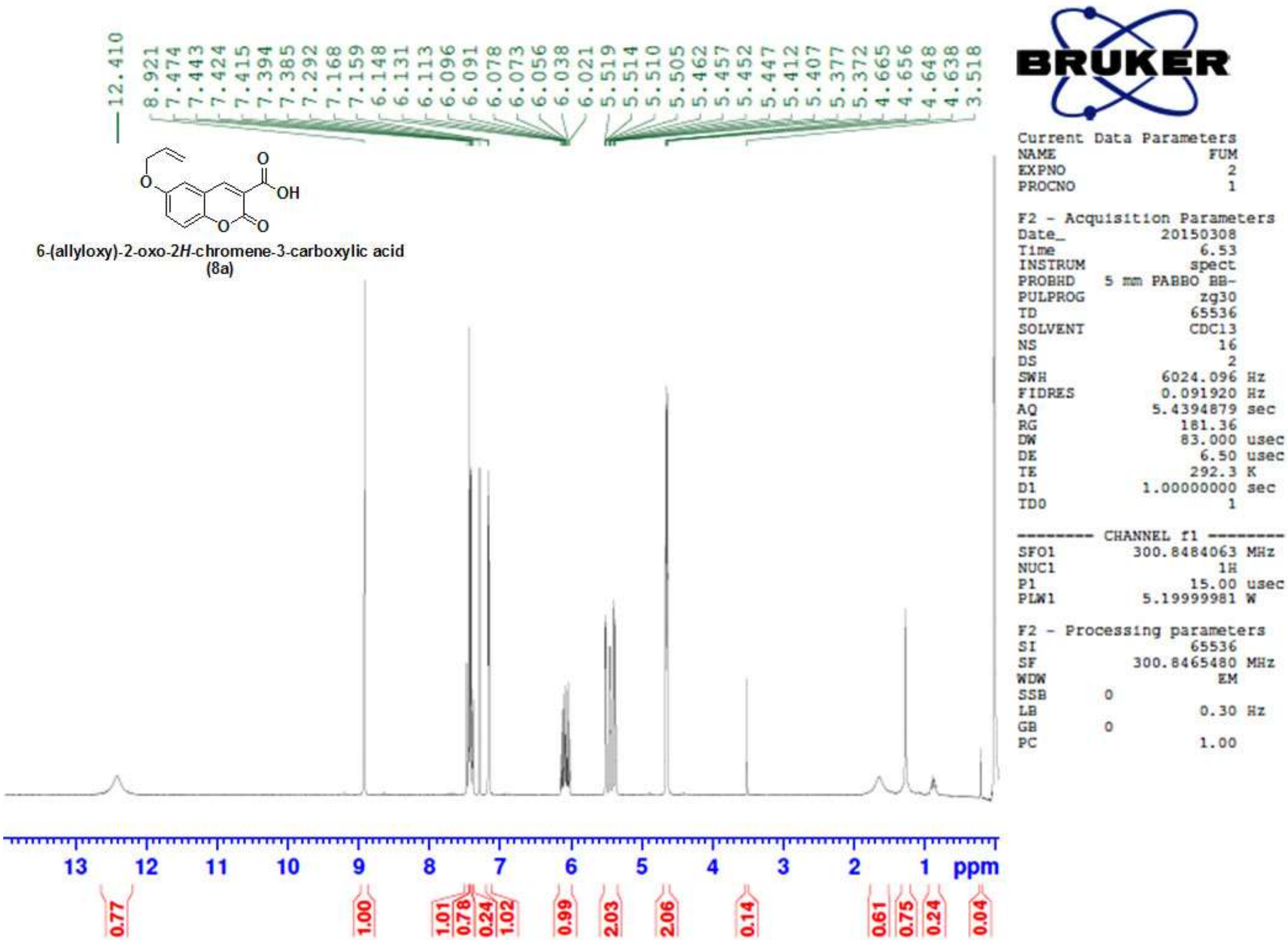

6-(allyloxy)-2-oxo-2*H*-chromene-3-carboxylic acid
(8a)

6-(allyloxy)-2-oxo-2*H*-chromene-3-carboxylic acid
(8a)

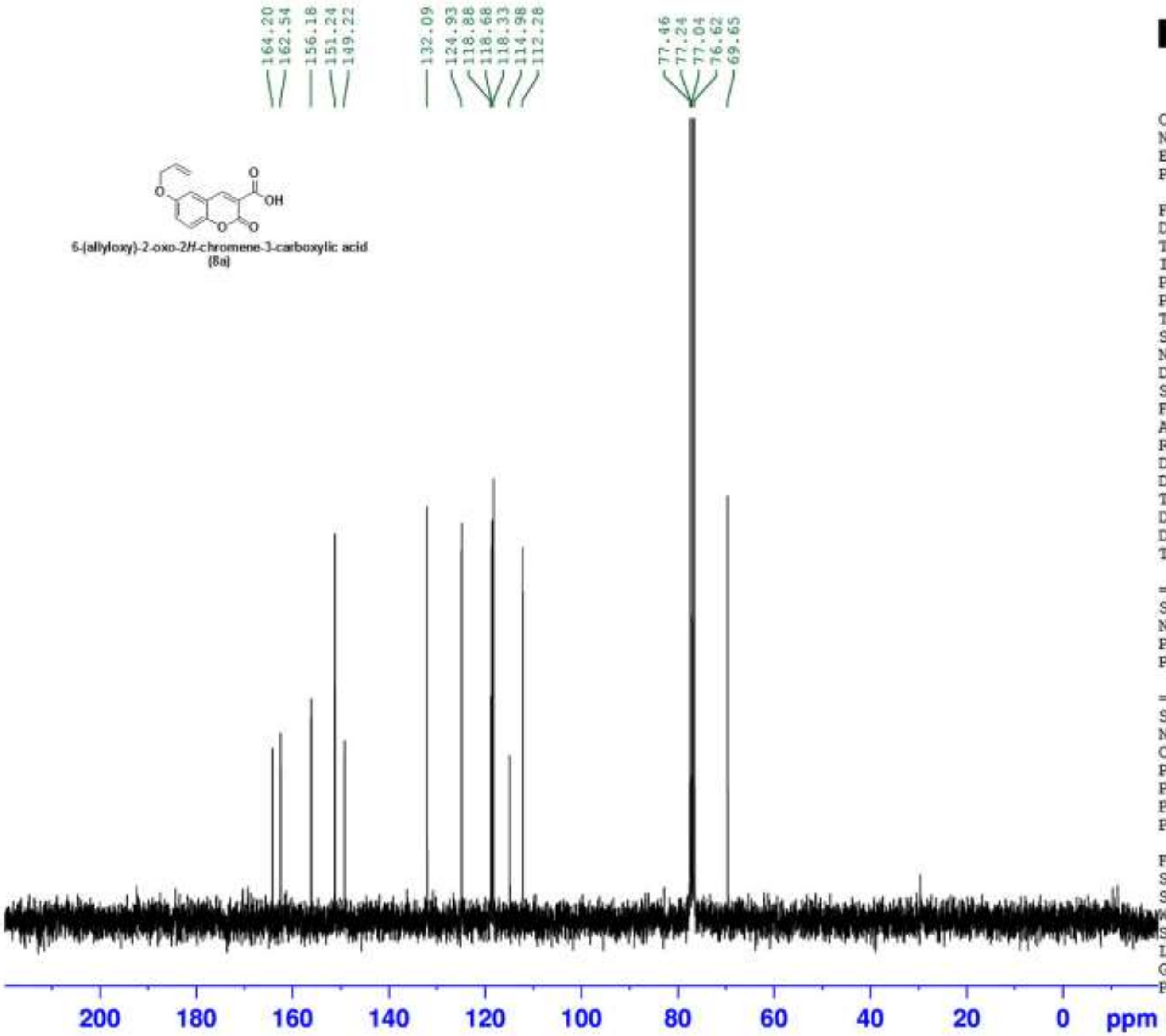

Current Data Parameters
NAME FUM
EXPNO 1165
PROCNO 1

F2 - Acquisition Parameters
Date_ 20150719
Time 9.12
INSTRUM spect
PROBHD 5 mm DUL 13C-1
PULPROG zgpg30
TD 65536
SOLVENT CDCl3
NS 256
DS 4
SWH 18115.941 Hz
FIDRES 0.276427 Hz
AQ 1.8087935 sec
RG 202
DW 27.600 usec
DE 6.50 usec
TE 296.0 K
D1 2.00000000 sec
D11 0.03000000 sec
TD0 1

======== CHANNEL f1 ========
SFO1 75.6554892 MHz
NUC1 13C
P1 10.00 usec
PLW1 20.42000008 W

======== CHANNEL f2 ========
SFO2 300.8477518 MHz
NUC2 1H
CPDPRG[2 waltz16
PCPD2 90.00 usec
PLW2 6.19999981 W
PLW12 0.17222001 W
PLW13 0.13950001 W

F2 - Processing parameters
SI 32768
SF 75.6479250 MHz
WDW EM
SSB 0
LB 1.00 Hz
GB 0
PC 1.40

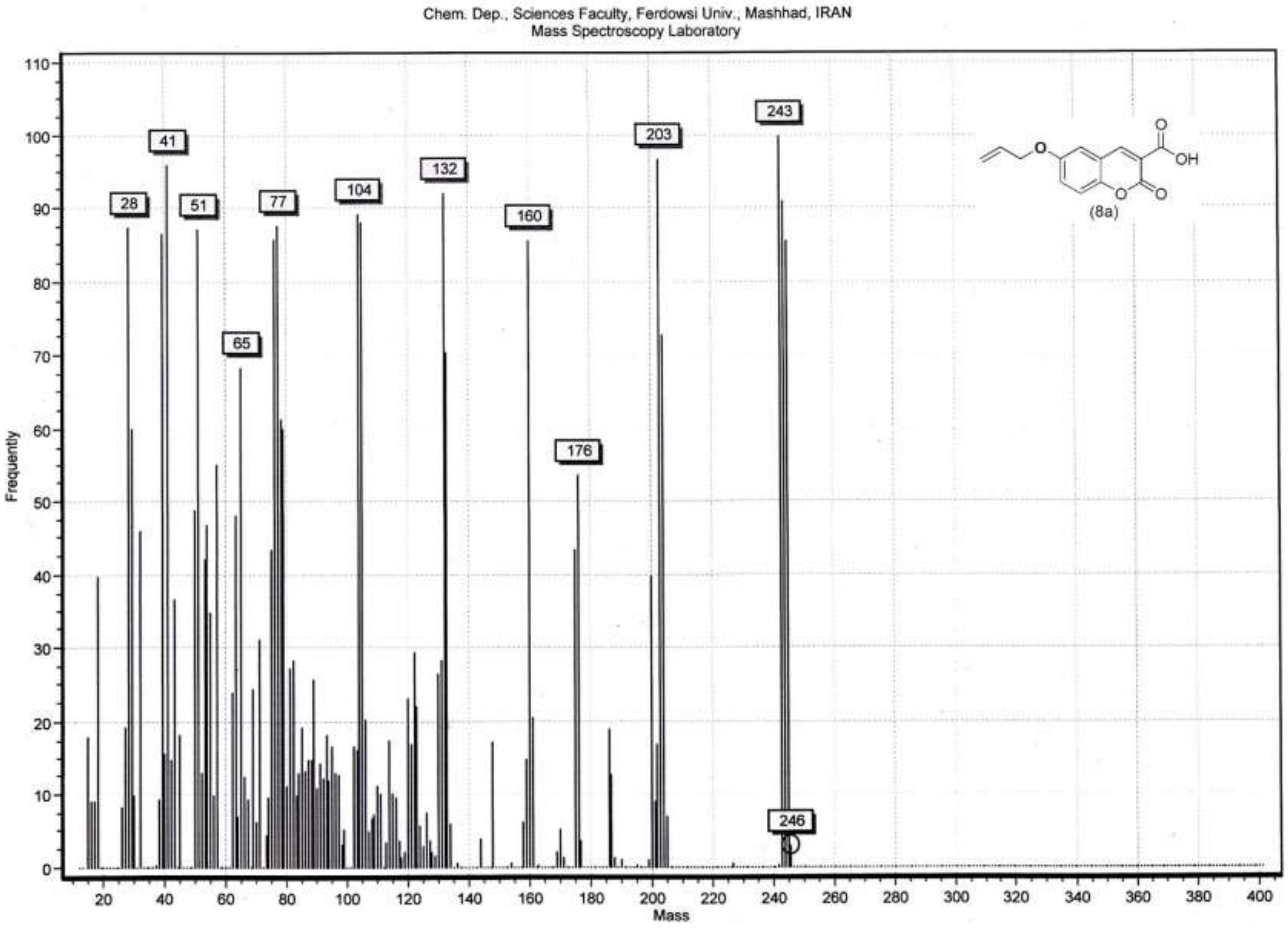
Chem. Dep., Sciences Faculty, Ferdowsi Univ., Mashhad, IRAN
Mass Spectroscopy Laboratory
Frequently
Mass
28
41
51
65
77
104
132
160
176
203
243
246
O
OH
(8a)

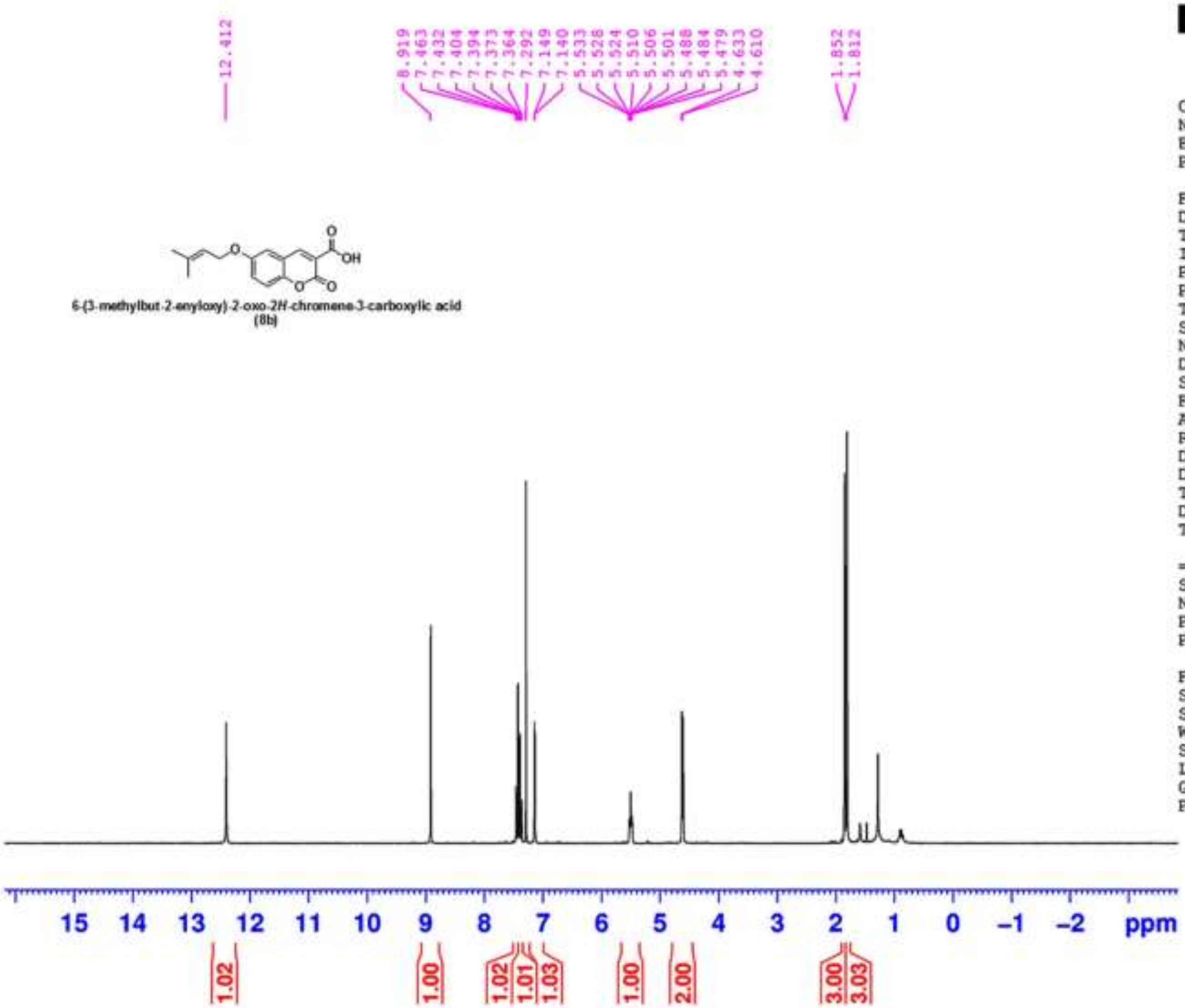

Current Data Parameters
NAME FUM
EXPNO 228
PROCNO 1

F2 - Acquisition Parameters
Date_ 20150621
Time 5.15
INSTRUM spect
PROBHD 5 mm DUL 13C-1
PULPROG zg30
TD 65536
SOLVENT CDCl3
NS 16
DS 2
SWH 6024.096 Hz
FIDRES 0.091920 Hz
AQ 5.4394879 sec
RG 202
DW 83.000 usec
DE 6.50 usec
TE 298.4 K
D1 1.00000000 sec
TD0 1

======== CHANNEL f1 ========
SFO1 300.8484063 MHz
NUC1 1H
P1 15.00 usec
PLW1 6.19999981 W

F2 - Processing parameters
SI 65536
SF 300.8465480 MHz
WDW EM
SSB 0
LB 0.30 Hz
GB 0
PC 1.00

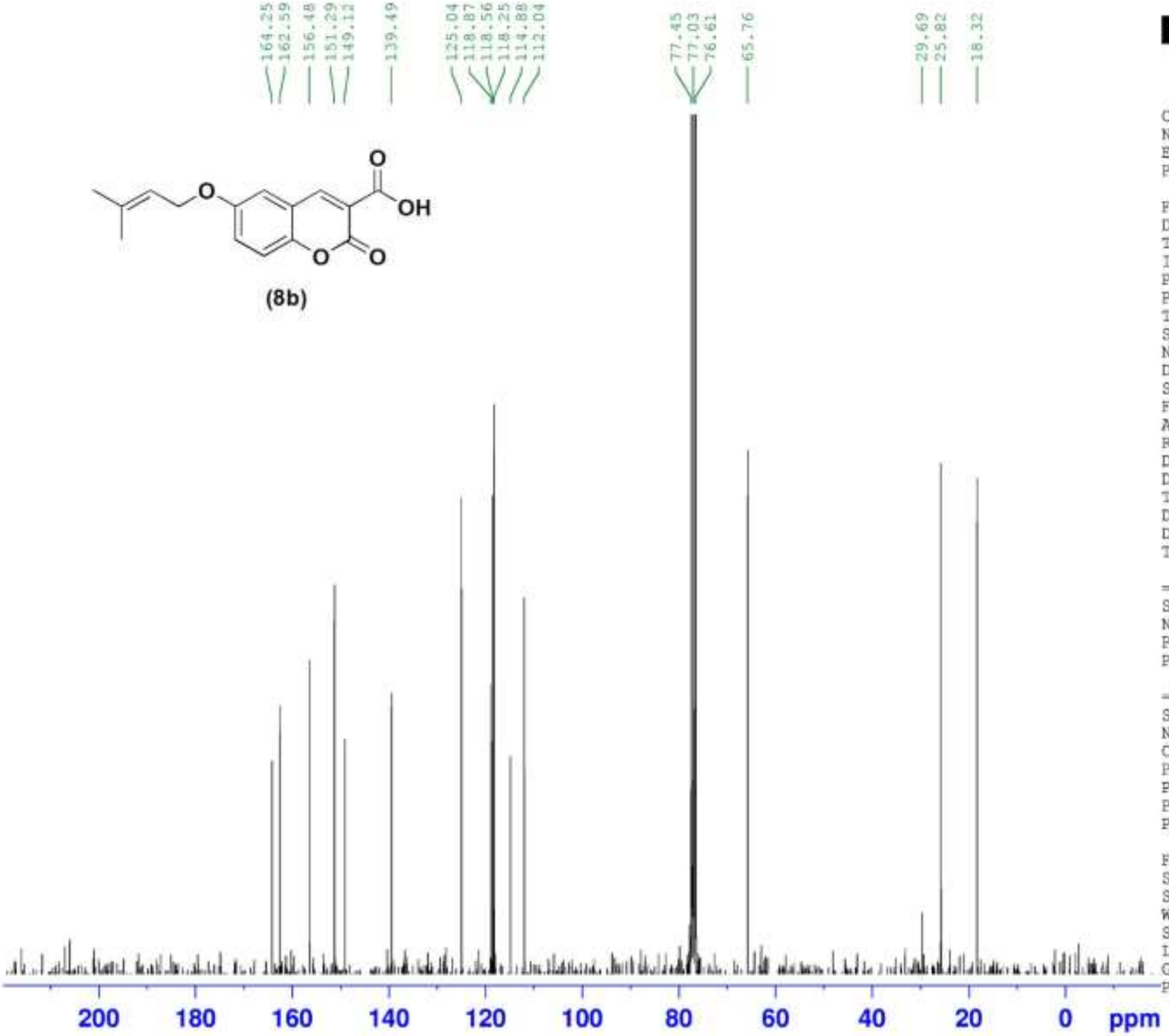


```
Current Data Parameters
NAME              FUM
EXPNO             238
PROCNO              1

F2 - Acquisition Parameters
Date_         20150621
Time              8.34
INSTRUM          spect
PROBHD   5 mm DUL 13C-1
PULPROG         zgpg30
TD               65536
SOLVENT          CDCl3
NS                 512
DS                   4
SWH          18115.941 Hz
FIDRES        0.276427 Hz
AQ           1.8087935 sec
RG                 202
DW              27.600 usec
DE                6.50 usec
TE               299.4 K
D1          2.00000000 sec
D11         0.03000000 sec
TD0                  1

======== CHANNEL f1 ========
SFO1        75.6554892 MHz
NUC1               13C
P1               10.00 usec
PLW1       20.42000008 W

======== CHANNEL f2 ========
SFO2       300.8477518 MHz
NUC2                1H
CPDPRG[2       waltz16
PCPD2            90.00 usec
PLW2        6.19999981 W
PLW12       0.17222001 W
PLW13       0.13950001 W

F2 - Processing parameters
SI               32768
SF          75.6479250 MHz
WDW                 EM
SSB      0
LB                1.00 Hz
GB       0
PC                1.40
```

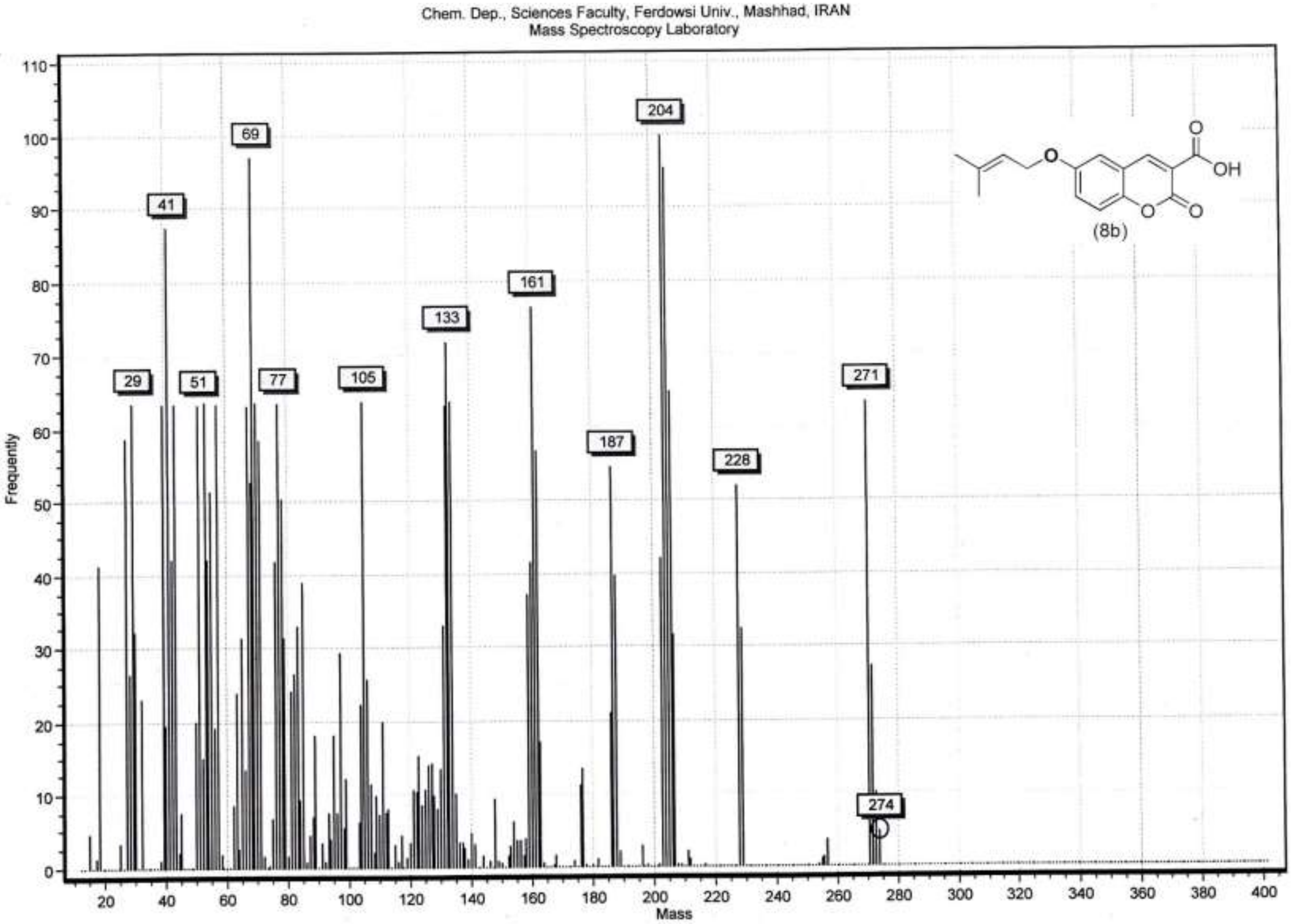
Chem. Dep., Sciences Faculty, Ferdowsi Univ., Mashhad, IRAN
Mass Spectroscopy Laboratory
Frequently
Mass
29
41
51
69
77
105
133
161
187
204
228
271
274
(8b)

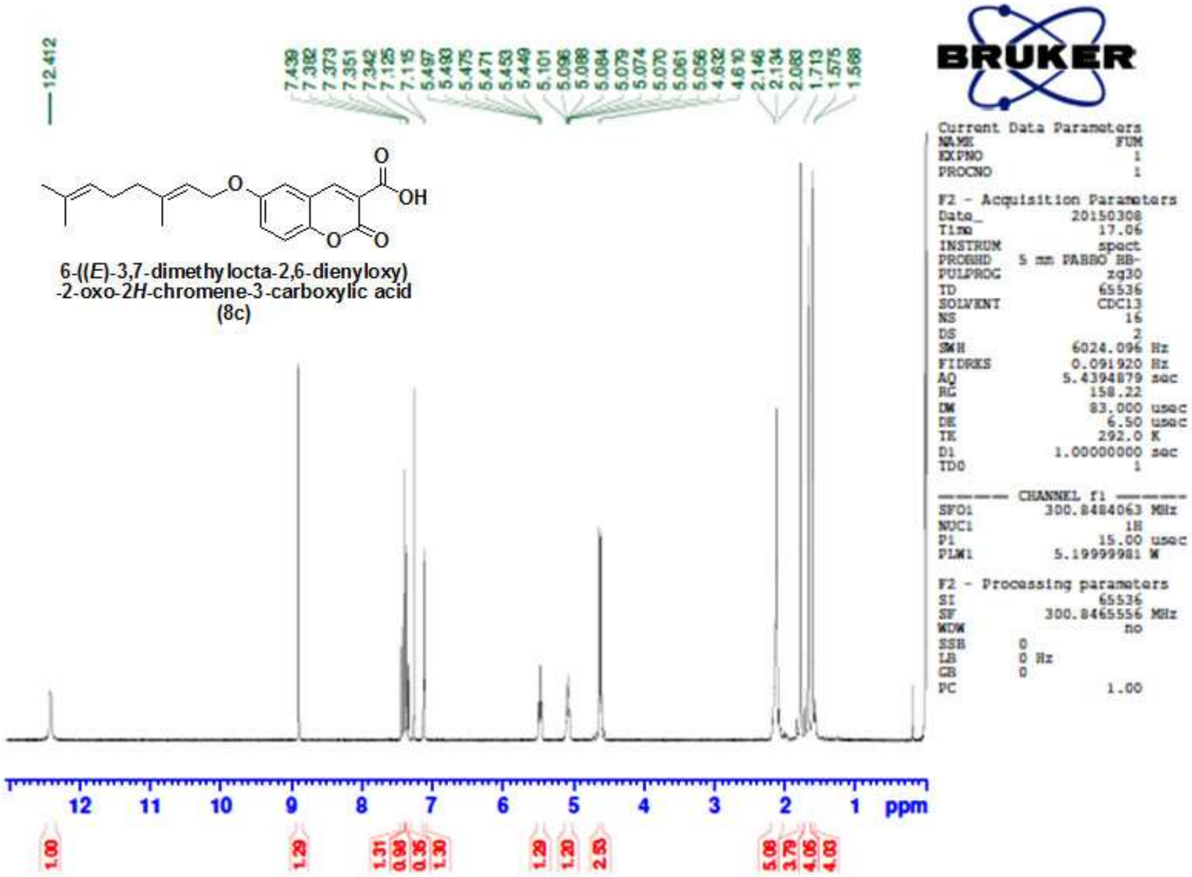

12.412
7.439
7.382
7.373
7.351
7.342
7.125
7.115
5.497
5.493
5.475
5.471
5.453
5.449
5.101
5.096
5.088
5.084
5.079
5.074
5.070
5.061
5.056
4.632
4.610
2.146
2.134
2.083
1.713
1.575
1.568
O
OH
O
O
O
6-((E)-3,7-dimethylocta-2,6-dienyloxy)
-2-oxo-2H-chromene-3-carboxylic acid
(8c)
BRUKER
Current Data Parameters
NAME FUM
EXPNO 1
PROCNO 1
F2 - Acquisition Parameters
Date_ 20150308
Time 17.06
INSTRUM spect
PROBHD 5 mm PABBO BB-
PULPROG zg30
TD 65536
SOLVENT CDCl3
NS 16
DS 2
SWH 6024.096 Hz
FIDRES 0.091920 Hz
AQ 5.4394879 sec
RG 158.22
DW 83.000 usec
DE 6.50 usec
TE 292.0 K
D1 1.00000000 sec
TD0 1
CHANNEL f1
SFO1 300.8484063 MHz
NUC1 1H
P1 15.00 usec
PLW1 5.19999981 W
F2 - Processing parameters
SI 65536
SF 300.8465556 MHz
WDW no
SSB 0
LB 0 Hz
GB 0
PC 1.00
12
11
10
9
8
7
6
5
4
3
2
1
ppm
1.00
1.29
1.31
0.90
0.95
1.30
1.29
1.20
2.53
5.08
3.79
4.05
4.00

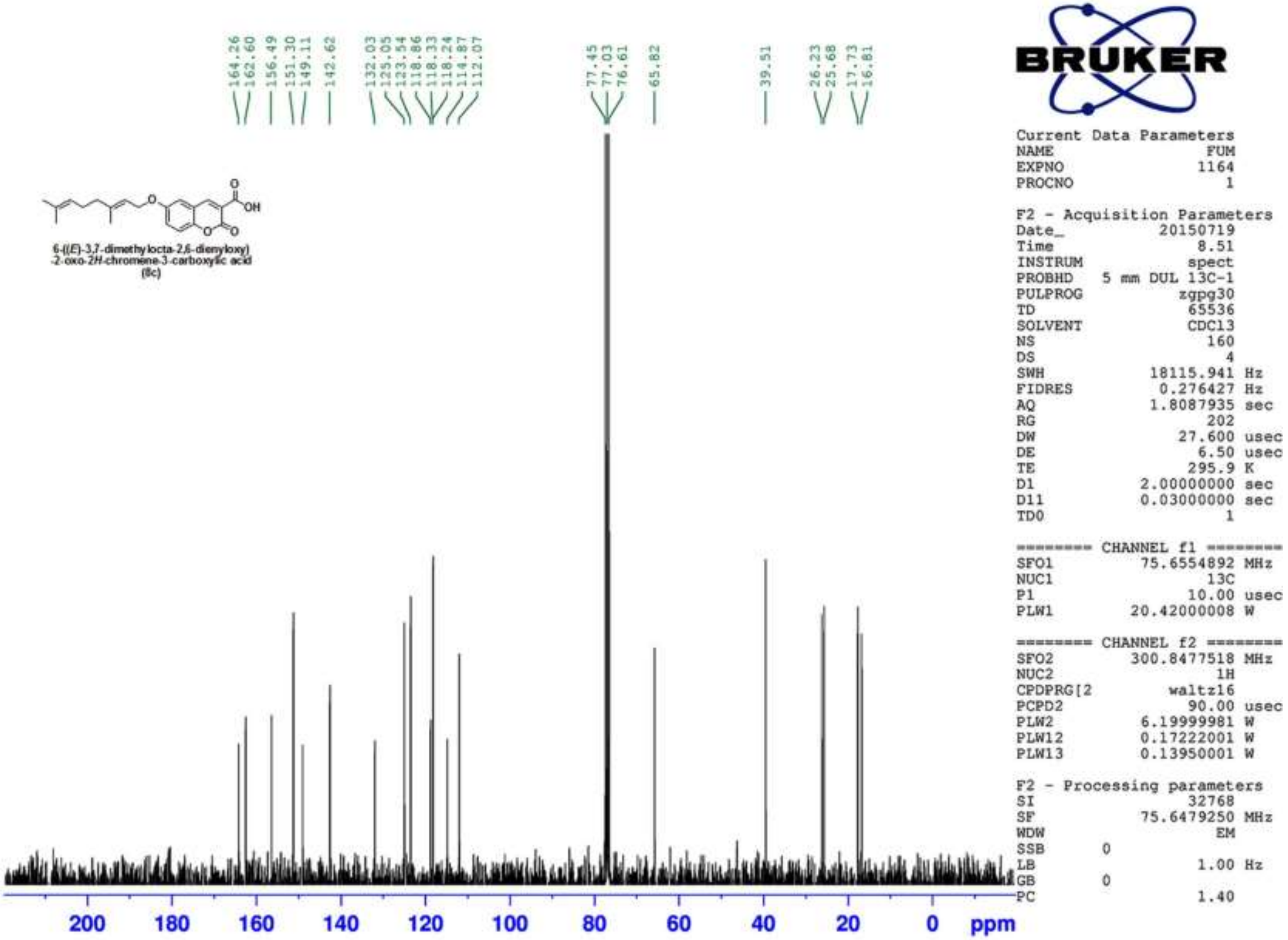

6-((*E*)-3,7-dimethylocta-2,6-dienyloxy)
-2-oxo-2*H*-chromene-3-carboxylic acid
(8c)

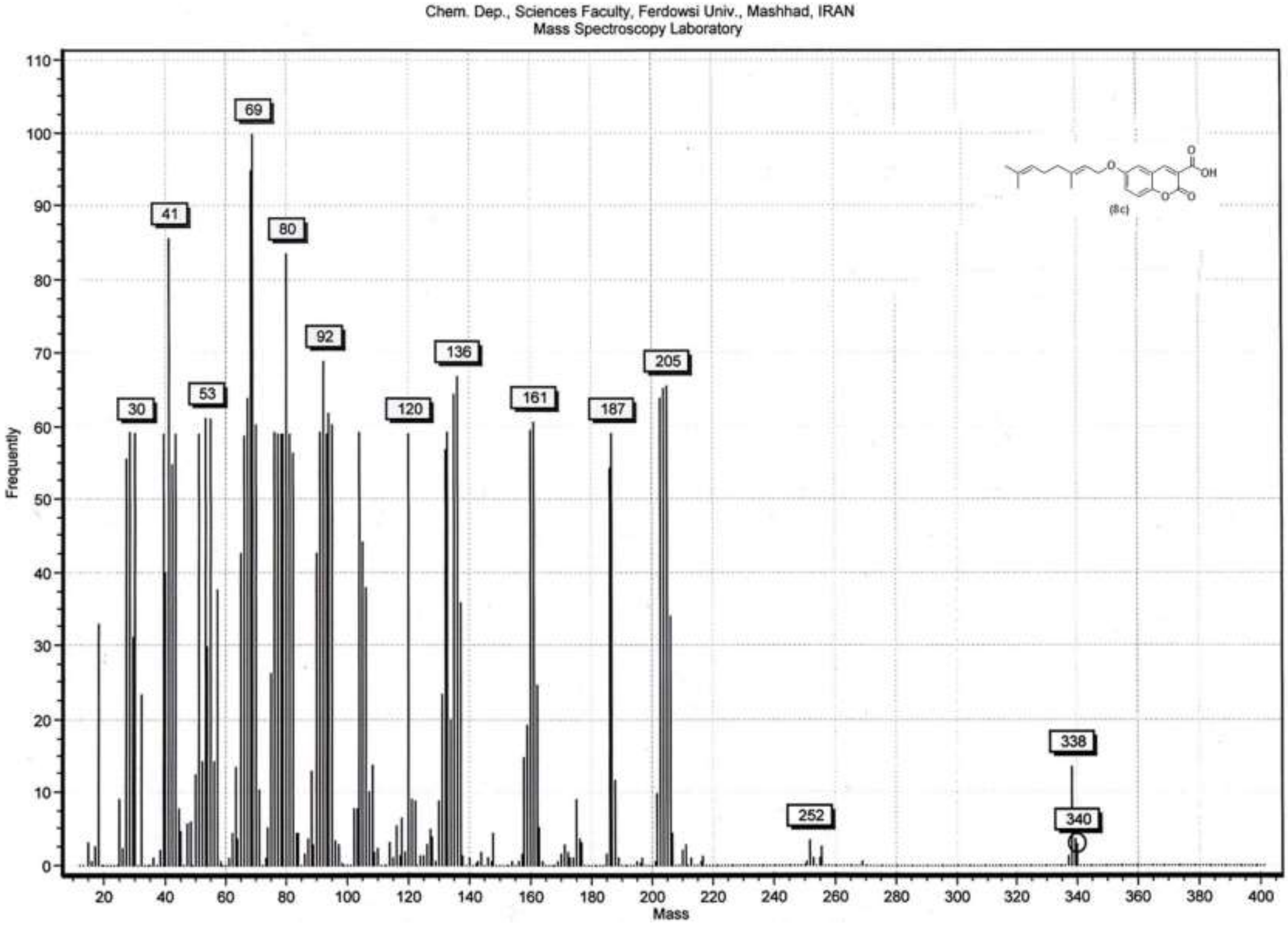

Chem. Dep., Sciences Faculty, Ferdowsi Univ., Mashhad, IRAN
Mass Spectroscopy Laboratory
Frequently
Mass
69
41
80
92
136
205
30
53
120
161
187
338
252
340
(8c)

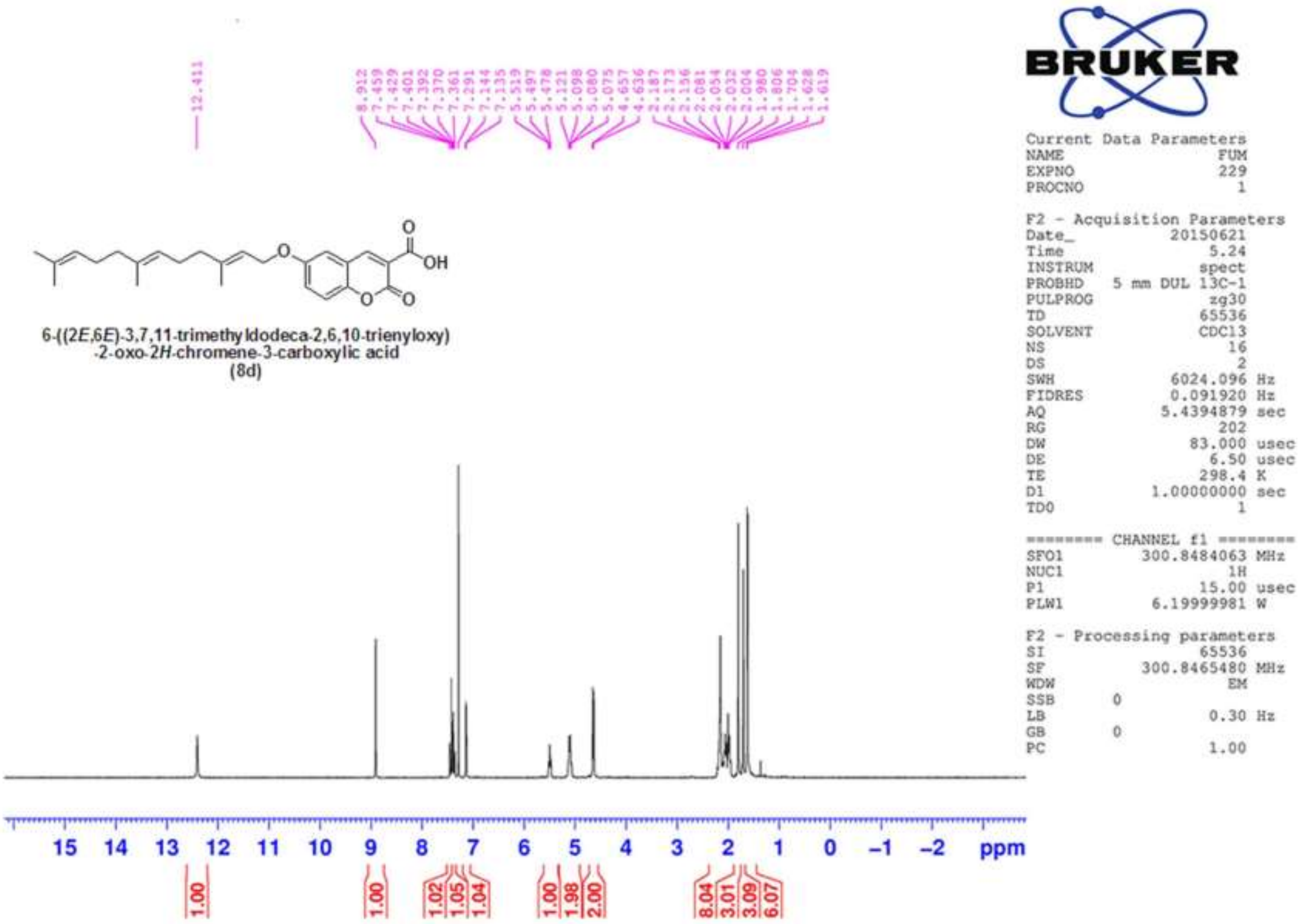

6-((2*E*,6*E*)-3,7,11-trimethyldodeca-2,6,10-trienyloxy)-2-oxo-2*H*-chromene-3-carboxylic acid
(8d)

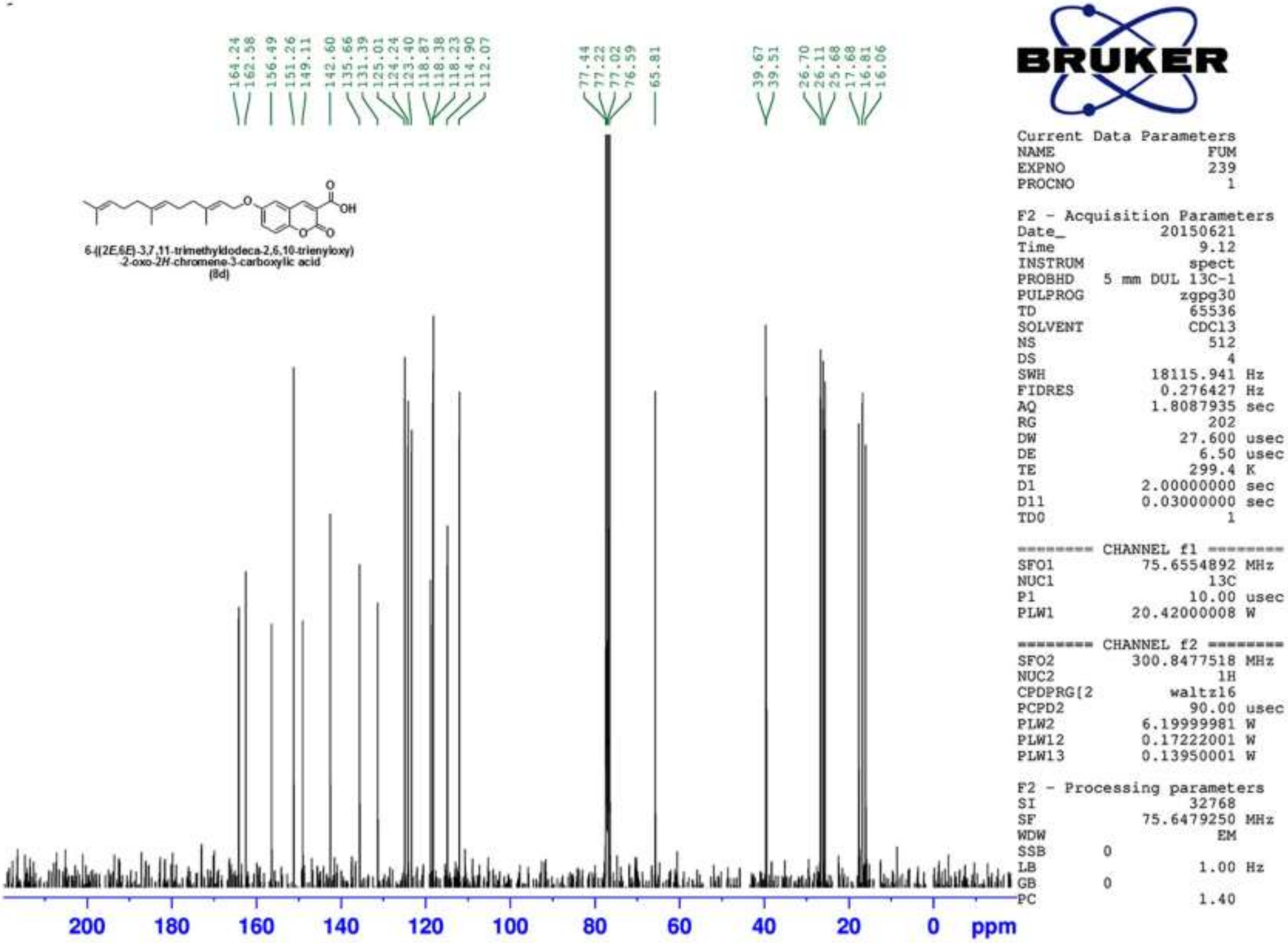
164.24
162.58
156.49
151.26
149.11
142.60
135.66
131.39
125.01
124.24
123.40
118.87
118.38
118.23
114.90
112.07
77.44
77.22
77.02
76.59
65.81
39.67
39.51
26.70
26.11
25.68
17.68
16.81
16.06
O
OH
O
O
O
6-((2E,6E)-3,7,11-trimethyldodeca-2,6,10-trienyloxy)
-2-oxo-2H-chromene-3-carboxylic acid
(8d)
200
180
160
140
120
100
80
60
40
20
0
ppm
BRUKER
Current Data Parameters
NAME FUM
EXPNO 239
PROCNO 1
F2 - Acquisition Parameters
Date_ 20150621
Time 9.12
INSTRUM spect
PROBHD 5 mm DUL 13C-1
PULPROG zgpg30
TD 65536
SOLVENT CDCl3
NS 512
DS 4
SWH 18115.941 Hz
FIDRES 0.276427 Hz
AQ 1.8087935 sec
RG 202
DW 27.600 usec
DE 6.50 usec
TE 299.4 K
D1 2.00000000 sec
D11 0.03000000 sec
TD0 1
======== CHANNEL f1 ========
SFO1 75.6554892 MHz
NUC1 13C
P1 10.00 usec
PLW1 20.42000008 W
======== CHANNEL f2 ========
SFO2 300.8477518 MHz
NUC2 1H
CPDPRG[2 waltz16
PCPD2 90.00 usec
PLW2 6.19999981 W
PLW12 0.17222001 W
PLW13 0.13950001 W
F2 - Processing parameters
SI 32768
SF 75.6479250 MHz
WDW EM
SSB 0
LB 1.00 Hz
GB 0
PC 1.40

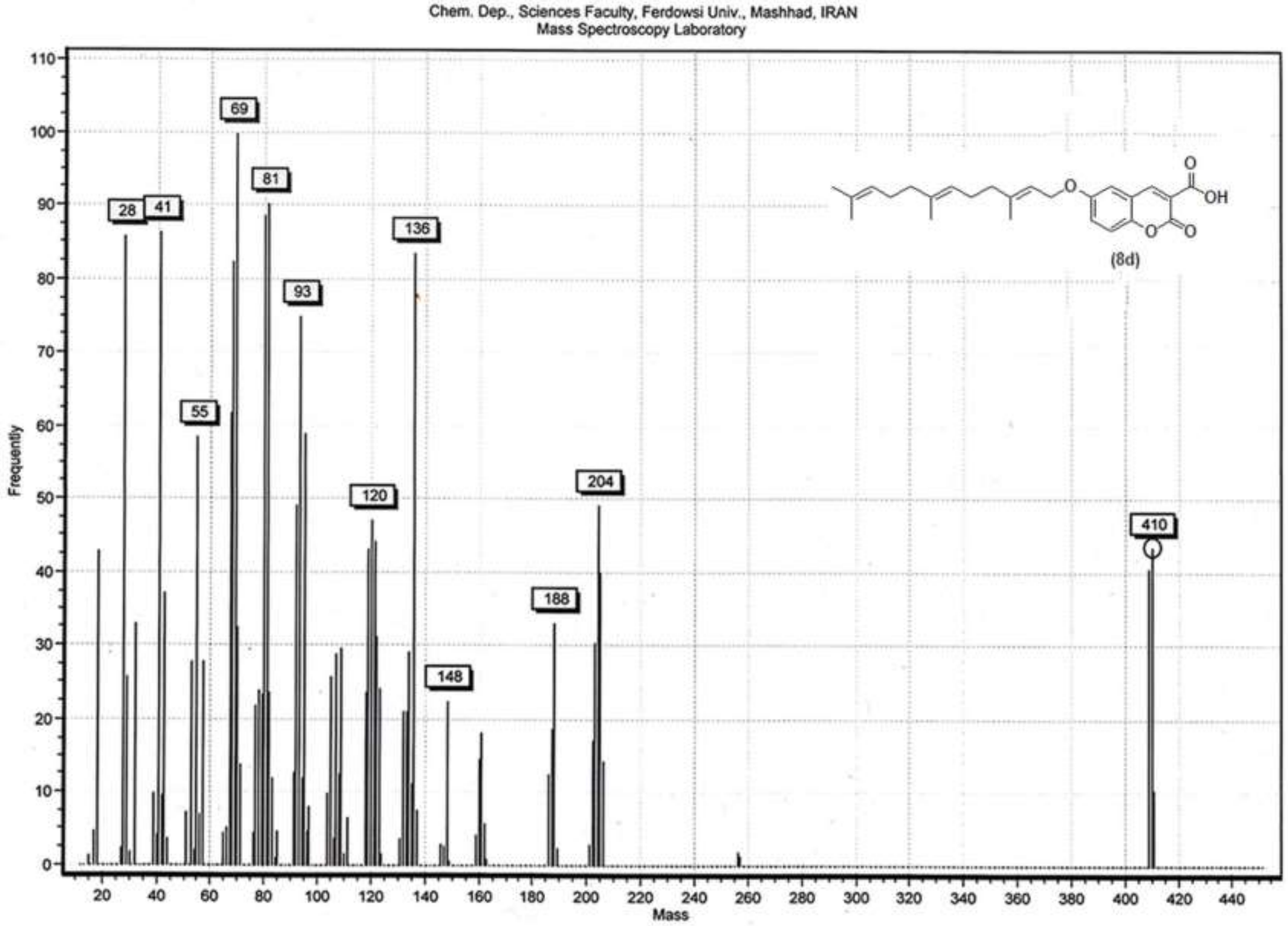
Chem. Dep., Sciences Faculty, Ferdowsi Univ., Mashhad, IRAN
Mass Spectroscopy Laboratory
Frequently
Mass
28
41
55
69
81
93
120
136
148
188
204
410
(8d)

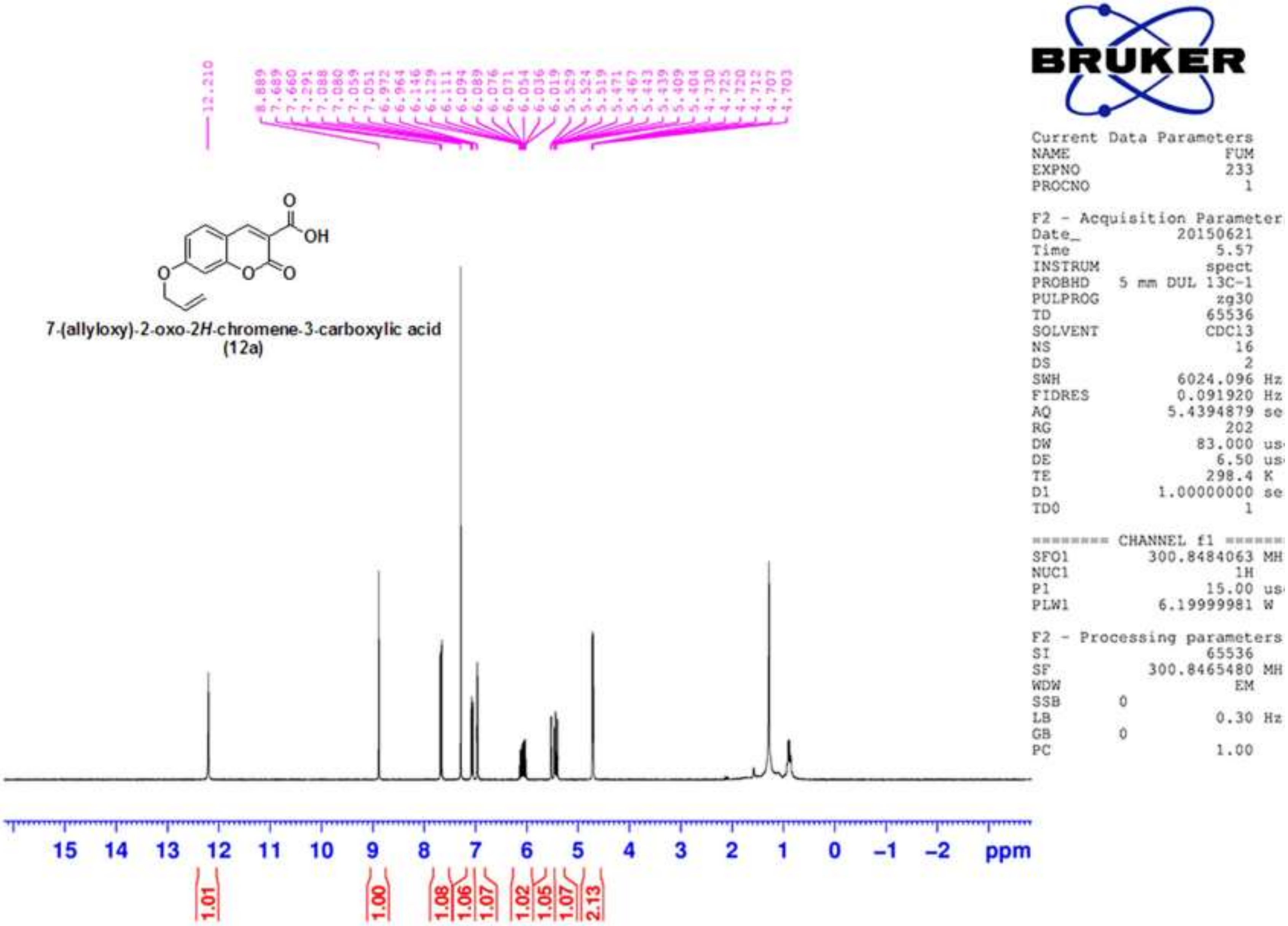
12.210
8.889
7.689
7.660
7.291
7.088
7.080
7.059
7.051
6.972
6.964
6.146
6.129
6.111
6.094
6.089
6.076
6.071
6.054
6.036
6.019
5.529
5.524
5.519
5.471
5.467
5.443
5.439
5.409
5.404
4.730
4.725
4.720
4.712
4.707
4.703
O
OH
O
O
O
7-(allyloxy)-2-oxo-2H-chromene-3-carboxylic acid
(12a)
BRUKER
Current Data Parameters
NAME FUM
EXPNO 233
PROCNO 1
F2 - Acquisition Parameter
Date_ 20150621
Time 5.57
INSTRUM spect
PROBHD 5 mm DUL 13C-1
PULPROG zg30
TD 65536
SOLVENT CDCl3
NS 16
DS 2
SWH 6024.096 Hz
FIDRES 0.091920 Hz
AQ 5.4394879 se
RG 202
DW 83.000 us
DE 6.50 us
TE 298.4 K
D1 1.00000000 se
TD0 1
======== CHANNEL f1 ======
SFO1 300.8484063 MH
NUC1 1H
P1 15.00 us
PLW1 6.19999981 W
F2 - Processing parameters
SI 65536
SF 300.8465480 MH
WDW EM
SSB 0
LB 0.30 Hz
GB 0
PC 1.00
15 14 13 12 11 10 9 8 7 6 5 4 3 2 1 0 -1 -2 ppm
1.01
1.00
1.08
1.06
1.07
1.02
1.05
1.07
2.13

7-(allyloxy)-2-oxo-2*H*-chromene-3-carboxylic acid
(12a)

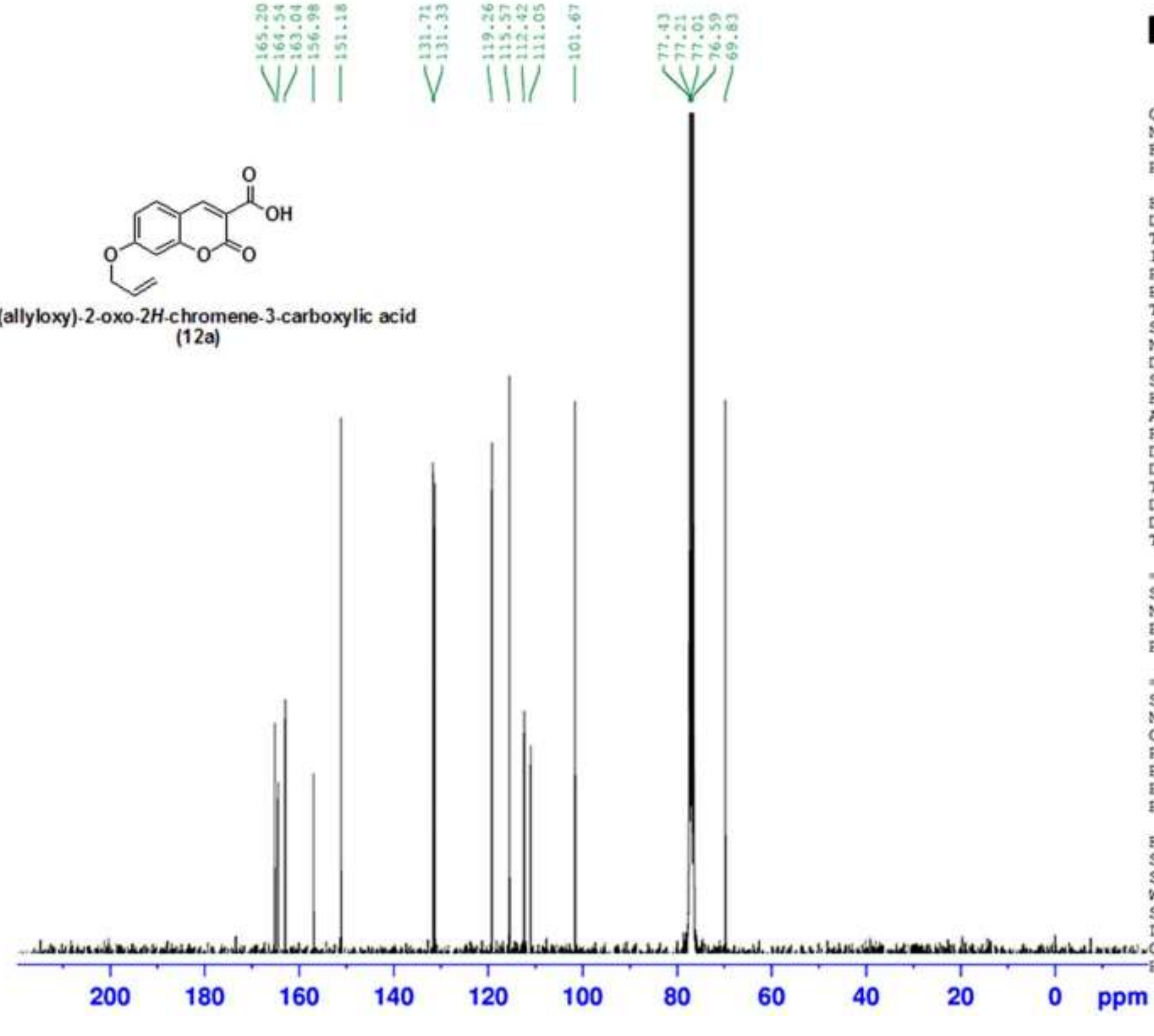

Current Data Parameters	
NAME	FUM
EXPNO	283
PROCNO	1

F2 - Acquisition Parameters	
Date_	20150623
Time	22.54
INSTRUM	spect
PROBHD	5 mm DUL 13C-1
PULPROG	zgpg30
TD	65536
SOLVENT	CDCl3
NS	14000
DS	4
SWH	18115.941 Hz
FIDRES	0.276427 Hz
AQ	1.8087935 sec
RG	202
DW	27.600 use
DE	6.50 use
TE	298.1 K
D1	2.00000000 sec
D11	0.03000000 sec
TD0	1

======== CHANNEL f1 =======	
SFO1	75.6554892 MHz
NUC1	13C
P1	10.00 use
PLW1	20.42000008 W

======== CHANNEL f2 =======	
SFO2	300.8477518 MHz
NUC2	1H
CPDPRG[2	waltz16
PCPD2	90.00 use
PLW2	6.19999981 W
PLW12	0.17222001 W
PLW13	0.13950001 W

F2 - Processing parameters	
SI	32768
SF	75.6479250 MHz
WDW	EM
SSB	0
LB	1.00 Hz
GB	0
PC	1.40

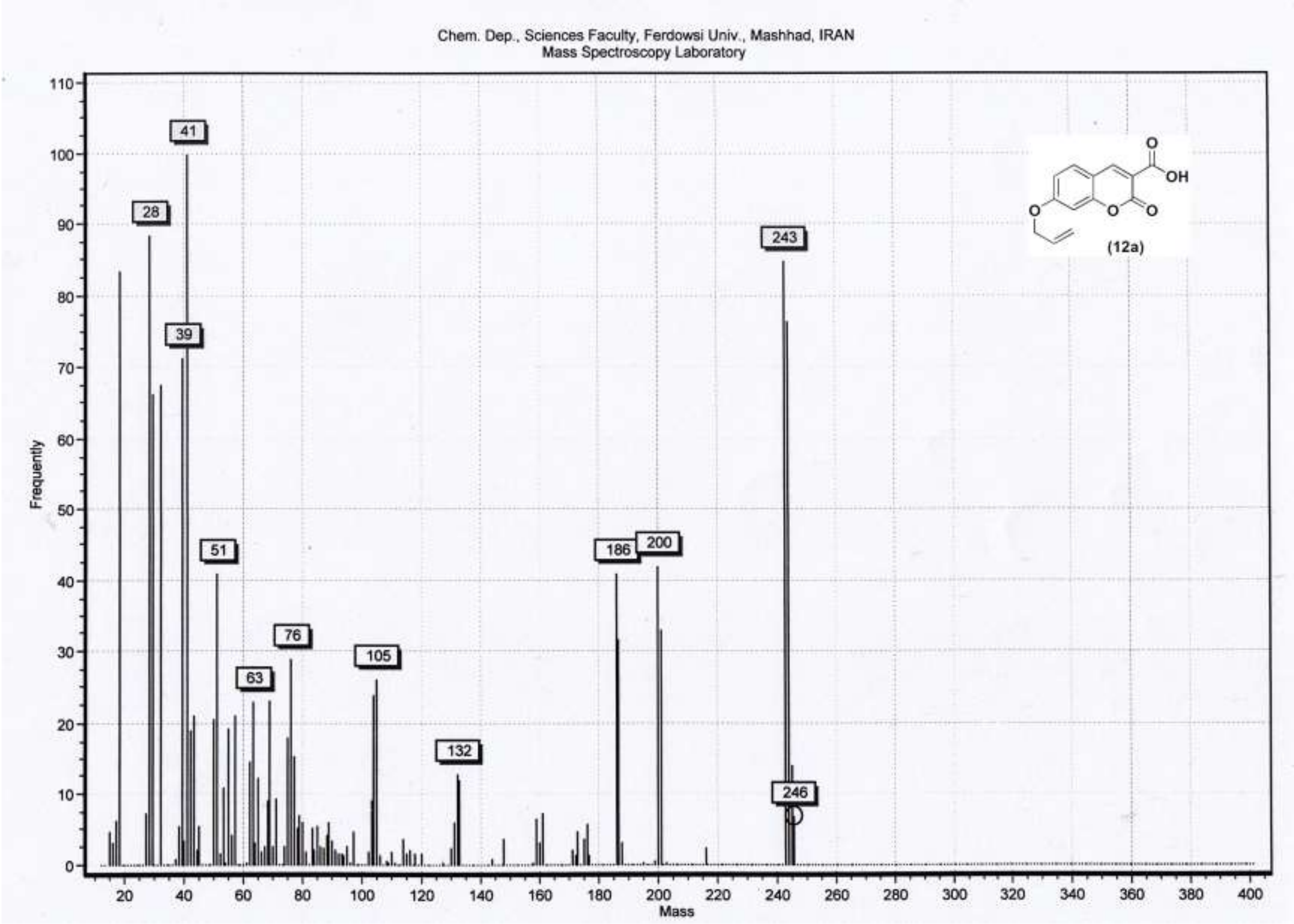

Chem. Dep., Sciences Faculty, Ferdowsi Univ., Mashhad, IRAN
Mass Spectroscopy Laboratory
Frequently
Mass
41
28
39
51
63
76
105
132
186
200
243
246
O
OH
(12a)

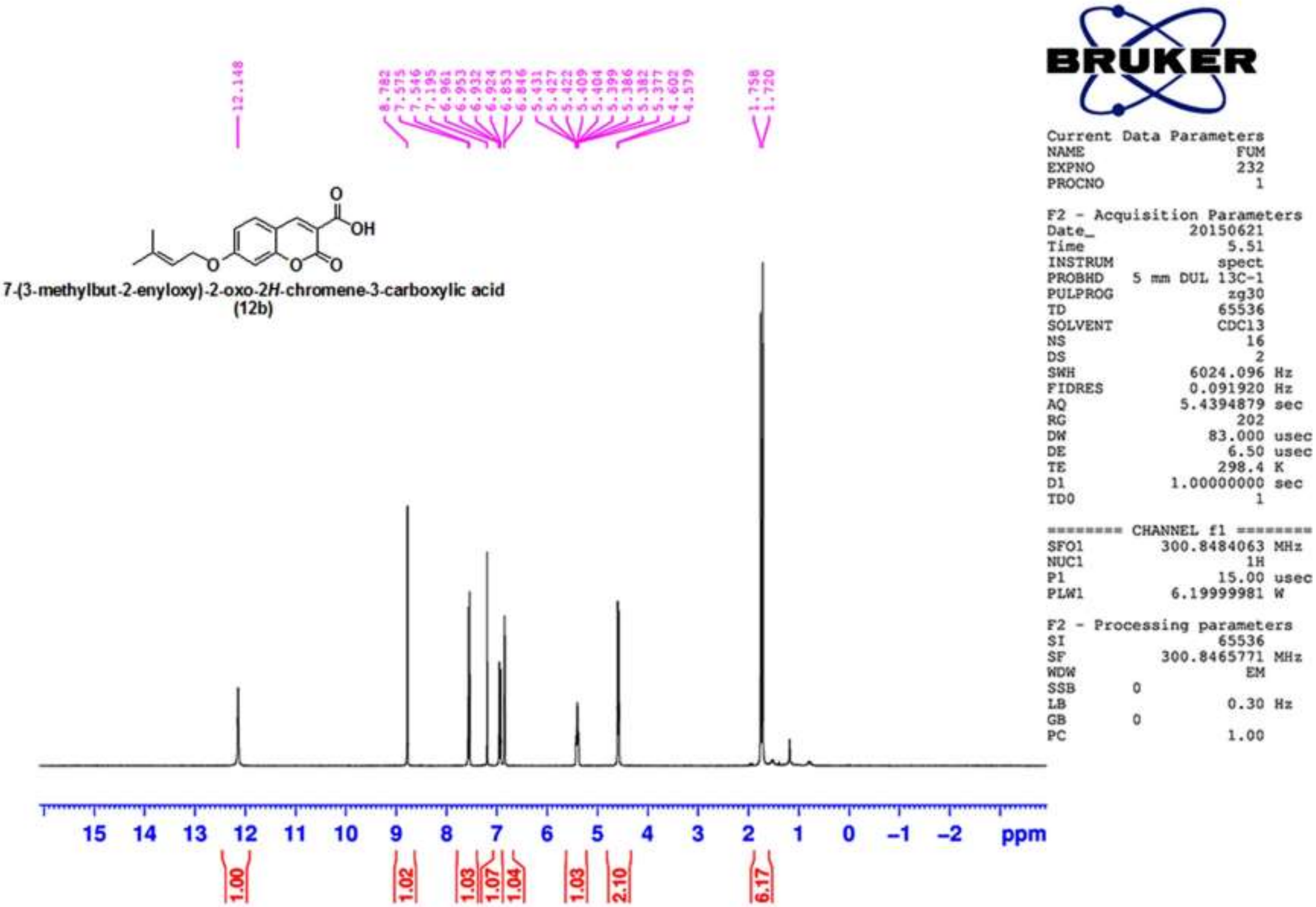
12.148
8.782
7.575
7.546
7.195
6.961
6.953
6.932
6.924
6.853
6.846
5.431
5.427
5.422
5.409
5.404
5.399
5.386
5.382
5.377
4.602
4.579
1.758
1.720
OH
7-(3-methylbut-2-enyloxy)-2-oxo-2H-chromene-3-carboxylic acid
(12b)
15 14 13 12 11 10 9 8 7 6 5 4 3 2 1 0 -1 -2 ppm
1.00
1.02
1.03
1.07
1.04
1.03
2.10
6.17
BRUKER
Current Data Parameters
NAME FUM
EXPNO 232
PROCNO 1
F2 - Acquisition Parameters
Date_ 20150621
Time 5.51
INSTRUM spect
PROBHD 5 mm DUL 13C-1
PULPROG zg30
TD 65536
SOLVENT CDCl3
NS 16
DS 2
SWH 6024.096 Hz
FIDRES 0.091920 Hz
AQ 5.4394879 sec
RG 202
DW 83.000 usec
DE 6.50 usec
TE 298.4 K
D1 1.00000000 sec
TD0 1
======== CHANNEL f1 ========
SFO1 300.8484063 MHz
NUC1 1H
P1 15.00 usec
PLW1 6.19999981 W
F2 - Processing parameters
SI 65536
SF 300.8465771 MHz
WDW EM
SSB 0
LB 0.30 Hz
GB 0
PC 1.00

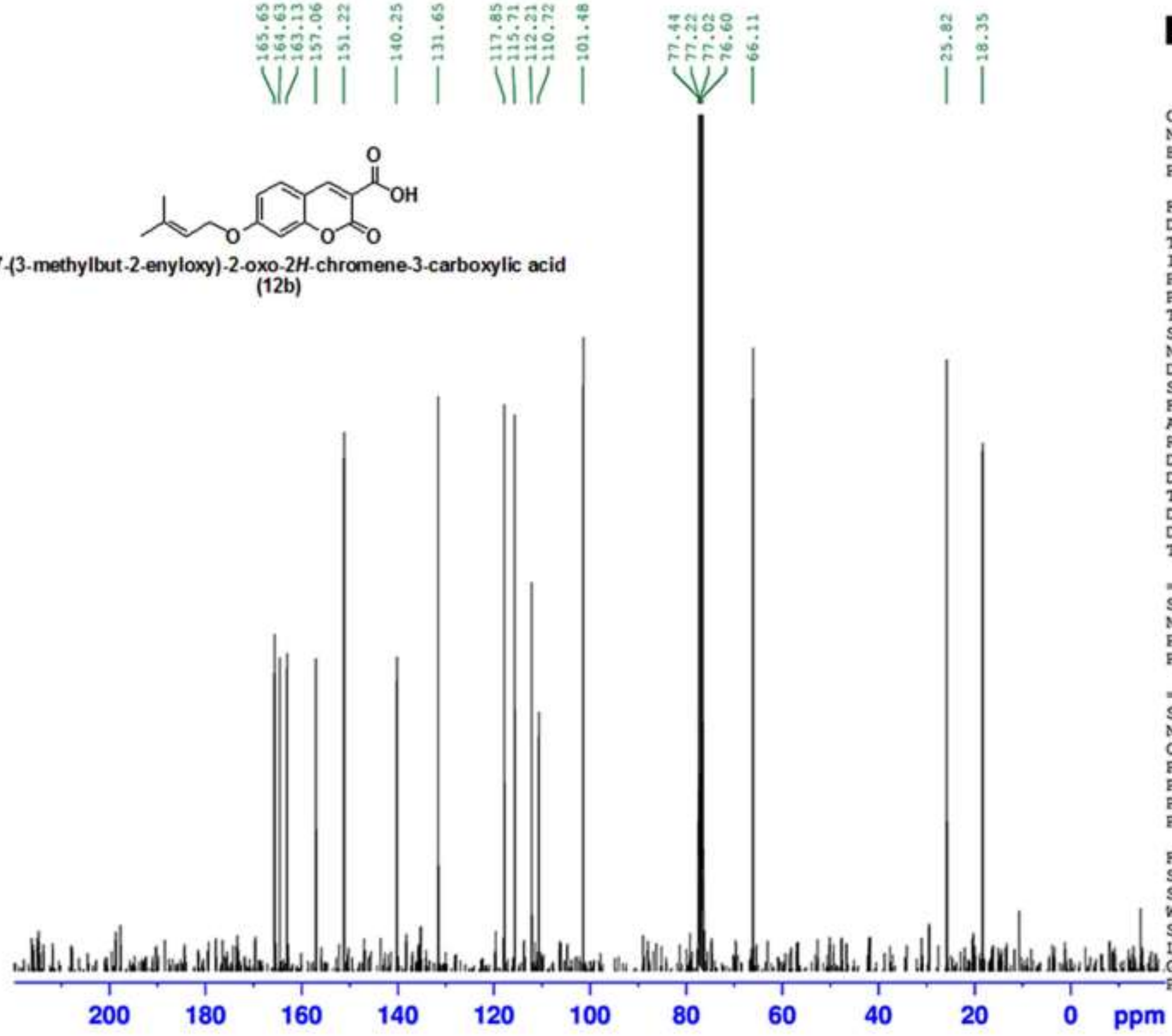

7-(3-methylbut-2-enyloxy)-2-oxo-2*H*-chromene-3-carboxylic acid
(12b)

BRUKER

Current Data Parameters
NAME FUM
EXPNO 242
PROCNO 1

F2 - Acquisition Parameters
Date_ 20150621
Time 11.26
INSTRUM spect
PROBHD 5 mm DUL 13C-1
PULPROG zgpg30
TD 65536
SOLVENT CDCl3
NS 208
DS 4
SWH 18115.941 Hz
FIDRES 0.276427 Hz
AQ 1.8087935 sec
RG 202
DW 27.600 usec
DE 6.50 usec
TE 299.3 K
D1 2.00000000 sec
D11 0.03000000 sec
TD0 1

======== CHANNEL f1 ========
SFO1 75.6554892 MHz
NUC1 13C
P1 10.00 usec
PLW1 20.42000008 W

======== CHANNEL f2 ========
SFO2 300.8477518 MHz
NUC2 1H
CPDPRG[2 waltz16
PCPD2 90.00 usec
PLW2 6.19999981 W
PLW12 0.17222001 W
PLW13 0.13950001 W

F2 - Processing parameters
SI 32768
SF 75.6479250 MHz
WDW EM
SSB 0
LB 1.00 Hz
GB 0
PC 1.40

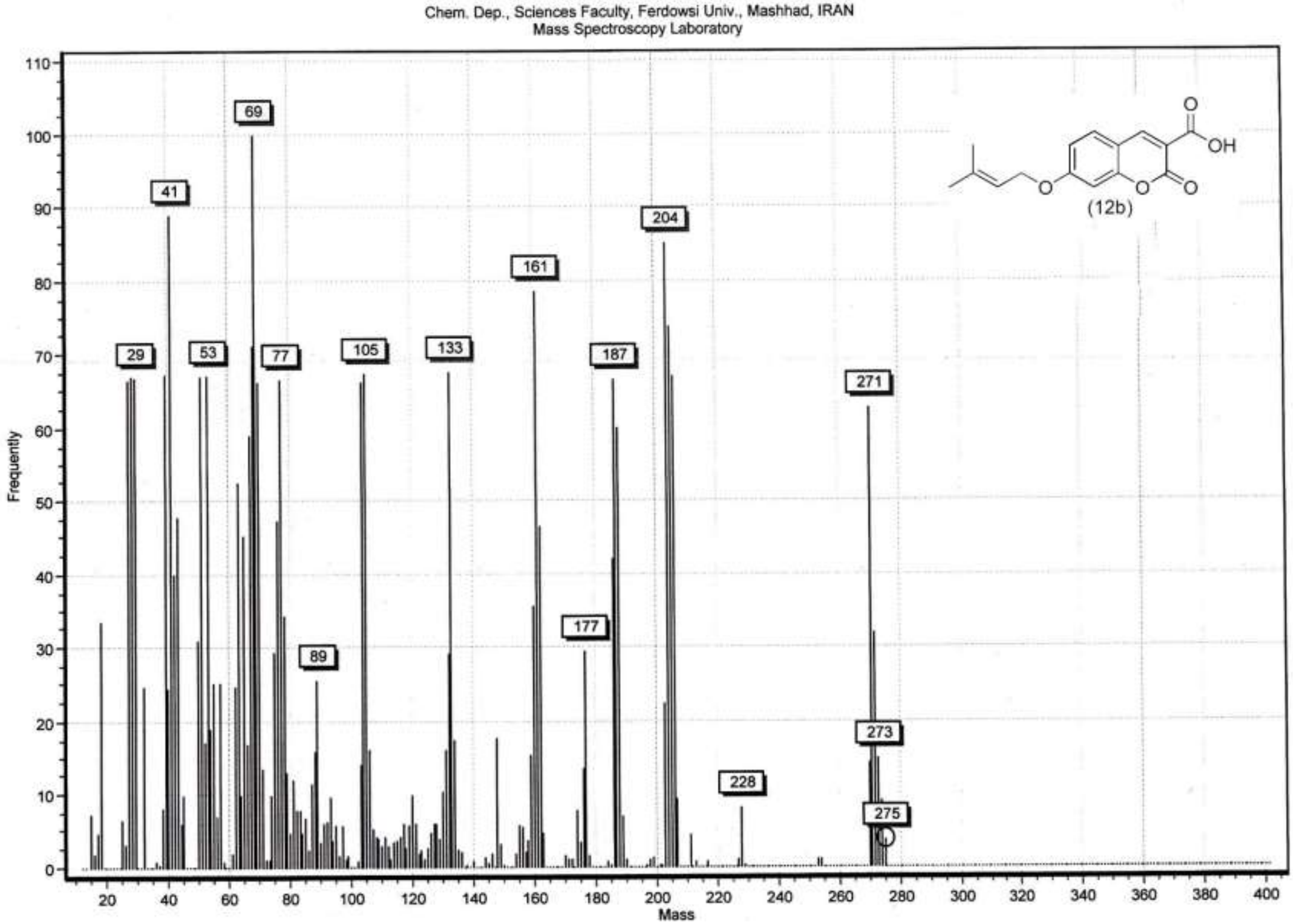
Chem. Dep., Sciences Faculty, Ferdowsi Univ., Mashhad, IRAN
Mass Spectroscopy Laboratory
Frequently
Mass
(12b)
OH
O
69
41
204
161
29
53
77
105
133
187
271
177
89
273
228
275

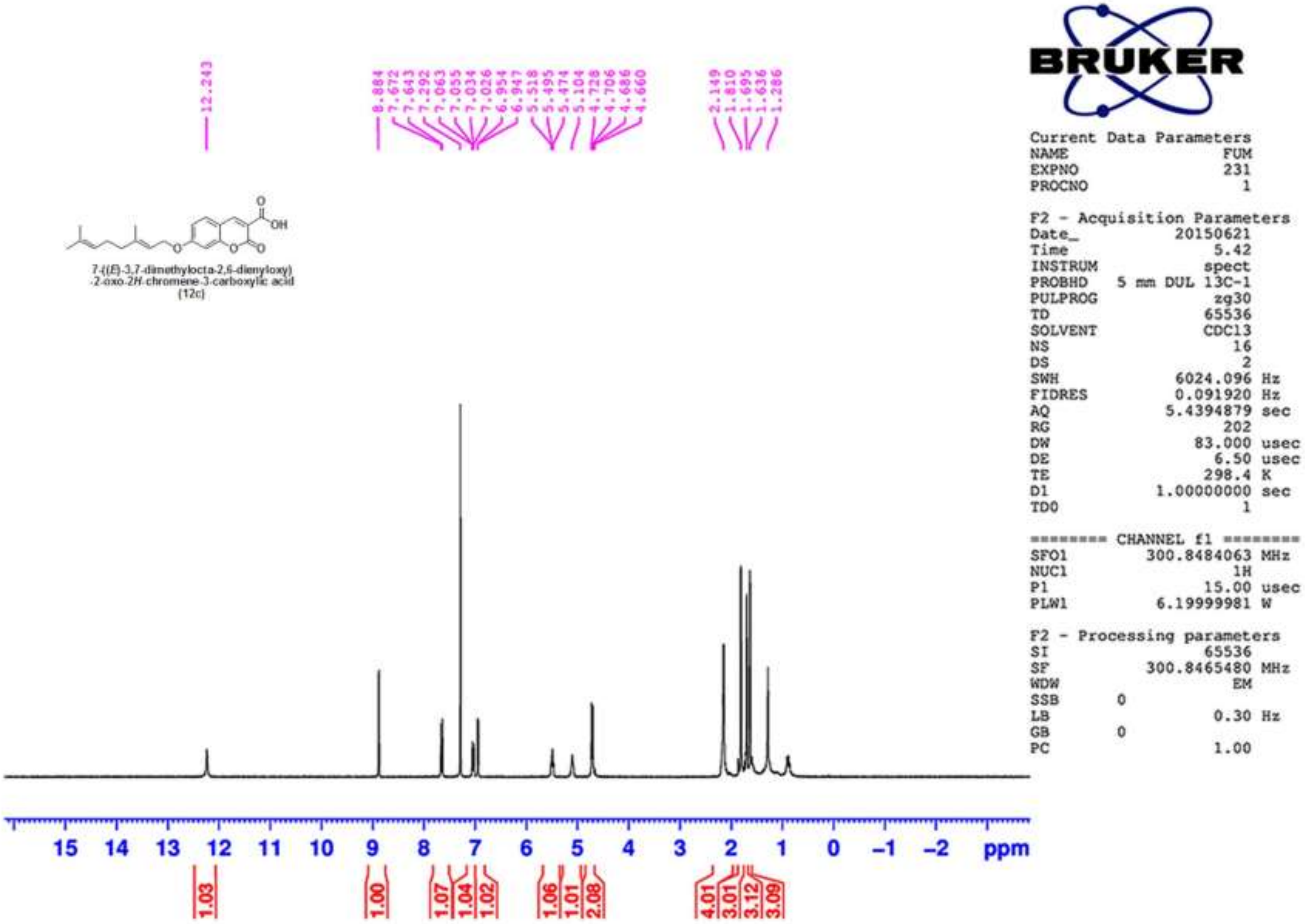
12.243
8.884 7.672 7.643 7.292 7.063 7.055 7.034 7.026 6.954 6.947 5.518 5.495 5.474 5.104 4.728 4.706 4.686 4.660
2.149 1.810 1.695 1.636 1.286
7-((E)-3,7-dimethylocta-2,6-dienyloxy)
-2-oxo-2H-chromene-3-carboxylic acid
(12c)
BRUKER
Current Data Parameters
NAME FUM
EXPNO 231
PROCNO 1
F2 - Acquisition Parameters
Date_ 20150621
Time 5.42
INSTRUM spect
PROBHD 5 mm DUL 13C-1
PULPROG zg30
TD 65536
SOLVENT CDCl3
NS 16
DS 2
SWH 6024.096 Hz
FIDRES 0.091920 Hz
AQ 5.4394879 sec
RG 202
DW 83.000 usec
DE 6.50 usec
TE 298.4 K
D1 1.00000000 sec
TD0 1
======== CHANNEL f1 ========
SFO1 300.8484063 MHz
NUC1 1H
P1 15.00 usec
PLW1 6.19999981 W
F2 - Processing parameters
SI 65536
SF 300.8465480 MHz
WDW EM
SSB 0
LB 0.30 Hz
GB 0
PC 1.00
15 14 13 12 11 10 9 8 7 6 5 4 3 2 1 0 -1 -2 ppm
1.03 1.00 1.07 1.04 1.02 1.06 1.01 2.08 4.01 3.01 3.12 3.09

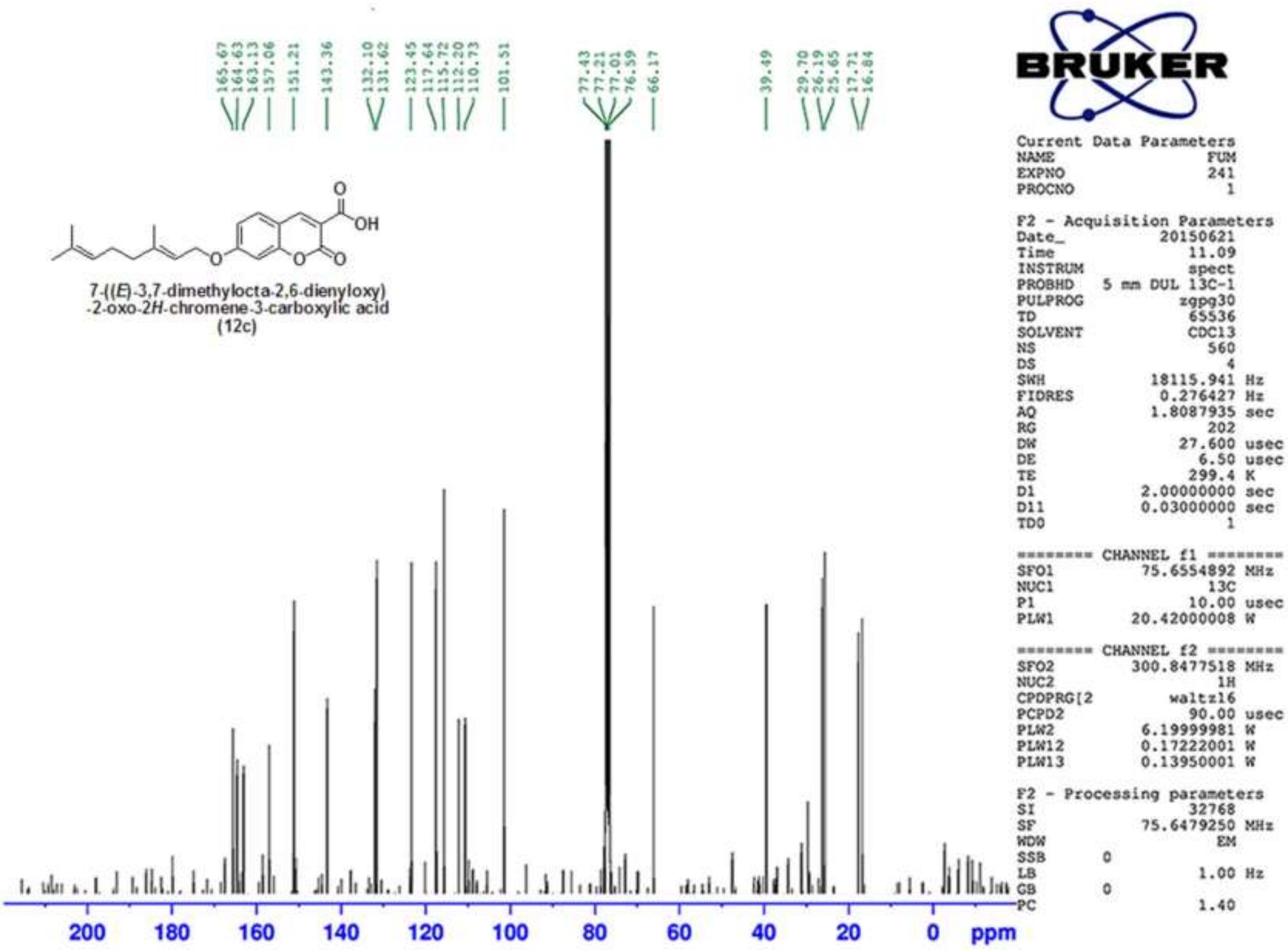
165.67
164.63
163.13
157.06
151.21
143.36
132.10
131.62
123.45
117.64
115.72
112.20
110.73
101.51
77.43
77.21
77.01
76.59
66.17
39.49
29.70
26.19
25.65
17.71
16.84
O
OH
O
O
O
7-((E)-3,7-dimethylocta-2,6-dienyloxy)
-2-oxo-2H-chromene-3-carboxylic acid
(12c)
200 180 160 140 120 100 80 60 40 20 0 ppm
BRUKER
Current Data Parameters
NAME FUM
EXPNO 241
PROCNO 1
F2 - Acquisition Parameters
Date_ 20150621
Time 11.09
INSTRUM spect
PROBHD 5 mm DUL 13C-1
PULPROG zgpg30
TD 65536
SOLVENT CDCl3
NS 560
DS 4
SWH 18115.941 Hz
FIDRES 0.276427 Hz
AQ 1.8087935 sec
RG 202
DW 27.600 usec
DE 6.50 usec
TE 299.4 K
D1 2.00000000 sec
D11 0.03000000 sec
TD0 1
======== CHANNEL f1 ========
SFO1 75.6554892 MHz
NUC1 13C
P1 10.00 usec
PLW1 20.42000008 W
======== CHANNEL f2 ========
SFO2 300.8477518 MHz
NUC2 1H
CPDPRG[2 waltz16
PCPD2 90.00 usec
PLW2 6.19999981 W
PLW12 0.17222001 W
PLW13 0.13950001 W
F2 - Processing parameters
SI 32768
SF 75.6479250 MHz
WDW EM
SSB 0
LB 1.00 Hz
GB 0
PC 1.40

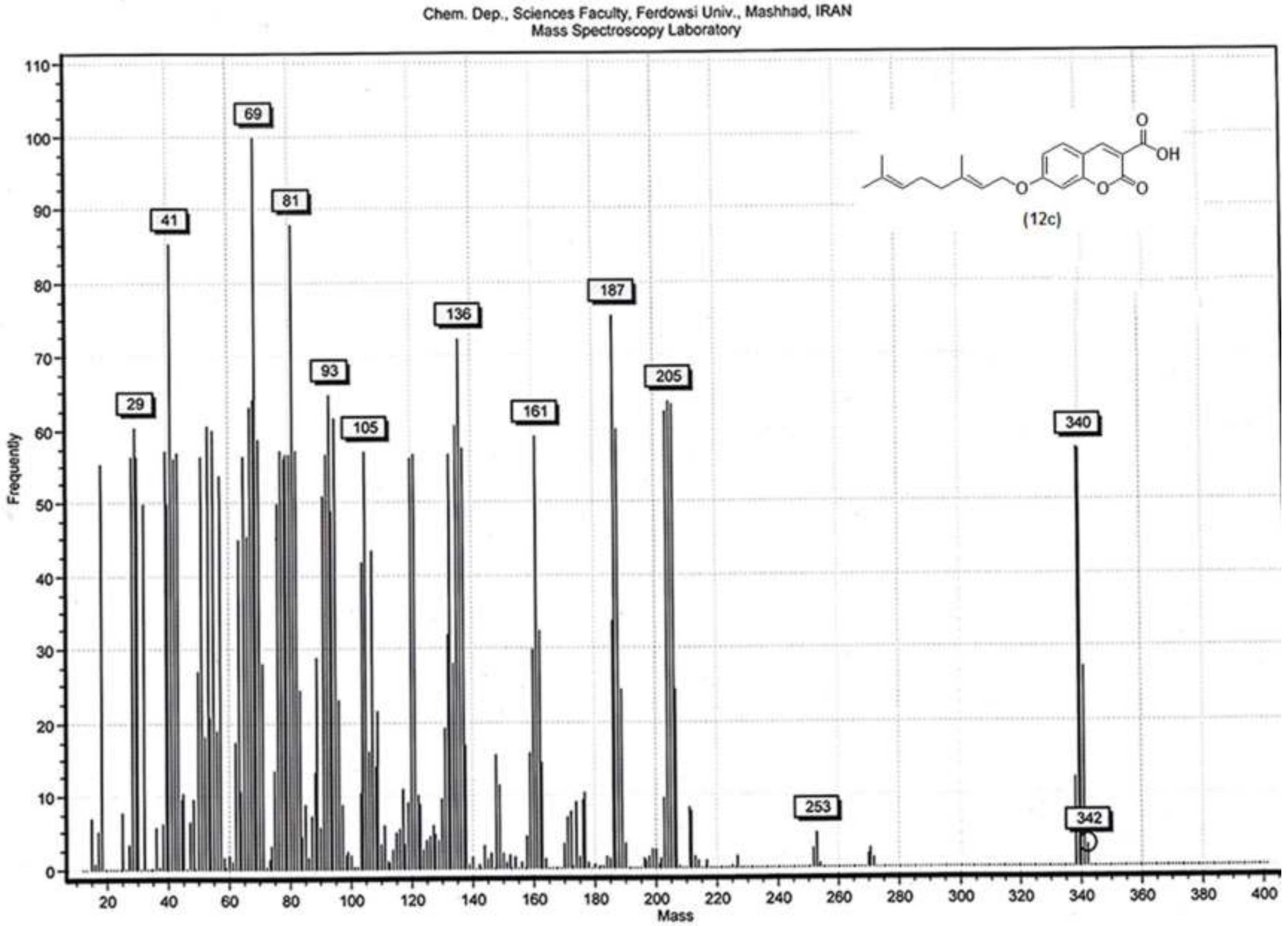
Chem. Dep., Sciences Faculty, Ferdowsi Univ., Mashhad, IRAN
Mass Spectroscopy Laboratory
(12c)
O
OH
O
O
O
Frequently
Mass
29
41
69
81
93
105
136
161
187
205
253
340
342
0
10
20
30
40
50
60
70
80
90
100
110
20
40
60
80
100
120
140
160
180
200
220
240
260
280
300
320
340
360
380
400

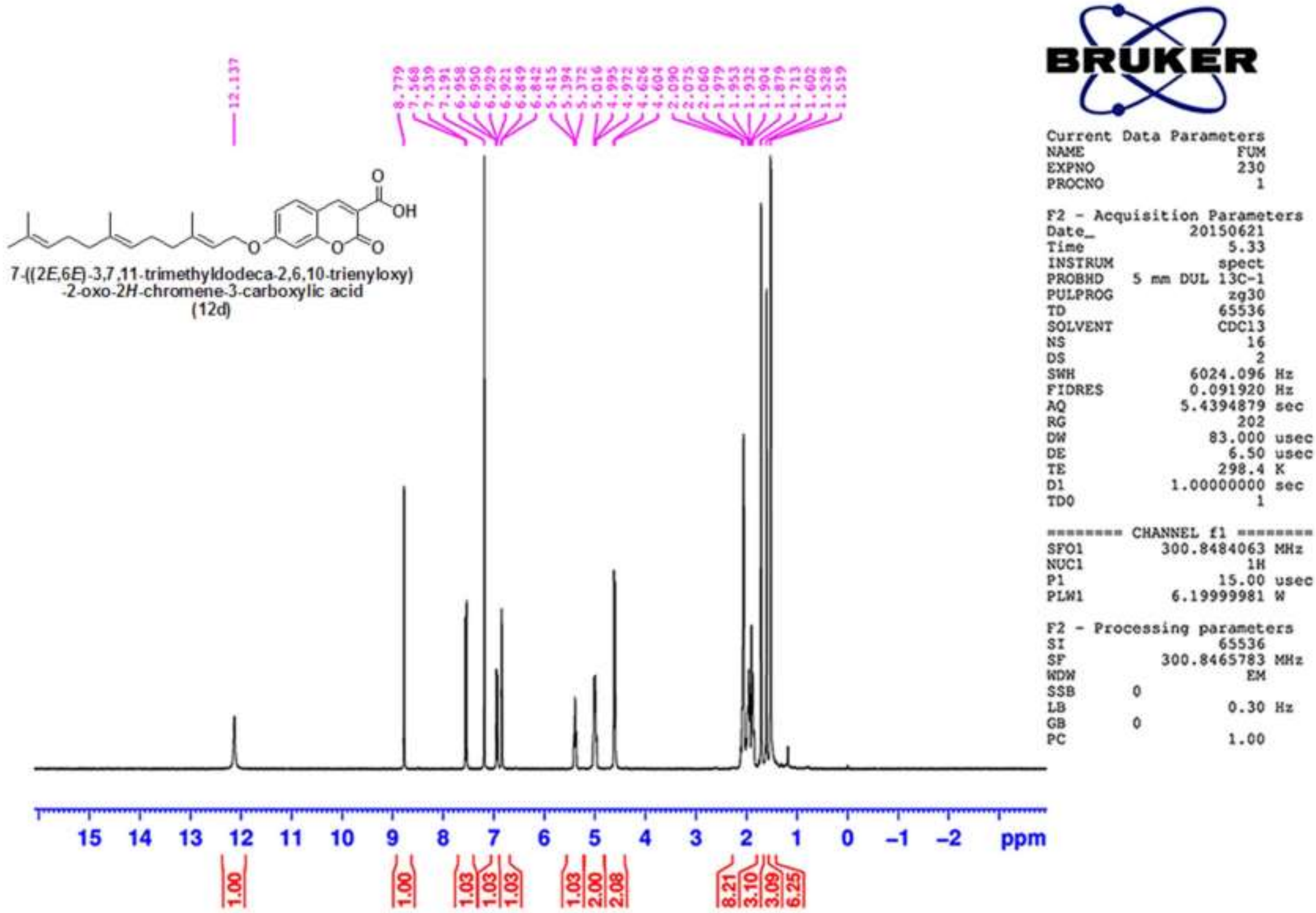
7-((2E,6E)-3,7,11-trimethyldodeca-2,6,10-trienyloxy)
-2-oxo-2H-chromene-3-carboxylic acid
(12d)
BRUKER
Current Data Parameters
NAME FUM
EXPNO 230
PROCNO 1
F2 - Acquisition Parameters
Date_ 20150621
Time 5.33
INSTRUM spect
PROBHD 5 mm DUL 13C-1
PULPROG zg30
TD 65536
SOLVENT CDCl3
NS 16
DS 2
SWH 6024.096 Hz
FIDRES 0.091920 Hz
AQ 5.4394879 sec
RG 202
DW 83.000 usec
DE 6.50 usec
TE 298.4 K
D1 1.00000000 sec
TD0 1
======== CHANNEL f1 ========
SFO1 300.8484063 MHz
NUC1 1H
P1 15.00 usec
PLW1 6.19999981 W
F2 - Processing parameters
SI 65536
SF 300.8465783 MHz
WDW EM
SSB 0
LB 0.30 Hz
GB 0
PC 1.00
ppm

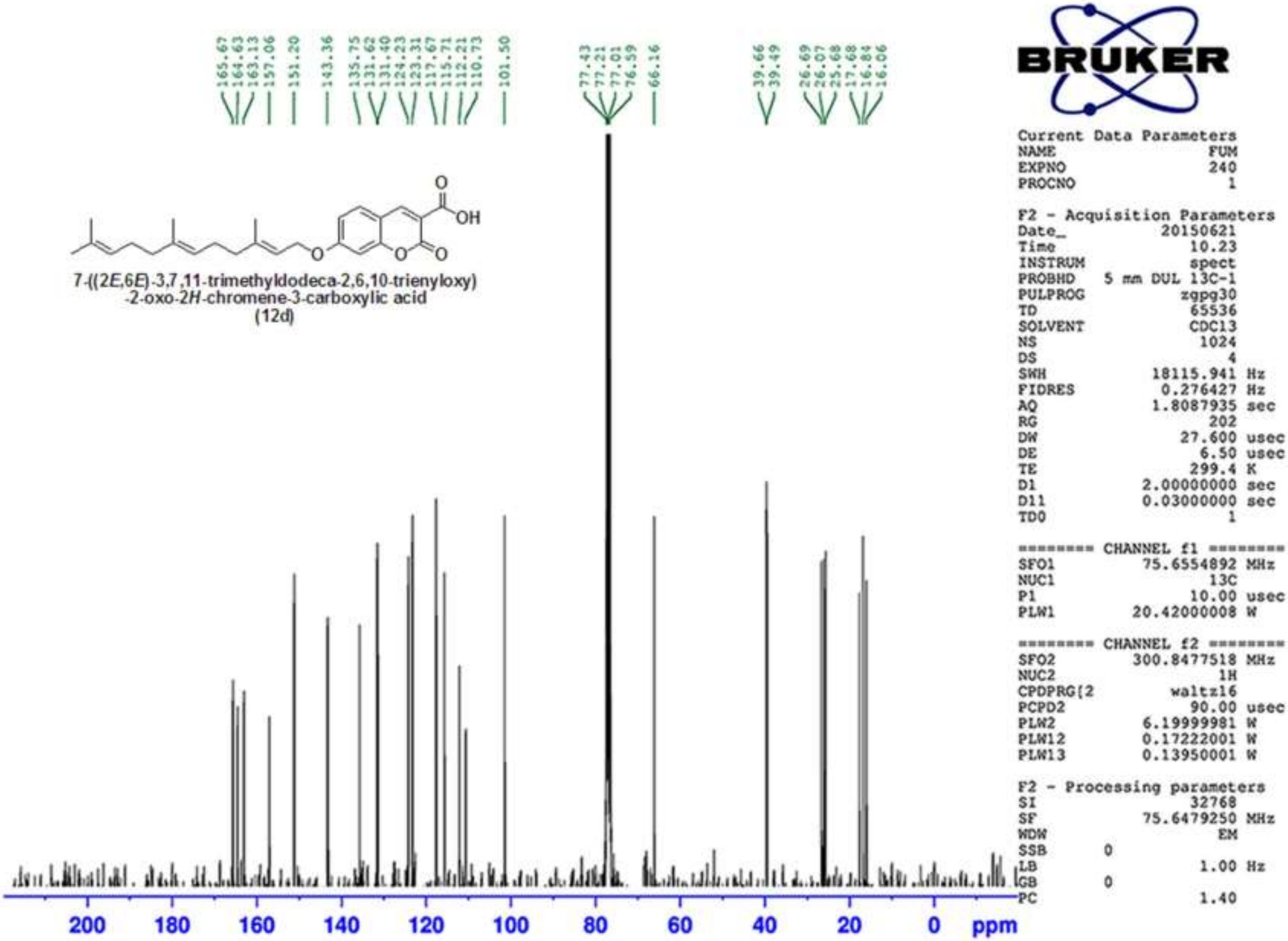
165.67
164.63
163.13
157.06
151.20
143.36
135.75
131.62
131.40
124.23
123.31
117.67
115.71
112.21
110.73
101.50
77.43
77.21
77.01
76.59
66.16
39.66
39.49
26.69
26.07
25.68
17.68
16.84
16.06
O
OH
O
O
O
7-((2E,6E)-3,7,11-trimethyldodeca-2,6,10-trienyloxy)
-2-oxo-2H-chromene-3-carboxylic acid
(12d)
BRUKER
Current Data Parameters
NAME FUM
EXPNO 240
PROCNO 1
F2 - Acquisition Parameters
Date_ 20150621
Time 10.23
INSTRUM spect
PROBHD 5 mm DUL 13C-1
PULPROG zgpg30
TD 65536
SOLVENT CDCl3
NS 1024
DS 4
SWH 18115.941 Hz
FIDRES 0.276427 Hz
AQ 1.8087935 sec
RG 202
DW 27.600 usec
DE 6.50 usec
TE 299.4 K
D1 2.00000000 sec
D11 0.03000000 sec
TD0 1
======== CHANNEL f1 ========
SFO1 75.6554892 MHz
NUC1 13C
P1 10.00 usec
PLW1 20.42000008 W
======== CHANNEL f2 ========
SFO2 300.8477518 MHz
NUC2 1H
CPDPRG[2 waltz16
PCPD2 90.00 usec
PLW2 6.19999981 W
PLW12 0.17222001 W
PLW13 0.13950001 W
F2 - Processing parameters
SI 32768
SF 75.6479250 MHz
WDW EM
SSB 0
LB 1.00 Hz
GB 0
PC 1.40
200
180
160
140
120
100
80
60
40
20
0
ppm

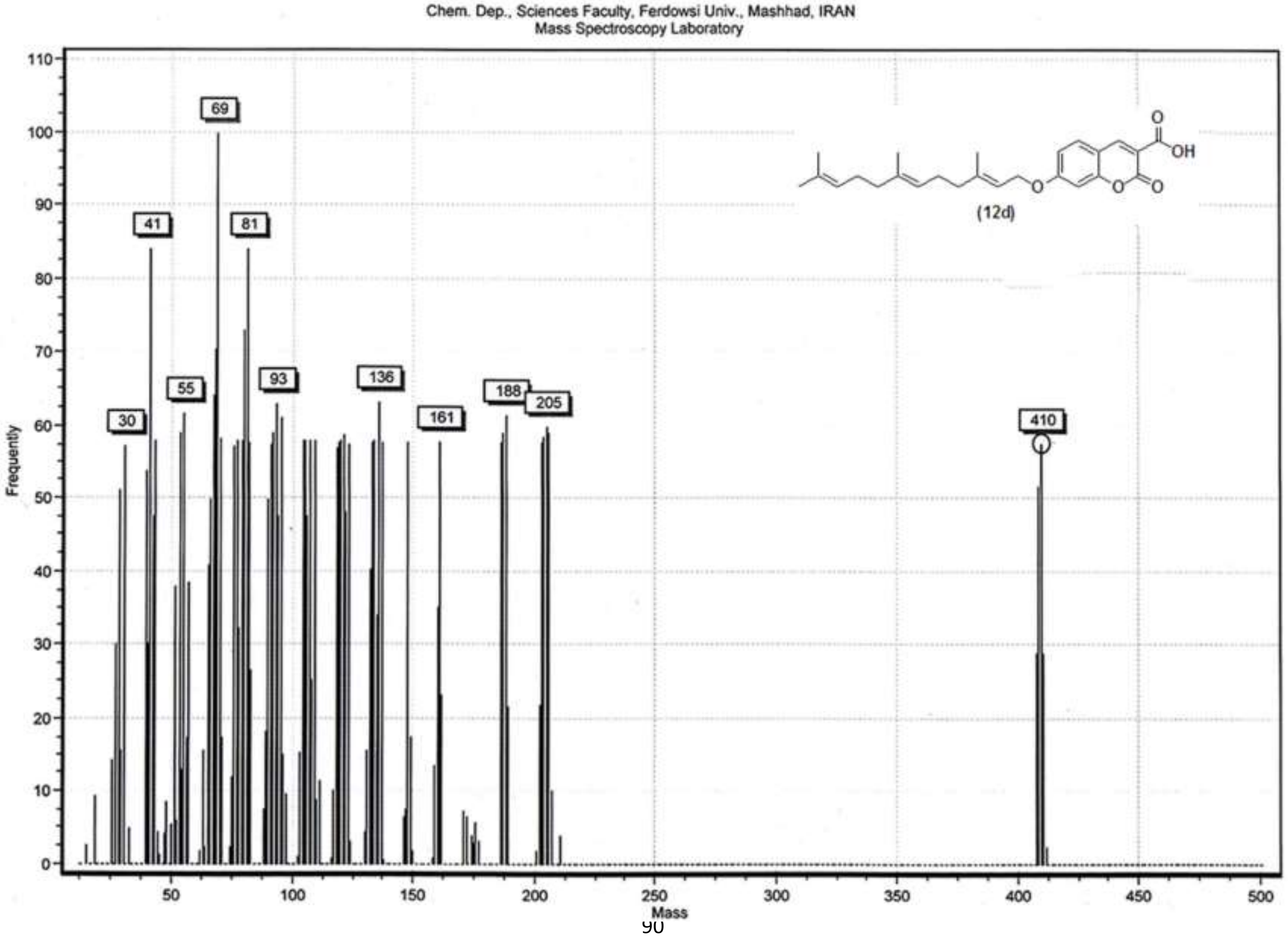
Chem. Dep., Sciences Faculty, Ferdowsi Univ., Mashhad, IRAN
Mass Spectroscopy Laboratory
(12d)
69
41
81
55
93
136
30
161
188
205
410
Frequently
Mass

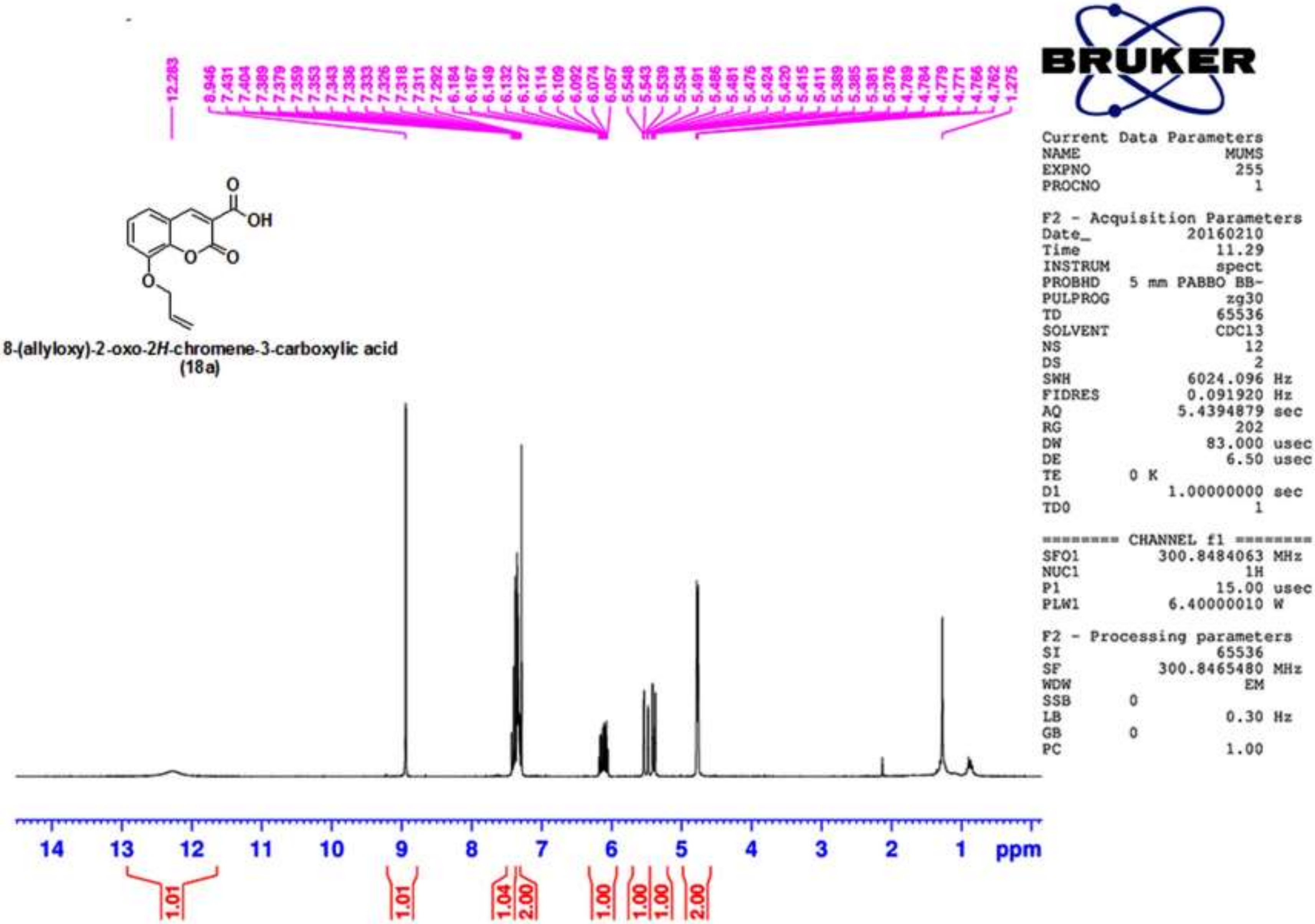
12.283
8.946
7.431
7.404
7.389
7.379
7.359
7.353
7.343
7.336
7.333
7.326
7.318
7.311
7.292
6.184
6.167
6.149
6.132
6.127
6.114
6.109
6.092
6.074
6.057
5.548
5.543
5.539
5.534
5.491
5.486
5.481
5.476
5.424
5.420
5.415
5.411
5.389
5.385
5.381
5.376
4.789
4.784
4.779
4.771
4.766
4.762
1.275
OH
8-(allyloxy)-2-oxo-2H-chromene-3-carboxylic acid
(18a)
BRUKER
Current Data Parameters
NAME MUMS
EXPNO 255
PROCNO 1
F2 - Acquisition Parameters
Date_ 20160210
Time 11.29
INSTRUM spect
PROBHD 5 mm PABBO BB-
PULPROG zg30
TD 65536
SOLVENT CDCl3
NS 12
DS 2
SWH 6024.096 Hz
FIDRES 0.091920 Hz
AQ 5.4394879 sec
RG 202
DW 83.000 usec
DE 6.50 usec
TE 0 K
D1 1.00000000 sec
TD0 1
======== CHANNEL f1 ========
SFO1 300.8484063 MHz
NUC1 1H
P1 15.00 usec
PLW1 6.40000010 W
F2 - Processing parameters
SI 65536
SF 300.8465480 MHz
WDW EM
SSB 0
LB 0.30 Hz
GB 0
PC 1.00
14 13 12 11 10 9 8 7 6 5 4 3 2 1 ppm
1.01
1.01
1.04
2.00
1.00
1.00
1.00
2.00

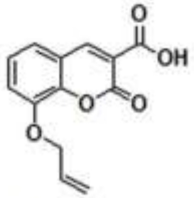

8-(allyloxy)-2-oxo-2*H*-chromene-3-carboxylic acid
(18a)

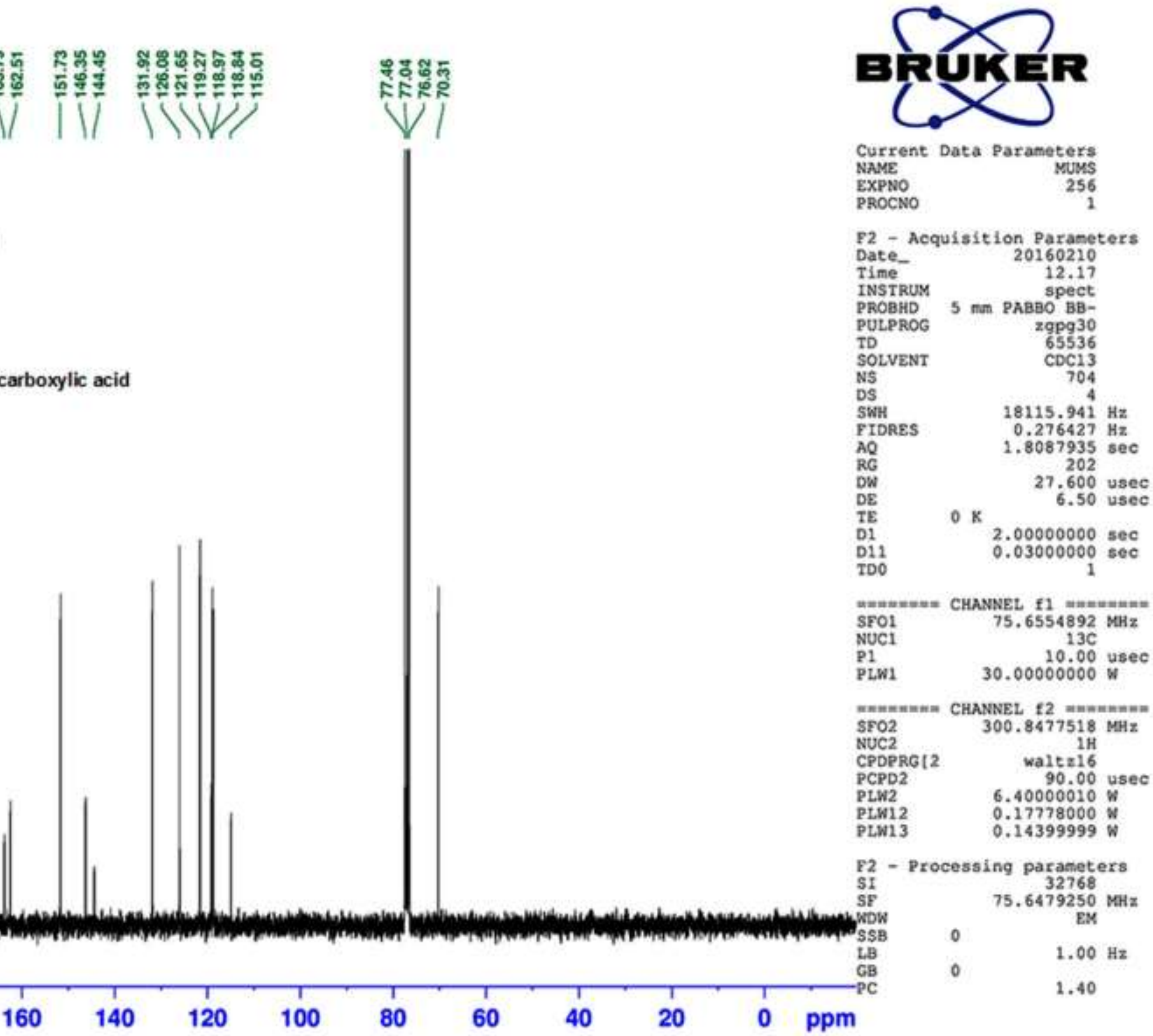

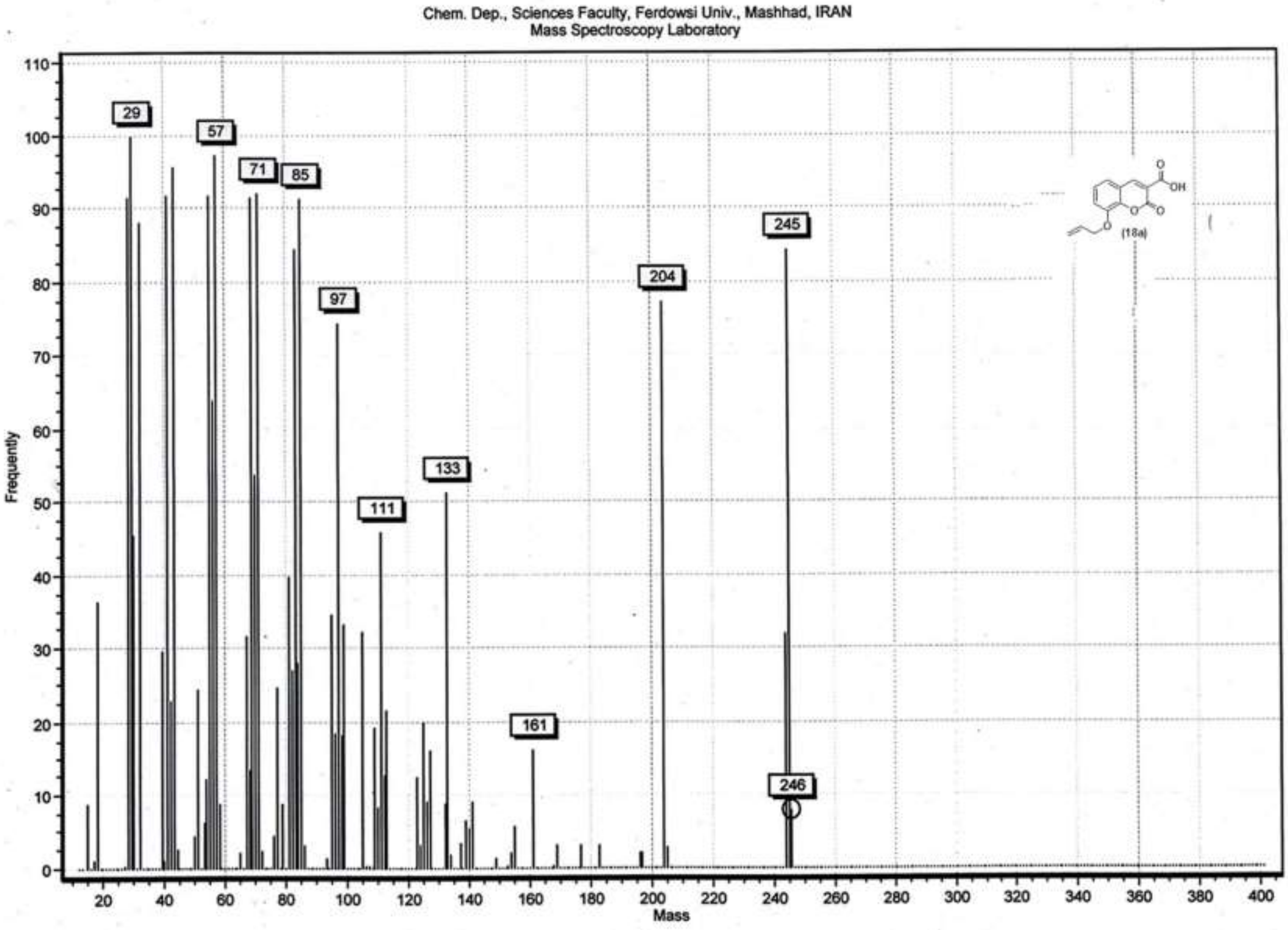
Chem. Dep., Sciences Faculty, Ferdowsi Univ., Mashhad, IRAN
Mass Spectroscopy Laboratory
Frequently
Mass
29
57
71
85
97
111
133
161
204
245
246
(18a)

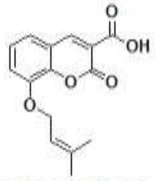

8-(3-methylbut-2-enyloxy)-2-oxo-2*H*-chromene-3-carboxylic acid
(18b)

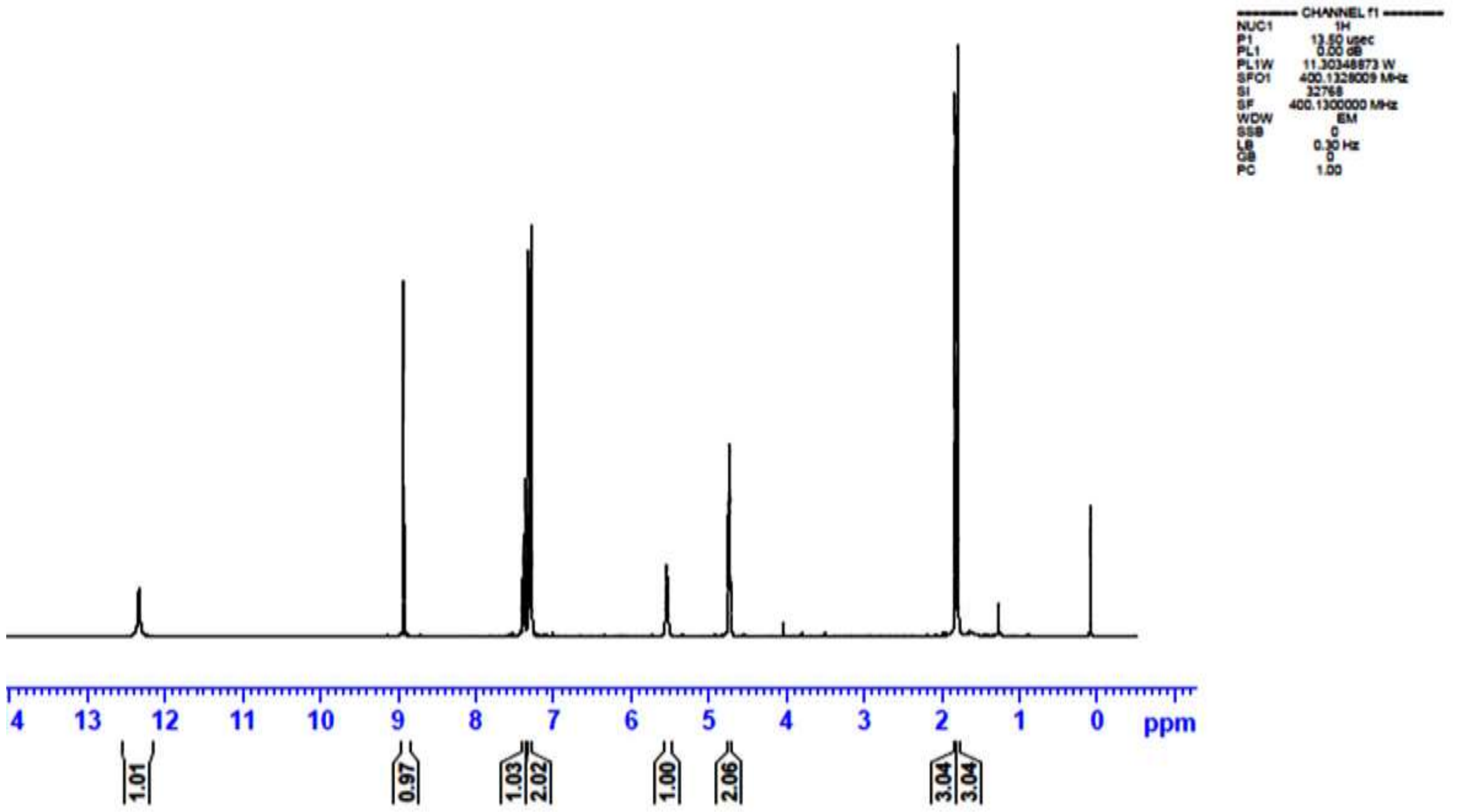

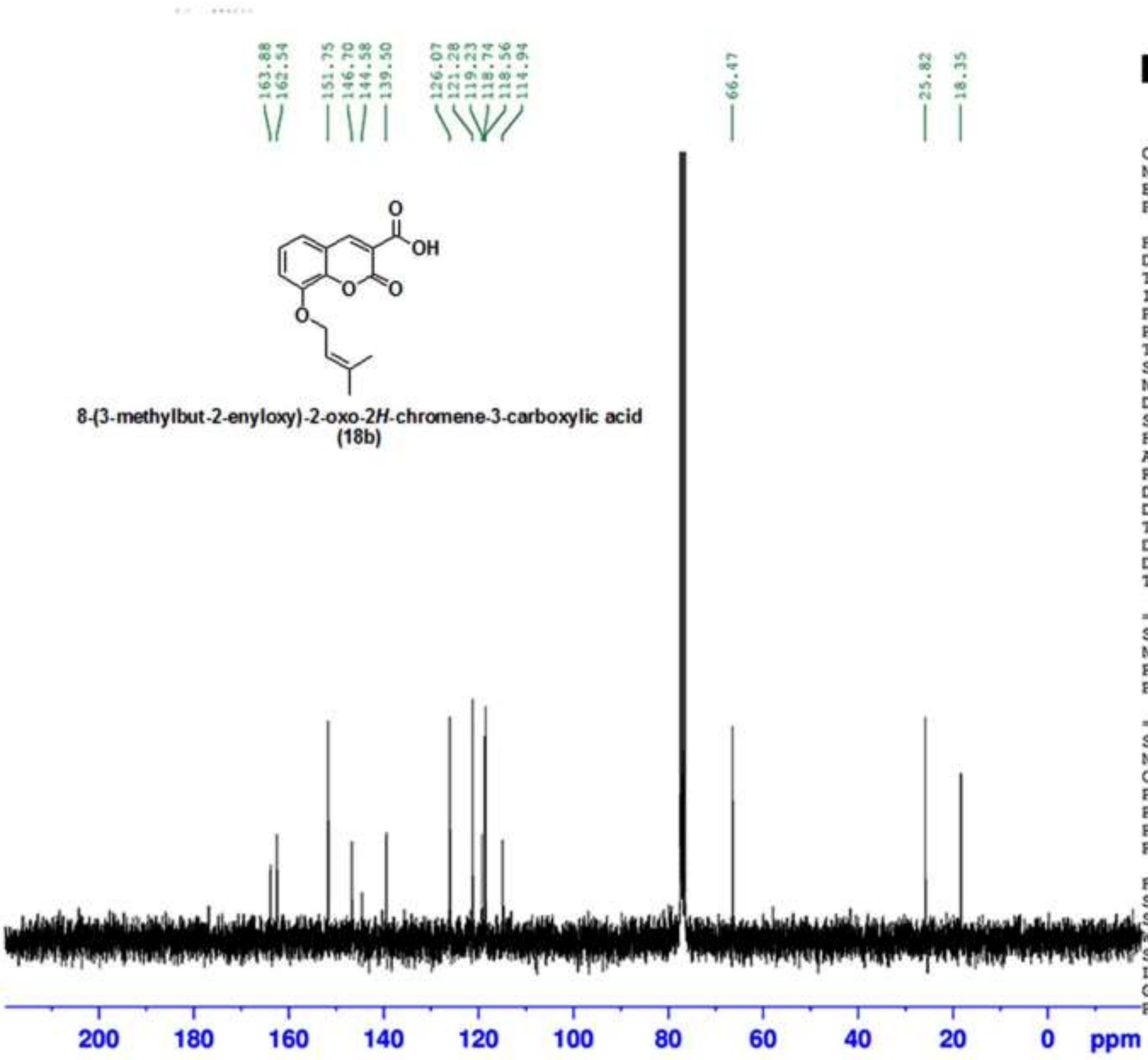

8-(3-methylbut-2-enyloxy)-2-oxo-2*H*-chromene-3-carboxylic acid
(18b)

```
Current Data Parameters
NAME                FUM
EXPNO              1166
PROCNO                1

F2 - Acquisition Parameters
Date_          20150719
Time              10.23
INSTRUM           spect
PROBHD    5 mm DUL 13C-1
PULPROG          zgpg30
TD                65536
SOLVENT           CDCl3
NS                 1024
DS                    4
SWH           18115.941 Hz
FIDRES         0.276427 Hz
AQ            1.8087935 sec
RG                  202
DW               27.600 usec
DE                 6.50 usec
TE                296.4 K
D1           2.00000000 sec
D11          0.03000000 sec
TD0                   1

======== CHANNEL f1 ========
SFO1         75.6554892 MHz
NUC1                13C
P1                10.00 usec
PLW1        20.42000008 W

======== CHANNEL f2 ========
SFO2        300.8477518 MHz
NUC2                 1H
CPDPRG[2        waltz16
PCPD2             90.00 usec
PLW2         6.19999981 W
PLW12        0.17222001 W
PLW13        0.13950001 W

F2 - Processing parameters
SI                32768
SF           75.6479250 MHz
WDW                  EM
SSB      0
LB                 1.00 Hz
GB       0
PC                 1.40
```

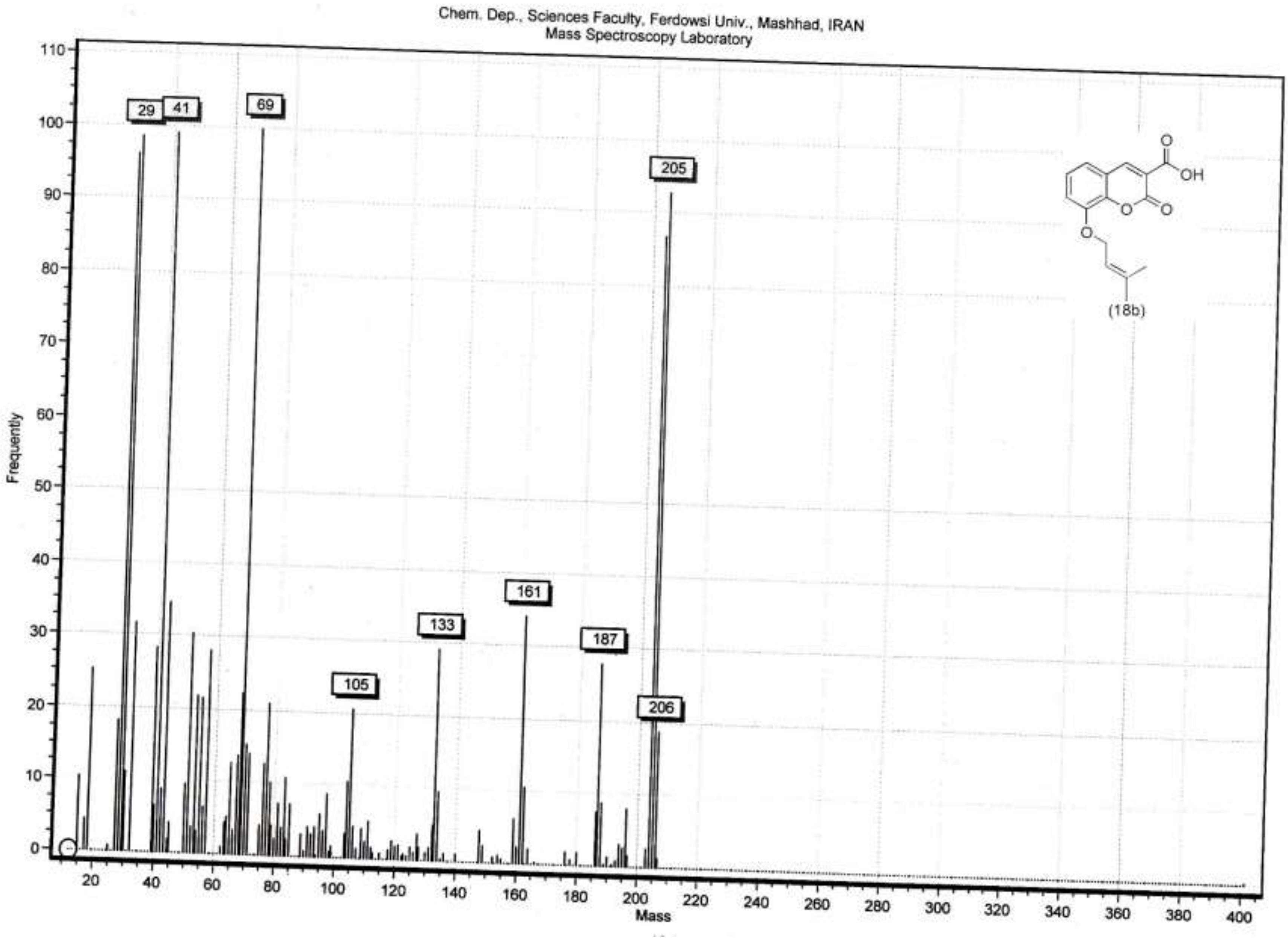

Chem. Dep., Sciences Faculty, Ferdowsi Univ., Mashhad, IRAN
Mass Spectroscopy Laboratory
Frequently
Mass
29
41
69
205
105
133
161
187
206
OH
O
(18b)

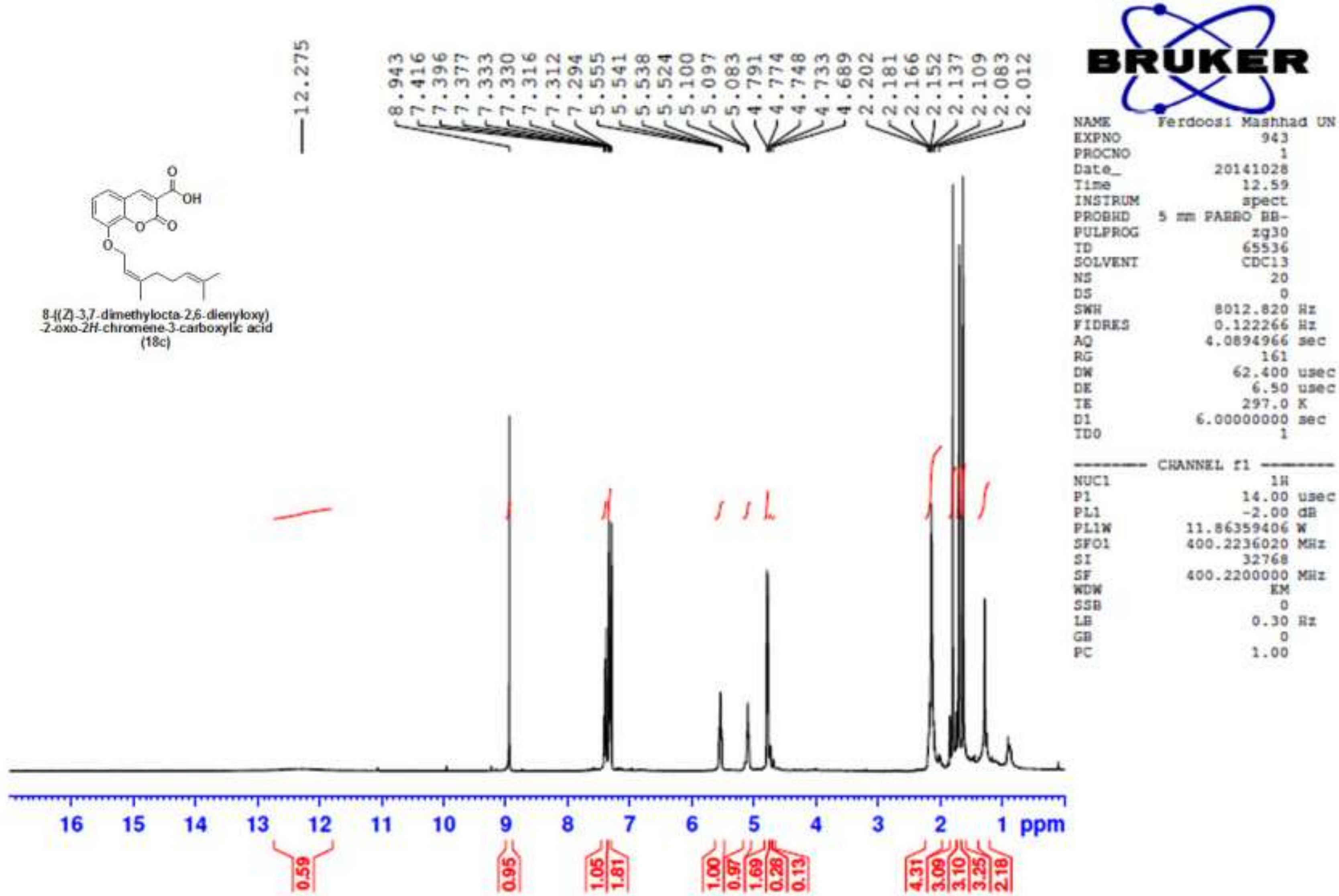
8-((Z)-3,7-dimethylocta-2,6-dienyloxy)
-2-oxo-2H-chromene-3-carboxylic acid
(18c)
12.275
8.943
7.416
7.396
7.377
7.333
7.330
7.316
7.312
7.294
5.555
5.541
5.538
5.524
5.100
5.097
5.083
4.791
4.774
4.748
4.733
4.689
2.202
2.181
2.166
2.152
2.137
2.109
2.083
2.012
BRUKER
NAME Ferdoosi Mashhad UN
EXPNO 943
PROCNO 1
Date_ 20141028
Time 12.59
INSTRUM spect
PROBHD 5 mm PABBO BB-
PULPROG zg30
TD 65536
SOLVENT CDCl3
NS 20
DS 0
SWH 8012.820 Hz
FIDRES 0.122266 Hz
AQ 4.0894966 sec
RG 161
DW 62.400 usec
DE 6.50 usec
TE 297.0 K
D1 6.00000000 sec
TD0 1
CHANNEL f1
NUC1 1H
P1 14.00 usec
PL1 -2.00 dB
PL1W 11.86359406 W
SFO1 400.2236020 MHz
SI 32768
SF 400.2200000 MHz
WDW EM
SSB 0
LB 0.30 Hz
GB 0
PC 1.00
16 15 14 13 12 11 10 9 8 7 6 5 4 3 2 1 ppm
0.59
0.95
1.05
1.81
1.00
0.97
1.69
0.28
0.13
4.31
3.09
3.10
3.25
2.18

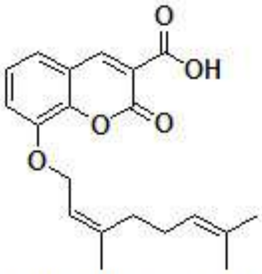

8-((*Z*)-3,7-dimethylocta-2,6-dienyloxy)
-2-oxo-2*H*-chromene-3-carboxylic acid
(18c)

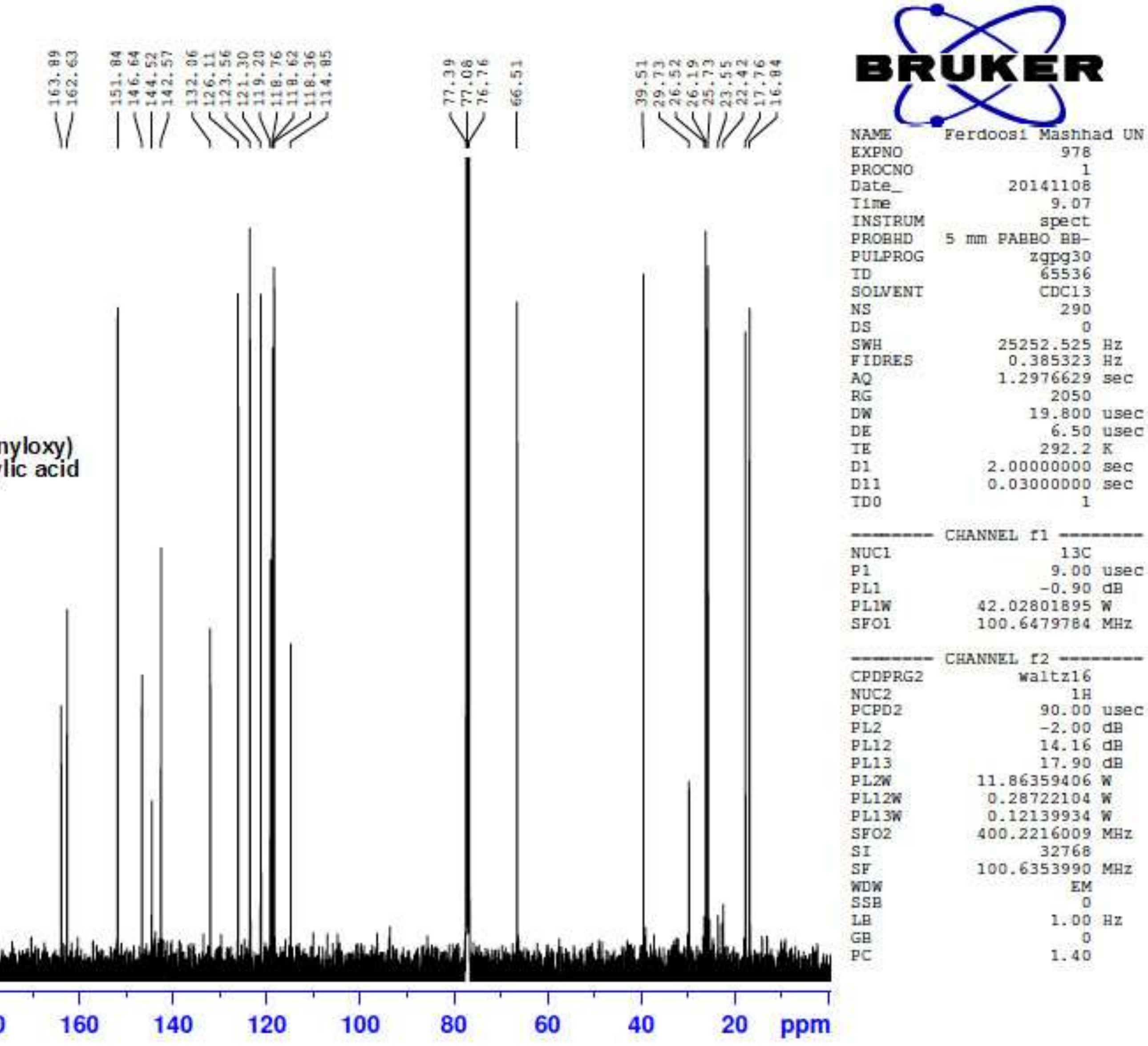

Chem. Dep., Sciences Faculty, Ferdowsi Univ., Mashhad, IRAN
Mass Spectroscopy Laboratory

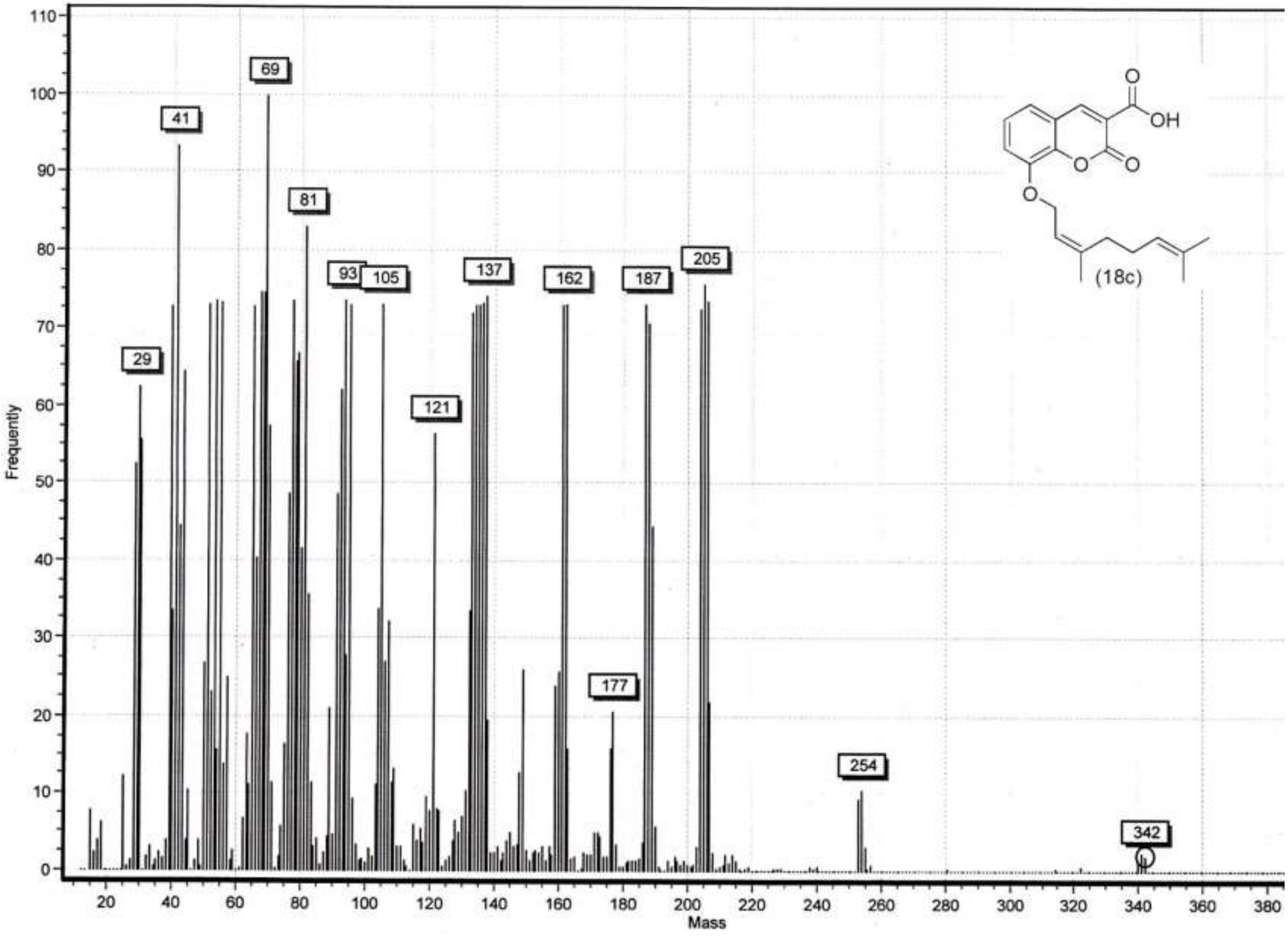

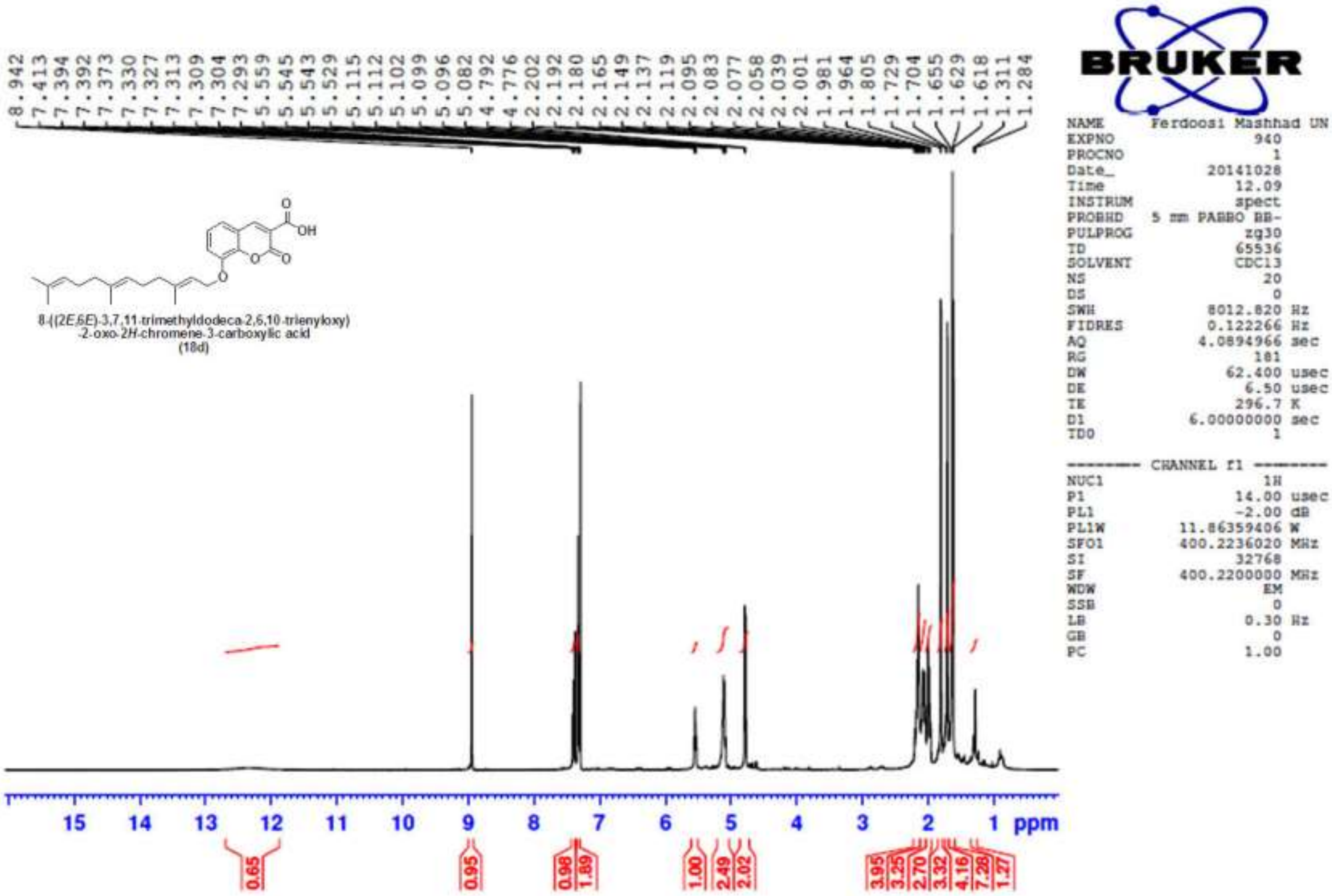

8-((2E,6E)-3,7,11-trimethyldodeca-2,6,10-trienyloxy)-2-oxo-2H-chromene-3-carboxylic acid
(18d)
NAME Ferdoosi Mashhad UN
EXPNO 940
PROCNO 1
Date_ 20141028
Time 12.09
INSTRUM spect
PROBHD 5 mm PABBO BB-
PULPROG zg30
TD 65536
SOLVENT CDCl3
NS 20
DS 0
SWH 8012.820 Hz
FIDRES 0.122266 Hz
AQ 4.0894966 sec
RG 181
DW 62.400 usec
DE 6.50 usec
TE 296.7 K
D1 6.00000000 sec
TD0 1
CHANNEL f1
NUC1 1H
P1 14.00 usec
PL1 -2.00 dB
PL1W 11.86359406 W
SFO1 400.2236020 MHz
SI 32768
SF 400.2200000 MHz
WDW EM
SSB 0
LB 0.30 Hz
GB 0
PC 1.00
ppm

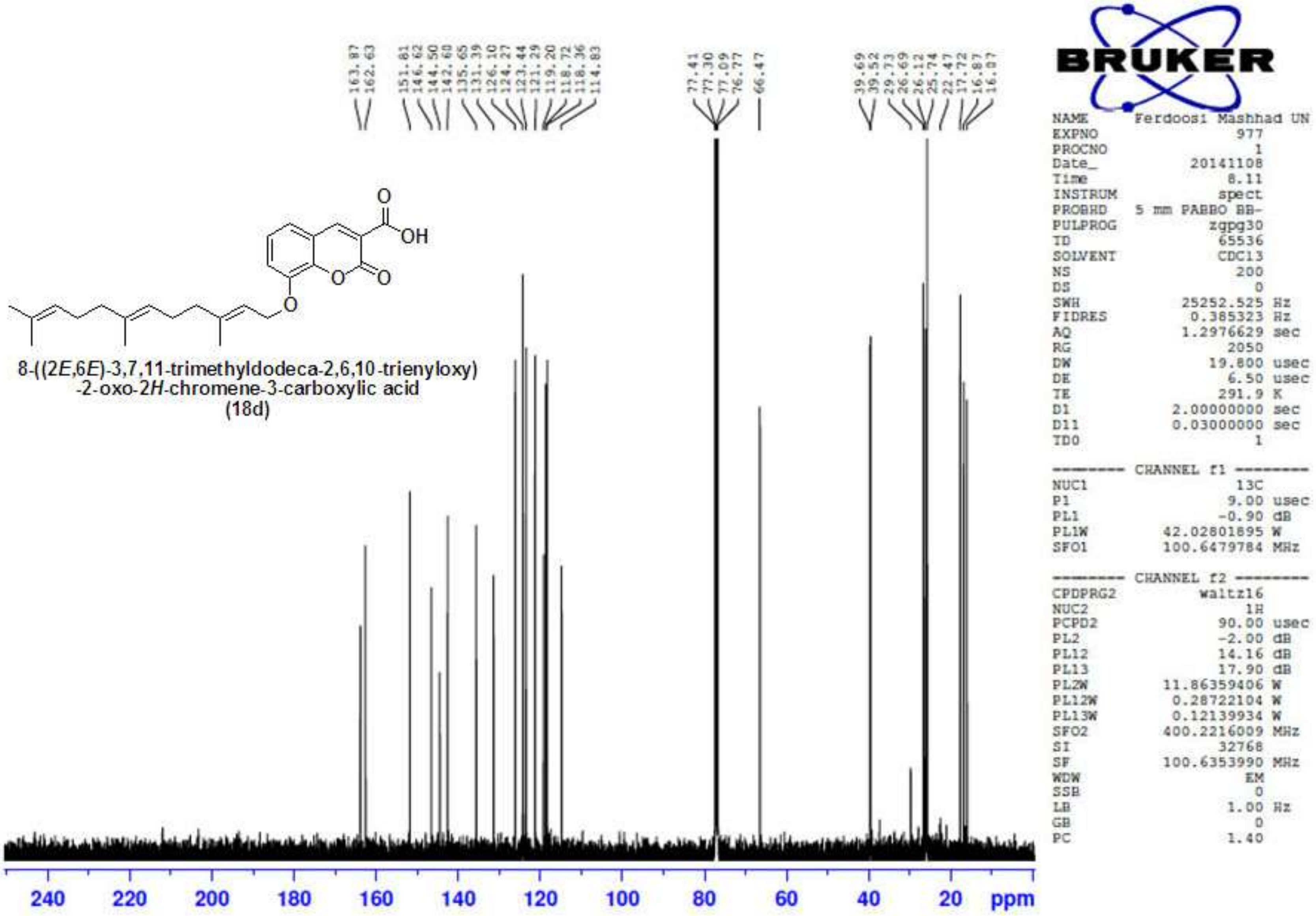

8-((2*E*,6*E*)-3,7,11-trimethyldodeca-2,6,10-trienyloxy)
-2-oxo-2*H*-chromene-3-carboxylic acid
(18d)

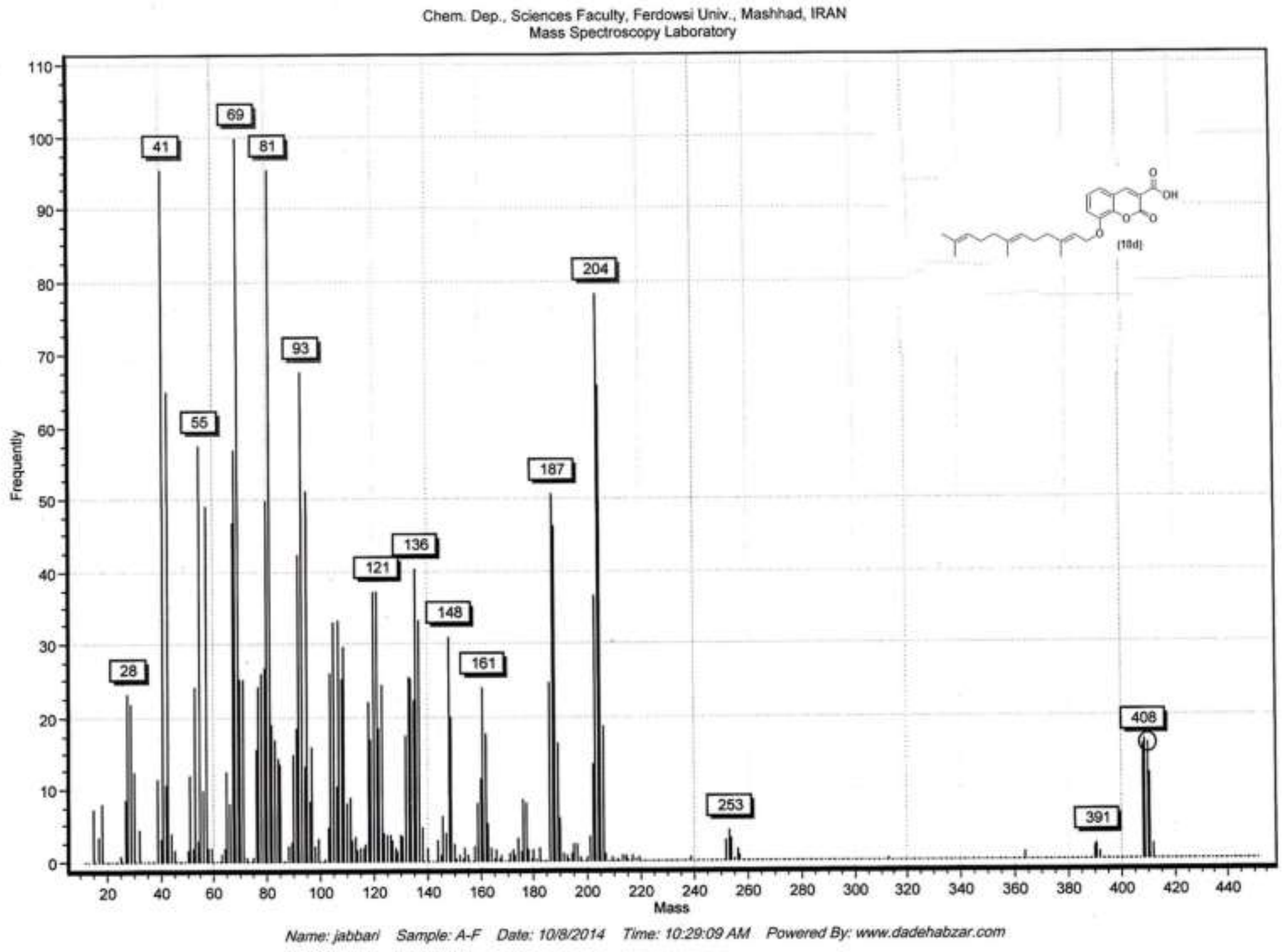
Chem. Dep., Sciences Faculty, Ferdowsi Univ., Mashhad, IRAN
Mass Spectroscopy Laboratory
Frequently
Mass
41
69
81
55
93
28
121
136
148
161
187
204
253
391
408
(18d)
Name: jabbari Sample: A-F Date: 10/8/2014 Time: 10:29:09 AM Powered By: www.dadehabzar.com
